# Global burden of disease among adolescents and young adults with drug use disorders, 1990–2021: based on GBD 2021

**DOI:** 10.3389/fpubh.2025.1583812

**Published:** 2025-09-11

**Authors:** Ming Wang, Ting Yu

**Affiliations:** ^1^Department of Anesthesiology, Beilun People's Hospital, Ningbo, Zhejiang, China; ^2^Department of Pharmacy, Beilun People's Hospital, Ningbo, Zhejiang, China

**Keywords:** drug use disorders (DUDs), adolescents and young adults, trend, age-standardized incidence rate (ASIR), disability-adjusted life years (DALYs)

## Abstract

**Background:**

The objective of this study was to assess the distribution and trends in the prevalence of drug use disorders (DUDs) among adolescents and young adults (aged 15 to 39 years), disaggregated by gender, region, country, and socio-demographic index (SDI), over the period from 1990 to 2021.

**Methods:**

Utilizing data from GBD 2021, we estimated the incidence, disability-adjusted life years (DALYs), age-standardized incidence rates (ASIR), age-standardized DALY rates, and age-standardized death rates (ASDR) for DUDs among adolescents and young adults across 204 countries and 21 regions from 1990 to 2021. Additionally, we assessed the trend of the estimated annual percentage change (EAPC).

**Results:**

Globally, the ASIR of new DUD cases among adolescents and young adults exhibited a decreasing trend from 1990 to 2021, with an EAPC of −0.46 (95% confidence interval: −0.52, −0.39). In 2021, the highest ASIR, age-standardized DALY rate, and ASDR were observed in high SDI areas, while the lowest rates were found in low SDI areas. The above two SDI regions demonstrated an increasing trend from 1990 to 2021. The ASIR, age-standardized DALY rates and ASDR for DUDs were consistently higher among males than females across low SDI, middle and low SDI, medium SDI, and middle and high SDI areas. The three countries with the highest ASIR were the United States (1096.05), Estonia (854.62), and New Zealand (815.92). The countries with the highest age-standardized DALY rate were the United States (3520.13), Canada (1665.49), and Estonia (1602.04). The countries with the highest ASDR were the United States (26.86), Canada (13.92), and Iceland (8.85).

**Conclusion:**

The disease burden of DUDs, measured by age-standardized DALY rates, varies across different SDI regions, with higher DALY rates observed in regions with high SDI. Additionally, gender plays a significant role in the distribution of this burden, with males experiencing a higher burden than females. Furthermore, there are distinct national and regional variations in the prevalence and trends of DUDs among adolescents and young adults.

## 1 Introduction

Drug use disorders (DUDs) are characterized by the compulsive and persistent use of specific drugs with addictive properties, primarily to achieve certain psychological effects rather than for medicinal purposes. This often leads to severe psychological and physiological repercussions, as well as societal issues such as cognitive deficits, suicidal inclinations, diminished quality of life, and heightened risk of communicable diseases (1, 2). DUDs represent a significant public health challenge (3). Since 1990, there has been a marked rise in the global prevalence of substance use disorders, attributed to both population growth and aging. Research indicates that between 1990 and 2016, the global new cases of substance use disorders related to opioids, cocaine, marijuana, and amphetamines surged by 47.3%, 39.7%, 25.6%, and 22.5% respectively (4). The Global Burden of Disease 2021 (GBD 2021) study on Injuries and Risk Factors reveals that DUDs remain a substantial source of global disease burden, ranking within the top 25 for years lived with disability (YLDs) from 2010 to 2021 (5). The World Drug Report 2023 estimates that by 2021, 39.5 million individuals will be affected by DUDs worldwide, marking a 45% increase over the previous decade. However, only one in five of these individuals receives treatment, and disparities in treatment accessibility continue to expand regionally.

Adolescents and young adults undergo a period of rapid mental and physical development (6). This stage is marked by prevalent issues related to alcohol, marijuana, and other substance use (7). Recent evidence indicates a significant rise in DUDs among this demographic. Data from the U. S. government reveals that 12 percent of individuals aged 12–17 have experienced at least one DUD in their lifetime, marking a 4 percent increase since 2004. Furthermore, DUDs are linked to decreased academic performance, future delinquent behavior, and various psychiatric disorders such as depression, anxiety, and suicidal ideation (8). Consequently, this age group represents a crucial period for public interventions concerning substance use, with significant implications for their future development, mental health, and social identity transformations (9).

This study primarily utilized data from GBD2021 to conduct a comprehensive analysis of the magnitude and temporal trends of the burden of DUDs among adolescents and young adults (aged 15 to 39 years) from 1990 to 2021. The analysis was conducted separately by gender and region, providing a foundation for the development of effective policies and strategies aimed at reducing the burden of DUDs in specific populations. Furthermore, this study primarily utilized data from GBD2021 to conduct a comprehensive analysis of the magnitude and temporal trends of the burden of DUDs among adolescents and young adults (aged 15 to 39 years) from 1990 to 2021. The analysis was conducted separately by gender and region, providing a foundation for the development of effective policies and strategies aimed at reducing the burden of DUDs in specific populations.

Although GBD studies have previously examined substance use burdens in adolescents and young adults—for instance, a comprehensive analysis of alcohol and drug use in individuals aged 10–24 years from 1990 to 2019 (10), broader surveys of SUD burden in this age range using GBD 2019 data (11), and more recent projections using GBD 2021 (12)—these investigations have generally focused on narrower developmental windows, combined alcohol and drug use, or examined single drug categories. In contrast, our study targets the full span of adolescence to young adulthood (15–39 years), focuses specifically on DUDs, and provides detailed stratification by SDI, gender, region, and country, alongside long-term EAPC trend analysis from 1990 to 2021. While studies on opioid use disorder (OUD) among 15–39 year olds exist (13), no prior work has offered such a comprehensive and up-to-date assessment of DUDs in this age group. Our analysis therefore extends prior GBD work and fills an important gap, offering novel, age-stratified, and subgroup-specific insights to inform targeted intervention strategies.

## 2 Methods

### 2.1 Data sources

Upon examination and evaluation of 369 diseases, injuries, and impairments, as well as 87 risk factors derived from the GDB 2021 database via the Global Health Data Exchange query tool (https://ghdx.healthdata.org/gbd-results-tool), we extracted data pertaining to DUDs, new cases, death rates, and disability-adjusted life years (DALYs) for 204 countries and 21 regions spanning the period from 1990 to 2021. Consistent with GBD 2019, the methodologies employed for data collection, processing, and comprehensive analysis in GBD 2021 have been detailed previously (5, 14, 15).

In the GBD 2021 study, DUDs are classified based on the criteria set forth in the Diagnostic and Statistical Manual of Mental Disorders (DSM-IV-TR) or the International Classification of Diseases (ICD-10). This includes opioid use disorder, cocaine use disorder, marijuana use disorder, amphetamine use disorder, and other DUDs such as hallucinogen dependence, inhalant or solvent dependence, sedative dependence, and other forms of drug and substance dependence (5, 14).

For the purpose of this study, “adolescents and young adults” were defined as individuals aged 15–39 years. This age range was chosen to capture the broader period of vulnerability and high risk for DUDs, while maintaining sufficient statistical power for regional and global comparisons. Subgroup analyses for finer age brackets (e.g., 15–19, 20–24, 25–29, 30–34, 35–39) were not conducted due to data sparsity in certain regions and to ensure comparability across 204 countries and 21 regions.

The Socio-demographic Index (SDI) referenced in this study serves as a holistic measure of a country's or region's developmental status. It is derived from a combination of factors including the total fertility rate among females under 25, the average educational attainment of females aged 15 and above, and per capita income, among other variables (16). For the purposes of this study, the world is divided into five categories based on SDI: low, medium-low, medium, medium-high, and high. For the use of identified data in GBD study, a waiver of informed consent has been approved by the University of Washington Institutional Review Board. This study did not involve individual participants. The ethics approval can be found at https://www.healthdata.org/.

### 2.2 Quality control and data validation

The GBD 2021 study framework implements standardized quality control and validation procedures to ensure the reliability and comparability of estimates across countries and over time. These procedures include systematic data cleaning, outlier detection, and internal consistency checks among related epidemiological measures. When available, cross-validation with independent data sources is performed (17). To address potential inconsistencies arising from changes in disease classification systems, the GBD study methodology applies standardized mapping and cross-walking procedures to adjust for changes between ICD-9 and ICD-10 codes, ensuring consistency of DUD definitions across the study period (16).

### 2.3 Statistical analysis

This study was designed as a descriptive epidemiological analysis aimed at depicting the temporal and spatial patterns of the burden of DUDs by gender, SDI, and geographic region. Data are presented as absolute numbers with 95% uncertainty intervals (UIs). For the purpose of comparing populations across different regions or time periods, the population was age-standardized in this study. Each parameter was described using 95% UIs, and the standardized population size was expressed as age-standardized rates (ASR) per 100, 000 population. The estimated annual percentage change (EAPC) was used to describe the trend of ASR over a specific time interval. The Joinpoint Regression Program 4.7.0.0, developed by the American Institute for Cancer Research, was utilized in this study to calculate the estimated EAPC and 95% confidence intervals (CIs) for age-standardized incidence rate (ASIR), age-standardized death rate (ASDR), age-standardized DALY rate, and standardized rate for the years 1990–2021. The ASDR and DALY standardized rate were used to assess the burden of DUDs among adolescents and young adults in 21 regions and 204 countries from 1990 to 2021, quantifying the trend over the entire period with EPAC. The regression-based model used to calculate the EAPC for assessing time trends fitted a regression line to the natural logarithm of the standardized rate, i.e., y = α + βx + ε, where y = ln (ASR) and x = calendar year. The EAPC 95% CIs were also derived from the linear regression model and were calculated as 100 × (exp(β) – 1). If the upper bound of the 95% CI of the EAPC is < 0, it indicates a downward trend, whereas if the lower bound of the 95% CI of the EAPC is >0, it signifies an upward trend of burden; otherwise, it is considered a stable trend (18). Assumptions, model fit, and handling of missing data The regression-based EAPC model assumes a linear change in the natural logarithm of age-standardized rates over time. Model fit was assessed through inspection of residuals and evaluation of goodness-of-fit statistics to ensure appropriateness of the linear approximation. Missing or uncertain country-level estimates were handled using GBD 2021 standard procedures, including multiple imputation and statistical modeling, producing estimates with associated 95% uncertainty intervals (19). Specific analysis methods have been detailed in previous studies (2, 14). *P*-values < 0.05 were deemed statistically significant.

## 3 Results

### 3.1 Distribution of the global burden of DUDs and its trends among adolescents and young adults

**Figure 1:**
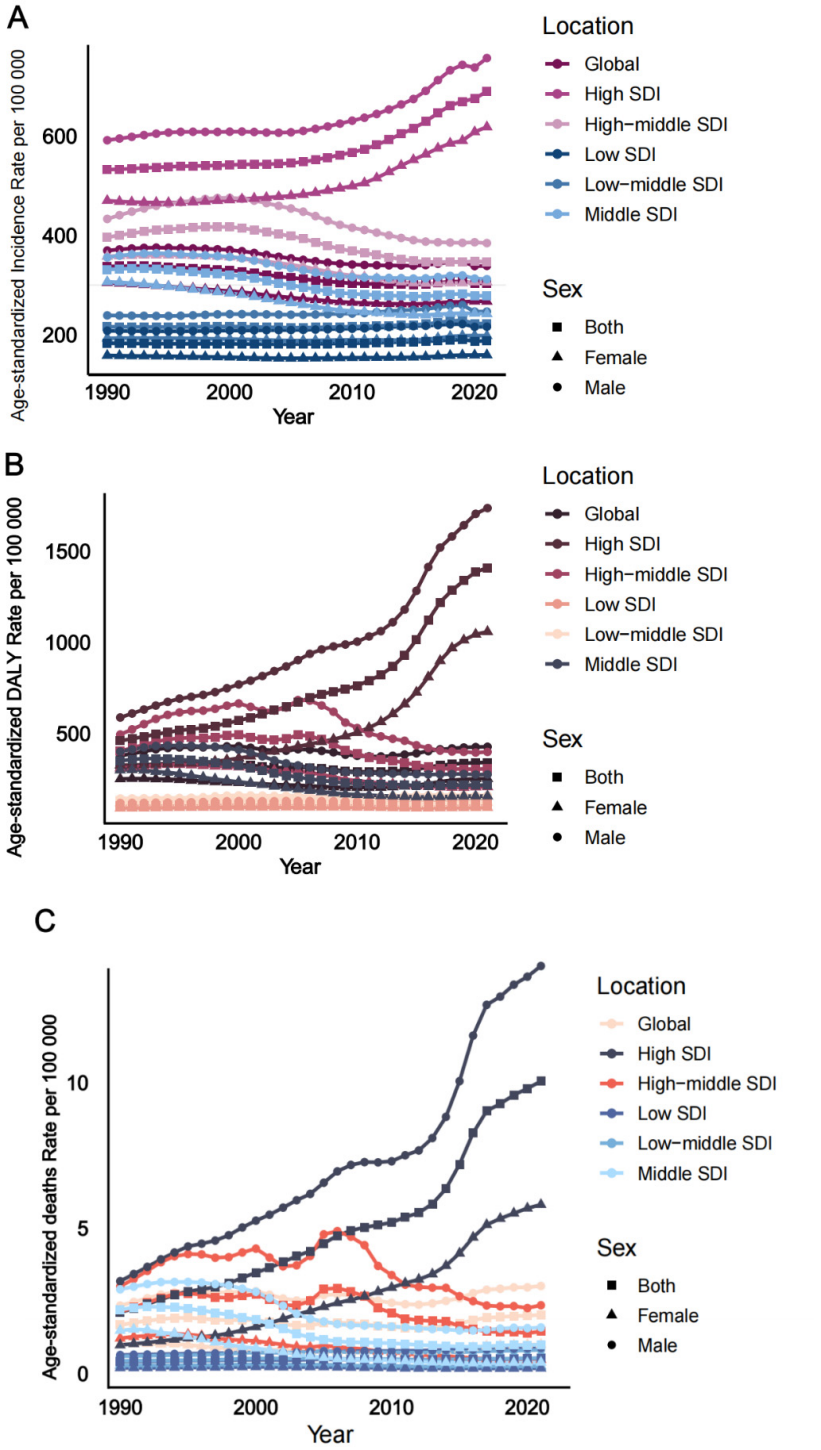
Trends in DUDs incidence, DALYs and deaths from 1990 to 2021. **(A)** Age-standardized incidence rate per 100,000. **(B)** Age-standardized DALY rate per 100,000. **(C)** Age-standardized deaths rate per 100,000.

Globally, ASIR for DUDs among adolescents and young adults showed a modest decline from 337. 53 in 1990 to 303. 55 in 2021 (EAPC = −0.46, 95% CI: −0.52, −0.39), suggesting a downward trend over the three decades. While this suggests a decreasing trend in ASIR, overlapping 95% UIs across years indicate that the changes should be interpreted with caution. The age-standardized DALYs were 314.26 in 1990 and 340.34 in 2021, and the ASDR was 1.67 in 1990 and 2.02 in 2021, with overlapping UIs, suggesting no statistically meaningful change from 1990 to 2021 (Table 1 and Figure 1).

**Table 1:** Global and regional trends in DUDs burden: age-standardized incidence, DALY and deaths (1990–2021).

**Location**	**1990**	**2021**	**EAPC (95%CI)**
	**ASR**	**ASR**	**ASR**
**Incidence**			
Global	337.53 (337.29, 337.78)	303.55 (303.35, 303.75)	−0.46 (−0.52, −0.39)
High-middle SDI	396.05 (395.47, 396.63)	346.97 (346.40, 347.54)	−0.69 (−0.82, −0.56)
High SDI	532.27 (531.49, 533.06)	689.81 (688.91, 690.71)	0.77 (0.62, 0.92)
Low-middle SDI	216.18 (215.75, 216.61)	222.51 (222.18, 222.84)	0.13 (0.08, 0.18)
Low SDI	182.93 (182.31, 183.56)	187.45 (187.04, 187.86)	0.12 (0.08, 0.15)
Middle SDI	331.36 (330.94, 331.77)	277.84 (277.49, 278.18)	−0.74 (−0.83, −0.66)
Central Europe, eastern Europe, and central Asia	404.60 (403.61, 405.59)	419.11 (418.00, 420.23)	−0.09 (−0.29, 0.11)
High-income	551.04 (550.26, 551.83)	712.00 (711.08, 712.92)	0.79 (0.67, 0.92)
Latin America and Caribbean	282.31 (281.49, 283.12)	281.18 (280.49, 281.86)	0.05 (0.02, 0.09)
Southeast Asia, east Asia, and Oceania	375.21 (374.78, 375.65)	300.10 (299.71, 300.50)	−1.04 (−1.17, −0.92)
Sub-Saharan Africa	175.27 (174.65, 175.89)	172.91 (172.52, 173.30)	−0.04 (−0.06, −0.03)
Andean Latin America	253.14 (250.61, 255.69)	257.54 (255.62, 259.46)	0.07 (0.06, 0.09)
Australasia	938.67 (931.98, 945.41)	816.61 (811.03, 822.22)	−0.48 (−0.56, −0.41)
Caribbean	320.21 (317.32, 323.11)	313.88 (311.29, 316.47)	−0.14 (−0.16, −0.12)
Central Asia	307.64 (305.60, 309.70)	312.99 (311.18, 314.81)	0.07 (0.06, 0.08)
Central Europe	316.84 (315.22, 318.47)	332.05 (330.07, 334.04)	0.20 (0.16, 0.24)
Central Latin America	239.82 (238.64, 241.00)	244.31 (243.34, 245.27)	0.14 (0.09, 0.18)
Central Sub-Saharan Africa	186.31 (184.41, 188.23)	189.37 (188.19, 190.56)	0.09 (0.05, 0.13)
East Asia	415.95 (415.42, 416.48)	321.85 (321.32, 322.38)	−1.26 (−1.43, −1.10)
Eastern Europe	488.82 (487.30, 490.33)	532.03 (530.17, 533.90)	−0.05 (−0.34, 0.23)
Eastern Sub-Saharan Africa	169.34 (168.36, 170.32)	172.23 (171.60, 172.86)	0.10 (0.06, 0.13)
High-income Asia Pacific	401.39 (399.88, 402.90)	393.02 (391.24, 394.81)	−0.08 (−0.10, −0.05)
High-income North America	690.51 (688.92, 692.10)	1,065.39 (1,063.53, 1,067.25)	1.44 (1.15, 1.73)
North Africa and Middle East	237.27 (236.43, 238.11)	252.02 (251.40, 252.64)	0.24 (0.18, 0.31)
Oceania	308.37 (301.72, 315.13)	310.40 (305.82, 315.02)	0.01 (0.00, 0.03)
South Asia	206.27 (205.84, 206.70)	223.20 (222.87, 223.53)	0.29 (0.20, 0.38)
Southeast Asia	257.40 (256.69, 258.11)	262.34 (261.73, 262.94)	0.03 (−0.01, 0.07)
Southern Latin America	332.67 (330.09, 335.27)	345.41 (343.13, 347.71)	0.13 (0.11, 0.15)
Southern Sub-Saharan Africa	282.02 (279.77, 284.29)	273.91 (272.15, 275.68)	−0.30 (−0.41, −0.19)
Tropical Latin America	329.08 (327.69, 330.48)	327.43 (326.22, 328.65)	0.08 (0.02, 0.15)
Western Europe	532.04 (530.83, 533.25)	552.08 (550.78, 553.39)	0.07 (0.01, 0.12)
Western Sub-Saharan Africa	145.27 (144.36, 146.19)	151.08 (150.51, 151.66)	0.17 (0.12, 0.22)
**DALYs (Disability-Adjusted Life Years)**			
Global	314.26 (314.03, 314.50)	340.34 (340.13, 340.55)	−0.14 (−0.35, 0.06)
High-middle SDI	403.48 (402.90, 404.06)	306.52 (306.00, 307.04)	−1.52 (−1.95, −1.09)
High SDI	459.22 (458.51, 459.93)	1,408.73 (1,407.51, 1,409.96)	3.67 (3.33, 4.01)
Low-middle SDI	130.99 (130.65, 131.33)	140.89 (140.63, 141.15)	0.13 (0.05, 0.22)
Low SDI	103.74 (103.26, 104.23)	114.42 (114.09, 114.74)	0.25 (0.19, 0.31)
Middle SDI	350.58 (350.16, 351.01)	216.62 (216.33, 216.92)	−2.14 (−2.39, −1.90)
Central Europe, Eastern Europe, and Central Asia	427.90 (426.88, 428.91)	535.21 (533.99, 536.43)	0.21 (−0.61, 1.04)
High-income	485.48 (484.76, 486.21)	1,474.26 (1,472.98, 1,475.54)	3.63 (3.29, 3.97)
Latin America and Caribbean	188.45 (187.78, 189.13)	210.92 (210.34, 211.51)	0.44 (0.36, 0.52)
Southeast Asia, east Asia, and Oceania	400.34 (399.89, 400.79)	199.92 (199.60, 200.24)	−3.23 (−3.63, −2.82)
Sub-Saharan Africa	112.65 (112.15, 113.16)	102.22 (101.92, 102.53)	−0.39 (−0.51, −0.27)
Andean Latin America	164.87 (162.83, 166.93)	178.33 (176.75, 179.92)	0.35 (0.19, 0.50)
Australasia	821.73 (815.51, 827.98)	852.10 (846.59, 857.65)	−0.47 (−0.84, −0.11)
Caribbean	181.19 (179.01, 183.39)	180.30 (178.36, 182.26)	−0.72 (−1.14, −0.31)
Central Asia	260.31 (258.43, 262.19)	306.08 (304.33, 307.85)	0.52 (0.03, 1.00)
Central Europe	200.99 (199.69, 202.29)	234.99 (233.35, 236.64)	0.46 (0.41, 0.50)
Central Latin America	171.50 (170.51, 172.50)	174.78 (173.97, 175.60)	0.05 (−0.03, 0.12)
Central Sub-Saharan Africa	94.54 (93.17, 95.92)	106.28 (105.38, 107.18)	0.45 (0.38, 0.52)
East Asia	490.88 (490.30, 491.46)	231.60 (231.16, 232.04)	−3.62 (−4.10, −3.15)
Eastern Europe	607.21 (605.55, 608.87)	825.89 (823.67, 828.11)	0.30 (−0.66, 1.27)
Eastern Sub-Saharan Africa	102.53 (101.73, 103.33)	113.56 (113.03, 114.09)	0.29 (0.28, 0.30)
High-income Asia Pacific	182.70 (181.68, 183.73)	182.76 (181.57, 183.95)	−0.04 (−0.17, 0.09)
High-income North America	708.16 (706.62, 709.70)	3,337.07 (3,333.88, 3,340.27)	5.61 (5.20, 6.02)
North Africa and Middle East	279.92 (279.00, 280.84)	304.49 (303.82, 305.17)	0.34 (0.09, 0.59)
Oceania	143.19 (138.67, 147.83)	140.01 (136.95, 143.12)	−0.10 (−0.13, −0.08)
South Asia	115.68 (115.35, 116.00)	128.76 (128.51, 129.01)	0.08 (−0.13, 0.29)
Southeast Asia	141.50 (140.97, 142.03)	147.09 (146.64, 147.54)	0.06 (0.00, 0.12)
Southern Latin America	209.51 (207.46, 211.58)	219.99 (218.18, 221.80)	0.21 (0.09, 0.32)
Southern Sub-Saharan Africa	306.27 (303.87, 308.68)	224.78 (223.19, 226.37)	−1.29 (−1.76, −0.81)
Tropical Latin America	213.56 (212.43, 214.69)	269.59 (268.50, 270.68)	1.03 (0.88, 1.17)
Western Europe	463.43 (462.33, 464.53)	527.05 (525.80, 528.30)	−0.08 (−0.32, 0.16)
Western Sub-Saharan Africa	69.16 (68.53, 69.79)	67.91 (67.53, 68.29)	0.01 (−0.02, 0.05)
**Deaths**			
Global	1.67 (1.66, 1.69)	2.02 (2.00, 2.04)	0.03 (−0.27, 0.33)
High-middle SDI	2.12 (2.08, 2.16)	1.45 (1.42, 1.49)	−2.06 (−2.67, −1.44)
High SDI	2.10 (2.05, 2.15)	10.05 (9.95, 10.15)	5.06 (4.81, 5.31)
Low-middle SDI	0.44 (0.42, 0.46)	0.54 (0.52, 0.55)	0.39 (0.21, 0.57)
Low SDI	0.41 (0.38, 0.45)	0.53 (0.50, 0.55)	0.59 (0.49, 0.69)
Middle SDI	2.20 (2.17, 2.24)	0.99 (0.97, 1.01)	−3.55 (−4.03, −3.07)
Central Europe, eastern Europe, and central Asia	2.14 (2.07, 2.21)	3.46 (3.37, 3.56)	0.84 (−0.38, 2.07)
High-income	2.28 (2.23, 2.33)	10.63 (10.53, 10.74)	4.98 (4.70, 5.26)
Latin America and Caribbean	0.35 (0.32, 0.38)	0.75 (0.71, 0.78)	2.27 (1.93, 2.62)
Southeast Asia, east Asia, and Oceania	2.53 (2.49, 2.57)	0.75 (0.73, 0.77)	−5.57 (−6.35, −4.78)
Sub-Saharan Africa	0.46 (0.43, 0.50)	0.46 (0.44, 0.49)	−0.03 (−0.28, 0.23)
Andean Latin America	0.46 (0.36, 0.59)	0.63 (0.54, 0.73)	1.08 (0.70, 1.46)
Australasia	4.43 (3.99, 4.91)	5.86 (5.42, 6.33)	−0.36 (−1.13, 0.41)
Caribbean	0.28 (0.20, 0.38)	0.51 (0.41, 0.63)	−1.09 (−2.84, 0.68)
Central Asia	0.59 (0.50, 0.69)	1.20 (1.10, 1.32)	2.47 (1.00, 3.95)
Central Europe	0.91 (0.83, 1.01)	1.09 (0.98, 1.20)	0.14 (−0.10, 0.39)
Central Latin America	0.54 (0.49, 0.60)	0.65 (0.61, 0.71)	0.09 (−0.24, 0.42)
Central Sub-Saharan Africa	0.39 (0.31, 0.49)	0.49 (0.43, 0.56)	0.80 (0.54, 1.05)
East Asia	3.30 (3.25, 3.35)	0.95 (0.92, 0.98)	−5.84 (−6.67, −5.01)
Eastern Europe	3.29 (3.18, 3.42)	5.91 (5.73, 6.09)	1.01 (−0.30, 2.33)
Eastern Sub–Saharan Africa	0.61 (0.55, 0.68)	0.81 (0.76, 0.86)	0.81 (0.77, 0.86)
High-income Asia Pacific	0.15 (0.12, 0.18)	0.23 (0.19, 0.27)	0.31 (−0.64, 1.28)
High-income North America	3.11 (3.01, 3.21)	25.57 (25.29, 25.85)	7.56 (7.26, 7.85)
North Africa and Middle East	1.50 (1.43, 1.57)	1.58 (1.53, 1.63)	0.20 (−0.13, 0.53)
Oceania	0.29 (0.12, 0.60)	0.20 (0.10, 0.36)	−1.56 (−1.89, −1.23)
South Asia	0.46 (0.44, 0.48)	0.49 (0.47, 0.50)	−0.26 (−0.58, 0.06)
Southeast Asia	0.31 (0.29, 0.34)	0.40 (0.38, 0.43)	0.64 (0.46, 0.83)
Southern Latin America	0.07 (0.04, 0.12)	0.21 (0.16, 0.28)	4.28 (3.71, 4.85)
Southern Sub-Saharan Africa	1.46 (1.30, 1.64)	1.07 (0.96, 1.18)	−1.16 (−2.07, −0.25)
Tropical Latin America	0.14 (0.11, 0.17)	0.94 (0.88, 1.01)	7.74 (6.82, 8.66)
Western Europe	2.69 (2.61, 2.78)	3.26 (3.16, 3.35)	−0.24 (−0.54, 0.06)
Western Sub-Saharan Africa	0.04 (0.03, 0.06)	0.03 (0.02, 0.04)	0.33 (−0.98, 1.66)

### 3.2 Burden and trends in the regional and gender distribution of SDI for DUDs for adolescents and young adults

The highest ASIR was observed in the high SDI region in 2021, exhibiting a significant upward trend from 1990 to 2021, with an EAPC of 0.77 (95% confidence interval: 0.62, 0.92). Conversely, the lowest ASIR was found in the low SDI region, which also demonstrated an increasing trend from 1990 to 2021, albeit at a slower rate, with an EAPC of 0.12 (95% CI: 0.08, 0.15). The age-standardized DALY rate was highest in the high SDI region, showing a significant increasing trend from 1990 to 2021, with an EAPC of 3.67 (95% CI: 3.33, 4.01). The lowest DALY rate was observed in the low SDI region, which exhibited a slight upward trend from 1990 to 2021, with an EAPC of 0. 25 (95% CI: 0.19, 0.31). The ASDR was highest in the high SDI region, demonstrating a significant upward trend from 1990 to 2021, with an EAPC of 5.06 (95% CI: 4.81, 5.31). It was lowest in the low SDI region, which also showed an increasing trend from 1990 to 2021, with an EAPC of 0. 59 (95% CI: 0.49, 0.69). The ASIR, DALY rate, and death rate, along with their rates of increase in the high SDI region, were higher than those in several other regions, as depicted in Table 1 and Figure 1.

### 3.3 National burden of disease distribution of DUDs for adolescents and young adults

**Figure 2:**
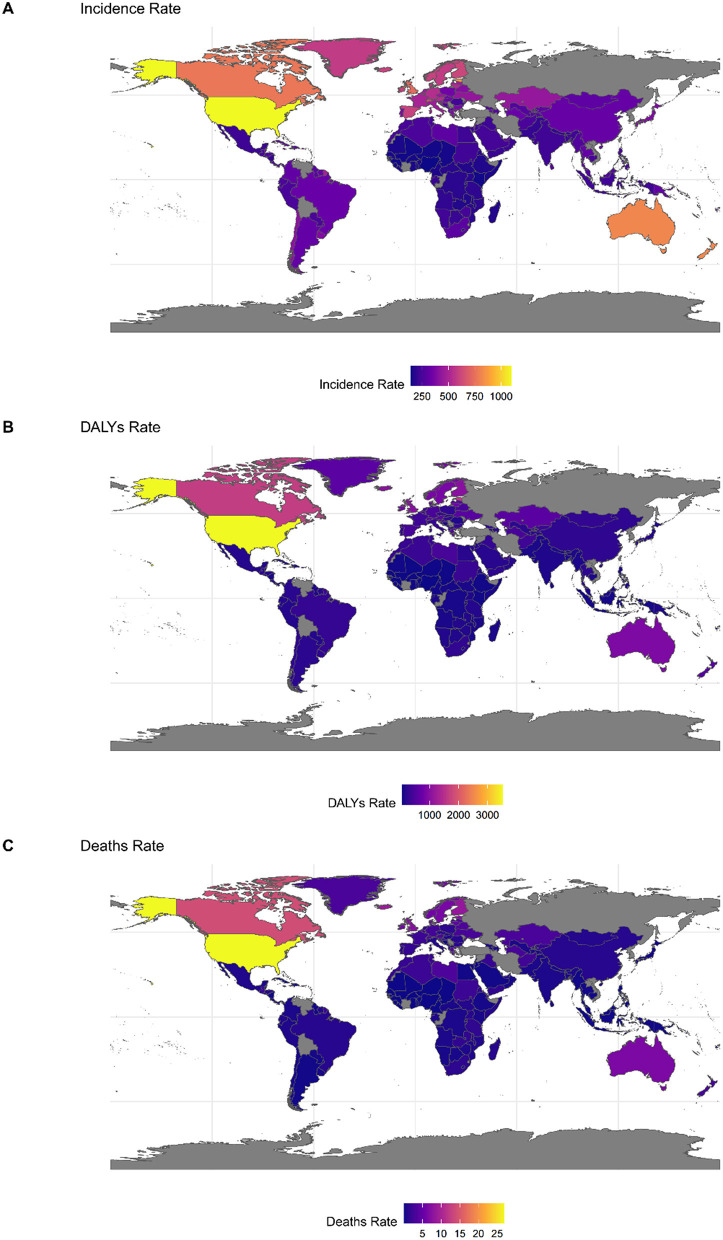
The global disease burden of DUDs for both sexes in 204 countries and territories in 2021. **(A)** Incidence rate. **(B)** DALYs rate. **(C)** Deaths.

In 2021, among the 204 countries examined, the United States (1, 096.05), Estonia (854.62), and New Zealand (815.92) recorded the highest ASIR for DUDs among adolescents and young adults. Conversely, Nigeria (144.58), Togo (146.34), and Kenya (149.14) reported the lowest rates, with Nigeria and Togo located in Western Sub-Saharan Africa, and Kenya in Eastern Sub-Saharan Africa (Supplementary Table 1 and Figure 2).

In 2021, among the 204 countries assessed, the United States (3520. 13), Canada (1665.49), and Estonia (1602.04) recorded the highest age-standardized DALY rates for adolescents and young adults. These nations are located in high-income North America and Eastern Europe respectively. Conversely, Nigeria (60.75), Guinea (65.58), and Guinea-Bissau (68.27) reported the lowest rates, all situated in the Western sub-Saharan Africa region (Supplementary Table 2 and Figure 2).

In 2021, among the 204 countries assessed, the United States (26.86) and Canada (13.92) in high-income North America, along with Iceland (8.85) in Western Europe, exhibited the highest ASDRs for DUDs among adolescents and young adults. Conversely, Palau (0.01) in Oceania, as well as Burkina Faso (0.02) and Niger (0.02) in Western sub-Saharan Africa, reported the lowest rates (Figure 2 and Supplementary Table 3).

### 3.4 Trends in national burden of disease for DUDs in adolescents and young adults

**Figure 3:**
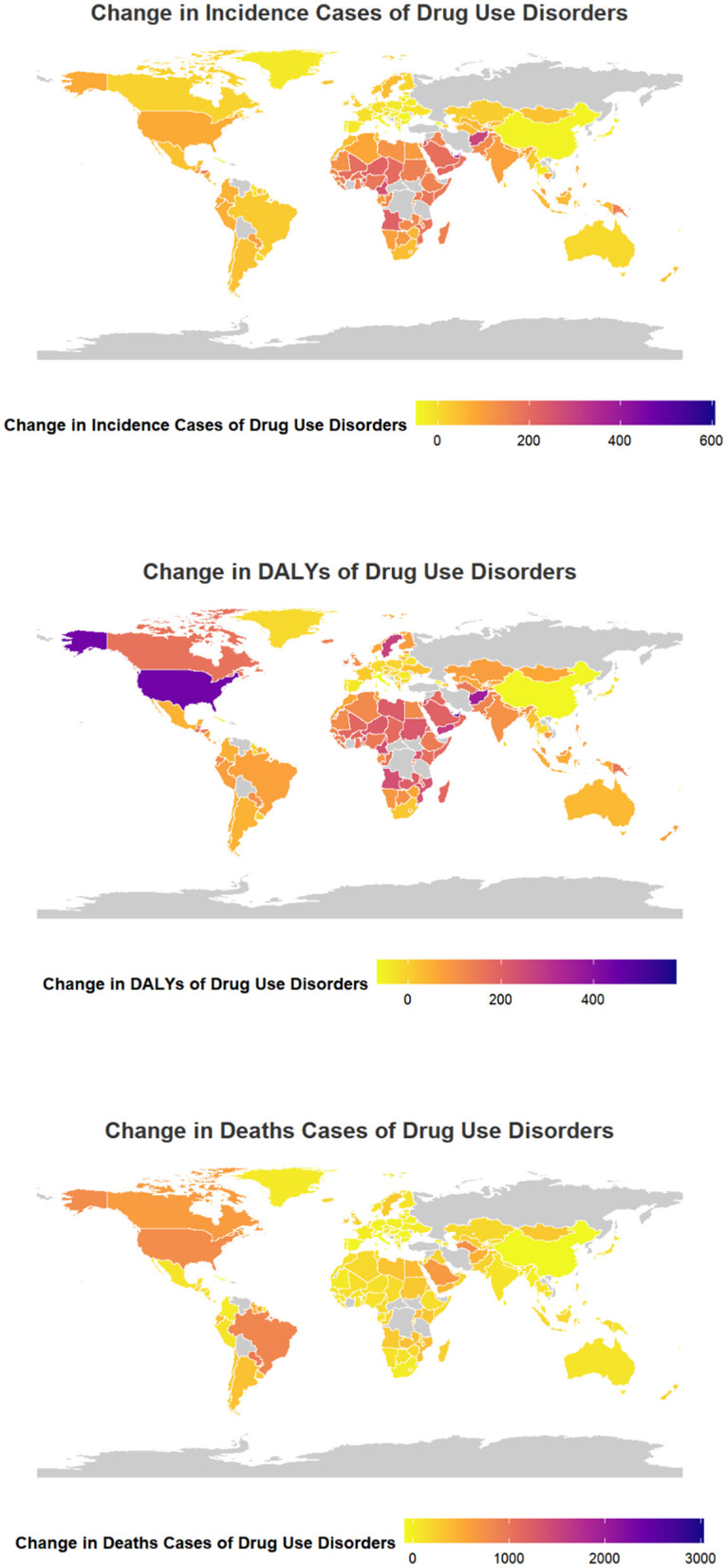
Change cases of DUDs for both sexes in 204 countries and territories. **(A)** Incidence cases. **(B)** DALYs. **(C)** Deaths cases.

Among the 204 countries analyzed Qatar (8.49), the United Arab Emirates (EAPC = 7.05), and Equatorial Guinea (EAPC = 5.89) exhibited the most significant increases in the new cases of DUDs among adolescents and young adults from 1990 to 2021. Conversely Georgia (EAPC = −1. 94), Latvia (EAPC = −1.89), and China (EAPC = −1.85) recorded the lowest changes during the same period (Supplementary Table 1 and Figure 3).

Among the 204 countries assessed, Qatar (EAPC = 8.63), United Arab Emirates (EAPC = 8.07), and Equatorial Guinea (EAPC = 6.18) recorded the highest increases in DALYs for DUDs among adolescents and young adults from 1990 to 2021. Conversely, Italy (EAPC = −4.31), China (EAPC = −4.16) and Switzerland (EAPC = −3.42) and experienced the most significant decreases (Supplementary Table 2 and Figure 3).

The three countries that experienced the most significant increase in DUD-related death cases among adolescents and young adults from 1990 to 2021, out of the 204 nations studied, were Maldives (EAPC = 9.31), Mauritius (EAPC = 9.02), and Paraguay (EAPC = 9. 02), respectively. Conversely, the three countries with the least change were Italy (EAPC = −6.93), Guam (EAPC = −6.59) and Northern Mariana Islands (EAPC = −6.50), respectively (Supplementary Table 2 and Figure 3).

## 4 Discussion

Using GBD 2021 data, we analyzed the burden of DUDs by gender, region, and country from 1990 to 2021. A decreasing trend in ASIR from 1990 to 2021 for DUDs among this demographic was observed. The DALY-based burden of DUDs varied across SDI regions, with high SDI regions experiencing higher age-standardized DALY rates. Gender also played a role in the burden of DUDs, with males showing higher burdens than females. Furthermore, significant national and regional differences were evident in both the burden of DUDs and the trend of this burden among adolescents and young adults. This suggests that targeted policies should be implemented in different regions and populations to reduce the burden of DUDs on adolescents and young adults. Our analysis extends beyond existing GBD reports by concentrating on adolescents and young adults, offering a finer stratification of burden and trends by socio-demographic and geographic factors, and identifying high-risk subgroups that have not been the focus of earlier GBD publications.

Despite a slight decline in the ASIR attributable to DUDs from 1990 to 2021, the age-standardized DALY rates and ASDR among adolescents and young adults remained relatively stable in 2021, indicating no meaningful overall change. The gap in intervention for mental and substance use disorders in developing countries is reported to be as high as 90%. Even in developed nations, treatment often commences years after the disorder's onset, with low treatment rates and delays contributing significantly to the high burden of DUDs. This situation can be attributed to three primary factors: scarcity of human and financial resources, inequitable distribution, and inefficient use (20, 21). Furthermore, the stigma associated with substance use is a significant factor that cannot be overlooked (4).

According to previous literature, women typically initiate substance use later than men and are generally less likely to develop DUDs (22). In the United States, studies have shown that men are 2.33 times more likely to have DUDs and 2.25 times more likely to be drug dependent than women (23). Similarly, in Canada, age-standardized years of life lost (YLD) are significantly higher for men than for women. The prevalence of opioid use disorders and the DALY rate are 1.6 times higher for men, while the death rate is 2.3 times higher. The YLD rate for men is 1.3 times higher than the YLL rate for women, and the YLL rate for men is nearly 2.5 times higher than the YLL rate for women (6). The 2019 GBD study found that globally, with the exception of Paraguay, men have higher rates of DALY for amphetamine, marijuana, cocaine, and opioid use disorders than women (24). This aligns with findings that among adolescents and young adults, men bear a greater burden of DUDs than women. Men are more likely to consume psychotropic medications, while women often face barriers to access due to childbirth, breastfeeding, and other factors (25). Potential reasons for these disparities include men's higher likelihood of consuming psychotropic drugs and women's limited access due to reproductive health factors. However, recent years have seen an increase in DUDs among women globally, including in countries like Canada, Australia, and the United States. This may be linked to women's vulnerability to violence, sleep disorders, and other conditions (25). Further research is needed to understand these differences and to develop targeted interventions to address the disease burden and health disparities between men and women.

DUDs exhibit substantial variation across different countries and geographic regions, as indicated by previous research. These studies have demonstrated that opioid-induced deaths among individuals with DUDs are most prevalent in high-income regions, including Canada and the United States, as well as in central and eastern Europe (6). The SDI serves as a reflection of national and regional healthcare systems (26). According to the findings of the GBD2016 study, the burden due to substance use escalates in correlation with increasing SDI levels (4). This underscores the need for targeted policies tailored to specific regions and populations to mitigate the burden of DUDs among adolescents and young adults. The observed increase in DALY rates in high SDI regions may be driven by factors such as greater availability of psychoactive substances, delayed initiation of treatment, elevated incarceration rates, and broader socio-economic determinants (27, 28). In contrast, the relatively lower burden in low SDI regions may partially reflect underdiagnosis, limited access to healthcare services, and social stigma associated with substance use. Moreover, gender disparities highlight the importance of considering structural and psychosocial influences: males generally exhibit higher rates of DUDs, whereas the emerging increase among females may be linked to heightened vulnerability to violence, sleep disturbances, and restricted access to healthcare (29, 30). Research conducted based on GBD2019 also identified a positive correlation between the burden of DUDs and the level of socio-demographic development at both regional and national levels, with the highest burden of DUDs being observed in regions with high SDI scores (26). This aligns with the findings of the current study, which reveals that regions with high SDI bear the greatest burden of DUDs and exhibit a highly significant increasing trend, while regions with low SDI carry the least burden. At the national level, the 2019 study found that high-income North America and Australasia had the greatest burdens (26). Other studies have identified that the age-standardized burden of drug use attribution in 2019 was highest in high-income North America (4). Developed nations, such as the United States and Canada, bear the greatest burden of DUDs, while certain impoverished countries like Somalia, Rwanda, and Nepal exhibit the least. This disparity can be attributed to multiple factors, including the structure of healthcare systems where interventions or treatments are administered, and the societal stigma associated with substance use. In high-income countries, healthcare is typically universal and free for all citizens, and psychoactive substances like methadone are often provided without charge (26, 31). U. S. studies suggest that incarceration may play a significant role as an upstream determinant of DUD-related deaths, in addition to local economic conditions and opioid prescription rates. Consequently, the rising incarceration rates in the U. S. over the past decades could have significantly contributed to the increase in DUD-related deaths (27).

Several treatments and interventions have demonstrated effectiveness and potential for public health impact (20). For instance, psychological and social interventions can address certain psychostimulant use disorders. Opioid therapy, in particular, can mitigate the risk of opioid use and injection, enhance physical and mental health, and decrease death rates, thereby progressively alleviating societal burden (32, 33). However, the prevalence of these interventions varies significantly across nations and is intimately tied to social development. Consequently, robust measures are required, with a focus on priority regions and populations, to effectively diminish the disease burden from DUDs.

This study utilized data from GBD 2021 to conduct a comprehensive descriptive analysis of the magnitude and temporal trends of the burden of DUDs among adolescents and young adults from 1990 to 2021. No multivariable or regression modeling was performed to assess independent associations between SDI, gender, and burden. Therefore, while our findings reveal important descriptive patterns, they should not be interpreted as evidence of causal or independent relationships. The analysis was conducted by gender, region, and country respectively, yielding more comprehensive results that serve as a foundation for the development of public health measures. However, this study has several limitations. Firstly, in GBD 2021, DUDs are defined according to the DSM-IV-TR or ICD-10 diagnostic criteria. The estimation of DUDs may vary if the Diagnostic and Statistical Manual of Mental Disorders (DSM-5) is employed. Secondly, estimates of the severity of DUDs may fluctuate across countries with diverse cultures and economies, potentially leading to an underestimation or overestimation of the global burden of disease for DUDs. Thirdly, the concept of disability in GBD is designed to describe only the health loss of an individual, excluding the impact on the family, social or other impacts, and socioeconomic consequences. Therefore, our estimates of the burden of disease may only represent partial estimates (4).

Based on our findings, policy and intervention strategies should be tailored according to SDI levels. In high-SDI regions, evidence-based interventions could include school- and community-based substance use prevention programs, early screening, and integrated mental health and substance use services, which have been shown to be effective in reducing the burden of DUDs (34, 35). In low-SDI regions, where incidence is increasing, strengthening healthcare infrastructure, expanding access to trained personnel, and culturally adapting prevention and treatment programs are crucial (24, 36). Cost-effective approaches, including leveraging digital health platforms and community health workers, could enhance feasibility. Alignment with international frameworks such as the WHO Global Action Plan on Substance Use Disorders can further guide implementation and resource allocation.

## 5 Conclusions

The significant health impact of DUDs among adolescents and young adults cannot be understated. There are distinct national and regional variations in both the burden of DUDs and their trends, with regions of high SDI experiencing greater burdens. Gender disparities are also evident, with males exhibiting a higher prevalence than females. Countries such as the United States, Estonia, New Zealand, Canada, and Iceland warrant global attention due to their pronounced DUD burdens. The findings indicate that future intervention strategies should prioritize males, adolescents, and high-risk regions to reduce avoidable deaths and disabilities, thereby enhancing the overall status related to DUDs. These findings provide novel, age-specific evidence that complements existing GBD studies, enabling more precise targeting of public health interventions for adolescents and young adults.

## Data Availability

Publicly available datasets were analyzed in this study. This data can be found here: Global Health Data Exchange database (GHDx) (http://ghdx.healthdata.org/gbd-results-tool).
