# Supplementary material for: Global burden of disease among adolescents and young adults with drug use disorders, 1990–2021: based on GBD 2021
Source: Front Public Health. 2025 Sep 11;13:1583812. doi: 10.3389/fpubh.2025.1583812 (PMC12460290; doi:10.3389/fpubh.2025.1583812)
Supplement: Supplementary file 1 [file Data_Sheet_1.doc]

**Supplementary table 1.** Incidence cases and ASIR of DUDs in 1990 and 2021 and its trends.

| location_name |  | 1990 |  | 2021 |  | EAPC_95%UI |  |
| --- | --- | --- | --- | --- | --- | --- | --- |
|  | sex | number_95%UI | ASR | number_95%UI | ASR | number_95%UI | ASR |
| Afghanistan | both | 7968.05(6567.53,9451.80) | 251.56(245.30,257.97) | 30822.54(25804.46,36329.29) | 253.46(250.50,256.45) | 4.32(4.01,4.63) | 0.23(0.12,0.33) |
| Albania | both | 4002.01(3208.87,4903.53) | 280.73(272.06,289.63) | 3035.00(2519.54,3688.66) | 321.52(310.13,333.25) | -0.84(-0.92,-0.76) | 0.52(0.48,0.55) |
| Algeria | both | 23485.87(19356.36,28859.96) | 236.49(233.40,239.62) | 43224.71(35594.68,51972.28) | 252.45(250.05,254.87) | 2.05(1.79,2.31) | 0.25(0.23,0.26) |
| American Samoa | both | 67.49(54.13,83.59) | 330.05(255.34,421.49) | 61.18(49.15,75.16) | 340.28(259.45,439.59) | -0.56(-0.79,-0.33) | 0.11(0.10,0.13) |
| Andorra | both | 121.61(97.08,149.52) | 492.67(407.34,592.21) | 130.45(107.85,158.08) | 513.33(427.14,613.11) | -0.12(-0.43,0.19) | 0.18(0.17,0.20) |
| Angola | both | 7352.28(6014.39,9027.38) | 190.72(186.33,195.20) | 23527.19(19160.29,28884.98) | 197.12(194.57,199.71) | 3.91(3.86,3.95) | 0.18(0.14,0.21) |
| Antigua and Barbuda | both | 88.77(71.71,110.60) | 345.24(277.00,425.93) | 116.26(94.14,142.86) | 344.86(284.76,414.37) | 0.96(0.74,1.18) | 0.09(0.07,0.12) |
| Argentina | both | 38771.35(32375.67,47592.34) | 315.60(312.46,318.76) | 54946.82(45317.69,67006.90) | 315.76(313.12,318.41) | 1.23(1.17,1.30) | 0.04(0.00,0.07) |
| Armenia | both | 4381.74(3644.39,5323.22) | 305.68(296.65,314.92) | 3084.65(2528.33,3748.48) | 292.51(281.97,303.38) | -0.96(-1.05,-0.87) | -0.12(-0.16,-0.09) |
| Australia | both | 65504.13(55580.88,76792.33) | 983.86(976.32,991.43) | 69824.02(58546.67,81145.15) | 815.31(809.20,821.46) | 0.23(0.16,0.30) | -0.58(-0.67,-0.49) |
| Austria | both | 13694.17(11302.97,16633.63) | 462.44(454.65,470.33) | 14670.74(12261.93,17207.40) | 529.90(521.21,538.71) | 0.01(-0.11,0.14) | 0.27(0.11,0.43) |
| Azerbaijan | both | 9268.44(7683.19,11284.26) | 290.21(284.28,296.24) | 11719.68(9574.03,14338.79) | 280.24(275.08,285.48) | 0.98(0.87,1.08) | -0.06(-0.13,0.00) |
| Bahamas | both | 445.56(360.00,540.07) | 377.10(342.71,414.24) | 592.83(481.24,719.50) | 382.34(352.17,414.42) | 0.93(0.85,1.02) | 0.11(0.05,0.17) |
| Bahrain | both | 618.22(496.65,774.05) | 238.75(219.78,259.12) | 1674.53(1355.71,2099.25) | 235.20(223.81,247.08) | 4.34(3.80,4.89) | 0.04(0.00,0.09) |
| Bangladesh | both | 86750.08(70422.87,106235.54) | 211.18(209.74,212.61) | 147104.89(118298.51,179664.13) | 214.84(213.75,215.95) | 1.72(1.62,1.81) | 0.11(0.09,0.14) |
| Barbados | both | 407.32(327.62,501.05) | 379.23(343.26,417.98) | 358.55(295.66,436.28) | 372.45(334.74,413.33) | -0.32(-0.40,-0.23) | -0.05(-0.09,0.00) |
| Belarus | both | 15616.27(13057.64,18903.93) | 399.33(393.04,405.70) | 11511.13(9497.97,13956.95) | 396.08(388.56,403.72) | -0.88(-1.15,-0.61) | 0.05(-0.10,0.19) |
| Belgium | both | 17468.53(14173.13,21525.07) | 480.27(473.11,487.51) | 16944.15(13870.05,20147.84) | 489.78(482.36,497.29) | -0.07(-0.16,0.02) | 0.03(-0.01,0.08) |
| Belize | both | 271.84(221.33,331.94) | 347.60(306.40,393.54) | 642.41(528.43,789.42) | 333.26(307.88,360.26) | 2.94(2.87,3.02) | -0.10(-0.14,-0.07) |
| Benin | both | 2620.38(2127.43,3289.09) | 156.75(150.72,162.98) | 8352.08(6868.41,10167.31) | 162.63(159.09,166.24) | 3.85(3.82,3.87) | 0.15(0.12,0.19) |
| Bermuda | both | 88.66(71.41,109.75) | 357.50(285.36,443.93) | 63.63(51.43,79.23) | 367.09(280.66,473.18) | -1.07(-1.17,-0.98) | 0.09(0.06,0.12) |
| Bhutan | both | 531.50(427.59,675.09) | 198.51(181.42,216.96) | 724.67(585.99,898.22) | 208.55(193.61,224.37) | 1.46(1.27,1.65) | 0.16(0.14,0.18) |
| Bolivia (Plurinational State of) | both | 6488.73(5447.71,7732.84) | 259.24(252.90,265.72) | 12851.85(10803.86,15314.97) | 262.06(257.54,266.63) | 2.29(2.12,2.45) | 0.00(-0.03,0.02) |
| Bosnia and Herzegovina | both | 4753.61(3770.99,5978.35) | 250.45(243.37,257.68) | 2556.99(2039.83,3160.59) | 252.86(243.04,263.00) | -1.42(-1.73,-1.12) | 0.18(0.07,0.28) |
| Botswana | both | 1046.27(866.01,1272.73) | 205.14(192.58,218.38) | 2245.63(1842.38,2708.27) | 210.24(201.61,219.14) | 2.49(2.35,2.63) | 0.09(0.05,0.13) |
| Brazil | both | 213320.39(176483.90,258490.64) | 331.18(329.77,332.60) | 274413.72(232213.05,320989.31) | 330.45(329.21,331.70) | 0.93(0.77,1.09) | 0.10(0.03,0.16) |
| Brunei Darussalam | both | 508.92(410.80,630.14) | 416.33(380.83,454.33) | 791.17(641.12,970.97) | 396.94(369.43,426.09) | 1.50(1.41,1.58) | -0.17(-0.20,-0.14) |
| Bulgaria | both | 9103.17(7521.67,11018.94) | 307.58(301.28,313.99) | 6611.18(5528.27,8001.53) | 364.59(355.61,373.75) | -1.12(-1.32,-0.92) | 0.48(0.45,0.50) |
| Burkina Faso | both | 4545.76(3666.79,5615.26) | 147.34(142.99,151.80) | 12905.85(10564.55,15682.32) | 154.47(151.78,157.21) | 3.56(3.51,3.61) | 0.21(0.17,0.25) |
| Burundi | both | 3504.16(2811.14,4362.69) | 169.41(163.78,175.21) | 8742.00(7036.26,10820.26) | 164.79(161.31,168.32) | 3.31(3.04,3.58) | -0.09(-0.13,-0.05) |
| Cabo Verde | both | 202.81(165.86,248.07) | 161.36(138.95,186.91) | 470.99(373.19,583.73) | 185.54(169.13,203.17) | 2.91(2.74,3.09) | 0.57(0.51,0.63) |
| Cambodia | both | 9760.14(7895.12,11761.72) | 249.41(244.40,254.50) | 18090.52(14727.91,22013.32) | 250.47(246.83,254.15) | 2.10(2.01,2.19) | 0.01(-0.02,0.05) |
| Cameroon | both | 5812.13(4727.67,7095.39) | 156.84(152.75,161.02) | 20678.96(17185.10,25113.38) | 164.24(161.98,166.52) | 4.28(4.23,4.33) | 0.19(0.16,0.22) |
| Canada | both | 74120.65(63533.94,85798.59) | 719.68(714.44,724.95) | 85667.05(74993.56,99504.42) | 769.25(764.05,774.49) | 0.63(0.51,0.76) | 0.40(0.31,0.50) |
| Central African Republic | both | 1853.54(1501.89,2263.63) | 180.11(171.84,188.70) | 3874.15(3214.26,4779.68) | 178.72(173.04,184.55) | 2.40(2.25,2.56) | -0.01(-0.03,0.00) |
| Chad | both | 3090.61(2491.32,3829.90) | 150.95(145.57,156.48) | 9276.41(7598.95,11334.51) | 153.19(149.98,156.46) | 3.64(3.55,3.74) | 0.10(0.06,0.14) |
| Chile | both | 20597.17(17184.03,24562.40) | 358.47(353.57,363.41) | 28291.11(23252.85,33779.46) | 417.42(412.52,422.37) | 0.97(0.89,1.06) | 0.41(0.36,0.47) |
| China | both | 2326865.84(1928952.10,2785332.05) | 418.25(417.71,418.79) | 1461472.75(1197417.81,1780772.23) | 320.66(320.12,321.20) | -1.85(-2.05,-1.65) | -1.31(-1.48,-1.13) |
| Colombia | both | 37041.60(31216.39,44316.87) | 262.26(259.57,264.97) | 61177.95(51642.70,73305.62) | 312.03(309.55,314.52) | 1.36(1.14,1.57) | 0.38(0.25,0.50) |
| Comoros | both | 296.48(235.83,371.73) | 171.85(152.20,193.60) | 546.66(441.94,674.15) | 177.22(162.63,192.82) | 1.97(1.92,2.02) | 0.12(0.10,0.14) |
| Congo | both | 1820.40(1476.26,2228.28) | 197.50(188.27,207.10) | 4466.03(3631.06,5456.81) | 203.29(197.35,209.36) | 2.96(2.84,3.07) | 0.15(0.12,0.18) |
| Cook Islands | both | 26.32(21.12,32.55) | 335.07(218.37,496.15) | 20.43(16.49,25.09) | 344.56(211.50,532.39) | -0.98(-1.13,-0.82) | 0.10(0.08,0.12) |
| Costa Rica | both | 3297.23(2748.62,4018.06) | 257.38(248.63,266.38) | 4989.38(4065.69,6138.73) | 264.30(256.97,271.78) | 1.33(1.09,1.56) | 0.12(0.08,0.17) |
| Croatia | both | 6583.57(5441.27,8068.38) | 370.24(361.29,379.37) | 5033.89(4211.96,6036.94) | 413.05(401.53,424.84) | -0.77(-0.89,-0.66) | 0.42(0.29,0.55) |
| Cuba | both | 15990.98(12773.49,20039.47) | 324.80(319.73,329.93) | 11031.34(8897.91,13625.61) | 318.35(312.37,324.42) | -1.30(-1.40,-1.19) | -0.08(-0.10,-0.06) |
| Cyprus | both | 1257.76(1018.51,1560.25) | 409.59(387.23,432.94) | 1974.93(1554.63,2458.01) | 388.70(370.88,407.28) | 1.83(1.73,1.93) | -0.09(-0.16,-0.01) |
| Czechia | both | 15027.02(12349.08,18010.17) | 403.04(396.59,409.57) | 12583.40(10399.20,15120.09) | 451.94(443.88,460.12) | -0.38(-0.76,0.00) | 0.65(0.41,0.89) |
| C?te d'Ivoire | both | 7115.21(5791.65,8859.31) | 154.20(150.57,157.91) | 17533.71(14136.29,21385.13) | 158.74(156.39,161.12) | 2.86(2.71,3.01) | 0.13(0.10,0.15) |
| Democratic People's Republic of Korea | both | 26686.70(21876.91,32361.81) | 318.46(314.63,322.32) | 31680.31(26000.55,38353.34) | 316.18(312.68,319.70) | 0.56(0.52,0.61) | -0.01(-0.04,0.02) |
| Democratic Republic of the Congo | both | 25981.23(21025.42,31823.08) | 184.17(181.89,186.47) | 66190.22(54052.72,81140.60) | 185.46(184.03,186.90) | 3.09(3.02,3.16) | 0.05(0.00,0.10) |
| Denmark | both | 10836.42(9145.81,13000.40) | 570.40(559.68,581.28) | 10298.61(8293.65,12310.89) | 565.69(554.77,576.78) | -0.24(-0.37,-0.12) | -0.01(-0.05,0.03) |
| Djibouti | both | 304.66(242.50,381.04) | 174.28(154.68,195.93) | 986.25(801.41,1210.49) | 182.33(171.12,194.09) | 3.88(3.72,4.04) | 0.17(0.14,0.20) |
| Dominica | both | 123.93(97.13,156.82) | 396.56(328.65,475.85) | 100.62(81.38,122.71) | 387.06(315.13,470.65) | -0.66(-0.75,-0.56) | -0.11(-0.21,-0.01) |
| Dominican Republic | both | 8119.38(6630.38,10197.02) | 262.62(256.83,268.51) | 12985.10(10585.13,16028.90) | 287.42(282.49,292.41) | 1.53(1.50,1.55) | 0.27(0.21,0.33) |
| Ecuador | both | 10569.37(8819.15,12626.83) | 253.31(248.44,258.25) | 18975.46(15748.26,22885.59) | 260.11(256.42,263.84) | 1.91(1.87,1.94) | 0.11(0.10,0.13) |
| Egypt | both | 46381.86(38177.36,57004.60) | 213.85(211.89,215.82) | 94373.36(78501.58,116697.09) | 223.73(222.30,225.16) | 2.40(2.33,2.47) | 0.17(0.12,0.21) |
| El Salvador | both | 4262.86(3544.44,5152.38) | 207.55(201.22,214.05) | 5660.29(4671.48,6989.97) | 219.61(213.90,225.45) | 0.71(0.65,0.77) | 0.00(-0.06,0.05) |
| Equatorial Guinea | both | 278.94(226.15,342.57) | 187.52(165.73,211.54) | 1470.88(1178.63,1800.72) | 215.70(204.63,227.26) | 5.89(5.74,6.03) | 0.65(0.57,0.73) |
| Eritrea | both | 2274.04(1814.97,2857.36) | 175.76(168.42,183.36) | 5138.26(4123.05,6339.45) | 185.09(180.03,190.27) | 3.11(2.94,3.28) | 0.18(0.17,0.19) |
| Estonia | both | 2760.94(2364.45,3291.46) | 495.21(476.82,514.15) | 3138.08(2754.03,3567.85) | 854.62(824.16,885.98) | 0.49(0.22,0.76) | 1.70(1.53,1.87) |
| Eswatini | both | 659.27(546.45,790.36) | 218.04(201.12,236.16) | 1189.68(996.66,1428.74) | 233.69(220.54,247.44) | 1.69(1.50,1.88) | 0.16(0.14,0.19) |
| Ethiopia | both | 30530.05(24788.99,37802.37) | 165.52(163.63,167.42) | 78043.77(63005.96,96606.91) | 167.33(166.13,168.53) | 2.95(2.87,3.02) | 0.04(-0.02,0.09) |
| Fiji | both | 1002.77(800.10,1255.03) | 307.03(288.24,326.80) | 1088.26(878.61,1332.71) | 305.09(287.22,323.78) | 0.24(0.15,0.32) | -0.02(-0.04,-0.01) |
| Finland | both | 9111.87(7439.58,10946.51) | 501.26(490.88,511.83) | 10134.94(8573.70,11837.05) | 614.52(602.50,626.74) | 0.29(0.21,0.38) | 0.51(0.39,0.63) |
| France | both | 102045.25(84245.48,123071.37) | 469.90(467.02,472.80) | 97358.77(79108.53,118598.56) | 491.41(488.32,494.52) | -0.03(-0.07,0.02) | 0.25(0.20,0.30) |
| Gabon | both | 788.15(640.45,976.17) | 209.63(195.05,225.07) | 1570.17(1274.58,1934.16) | 213.13(202.64,224.04) | 2.25(2.23,2.26) | 0.07(0.06,0.09) |
| Gambia | both | 571.30(461.44,702.54) | 155.28(142.52,168.99) | 1519.98(1247.29,1848.62) | 155.92(148.05,164.14) | 3.08(3.03,3.13) | 0.05(0.01,0.08) |
| Georgia | both | 5954.15(4939.52,7246.70) | 279.69(272.62,286.91) | 3130.88(2585.65,3782.41) | 277.41(267.61,287.50) | -1.94(-2.07,-1.81) | 0.13(0.01,0.25) |
| Germany | both | 139017.40(114057.59,172488.05) | 479.47(476.89,482.06) | 133588.16(112316.93,163556.82) | 535.29(532.36,538.23) | -0.44(-0.62,-0.26) | 0.26(0.20,0.31) |
| Ghana | both | 8850.53(7148.17,10985.25) | 157.17(153.87,160.53) | 22622.23(18508.02,27782.41) | 159.58(157.50,161.68) | 3.20(3.14,3.26) | 0.07(0.04,0.10) |
| Greece | both | 13431.95(10776.31,16938.30) | 357.23(351.21,363.32) | 10663.80(8667.33,13208.33) | 384.20(376.84,391.68) | -1.23(-1.63,-0.83) | -0.13(-0.28,0.03) |
| Greenland | both | 142.71(119.40,170.37) | 591.19(494.49,703.05) | 112.02(92.36,136.58) | 577.78(474.80,697.44) | -0.55(-0.66,-0.44) | 0.00(-0.05,0.05) |
| Grenada | both | 121.12(97.51,150.94) | 351.66(291.16,422.07) | 141.28(113.21,173.53) | 355.73(299.27,420.11) | 0.49(0.17,0.82) | 0.16(0.13,0.20) |
| Guam | both | 211.58(170.46,262.64) | 337.02(292.96,386.02) | 198.38(159.85,245.56) | 358.67(310.47,412.30) | -0.21(-0.29,-0.12) | 0.24(0.22,0.26) |
| Guatemala | both | 8014.28(6644.05,9589.23) | 288.39(282.01,294.89) | 16653.43(13596.84,20675.37) | 249.95(246.13,253.81) | 2.53(2.28,2.78) | -0.41(-0.60,-0.22) |
| Guinea | both | 3018.52(2438.10,3726.38) | 149.46(144.14,154.94) | 7675.09(6224.41,9416.20) | 152.57(149.13,156.08) | 2.90(2.79,3.02) | 0.08(0.04,0.12) |
| Guinea-Bissau | both | 538.87(438.44,666.85) | 149.40(136.80,162.93) | 1264.23(1042.71,1535.93) | 153.06(144.66,161.86) | 2.88(2.84,2.93) | 0.10(0.08,0.13) |
| Guyana | both | 977.66(807.63,1192.77) | 282.26(264.60,300.87) | 889.63(729.56,1100.97) | 287.93(269.19,307.71) | -0.35(-0.48,-0.22) | 0.03(-0.01,0.07) |
| Haiti | both | 7011.82(5749.10,8740.60) | 281.68(275.06,288.41) | 16423.80(13248.63,20195.33) | 297.02(292.49,301.60) | 2.75(2.63,2.87) | 0.07(0.04,0.11) |
| Honduras | both | 4347.54(3560.64,5300.69) | 251.88(244.29,259.67) | 11415.79(9308.18,14137.64) | 260.50(255.70,265.36) | 3.19(3.12,3.25) | 0.07(0.05,0.08) |
| Hungary | both | 11934.73(9839.92,14361.79) | 327.20(321.30,333.19) | 9119.50(7514.22,11024.09) | 340.09(333.05,347.24) | -0.75(-0.94,-0.56) | 0.25(0.16,0.34) |
| Iceland | both | 513.61(417.20,625.83) | 495.64(453.65,540.54) | 714.19(604.59,839.79) | 602.70(558.99,649.09) | 1.24(1.16,1.32) | 0.70(0.65,0.75) |
| India | both | 705810.16(560533.11,878072.72) | 206.41(205.93,206.90) | 1389128.72(1127333.74,1688849.76) | 227.70(227.32,228.08) | 2.30(2.19,2.40) | 0.34(0.20,0.47) |
| Indonesia | both | 172798.39(139185.51,210816.78) | 219.65(218.61,220.70) | 262219.13(211588.99,316179.19) | 231.25(230.37,232.14) | 1.15(1.04,1.26) | 0.07(-0.04,0.17) |
| Iran (Islamic Republic of) | both | 65610.45(55696.55,77589.57) | 304.69(302.31,307.08) | 127164.36(106485.88,150778.74) | 376.35(374.21,378.49) | 2.21(1.61,2.82) | 0.60(0.41,0.80) |
| Iraq | both | 15814.66(13177.52,19371.69) | 222.16(218.62,225.74) | 39243.97(32616.74,47980.88) | 225.70(223.46,227.95) | 2.94(2.76,3.13) | 0.07(0.06,0.08) |
| Ireland | both | 7362.61(6140.44,8819.77) | 522.30(510.39,534.43) | 9403.95(7979.50,11052.26) | 601.61(589.41,614.00) | 0.86(0.68,1.05) | 0.32(0.24,0.39) |
| Israel | both | 8431.81(6932.81,10168.80) | 440.07(430.70,449.59) | 13885.11(11164.27,17060.87) | 417.25(410.34,424.26) | 1.75(1.66,1.84) | -0.06(-0.10,-0.01) |
| Italy | both | 120333.97(99646.29,145768.14) | 567.88(564.67,571.10) | 72262.61(59173.84,86752.67) | 463.58(460.17,467.01) | -1.64(-1.77,-1.51) | -0.70(-0.80,-0.59) |
| Jamaica | both | 3390.02(2779.76,4215.71) | 332.44(321.15,344.06) | 3982.14(3261.13,4840.51) | 344.60(333.94,355.52) | 0.50(0.38,0.61) | 0.00(-0.04,0.05) |
| Japan | both | 180500.36(144904.28,224188.41) | 401.65(399.80,403.52) | 122678.30(98625.28,149108.99) | 386.94(384.76,389.14) | -1.35(-1.43,-1.27) | -0.13(-0.15,-0.11) |
| Jordan | both | 3445.52(2829.84,4153.19) | 226.50(218.60,234.65) | 12122.28(9951.36,14909.09) | 226.29(222.27,230.37) | 4.24(4.01,4.47) | 0.06(0.04,0.09) |
| Kazakhstan | both | 25060.40(20726.60,30279.80) | 369.18(364.61,373.78) | 30706.35(25783.98,36199.70) | 437.94(432.98,442.93) | 0.84(0.75,0.93) | 0.45(0.33,0.57) |
| Kenya | both | 11572.02(9360.62,14129.47) | 136.42(133.85,139.03) | 32177.37(26049.47,38884.77) | 149.14(147.50,150.81) | 3.48(3.38,3.59) | 0.43(0.31,0.54) |
| Kiribati | both | 93.54(75.65,115.19) | 301.72(243.20,371.16) | 155.18(127.13,186.63) | 309.52(262.69,362.52) | 1.83(1.74,1.93) | 0.08(0.06,0.10) |
| Kuwait | both | 2151.60(1720.19,2631.35) | 252.42(241.69,263.54) | 5684.15(4517.90,7119.87) | 264.93(257.51,272.53) | 4.00(3.69,4.30) | 0.27(0.21,0.33) |
| Kyrgyzstan | both | 5477.39(4566.48,6565.55) | 303.75(295.72,311.96) | 8086.77(6736.31,9751.26) | 297.82(291.35,304.41) | 1.33(1.23,1.43) | -0.06(-0.09,-0.03) |
| Lao People's Democratic Republic | both | 3938.76(3206.57,4770.92) | 251.76(243.85,259.89) | 8200.34(6705.71,9889.19) | 255.90(250.38,261.50) | 2.45(2.28,2.62) | 0.03(-0.01,0.08) |
| Latvia | both | 4088.00(3444.46,4946.62) | 435.02(421.73,448.64) | 2391.26(1997.13,2875.00) | 462.18(443.28,481.74) | -1.89(-2.10,-1.68) | 0.00(-0.07,0.07) |
| Lebanon | both | 2862.96(2350.67,3470.74) | 250.58(241.40,260.05) | 6761.11(5592.12,8174.28) | 293.25(286.14,300.51) | 3.26(2.97,3.54) | 0.57(0.55,0.59) |
| Lesotho | both | 1127.03(938.43,1352.92) | 207.58(195.45,220.30) | 1808.54(1505.10,2139.78) | 217.27(207.29,227.62) | 1.48(1.40,1.55) | 0.14(0.11,0.17) |
| Liberia | both | 1468.02(1189.16,1812.74) | 161.10(152.90,169.66) | 3742.60(3059.48,4617.92) | 167.04(161.68,172.55) | 4.26(3.92,4.60) | 0.11(0.09,0.13) |
| Libya | both | 4084.70(3340.55,4971.66) | 251.19(243.33,259.25) | 8963.31(7357.94,11015.14) | 294.84(288.73,301.04) | 2.89(2.49,3.28) | 0.63(0.56,0.71) |
| Lithuania | both | 5200.65(4364.62,6182.90) | 373.56(363.46,383.89) | 4246.76(3623.91,4962.53) | 527.53(511.51,543.96) | -0.95(-1.13,-0.77) | 0.96(0.70,1.23) |
| Luxembourg | both | 806.65(663.03,986.70) | 560.76(521.84,602.08) | 1278.88(1037.18,1532.09) | 592.75(559.84,627.26) | 1.35(1.19,1.52) | 0.01(-0.08,0.10) |
| Madagascar | both | 7760.12(6163.42,9697.84) | 171.36(167.49,175.31) | 20043.24(16122.42,24797.19) | 171.49(169.08,173.93) | 3.10(3.06,3.14) | 0.01(-0.02,0.04) |
| Malawi | both | 6325.81(5055.74,7896.40) | 169.50(165.23,173.87) | 13993.99(11220.24,17472.07) | 169.96(167.06,172.89) | 2.54(2.30,2.78) | 0.02(-0.02,0.05) |
| Malaysia | both | 20663.55(16783.10,25296.05) | 278.51(274.72,282.35) | 37646.50(30123.42,45998.60) | 270.85(268.12,273.61) | 2.00(1.84,2.16) | -0.06(-0.09,-0.04) |
| Maldives | both | 227.90(183.48,280.12) | 276.17(240.12,316.96) | 736.01(584.29,906.22) | 289.61(267.34,313.54) | 4.09(3.98,4.19) | 0.17(0.14,0.20) |
| Mali | both | 4378.39(3555.78,5436.93) | 149.91(145.44,154.49) | 12934.61(10571.84,15752.16) | 150.36(147.69,153.07) | 3.67(3.52,3.81) | 0.03(-0.02,0.07) |
| Malta | both | 549.83(447.07,690.23) | 398.16(365.33,433.28) | 641.68(522.60,778.30) | 487.12(449.05,527.88) | 0.64(0.46,0.82) | 0.60(0.49,0.71) |
| Marshall Islands | both | 57.80(46.39,72.06) | 327.11(247.03,426.90) | 80.45(64.57,96.77) | 335.98(266.50,418.38) | 0.89(0.69,1.08) | 0.09(0.08,0.10) |
| Mauritania | both | 1173.35(951.05,1444.69) | 156.10(147.17,165.47) | 2630.07(2146.85,3207.35) | 158.36(152.26,164.66) | 2.62(2.58,2.67) | 0.10(0.06,0.14) |
| Mauritius | both | 1539.69(1269.82,1851.60) | 310.73(295.37,326.69) | 1790.34(1488.34,2212.81) | 394.55(376.45,413.33) | 0.58(0.41,0.75) | 0.92(0.82,1.03) |
| Mexico | both | 81555.20(68648.00,97830.03) | 227.76(226.17,229.36) | 114575.55(95931.76,138429.91) | 222.64(221.35,223.93) | 1.25(1.15,1.35) | 0.15(0.05,0.26) |
| Micronesia (Federated States of) | both | 132.92(106.66,165.00) | 320.07(267.14,381.16) | 140.28(112.46,171.98) | 322.87(271.27,381.97) | -0.02(-0.12,0.09) | 0.03(0.02,0.03) |
| Monaco | both | 44.95(36.06,55.86) | 499.17(360.59,680.18) | 49.75(40.45,61.13) | 532.88(394.36,706.11) | 0.30(0.23,0.36) | 0.25(0.22,0.28) |
| Mongolia | both | 2660.20(2220.15,3174.99) | 295.68(284.29,307.46) | 3759.51(3111.33,4536.73) | 304.07(294.32,314.08) | 1.16(0.96,1.36) | 0.16(0.14,0.19) |
| Montenegro | both | 790.50(638.45,975.52) | 315.13(293.54,337.91) | 633.01(507.06,778.91) | 307.07(283.47,332.18) | -0.61(-0.67,-0.55) | -0.01(-0.07,0.04) |
| Morocco | both | 24416.47(20121.28,29562.45) | 235.95(232.96,238.96) | 36390.78(29834.42,43624.29) | 247.59(245.05,250.15) | 1.29(1.17,1.41) | 0.17(0.16,0.19) |
| Mozambique | both | 7951.17(6378.63,9881.00) | 166.54(162.84,170.31) | 22201.14(17785.43,27481.62) | 188.26(185.71,190.83) | 3.35(3.25,3.44) | 0.45(0.43,0.47) |
| Myanmar | both | 47469.88(38388.85,56873.88) | 271.94(269.48,274.43) | 61547.29(49928.46,74669.85) | 272.46(270.31,274.62) | 0.72(0.63,0.81) | 0.01(-0.01,0.03) |
| Namibia | both | 1240.24(1034.03,1521.99) | 221.71(209.19,234.86) | 2424.00(2005.00,2914.02) | 231.86(222.69,241.33) | 2.11(1.93,2.29) | 0.16(0.14,0.18) |
| Nauru | both | 13.21(10.66,16.25) | 322.28(172.10,556.22) | 15.39(12.49,18.93) | 325.15(182.99,539.34) | 0.33(0.27,0.40) | 0.01(-0.04,0.07) |
| Nepal | both | 14343.97(11529.96,17956.62) | 196.84(193.60,200.13) | 26958.59(21637.25,33550.41) | 201.64(199.23,204.09) | 2.02(1.96,2.08) | 0.04(0.01,0.08) |
| Netherlands | both | 28693.90(23563.42,34475.18) | 492.86(487.14,498.63) | 24260.46(20106.15,29425.11) | 465.19(459.34,471.11) | -0.58(-0.71,-0.46) | -0.18(-0.25,-0.11) |
| New Zealand | both | 9898.05(8011.58,12027.38) | 720.37(706.25,734.71) | 14156.33(11463.11,17198.45) | 815.92(802.44,829.59) | 0.74(0.58,0.91) | 0.03(-0.10,0.17) |
| Nicaragua | both | 3479.47(2902.02,4220.30) | 237.51(229.48,245.79) | 6105.51(5006.87,7423.10) | 214.96(209.59,220.42) | 1.89(1.76,2.01) | -0.25(-0.29,-0.22) |
| Niger | both | 4096.59(3309.04,5027.76) | 151.21(146.51,156.03) | 12946.21(10601.98,15666.29) | 150.83(148.12,153.58) | 3.61(3.51,3.71) | 0.02(-0.02,0.06) |
| Nigeria | both | 45274.02(36605.99,55142.04) | 136.37(135.09,137.66) | 125146.68(101669.10,152102.09) | 144.58(143.76,145.40) | 3.30(3.26,3.35) | 0.24(0.17,0.31) |
| Niue | both | 2.73(2.19,3.40) | 330.34(59.85,1060.91) | 1.96(1.58,2.39) | 340.88(39.70,1287.92) | -1.38(-1.67,-1.08) | 0.13(0.11,0.15) |
| North Macedonia | both | 2220.31(1801.91,2697.93) | 279.60(268.09,291.49) | 2248.92(1815.87,2769.14) | 295.23(282.88,308.02) | 0.48(0.30,0.66) | 0.41(0.32,0.51) |
| Northern Mariana Islands | both | 75.95(62.03,93.57) | 331.61(260.64,417.15) | 58.58(47.86,71.13) | 353.50(268.59,458.00) | -1.54(-2.21,-0.87) | 0.25(0.19,0.30) |
| Norway | both | 8099.65(6601.70,10047.67) | 505.52(494.55,516.68) | 10578.19(8792.69,12681.32) | 600.14(588.67,611.79) | 0.72(0.64,0.79) | 0.33(0.16,0.50) |
| Oman | both | 1834.54(1497.52,2272.53) | 221.13(211.07,231.56) | 5181.87(4143.69,6482.95) | 221.63(215.13,228.32) | 3.67(3.49,3.86) | 0.04(0.03,0.06) |
| Pakistan | both | 83777.47(66556.03,104419.13) | 204.25(202.84,205.67) | 201508.47(161753.61,248155.15) | 203.84(202.94,204.73) | 3.09(2.92,3.27) | 0.11(-0.02,0.24) |
| Palau | both | 23.07(18.82,28.25) | 328.73(208.37,495.79) | 19.89(16.29,24.13) | 340.93(207.15,530.76) | -0.84(-1.22,-0.45) | 0.13(0.12,0.14) |
| Palestine | both | 1789.41(1475.93,2193.20) | 233.67(222.51,245.33) | 5107.40(4190.15,6250.79) | 233.64(227.21,240.23) | 3.49(3.31,3.68) | -0.03(-0.04,-0.01) |
| Panama | both | 2872.76(2368.42,3484.61) | 281.29(271.00,291.90) | 4338.85(3542.06,5327.57) | 263.71(255.91,271.68) | 1.29(1.22,1.36) | -0.15(-0.21,-0.09) |
| Papua New Guinea | both | 5208.24(4174.04,6450.76) | 306.76(298.37,315.35) | 13360.05(10801.35,16430.37) | 309.75(304.51,315.07) | 3.20(3.15,3.26) | 0.03(0.01,0.05) |
| Paraguay | both | 3897.68(3239.50,4647.23) | 247.57(239.80,255.54) | 7705.21(6379.45,9235.24) | 251.68(246.09,257.37) | 2.32(2.25,2.38) | 0.05(0.02,0.09) |
| Peru | both | 22322.50(18443.44,26802.18) | 251.42(248.08,254.79) | 37761.65(30826.34,45198.28) | 254.33(251.77,256.92) | 1.64(1.61,1.67) | 0.07(0.05,0.09) |
| Philippines | both | 69193.19(56152.77,84300.70) | 262.82(260.84,264.82) | 118285.04(96483.54,144022.89) | 248.69(247.27,250.11) | 1.69(1.64,1.73) | -0.24(-0.27,-0.21) |
| Poland | both | 49565.89(40910.46,59505.41) | 353.38(350.24,356.54) | 39901.37(32342.80,47938.46) | 350.76(347.20,354.36) | -0.66(-0.80,-0.51) | -0.05(-0.11,0.00) |
| Portugal | both | 15897.15(13087.60,19380.87) | 418.15(411.67,424.70) | 11344.47(8997.58,13950.84) | 386.51(379.37,393.76) | -1.20(-1.28,-1.11) | -0.26(-0.37,-0.15) |
| Puerto Rico | both | 6805.49(5573.74,8308.32) | 479.73(468.38,491.28) | 4611.71(3731.80,5644.16) | 446.16(433.36,459.26) | -1.47(-1.79,-1.14) | -0.44(-0.59,-0.29) |
| Qatar | both | 542.42(435.26,683.67) | 226.31(206.48,247.88) | 3835.68(2996.35,4812.43) | 226.33(217.59,235.47) | 8.49(7.49,9.49) | 0.07(0.04,0.11) |
| Republic of Korea | both | 84293.38(68485.57,105908.02) | 400.43(397.72,403.15) | 62505.94(50422.85,77041.98) | 403.50(400.25,406.77) | -0.89(-0.98,-0.80) | 0.02(-0.01,0.04) |
| Republic of Moldova | both | 6241.29(5172.94,7512.11) | 362.73(353.71,371.92) | 4063.19(3299.88,4937.91) | 335.84(325.07,346.91) | -1.23(-1.37,-1.09) | -0.25(-0.30,-0.21) |
| Romania | both | 20688.26(16648.90,25472.40) | 237.42(234.17,240.71) | 12998.40(10326.34,16563.99) | 236.30(232.20,240.46) | -1.48(-1.63,-1.32) | 0.01(-0.09,0.10) |
| Russian Federation | both | 308810.28(261641.89,366963.89) | 550.14(548.18,552.11) | 257102.90(218122.51,300438.83) | 572.24(569.94,574.55) | -0.92(-1.46,-0.39) | -0.29(-0.66,0.08) |
| Rwanda | both | 4868.20(3906.95,6069.93) | 176.34(171.35,181.45) | 10885.20(8745.78,13580.24) | 188.41(184.85,192.01) | 3.12(2.70,3.54) | 0.27(0.22,0.32) |
| Saint Kitts and Nevis | both | 58.34(46.95,72.61) | 332.82(252.42,432.61) | 75.88(61.57,93.22) | 339.83(267.21,426.93) | 1.00(0.88,1.12) | 0.09(0.07,0.11) |
| Saint Lucia | both | 241.40(190.90,299.56) | 400.64(350.66,456.70) | 231.58(185.69,287.65) | 361.14(315.85,411.33) | 0.23(0.00,0.47) | 0.04(-0.06,0.13) |
| Saint Vincent and the Grenadines | both | 155.98(124.08,196.52) | 328.68(277.92,387.26) | 142.08(114.21,174.76) | 345.94(291.37,407.89) | -0.45(-0.53,-0.36) | 0.12(0.09,0.15) |
| Samoa | both | 241.03(190.36,312.29) | 337.16(294.37,385.31) | 309.14(253.73,375.75) | 366.12(326.08,410.09) | 0.59(0.52,0.66) | 0.16(0.09,0.23) |
| San Marino | both | 45.19(36.71,55.62) | 484.09(353.11,648.89) | 46.36(38.03,57.44) | 517.61(379.18,690.96) | -0.13(-0.28,0.03) | 0.23(0.19,0.26) |
| Sao Tome and Principe | both | 65.23(52.75,79.99) | 157.49(120.20,204.13) | 144.97(117.57,177.05) | 161.84(136.39,190.81) | 2.64(2.61,2.68) | 0.12(0.06,0.17) |
| Saudi Arabia | both | 15338.67(12580.77,18799.23) | 232.42(228.73,236.16) | 44942.30(36411.12,55766.21) | 238.88(236.59,241.19) | 3.94(3.74,4.14) | 0.19(0.15,0.23) |
| Senegal | both | 4174.91(3402.25,5143.73) | 155.73(150.92,160.67) | 9875.18(8099.61,12160.38) | 157.06(153.92,160.24) | 2.73(2.67,2.80) | 0.05(0.01,0.09) |
| Serbia | both | 10348.55(8298.53,12859.02) | 288.55(283.00,294.18) | 8858.52(7290.60,10779.89) | 298.21(291.97,304.56) | -0.60(-0.73,-0.46) | 0.22(0.16,0.27) |
| Seychelles | both | 109.41(89.27,134.11) | 343.98(281.96,417.07) | 120.71(99.86,145.67) | 321.31(266.11,385.04) | 0.45(0.28,0.62) | -0.05(-0.14,0.04) |
| Sierra Leone | both | 2445.32(1982.03,3046.74) | 155.44(149.28,161.81) | 5679.92(4681.52,7039.46) | 155.72(151.63,159.89) | 3.23(2.92,3.54) | 0.03(-0.02,0.08) |
| Singapore | both | 6085.22(4870.72,7604.89) | 408.59(398.32,419.07) | 7466.33(5911.30,9271.78) | 406.70(396.74,416.88) | 1.10(0.77,1.43) | -0.03(-0.06,0.00) |
| Slovakia | both | 7201.58(5947.99,8726.04) | 355.10(346.91,363.44) | 5484.12(4497.21,6665.96) | 332.95(323.91,342.20) | -0.90(-1.18,-0.62) | -0.28(-0.39,-0.17) |
| Slovenia | both | 2764.86(2241.66,3402.92) | 372.77(358.91,387.04) | 2357.84(1978.51,2819.30) | 433.58(415.79,451.99) | -0.32(-0.56,-0.08) | 0.73(0.57,0.88) |
| Solomon Islands | both | 394.94(313.97,497.89) | 290.87(261.87,322.63) | 827.12(676.55,1004.48) | 293.50(273.69,314.45) | 2.24(2.14,2.34) | 0.01(-0.02,0.03) |
| Somalia | both | 4931.14(3926.62,6135.20) | 167.37(162.66,172.19) | 13638.51(10899.46,17128.93) | 162.15(159.36,164.99) | 3.68(3.55,3.80) | -0.09(-0.14,-0.05) |
| South Africa | both | 48084.41(41196.57,55841.35) | 301.93(299.21,304.67) | 70431.04(59431.04,83065.59) | 293.11(290.94,295.29) | 0.95(0.65,1.24) | -0.37(-0.52,-0.23) |
| South Sudan | both | 4035.70(3214.57,5044.89) | 175.25(169.70,180.95) | 6265.51(5002.40,7892.54) | 170.84(166.45,175.33) | 1.58(1.27,1.89) | -0.05(-0.07,-0.03) |
| Spain | both | 90042.15(78257.22,103429.27) | 604.47(600.52,608.43) | 72558.21(61307.37,84545.66) | 602.23(597.79,606.68) | -0.87(-1.23,-0.51) | -0.23(-0.44,-0.01) |
| Sri Lanka | both | 22054.03(18223.75,26722.68) | 297.68(293.76,301.64) | 23228.85(18891.96,28057.86) | 287.94(284.25,291.67) | 0.03(-0.04,0.11) | -0.16(-0.21,-0.12) |
| Sudan | both | 16236.62(13323.30,19583.13) | 216.03(212.65,219.46) | 42017.42(35010.62,50128.91) | 229.02(226.82,231.25) | 3.08(3.04,3.11) | 0.26(0.23,0.29) |
| Suriname | both | 517.82(419.61,647.54) | 315.10(288.05,344.27) | 692.55(567.26,837.43) | 322.37(298.80,347.34) | 1.15(0.95,1.35) | 0.16(0.13,0.18) |
| Sweden | both | 12309.92(9815.53,15485.97) | 417.40(410.04,424.87) | 18705.80(15297.99,22844.43) | 579.93(571.55,588.41) | 1.59(1.47,1.71) | 1.29(1.21,1.36) |
| Switzerland | both | 19784.24(16934.25,23246.66) | 795.30(784.06,806.67) | 16986.79(14121.99,20516.33) | 629.01(619.32,638.82) | -0.54(-0.67,-0.40) | -0.89(-0.98,-0.80) |
| Syrian Arab Republic | both | 10009.98(8182.14,12168.19) | 212.31(208.03,216.66) | 11321.11(9366.93,13604.66) | 222.56(218.04,227.18) | 0.49(-0.49,1.47) | 0.18(0.13,0.22) |
| Taiwan (Province of China) | both | 33485.84(26760.45,41188.07) | 361.57(357.70,365.47) | 30949.07(24942.78,37894.55) | 401.15(396.58,405.76) | -0.31(-0.42,-0.21) | 0.21(0.13,0.29) |
| Tajikistan | both | 6111.07(5076.82,7348.02) | 286.08(278.82,293.51) | 11391.85(9409.32,13822.59) | 273.14(268.13,278.21) | 2.25(2.15,2.35) | -0.13(-0.18,-0.08) |
| Thailand | both | 80200.90(65236.57,96683.17) | 304.70(302.59,306.83) | 65569.36(54069.88,78678.90) | 315.83(313.40,318.27) | -0.79(-1.02,-0.56) | 0.08(-0.04,0.20) |
| Timor-Leste | both | 849.73(694.52,1031.99) | 264.46(246.85,283.09) | 1572.00(1265.50,1925.64) | 268.23(254.69,282.40) | 2.02(1.81,2.23) | 0.06(0.04,0.09) |
| Togo | both | 1886.87(1529.11,2354.61) | 142.94(136.41,149.74) | 4814.90(3930.81,5875.61) | 146.34(142.21,150.56) | 3.09(3.06,3.12) | 0.14(0.09,0.19) |
| Tokelau | both | 1.90(1.52,2.36) | 317.76(34.10,1296.16) | 1.64(1.32,2.01) | 327.12(27.85,1386.80) | -0.58(-0.90,-0.25) | 0.12(0.10,0.14) |
| Tonga | both | 121.90(96.53,156.58) | 311.88(256.72,377.13) | 131.95(107.47,160.42) | 326.85(272.77,389.15) | 0.14(0.05,0.24) | 0.12(0.11,0.13) |
| Trinidad and Tobago | both | 1528.15(1251.01,1900.95) | 304.42(289.31,320.14) | 1534.95(1252.10,1863.14) | 312.53(296.83,328.87) | -0.06(-0.24,0.12) | 0.13(0.11,0.14) |
| Tunisia | both | 7692.19(6299.52,9429.93) | 226.06(220.97,231.26) | 10842.58(8730.24,13258.27) | 246.90(242.23,251.65) | 1.14(0.89,1.38) | 0.34(0.31,0.37) |
| Türkiye | both | 51648.98(41424.40,63282.24) | 218.25(216.36,220.16) | 69368.92(55146.94,86094.69) | 215.96(214.35,217.58) | 0.92(0.81,1.02) | 0.01(-0.02,0.04) |
| Turkmenistan | both | 4529.54(3786.96,5432.66) | 293.09(284.53,301.87) | 6851.52(5775.84,8190.88) | 329.95(322.18,337.87) | 1.43(1.39,1.48) | 0.46(0.40,0.51) |
| Tuvalu | both | 11.10(9.03,13.66) | 306.64(153.55,550.33) | 16.04(13.08,19.52) | 318.62(181.90,522.39) | 1.35(1.19,1.51) | 0.11(0.10,0.12) |
| Uganda | both | 12158.73(9727.33,15449.46) | 184.21(180.82,187.66) | 31760.23(25647.79,39081.31) | 181.38(179.32,183.47) | 3.19(3.13,3.26) | -0.02(-0.04,0.01) |
| Ukraine | both | 65993.63(55030.01,78338.73) | 348.75(346.08,351.44) | 57887.47(49180.03,67999.03) | 435.49(431.79,439.22) | -0.29(-0.39,-0.18) | 0.76(0.68,0.84) |
| United Arab Emirates | both | 2707.04(2159.05,3367.50) | 275.94(265.12,287.16) | 13467.47(10267.28,17174.97) | 323.28(315.91,330.81) | 7.05(5.87,8.23) | 0.61(0.53,0.68) |
| United Kingdom | both | 130614.65(107252.63,155533.19) | 645.20(641.69,648.73) | 155026.47(128791.49,183812.39) | 734.60(730.91,738.31) | 0.60(0.52,0.69) | 0.36(0.24,0.49) |
| United Republic of Tanzania | both | 18748.81(15237.57,22767.75) | 188.88(186.10,191.69) | 44155.46(35664.57,54975.86) | 189.69(187.89,191.50) | 2.75(2.69,2.81) | 0.04(0.00,0.07) |
| United States of America | both | 665491.50(553859.46,795345.23) | 687.41(685.74,689.08) | 1180842.97(1029274.35,1357884.76) | 1096.05(1094.07,1098.04) | 1.96(1.66,2.27) | 1.54(1.23,1.85) |
| United States Virgin Islands | both | 153.24(122.34,191.53) | 379.52(321.62,445.17) | 93.34(76.86,116.24) | 398.87(321.63,489.94) | -1.46(-1.72,-1.20) | 0.19(0.16,0.22) |
| Uruguay | both | 4456.50(3690.40,5354.35) | 389.61(378.25,401.23) | 4585.30(3832.09,5399.51) | 388.57(377.39,400.00) | 0.12(0.04,0.19) | 0.02(0.00,0.04) |
| Uzbekistan | both | 24261.19(20228.27,29375.71) | 280.90(277.34,284.49) | 37837.83(31065.51,45787.06) | 277.39(274.58,280.21) | 1.62(1.49,1.75) | 0.00(-0.05,0.04) |
| Vanuatu | both | 174.87(140.70,217.65) | 291.42(249.37,339.04) | 371.76(305.13,453.11) | 293.19(263.95,324.96) | 2.46(2.40,2.51) | 0.01(-0.01,0.02) |
| Venezuela (Bolivarian Republic of) | both | 19030.64(15505.06,23288.76) | 238.67(235.27,242.11) | 21768.67(17925.33,26303.62) | 231.54(228.44,234.67) | 1.13(0.76,1.50) | -0.05(-0.07,-0.04) |
| Viet Nam | both | 82987.73(68400.69,100248.18) | 288.73(286.75,290.72) | 124323.19(101775.49,150184.74) | 328.28(326.43,330.13) | 1.45(1.29,1.61) | 0.48(0.41,0.55) |
| Yemen | both | 9513.65(7842.49,11550.00) | 210.24(205.98,214.58) | 29445.05(24355.15,35223.24) | 215.96(213.48,218.45) | 3.88(3.74,4.02) | 0.17(0.13,0.20) |
| Zambia | both | 5626.30(4596.95,6869.87) | 182.40(177.42,187.51) | 13534.67(11140.86,16519.30) | 170.62(167.71,173.57) | 3.19(3.00,3.39) | -0.04(-0.11,0.03) |
| Zimbabwe | both | 9380.64(7698.54,11450.28) | 239.79(234.79,244.87) | 14954.46(12366.04,18088.49) | 238.05(234.20,241.94) | 1.27(1.17,1.36) | -0.09(-0.12,-0.07) |
| Afghanistan | female | 4789.22(3901.36,5783.82) | 271.90(263.50,280.56) | 16410.70(13564.09,19413.87) | 274.61(270.23,279.06) | 3.90(3.65,4.14) | 0.21(0.10,0.32) |
| Albania | female | 1665.75(1304.16,2109.23) | 238.74(227.33,250.62) | 1102.67(882.97,1400.65) | 244.99(230.68,260.00) | -1.42(-1.47,-1.37) | 0.13(0.07,0.18) |
| Algeria | female | 11120.93(9099.30,13799.15) | 228.11(223.78,232.52) | 20386.63(16218.33,25266.80) | 239.10(235.79,242.44) | 2.13(1.87,2.38) | 0.24(0.21,0.26) |
| American Samoa | female | 28.67(22.94,35.42) | 284.06(188.74,414.64) | 26.32(21.00,32.59) | 305.44(199.25,450.12) | -0.44(-0.68,-0.20) | 0.30(0.27,0.34) |
| Andorra | female | 46.66(35.49,61.31) | 403.14(294.25,542.74) | 54.03(42.27,69.20) | 429.92(320.35,568.25) | 0.14(-0.21,0.49) | 0.26(0.23,0.29) |
| Angola | female | 2886.47(2332.98,3581.28) | 152.45(146.87,158.21) | 9716.74(7803.42,12043.58) | 156.95(153.80,160.15) | 4.09(4.05,4.14) | 0.15(0.12,0.19) |
| Antigua and Barbuda | female | 37.45(30.39,46.12) | 283.59(199.97,391.65) | 48.20(39.07,60.10) | 281.17(206.99,374.64) | 0.80(0.52,1.09) | 0.05(0.02,0.07) |
| Argentina | female | 17984.84(14583.00,22996.53) | 292.14(287.89,296.45) | 25713.99(20434.03,32511.41) | 291.20(287.64,294.79) | 1.33(1.26,1.40) | 0.08(0.05,0.12) |
| Armenia | female | 2023.64(1654.67,2506.51) | 278.11(266.05,290.60) | 1457.97(1176.26,1807.36) | 272.20(257.85,287.22) | -0.84(-0.94,-0.75) | -0.01(-0.05,0.04) |
| Australia | female | 26192.18(21643.78,31918.09) | 785.42(775.91,795.01) | 31254.03(24915.12,39283.79) | 711.73(703.74,719.81) | 0.62(0.57,0.66) | -0.22(-0.32,-0.13) |
| Austria | female | 5479.78(4335.38,6949.18) | 373.91(363.99,384.04) | 6251.35(4937.73,7843.97) | 456.74(445.28,468.45) | 0.36(0.30,0.43) | 0.66(0.61,0.70) |
| Azerbaijan | female | 4293.50(3515.62,5306.45) | 264.02(256.10,272.14) | 5329.69(4264.02,6606.39) | 256.45(249.45,263.63) | 0.86(0.78,0.95) | -0.03(-0.10,0.04) |
| Bahamas | female | 181.23(149.11,219.59) | 303.22(260.48,351.39) | 246.28(201.91,302.63) | 310.84(273.21,352.27) | 0.99(0.90,1.09) | 0.15(0.09,0.21) |
| Bahrain | female | 237.07(189.41,297.75) | 235.20(205.86,267.84) | 579.96(456.74,727.46) | 237.55(218.55,257.82) | 3.64(3.25,4.03) | 0.12(0.09,0.15) |
| Bangladesh | female | 40930.94(33016.22,50976.87) | 202.53(200.52,204.56) | 76139.72(60086.61,94590.10) | 213.38(211.86,214.90) | 1.96(1.85,2.06) | 0.15(0.12,0.17) |
| Barbados | female | 177.52(144.01,218.86) | 326.64(280.29,378.61) | 154.89(125.94,189.80) | 316.71(268.51,371.35) | -0.32(-0.40,-0.23) | -0.06(-0.11,0.00) |
| Belarus | female | 6793.12(5388.27,8646.06) | 341.83(333.69,350.13) | 4951.63(3862.90,6341.38) | 334.44(324.74,344.38) | -0.92(-1.02,-0.82) | 0.01(-0.03,0.05) |
| Belgium | female | 7058.86(5626.55,9025.68) | 390.94(381.80,400.25) | 7092.19(5591.49,8819.59) | 407.18(397.66,416.89) | 0.08(-0.02,0.18) | 0.15(0.11,0.20) |
| Belize | female | 108.65(89.27,131.68) | 287.35(234.34,350.43) | 262.86(216.63,324.79) | 272.20(240.18,307.45) | 3.04(2.97,3.11) | -0.12(-0.16,-0.09) |
| Benin | female | 1303.42(1052.38,1671.12) | 143.16(135.39,151.30) | 3747.09(3037.26,4619.14) | 142.17(137.56,146.90) | 3.49(3.48,3.51) | 0.01(-0.04,0.05) |
| Bermuda | female | 37.55(29.92,47.55) | 294.83(206.09,412.95) | 26.78(21.61,33.43) | 302.90(197.00,449.22) | -1.06(-1.17,-0.96) | 0.12(0.09,0.16) |
| Bhutan | female | 224.43(181.11,281.34) | 185.58(161.39,212.73) | 322.56(260.03,406.16) | 195.66(174.87,218.29) | 1.55(1.38,1.73) | 0.15(0.12,0.18) |
| Bolivia (Plurinational State of) | female | 3051.91(2540.98,3686.99) | 242.72(234.10,251.60) | 6051.75(5019.47,7389.42) | 248.14(241.92,254.48) | 2.26(2.12,2.41) | 0.02(0.00,0.05) |
| Bosnia and Herzegovina | female | 2090.31(1602.23,2689.02) | 227.55(217.89,237.53) | 1157.96(899.57,1475.29) | 230.33(217.10,244.20) | -1.51(-1.73,-1.28) | 0.10(-0.01,0.22) |
| Botswana | female | 488.56(394.97,603.19) | 182.78(166.59,200.25) | 989.53(787.57,1235.36) | 183.25(171.99,195.08) | 2.32(2.22,2.43) | 0.03(-0.02,0.07) |
| Brazil | female | 94614.46(79633.15,113923.10) | 291.94(290.07,293.81) | 116813.54(98953.92,138599.38) | 278.09(276.49,279.70) | 0.74(0.61,0.86) | -0.12(-0.17,-0.06) |
| Brunei Darussalam | female | 224.68(178.14,282.86) | 393.32(343.41,448.75) | 355.91(286.39,445.27) | 385.08(345.69,428.01) | 1.49(1.32,1.67) | -0.06(-0.08,-0.04) |
| Bulgaria | female | 3716.51(2946.47,4685.23) | 251.20(243.16,259.44) | 2527.30(2049.63,3163.50) | 276.73(265.72,288.11) | -1.27(-1.45,-1.10) | 0.27(0.25,0.30) |
| Burkina Faso | female | 2266.59(1795.42,2859.19) | 135.37(129.77,141.16) | 5979.88(4799.33,7453.37) | 133.55(130.14,137.03) | 3.26(3.21,3.32) | -0.02(-0.08,0.03) |
| Burundi | female | 1490.91(1183.77,1909.13) | 141.33(134.16,148.82) | 3577.46(2848.32,4476.59) | 135.64(131.17,140.23) | 3.15(2.87,3.42) | -0.15(-0.20,-0.09) |
| Cabo Verde | female | 97.57(79.15,121.18) | 146.67(118.27,180.65) | 181.20(142.48,228.50) | 149.16(128.21,172.63) | 2.10(2.02,2.18) | 0.10(0.06,0.14) |
| Cambodia | female | 4677.45(3741.60,5760.24) | 223.04(216.63,229.60) | 8099.23(6509.28,9987.70) | 225.36(220.47,230.33) | 1.87(1.83,1.91) | 0.01(-0.04,0.06) |
| Cameroon | female | 2809.73(2250.07,3487.62) | 144.67(139.26,150.26) | 9127.99(7401.45,11344.84) | 142.49(139.55,145.47) | 3.94(3.91,3.97) | -0.03(-0.08,0.01) |
| Canada | female | 35289.25(29728.02,42378.98) | 683.25(676.02,690.53) | 38147.14(32314.22,45512.77) | 687.87(680.88,694.92) | 0.41(0.30,0.53) | 0.21(0.12,0.30) |
| Central African Republic | female | 774.73(619.98,955.83) | 147.74(137.32,158.81) | 1619.78(1313.70,1978.13) | 144.56(137.50,151.90) | 2.45(2.36,2.54) | -0.04(-0.07,-0.01) |
| Chad | female | 1513.85(1201.53,1911.96) | 140.24(133.16,147.61) | 4279.49(3428.69,5294.41) | 136.22(132.06,140.49) | 3.44(3.34,3.54) | -0.06(-0.12,-0.01) |
| Chile | female | 9434.89(7732.60,11857.90) | 326.01(319.45,332.67) | 12550.22(10216.32,15302.06) | 369.75(363.24,376.35) | 0.89(0.83,0.95) | 0.37(0.33,0.40) |
| China | female | 1092175.10(904513.35,1311416.14) | 405.54(404.77,406.31) | 655126.17(527343.14,809214.67) | 295.71(294.97,296.46) | -1.89(-2.08,-1.69) | -1.39(-1.53,-1.24) |
| Colombia | female | 17267.94(14360.99,21148.72) | 239.23(235.64,242.85) | 25298.76(21023.99,30655.86) | 257.65(254.48,260.86) | 1.04(0.89,1.20) | 0.17(0.10,0.23) |
| Comoros | female | 122.35(96.12,153.84) | 141.96(117.29,170.75) | 216.78(171.79,270.76) | 141.64(123.36,161.97) | 1.82(1.79,1.85) | -0.01(-0.05,0.04) |
| Congo | female | 722.38(584.87,894.71) | 155.79(144.35,167.96) | 1849.36(1497.06,2300.72) | 164.74(157.29,172.44) | 3.24(3.12,3.35) | 0.32(0.27,0.36) |
| Cook Islands | female | 10.91(8.79,13.58) | 290.89(143.45,533.67) | 9.51(7.59,11.86) | 301.76(141.34,567.90) | -0.59(-0.71,-0.47) | 0.14(0.12,0.16) |
| Costa Rica | female | 1443.92(1189.05,1791.02) | 224.08(212.61,236.04) | 2264.02(1826.00,2791.93) | 230.83(221.36,240.63) | 1.46(1.26,1.66) | 0.13(0.09,0.17) |
| Croatia | female | 2560.33(2064.52,3174.80) | 290.68(279.44,302.26) | 1840.36(1479.03,2274.32) | 304.77(290.76,319.33) | -0.81(-0.93,-0.70) | 0.42(0.30,0.54) |
| Cuba | female | 6746.28(5423.06,8385.21) | 278.48(271.81,285.28) | 4650.92(3692.77,5840.56) | 271.87(264.02,279.91) | -1.39(-1.51,-1.27) | -0.09(-0.11,-0.07) |
| Cyprus | female | 513.67(399.85,659.52) | 342.19(313.18,373.24) | 820.79(615.55,1073.38) | 314.29(292.03,338.08) | 1.93(1.79,2.07) | -0.16(-0.26,-0.06) |
| Czechia | female | 6156.14(4947.70,7544.06) | 334.44(326.09,342.96) | 5112.14(4161.56,6326.49) | 366.68(356.43,377.17) | -0.40(-0.77,-0.02) | 0.55(0.30,0.79) |
| C?te d'Ivoire | female | 3206.95(2575.57,4002.45) | 141.61(136.62,146.75) | 7530.32(6032.99,9512.80) | 138.17(135.05,141.34) | 2.67(2.55,2.79) | -0.07(-0.11,-0.02) |
| Democratic People's Republic of Korea | female | 13358.09(10783.55,16394.52) | 301.22(296.08,306.42) | 14243.13(11468.31,17623.48) | 296.50(291.63,301.43) | 0.10(0.05,0.14) | -0.05(-0.10,-0.01) |
| Democratic Republic of the Congo | female | 10438.81(8486.12,12919.66) | 148.88(145.99,151.82) | 25782.66(21035.07,31889.28) | 148.41(146.57,150.26) | 3.01(2.91,3.12) | 0.04(-0.02,0.10) |
| Denmark | female | 4352.29(3403.86,5749.78) | 466.36(452.57,480.48) | 4188.85(3267.43,5277.91) | 467.93(453.81,482.40) | -0.20(-0.28,-0.13) | 0.04(0.00,0.07) |
| Djibouti | female | 114.40(89.55,144.73) | 142.32(116.68,172.54) | 360.21(282.31,453.50) | 142.49(128.12,158.06) | 3.80(3.64,3.96) | -0.01(-0.05,0.04) |
| Dominica | female | 47.26(37.78,58.46) | 324.01(236.67,436.31) | 41.84(33.91,51.91) | 330.76(238.19,447.85) | -0.35(-0.46,-0.24) | 0.09(-0.01,0.19) |
| Dominican Republic | female | 3649.16(2951.11,4517.72) | 230.47(222.92,238.23) | 5702.39(4613.16,7122.25) | 254.48(247.91,261.18) | 1.42(1.39,1.46) | 0.34(0.29,0.38) |
| Ecuador | female | 4974.09(4123.29,6042.13) | 238.02(231.36,244.82) | 8992.71(7404.84,10978.90) | 246.61(241.54,251.77) | 1.92(1.88,1.97) | 0.14(0.12,0.15) |
| Egypt | female | 23942.46(19448.05,30208.19) | 225.98(223.10,228.88) | 46302.52(37723.07,57422.39) | 225.23(223.19,227.29) | 2.24(2.14,2.35) | 0.03(0.00,0.05) |
| El Salvador | female | 2089.00(1694.26,2567.47) | 197.06(188.53,205.91) | 2763.88(2245.94,3431.40) | 203.46(195.92,211.23) | 0.76(0.71,0.82) | -0.01(-0.05,0.03) |
| Equatorial Guinea | female | 121.05(97.23,150.11) | 150.73(124.74,180.79) | 492.80(396.90,615.04) | 165.61(151.21,181.08) | 4.94(4.76,5.12) | 0.50(0.42,0.59) |
| Eritrea | female | 924.66(730.81,1160.18) | 142.96(133.73,152.70) | 1863.12(1506.17,2330.96) | 139.73(133.42,146.27) | 2.68(2.55,2.81) | -0.10(-0.15,-0.05) |
| Estonia | female | 1123.09(927.13,1376.42) | 401.30(378.00,425.72) | 1048.84(878.67,1264.29) | 578.67(543.23,615.95) | -0.03(-0.15,0.08) | 1.26(1.11,1.40) |
| Eswatini | female | 308.93(253.34,381.31) | 190.41(169.18,213.84) | 495.15(403.47,610.93) | 190.66(174.20,208.32) | 1.37(1.20,1.54) | -0.02(-0.04,0.01) |
| Ethiopia | female | 12637.97(10226.08,15538.82) | 133.31(130.96,135.69) | 29930.03(24254.67,36571.58) | 129.65(128.16,131.15) | 2.73(2.63,2.82) | -0.11(-0.19,-0.03) |
| Fiji | female | 441.90(356.32,552.58) | 276.56(251.26,303.84) | 475.52(385.93,583.54) | 272.37(248.43,298.01) | 0.26(0.20,0.32) | -0.04(-0.06,-0.01) |
| Finland | female | 3761.31(2925.20,4815.70) | 417.27(403.85,431.05) | 3989.82(3198.46,4960.02) | 488.68(473.49,504.26) | 0.18(0.13,0.24) | 0.43(0.34,0.52) |
| France | female | 43640.90(34936.32,55198.28) | 402.13(398.36,405.93) | 41738.07(32530.88,52180.11) | 419.43(415.40,423.49) | -0.01(-0.06,0.03) | 0.25(0.20,0.31) |
| Gabon | female | 300.39(242.27,371.92) | 162.79(144.49,182.95) | 643.35(518.82,800.62) | 164.99(152.38,178.41) | 2.52(2.51,2.53) | 0.11(0.09,0.13) |
| Gambia | female | 275.75(218.99,349.60) | 144.76(127.77,163.65) | 702.10(567.13,877.21) | 139.09(128.84,149.99) | 2.95(2.91,3.00) | -0.11(-0.16,-0.06) |
| Georgia | female | 2853.62(2309.10,3508.06) | 262.19(252.62,272.05) | 1435.59(1156.60,1788.21) | 258.91(245.38,273.04) | -2.14(-2.23,-2.05) | 0.08(-0.01,0.16) |
| Germany | female | 55055.11(42344.54,71628.61) | 384.42(381.14,387.72) | 52653.25(40538.95,67995.37) | 437.02(433.22,440.86) | -0.44(-0.59,-0.29) | 0.29(0.22,0.37) |
| Ghana | female | 4248.60(3416.39,5339.60) | 146.36(141.93,150.90) | 10851.24(8674.05,13609.87) | 147.34(144.57,150.15) | 3.21(3.14,3.28) | 0.04(0.01,0.08) |
| Greece | female | 5703.73(4363.48,7359.24) | 303.55(295.72,311.54) | 4586.81(3586.59,5910.26) | 323.57(314.12,333.26) | -0.81(-1.02,-0.59) | 0.17(0.08,0.26) |
| Greenland | female | 63.75(52.67,77.35) | 569.52(433.79,738.31) | 53.80(44.03,66.08) | 564.20(422.64,739.82) | -0.32(-0.43,-0.21) | 0.06(0.01,0.12) |
| Grenada | female | 50.20(40.15,62.78) | 299.32(221.53,397.65) | 57.52(46.74,71.87) | 302.10(228.87,392.01) | 0.45(0.08,0.83) | 0.16(0.11,0.22) |
| Guam | female | 83.21(67.21,103.34) | 292.00(232.53,362.34) | 78.09(62.63,97.10) | 295.22(233.38,368.61) | -0.30(-0.46,-0.15) | 0.05(0.04,0.06) |
| Guatemala | female | 3293.72(2628.47,4029.09) | 226.22(218.43,234.23) | 7619.90(6220.23,9396.36) | 219.27(214.34,224.29) | 2.76(2.64,2.89) | -0.19(-0.24,-0.14) |
| Guinea | female | 1513.84(1203.94,1949.79) | 137.89(130.99,145.08) | 3642.91(2887.40,4534.63) | 134.04(129.68,138.52) | 2.79(2.67,2.92) | -0.10(-0.16,-0.05) |
| Guinea-Bissau | female | 261.60(210.62,328.21) | 137.63(121.22,155.79) | 579.11(465.28,720.00) | 134.16(123.39,145.67) | 2.72(2.65,2.80) | -0.08(-0.14,-0.03) |
| Guyana | female | 418.95(342.79,511.78) | 242.31(219.35,267.22) | 384.40(316.78,469.34) | 246.39(222.23,272.58) | -0.37(-0.52,-0.22) | 0.03(-0.01,0.07) |
| Haiti | female | 3121.73(2588.51,3819.59) | 241.01(232.57,249.70) | 6948.07(5692.96,8519.01) | 246.13(240.38,251.99) | 2.61(2.49,2.73) | -0.02(-0.06,0.02) |
| Honduras | female | 1820.60(1512.55,2249.45) | 208.35(198.70,218.37) | 4783.97(3909.08,5894.05) | 210.52(204.57,216.61) | 3.20(3.16,3.25) | -0.03(-0.07,0.00) |
| Hungary | female | 5010.81(4013.92,6273.99) | 274.57(266.93,282.38) | 3677.05(2959.03,4640.18) | 273.66(264.75,282.80) | -0.81(-1.02,-0.61) | 0.12(0.02,0.22) |
| Iceland | female | 216.28(170.86,280.39) | 424.64(369.86,485.42) | 313.68(257.18,387.72) | 545.28(486.30,609.79) | 1.39(1.30,1.49) | 0.93(0.87,0.98) |
| India | female | 278489.66(221636.68,345850.60) | 170.44(169.81,171.08) | 570446.77(461463.28,698046.74) | 193.06(192.56,193.56) | 2.14(1.99,2.29) | 0.16(-0.03,0.36) |
| Indonesia | female | 85881.66(68907.92,105155.74) | 215.53(214.08,216.99) | 119625.74(95128.33,145383.10) | 215.25(214.03,216.48) | 0.91(0.82,0.99) | -0.06(-0.13,0.02) |
| Iran (Islamic Republic of) | female | 26880.90(22181.30,32700.37) | 254.37(251.28,257.50) | 46033.47(37063.42,56932.57) | 266.99(264.47,269.53) | 1.89(1.43,2.34) | 0.10(0.00,0.20) |
| Iraq | female | 7496.31(6119.19,9425.45) | 221.69(216.58,226.91) | 18114.48(14661.95,22707.39) | 218.38(215.20,221.60) | 2.91(2.78,3.04) | 0.00(-0.02,0.02) |
| Ireland | female | 3056.95(2485.70,3807.98) | 441.11(425.57,457.08) | 3960.87(3136.51,4970.62) | 494.02(478.56,509.86) | 1.04(0.88,1.21) | 0.28(0.24,0.32) |
| Israel | female | 3407.46(2697.12,4325.93) | 356.28(344.39,368.47) | 5727.79(4555.77,7250.56) | 349.19(340.20,358.36) | 1.78(1.71,1.84) | 0.00(-0.06,0.05) |
| Italy | female | 46686.64(38104.35,57651.78) | 445.64(441.60,449.71) | 29031.93(23434.16,35735.62) | 378.87(374.47,383.31) | -1.51(-1.67,-1.35) | -0.55(-0.67,-0.42) |
| Jamaica | female | 1439.63(1180.26,1775.62) | 280.64(266.10,295.84) | 1721.25(1388.06,2094.85) | 295.33(281.48,309.70) | 0.50(0.39,0.61) | 0.06(0.01,0.10) |
| Japan | female | 85129.77(67999.34,106144.77) | 384.53(381.94,387.13) | 59415.03(47311.87,73499.33) | 379.17(376.09,382.28) | -1.24(-1.34,-1.15) | -0.06(-0.08,-0.05) |
| Jordan | female | 1616.57(1286.56,1970.41) | 230.15(218.55,242.28) | 5661.49(4552.20,7041.52) | 233.03(226.97,239.22) | 4.21(4.05,4.38) | 0.12(0.09,0.15) |
| Kazakhstan | female | 10602.44(8649.74,13122.88) | 314.01(308.05,320.07) | 11918.81(9512.38,14804.25) | 339.85(333.68,346.11) | 0.70(0.54,0.85) | 0.30(0.24,0.36) |
| Kenya | female | 5014.31(4054.96,6254.13) | 118.54(115.14,122.01) | 12717.34(10172.88,15724.48) | 116.90(114.85,118.98) | 3.09(3.02,3.16) | 0.01(-0.09,0.12) |
| Kiribati | female | 41.13(33.34,50.38) | 262.89(188.22,359.39) | 66.42(53.95,80.44) | 260.79(201.83,331.94) | 1.71(1.63,1.78) | -0.04(-0.09,0.01) |
| Kuwait | female | 849.31(677.40,1054.57) | 238.65(222.78,255.40) | 2542.27(1987.20,3249.89) | 236.86(226.99,247.13) | 4.52(4.03,5.02) | 0.07(0.04,0.10) |
| Kyrgyzstan | female | 2470.47(2012.03,3032.08) | 274.92(264.13,286.06) | 3586.89(2933.05,4390.54) | 264.53(255.92,273.37) | 1.31(1.24,1.38) | -0.08(-0.12,-0.04) |
| Lao People's Democratic Republic | female | 1941.15(1564.00,2392.60) | 238.97(228.30,250.06) | 3890.97(3144.83,4767.31) | 244.66(237.03,252.49) | 2.32(2.14,2.50) | 0.05(-0.02,0.12) |
| Latvia | female | 1733.60(1402.38,2136.92) | 366.66(349.51,384.48) | 935.66(745.65,1181.92) | 356.79(333.55,381.35) | -2.05(-2.27,-1.84) | -0.17(-0.23,-0.11) |
| Lebanon | female | 1469.16(1181.03,1800.40) | 245.50(233.04,258.50) | 2924.83(2333.14,3656.29) | 259.53(249.96,269.40) | 2.68(2.46,2.91) | 0.25(0.21,0.29) |
| Lesotho | female | 543.52(445.75,678.25) | 180.46(165.47,196.50) | 753.60(618.79,910.51) | 181.87(169.02,195.50) | 1.01(0.97,1.05) | 0.01(-0.03,0.06) |
| Liberia | female | 689.75(555.03,865.96) | 147.41(136.49,159.02) | 1641.83(1312.93,2029.73) | 146.65(139.57,154.00) | 3.83(3.55,4.11) | -0.05(-0.09,0.00) |
| Libya | female | 1814.23(1476.56,2256.69) | 241.14(229.71,253.06) | 4025.92(3244.71,5052.88) | 272.62(264.24,281.21) | 2.78(2.38,3.17) | 0.52(0.43,0.60) |
| Lithuania | female | 2157.77(1701.42,2728.39) | 309.22(296.27,322.60) | 1313.60(1018.05,1650.13) | 327.52(309.76,346.13) | -1.89(-1.99,-1.79) | 0.08(-0.08,0.24) |
| Luxembourg | female | 305.64(242.56,388.63) | 425.48(378.05,477.87) | 527.44(411.72,657.72) | 488.79(446.79,534.08) | 1.64(1.54,1.73) | 0.29(0.19,0.39) |
| Madagascar | female | 3193.13(2523.97,4061.44) | 142.13(137.14,147.28) | 8223.04(6507.48,10270.01) | 139.54(136.49,142.65) | 3.08(3.03,3.13) | -0.07(-0.11,-0.02) |
| Malawi | female | 2666.93(2104.73,3322.63) | 142.34(136.84,148.04) | 5762.52(4628.76,7196.57) | 137.65(134.03,141.35) | 2.46(2.21,2.71) | -0.14(-0.20,-0.07) |
| Malaysia | female | 8430.30(6776.30,10453.83) | 227.77(222.92,232.70) | 15327.96(12031.42,19172.39) | 231.01(227.36,234.71) | 1.95(1.83,2.06) | 0.05(0.01,0.09) |
| Maldives | female | 99.22(79.36,123.84) | 244.26(196.62,301.89) | 216.96(170.48,273.11) | 245.31(212.95,281.62) | 2.70(2.48,2.93) | -0.02(-0.05,0.01) |
| Mali | female | 2150.31(1729.95,2681.33) | 138.79(132.92,144.87) | 6050.29(4874.57,7560.63) | 136.40(132.90,139.98) | 3.54(3.37,3.72) | -0.06(-0.11,-0.01) |
| Malta | female | 237.26(184.49,304.55) | 345.98(302.99,393.66) | 244.55(189.74,312.02) | 376.47(329.46,429.02) | 0.25(-0.02,0.51) | 0.24(0.03,0.46) |
| Marshall Islands | female | 24.79(20.03,30.51) | 293.38(187.89,441.37) | 35.19(28.40,42.96) | 305.09(212.56,425.11) | 0.99(0.80,1.17) | 0.16(0.14,0.17) |
| Mauritania | female | 556.98(446.87,696.10) | 144.77(132.84,157.54) | 1227.37(990.29,1514.45) | 142.61(134.62,150.98) | 2.54(2.50,2.59) | -0.02(-0.07,0.03) |
| Mauritius | female | 621.96(503.37,760.30) | 254.69(235.03,275.59) | 613.73(497.12,754.26) | 272.90(251.70,295.47) | -0.03(-0.14,0.07) | 0.30(0.27,0.32) |
| Mexico | female | 39234.84(32767.69,46878.56) | 214.87(212.71,217.06) | 53747.55(44462.04,64992.40) | 205.21(203.48,206.95) | 1.13(1.03,1.23) | 0.06(-0.05,0.18) |
| Micronesia (Federated States of) | female | 56.64(45.75,69.92) | 282.86(212.94,370.21) | 59.37(48.15,72.48) | 286.60(217.86,371.26) | -0.05(-0.15,0.05) | 0.06(0.05,0.07) |
| Monaco | female | 18.50(14.32,23.80) | 403.40(236.82,659.86) | 20.94(16.51,26.92) | 444.55(273.55,687.99) | 0.45(0.41,0.48) | 0.37(0.34,0.40) |
| Mongolia | female | 1172.06(963.08,1423.92) | 265.34(250.00,281.50) | 1677.75(1363.44,2071.80) | 268.25(255.42,281.60) | 1.22(1.05,1.40) | 0.14(0.09,0.18) |
| Montenegro | female | 315.13(244.13,398.83) | 259.41(231.55,289.73) | 259.75(202.02,333.35) | 255.32(224.98,288.81) | -0.49(-0.55,-0.43) | 0.04(-0.02,0.10) |
| Morocco | female | 12342.61(9935.07,15003.96) | 236.30(232.10,240.56) | 17343.81(13859.69,21126.60) | 236.89(233.37,240.44) | 1.13(0.97,1.28) | 0.05(0.04,0.07) |
| Mozambique | female | 3557.69(2820.92,4450.56) | 138.53(133.95,143.23) | 8628.22(6932.68,10669.00) | 137.87(134.89,140.91) | 2.80(2.70,2.91) | -0.03(-0.09,0.02) |
| Myanmar | female | 19899.01(16168.01,24213.43) | 227.00(223.83,230.21) | 27030.05(21635.45,33329.64) | 235.26(232.46,238.09) | 0.80(0.72,0.89) | 0.07(0.01,0.12) |
| Namibia | female | 548.46(449.72,681.00) | 194.23(177.91,211.79) | 1055.70(852.99,1292.85) | 198.48(186.66,210.89) | 2.04(1.89,2.19) | 0.08(0.06,0.10) |
| Nauru | female | 5.71(4.61,7.05) | 282.86(100.04,643.57) | 6.53(5.29,8.09) | 282.89(108.90,608.68) | 0.28(0.22,0.35) | -0.01(-0.06,0.04) |
| Nepal | female | 6984.77(5645.64,8699.39) | 186.50(182.10,190.98) | 13876.55(11185.21,17178.12) | 191.38(188.20,194.60) | 2.28(2.25,2.30) | 0.05(0.00,0.09) |
| Netherlands | female | 12151.52(9825.88,15162.76) | 422.84(415.32,430.48) | 10466.90(8422.44,13094.81) | 404.81(397.06,412.68) | -0.55(-0.67,-0.44) | -0.15(-0.22,-0.07) |
| New Zealand | female | 3921.79(3240.27,4746.86) | 569.34(551.65,587.45) | 5671.66(4569.90,6969.97) | 662.86(645.57,680.51) | 0.91(0.77,1.05) | 0.27(0.18,0.36) |
| Nicaragua | female | 1639.35(1350.88,2003.58) | 217.25(206.61,228.35) | 2858.04(2310.16,3587.49) | 200.72(193.43,208.22) | 1.84(1.72,1.97) | -0.21(-0.24,-0.18) |
| Niger | female | 1966.70(1572.62,2477.45) | 140.24(133.97,146.77) | 5907.07(4750.25,7325.16) | 137.08(133.45,140.80) | 3.47(3.39,3.56) | -0.06(-0.11,-0.01) |
| Nigeria | female | 21165.93(17285.14,26002.56) | 127.21(125.45,129.00) | 56914.04(46053.26,70066.86) | 123.47(122.43,124.51) | 3.18(3.11,3.25) | -0.06(-0.15,0.03) |
| Niue | female | 1.09(0.88,1.35) | 283.70(9.15,1554.23) | 0.81(0.65,1.01) | 292.09(3.50,1933.00) | -1.28(-1.61,-0.95) | 0.13(0.11,0.15) |
| North Macedonia | female | 903.12(704.36,1154.32) | 231.79(216.92,247.43) | 897.50(696.74,1132.54) | 237.90(222.26,254.46) | 0.36(0.24,0.48) | 0.29(0.23,0.36) |
| Northern Mariana Islands | female | 33.71(27.01,42.14) | 289.18(199.44,407.35) | 23.20(18.63,28.57) | 292.89(185.68,442.68) | -2.27(-3.21,-1.32) | 0.05(0.04,0.06) |
| Norway | female | 3283.29(2611.58,4168.15) | 418.07(403.87,432.65) | 4452.99(3613.24,5502.83) | 512.54(497.48,527.96) | 0.79(0.74,0.85) | 0.43(0.30,0.55) |
| Oman | female | 645.85(515.95,817.33) | 228.18(210.81,246.69) | 1843.74(1437.07,2333.95) | 230.46(219.83,241.51) | 3.57(3.44,3.71) | 0.08(0.06,0.09) |
| Pakistan | female | 39226.50(31588.00,48244.54) | 202.01(199.97,204.07) | 95118.51(76773.35,116102.10) | 194.50(193.26,195.74) | 3.22(2.93,3.52) | 0.05(-0.17,0.27) |
| Palau | female | 9.49(7.70,11.69) | 284.75(133.27,537.19) | 7.02(5.67,8.53) | 290.83(116.27,613.96) | -1.35(-1.72,-0.97) | 0.07(0.06,0.08) |
| Palestine | female | 885.01(709.18,1098.58) | 236.70(220.78,253.60) | 2477.49(1977.95,3070.18) | 232.75(223.58,242.22) | 3.57(3.40,3.73) | -0.02(-0.04,-0.01) |
| Panama | female | 1208.95(993.96,1502.37) | 240.16(226.69,254.28) | 1890.87(1535.58,2377.92) | 233.58(223.16,244.36) | 1.45(1.38,1.52) | 0.01(-0.05,0.07) |
| Papua New Guinea | female | 2169.44(1753.77,2683.72) | 267.59(256.29,279.30) | 5629.01(4583.58,6947.96) | 269.08(262.09,276.22) | 3.21(3.16,3.25) | 0.01(-0.02,0.05) |
| Paraguay | female | 1792.00(1483.90,2155.07) | 230.90(220.24,241.96) | 3491.69(2887.23,4265.45) | 232.45(224.80,240.30) | 2.26(2.20,2.31) | 0.03(-0.01,0.06) |
| Peru | female | 10756.93(8824.31,13096.29) | 241.33(236.73,246.00) | 17976.33(14634.28,21992.66) | 243.78(240.22,247.38) | 1.63(1.60,1.66) | 0.08(0.06,0.09) |
| Philippines | female | 29339.67(23728.62,35549.11) | 225.18(222.58,227.81) | 51822.57(41690.74,62850.92) | 224.05(222.12,226.00) | 1.83(1.79,1.86) | -0.03(-0.08,0.02) |
| Poland | female | 19573.52(15681.26,23959.90) | 277.52(273.59,281.49) | 15756.07(12471.58,19538.73) | 268.59(264.24,273.00) | -0.60(-0.71,-0.50) | -0.15(-0.20,-0.10) |
| Portugal | female | 6891.61(5475.71,8794.07) | 362.51(354.00,371.18) | 5088.24(3929.71,6565.28) | 340.35(330.96,349.96) | -1.01(-1.10,-0.92) | -0.18(-0.27,-0.09) |
| Puerto Rico | female | 2568.05(2040.40,3177.81) | 351.90(338.42,365.79) | 1929.12(1512.90,2437.42) | 365.55(349.37,382.31) | -0.88(-1.13,-0.63) | 0.17(0.10,0.25) |
| Qatar | female | 153.69(120.65,196.75) | 232.70(196.88,273.58) | 1030.08(801.85,1331.59) | 238.28(222.56,254.99) | 7.21(6.72,7.71) | 0.20(0.16,0.24) |
| Republic of Korea | female | 39370.32(31529.78,49753.18) | 383.51(379.72,387.33) | 29146.37(23030.71,36565.72) | 391.46(386.86,396.11) | -0.93(-1.01,-0.85) | 0.06(0.05,0.08) |
| Republic of Moldova | female | 2753.27(2182.49,3454.30) | 308.87(297.34,320.77) | 1801.79(1400.16,2292.94) | 290.77(276.80,305.34) | -1.17(-1.26,-1.09) | -0.15(-0.24,-0.07) |
| Romania | female | 9577.89(7495.86,12130.58) | 221.88(217.42,226.41) | 5927.73(4526.88,7757.71) | 218.69(213.08,224.42) | -1.53(-1.71,-1.35) | -0.06(-0.13,0.01) |
| Russian Federation | female | 125548.80(103639.26,153732.86) | 440.98(438.51,443.47) | 100471.44(83560.98,121513.04) | 437.78(434.95,440.62) | -0.82(-1.17,-0.47) | -0.23(-0.45,-0.01) |
| Rwanda | female | 1994.39(1575.62,2524.78) | 144.56(138.19,151.17) | 4272.37(3378.94,5421.80) | 147.89(143.45,152.42) | 2.86(2.56,3.16) | 0.08(0.04,0.12) |
| Saint Kitts and Nevis | female | 24.21(19.69,30.08) | 277.05(177.37,416.49) | 32.17(26.23,40.32) | 286.09(195.40,406.32) | 1.08(0.98,1.18) | 0.14(0.12,0.16) |
| Saint Lucia | female | 100.06(80.74,124.20) | 331.41(268.49,406.49) | 94.91(76.80,118.27) | 297.93(240.64,365.39) | 0.14(-0.08,0.37) | 0.01(-0.08,0.10) |
| Saint Vincent and the Grenadines | female | 61.93(49.47,78.29) | 268.92(204.73,349.48) | 54.74(44.88,66.90) | 270.94(203.90,353.21) | -0.50(-0.56,-0.44) | -0.01(-0.03,0.00) |
| Samoa | female | 92.88(74.01,117.57) | 291.27(233.38,360.73) | 122.44(99.80,150.06) | 305.83(253.64,366.27) | 0.82(0.77,0.87) | 0.19(0.15,0.22) |
| San Marino | female | 18.66(14.79,23.56) | 396.23(236.95,624.81) | 19.36(15.00,24.86) | 425.15(256.67,666.24) | -0.05(-0.30,0.19) | 0.28(0.23,0.32) |
| Sao Tome and Principe | female | 30.70(24.49,38.42) | 147.05(98.38,213.96) | 63.03(50.31,78.55) | 142.33(109.15,182.79) | 2.33(2.30,2.36) | -0.12(-0.17,-0.06) |
| Saudi Arabia | female | 6469.56(5118.04,8061.35) | 237.25(231.39,243.22) | 19572.37(15534.22,24489.27) | 251.25(247.65,254.89) | 4.05(3.86,4.25) | 0.28(0.24,0.32) |
| Senegal | female | 2027.95(1642.35,2518.33) | 144.83(138.45,151.45) | 4426.59(3587.27,5623.54) | 140.67(136.51,144.92) | 2.45(2.40,2.51) | -0.10(-0.14,-0.06) |
| Serbia | female | 4023.86(3083.33,5131.39) | 227.92(220.92,235.10) | 3160.05(2480.48,4039.30) | 219.95(212.28,227.85) | -0.82(-0.95,-0.68) | 0.00(-0.11,0.10) |
| Seychelles | female | 43.97(35.17,54.21) | 282.85(204.31,385.81) | 46.34(36.50,57.11) | 270.02(197.76,360.62) | 0.16(0.01,0.31) | -0.02(-0.08,0.03) |
| Sierra Leone | female | 1219.38(974.12,1542.67) | 144.90(136.78,153.40) | 2607.97(2106.01,3266.67) | 139.54(134.14,145.10) | 2.83(2.56,3.10) | -0.13(-0.19,-0.07) |
| Singapore | female | 2867.18(2289.22,3640.14) | 392.59(378.22,407.41) | 3710.04(2924.60,4781.89) | 396.11(382.26,410.41) | 1.25(0.97,1.53) | 0.03(0.01,0.05) |
| Slovakia | female | 2948.13(2337.62,3606.12) | 291.63(281.15,302.42) | 2315.71(1832.44,2883.71) | 277.41(265.85,289.38) | -0.67(-0.93,-0.41) | -0.14(-0.25,-0.03) |
| Slovenia | female | 1097.00(866.79,1386.36) | 298.88(281.34,317.27) | 851.42(674.73,1065.84) | 315.67(294.24,338.36) | -0.49(-0.70,-0.29) | 0.58(0.40,0.75) |
| Solomon Islands | female | 167.45(135.47,206.30) | 259.00(219.89,303.95) | 356.27(287.99,432.23) | 259.09(232.76,287.70) | 2.34(2.24,2.43) | -0.02(-0.06,0.02) |
| Somalia | female | 1941.23(1540.13,2463.77) | 140.15(133.93,146.59) | 5244.08(4205.33,6622.60) | 133.43(129.74,137.20) | 3.57(3.45,3.70) | -0.19(-0.26,-0.12) |
| South Africa | female | 21953.28(18660.50,25719.73) | 272.29(268.66,275.96) | 29108.86(24219.48,35217.53) | 241.10(238.33,243.90) | 0.60(0.24,0.95) | -0.64(-0.85,-0.43) |
| South Sudan | female | 1562.94(1234.32,1976.74) | 145.13(137.79,152.80) | 2598.40(2037.31,3245.70) | 140.84(135.33,146.53) | 1.81(1.53,2.08) | -0.11(-0.14,-0.07) |
| Spain | female | 33940.89(28453.50,41017.17) | 463.73(458.81,468.70) | 30017.17(25255.82,36405.36) | 499.43(493.70,505.20) | -0.70(-1.07,-0.33) | -0.13(-0.37,0.11) |
| Sri Lanka | female | 9050.92(7320.58,11173.16) | 244.79(239.77,249.90) | 10378.16(8311.44,12743.19) | 251.76(246.94,256.66) | 0.37(0.31,0.42) | 0.08(0.04,0.11) |
| Sudan | female | 8262.10(6729.09,10123.19) | 213.26(208.61,218.00) | 20276.43(16567.21,24999.84) | 220.69(217.65,223.77) | 2.96(2.94,2.98) | 0.18(0.15,0.21) |
| Suriname | female | 206.39(169.56,257.85) | 260.46(225.65,299.60) | 286.05(234.81,348.39) | 264.55(234.75,297.13) | 1.28(1.10,1.47) | 0.14(0.11,0.18) |
| Sweden | female | 4939.58(3837.78,6309.89) | 341.47(331.99,351.17) | 6928.24(5509.08,8890.40) | 433.93(423.64,444.41) | 1.33(1.22,1.44) | 1.02(0.93,1.10) |
| Switzerland | female | 7772.28(6243.65,9472.48) | 637.18(622.85,651.78) | 7348.61(5848.53,9083.85) | 547.78(534.95,560.86) | -0.21(-0.28,-0.13) | -0.59(-0.64,-0.53) |
| Syrian Arab Republic | female | 5287.00(4261.98,6449.70) | 226.86(220.59,233.29) | 6657.60(5456.12,8181.93) | 232.52(226.56,238.62) | 0.81(-0.04,1.65) | 0.10(0.08,0.12) |
| Taiwan (Province of China) | female | 14952.22(11874.43,18601.16) | 329.35(324.08,334.68) | 12840.42(10065.13,16247.32) | 338.91(332.91,344.99) | -0.49(-0.58,-0.40) | -0.01(-0.08,0.06) |
| Tajikistan | female | 2771.30(2270.49,3413.27) | 261.19(251.37,271.34) | 5183.76(4261.27,6331.90) | 251.71(244.89,258.67) | 2.26(2.15,2.36) | -0.07(-0.13,-0.01) |
| Thailand | female | 30642.72(24834.76,37554.35) | 234.31(231.69,236.96) | 26368.78(20944.29,32447.67) | 243.78(240.82,246.78) | -0.62(-0.79,-0.44) | 0.13(0.10,0.17) |
| Timor-Leste | female | 357.35(287.39,443.79) | 230.36(206.91,255.94) | 681.59(550.14,839.85) | 235.85(217.96,254.98) | 2.12(1.89,2.35) | 0.08(0.05,0.12) |
| Togo | female | 918.35(733.74,1169.54) | 132.72(124.12,141.79) | 2211.66(1736.04,2780.99) | 129.01(123.68,134.52) | 2.93(2.91,2.96) | -0.04(-0.11,0.02) |
| Tokelau | female | 0.80(0.65,0.98) | 275.60(2.96,2001.49) | 0.68(0.54,0.83) | 279.86(1.50,2166.99) | -0.77(-1.19,-0.34) | 0.06(0.04,0.08) |
| Tonga | female | 53.82(42.87,68.74) | 285.65(212.36,378.84) | 60.80(49.19,74.89) | 299.96(228.87,387.09) | 0.28(0.20,0.35) | 0.10(0.08,0.12) |
| Trinidad and Tobago | female | 646.86(526.59,800.08) | 259.67(240.00,280.58) | 637.00(518.92,788.25) | 259.09(239.02,280.48) | -0.07(-0.22,0.08) | 0.03(0.01,0.05) |
| Tunisia | female | 3779.58(3034.54,4766.71) | 223.43(216.27,230.78) | 5269.17(4168.71,6674.75) | 235.63(229.23,242.18) | 1.15(0.90,1.41) | 0.25(0.21,0.28) |
| Türkiye | female | 26728.59(21011.33,32835.11) | 230.13(227.36,232.94) | 36143.73(28466.47,45618.80) | 228.93(226.57,231.31) | 0.95(0.84,1.06) | 0.03(0.01,0.05) |
| Turkmenistan | female | 2055.85(1698.28,2512.01) | 266.64(255.13,278.57) | 2956.72(2476.13,3557.89) | 304.80(293.91,316.00) | 1.27(1.22,1.33) | 0.56(0.47,0.65) |
| Tuvalu | female | 5.14(4.13,6.32) | 270.00(89.40,628.39) | 6.38(5.24,7.90) | 275.10(104.36,599.97) | 0.77(0.56,0.99) | 0.03(0.01,0.05) |
| Uganda | female | 4935.95(3966.36,6229.20) | 149.85(145.52,154.29) | 12409.50(9990.19,15391.50) | 142.54(139.98,145.15) | 3.07(3.03,3.10) | -0.14(-0.19,-0.09) |
| Ukraine | female | 27489.18(22206.32,34025.92) | 286.35(282.95,289.79) | 20730.69(16547.05,25986.40) | 302.03(297.72,306.39) | -0.80(-0.86,-0.73) | 0.18(0.11,0.26) |
| United Arab Emirates | female | 765.52(603.75,987.92) | 256.61(238.58,275.71) | 3128.66(2428.21,4002.51) | 269.54(258.98,280.47) | 6.00(5.16,6.84) | 0.23(0.18,0.27) |
| United Kingdom | female | 53393.17(43605.05,65074.18) | 528.10(523.61,532.63) | 65247.24(54303.16,78175.67) | 603.71(599.02,608.42) | 0.68(0.61,0.74) | 0.42(0.32,0.53) |
| United Republic of Tanzania | female | 8395.77(6791.81,10493.73) | 162.27(158.71,165.90) | 18537.12(14768.36,23413.64) | 152.12(149.90,154.37) | 2.46(2.41,2.52) | -0.28(-0.31,-0.24) |
| United States of America | female | 296304.43(248575.78,357956.28) | 612.22(609.99,614.45) | 528244.35(458155.28,610160.28) | 988.66(985.99,991.34) | 2.06(1.73,2.39) | 1.67(1.35,1.99) |
| United States Virgin Islands | female | 63.03(50.50,79.02) | 299.97(230.34,384.65) | 38.19(30.48,47.69) | 320.39(226.34,442.40) | -1.43(-1.76,-1.09) | 0.29(0.22,0.36) |
| Uruguay | female | 2043.87(1666.72,2515.95) | 356.75(341.45,372.56) | 2090.13(1731.76,2547.22) | 350.13(335.24,365.52) | 0.15(0.08,0.22) | 0.01(-0.02,0.04) |
| Uzbekistan | female | 10973.10(8973.23,13476.76) | 255.72(250.92,260.60) | 17322.58(14035.70,21239.93) | 254.07(250.27,257.91) | 1.67(1.56,1.78) | 0.04(-0.01,0.10) |
| Vanuatu | female | 77.14(62.27,95.09) | 259.10(203.97,325.68) | 164.17(133.16,200.54) | 260.29(221.81,303.85) | 2.50(2.47,2.54) | 0.01(-0.02,0.04) |
| Venezuela (Bolivarian Republic of) | female | 8617.19(6957.74,10797.08) | 215.96(211.40,220.60) | 10614.08(8621.95,13275.14) | 212.59(208.53,216.72) | 1.26(0.94,1.59) | 0.01(-0.01,0.03) |
| Viet Nam | female | 34939.52(28375.90,43192.77) | 236.74(234.24,239.26) | 46465.52(36425.83,58539.85) | 247.63(245.35,249.93) | 1.04(0.89,1.18) | 0.17(0.11,0.23) |
| Yemen | female | 4663.27(3752.26,5766.77) | 203.67(197.81,209.66) | 13907.18(11339.72,17196.06) | 204.84(201.43,208.29) | 3.86(3.74,3.98) | 0.12(0.08,0.15) |
| Zambia | female | 2357.40(1912.20,2893.77) | 151.67(145.29,158.29) | 5419.74(4354.92,6828.33) | 134.22(130.61,137.91) | 2.96(2.75,3.17) | -0.28(-0.36,-0.21) |
| Zimbabwe | female | 3796.85(3050.16,4708.82) | 188.59(182.47,194.89) | 5765.05(4665.46,7248.73) | 175.79(171.24,180.42) | 1.11(1.01,1.21) | -0.31(-0.38,-0.24) |
| Afghanistan | male | 3178.83(2599.59,3948.53) | 224.28(214.95,234.01) | 14411.84(11970.02,17145.55) | 233.21(229.23,237.25) | 4.86(4.45,5.26) | 0.33(0.23,0.42) |
| Albania | male | 2336.26(1873.77,2835.26) | 322.50(309.52,335.92) | 1932.33(1625.97,2338.70) | 390.22(372.91,408.16) | -0.46(-0.57,-0.35) | 0.69(0.66,0.72) |
| Algeria | male | 12364.93(10207.89,15218.18) | 244.41(240.01,248.88) | 22838.09(18878.82,27119.60) | 265.28(261.82,268.77) | 1.98(1.71,2.25) | 0.26(0.24,0.28) |
| American Samoa | male | 38.82(30.80,48.10) | 374.47(265.14,517.19) | 34.85(27.35,43.69) | 373.01(257.93,525.24) | -0.65(-0.88,-0.42) | -0.02(-0.03,-0.01) |
| Andorra | male | 74.95(58.89,93.32) | 571.55(446.62,723.92) | 76.42(62.38,92.11) | 590.45(463.13,744.38) | -0.29(-0.59,0.01) | 0.14(0.11,0.17) |
| Angola | male | 4465.82(3563.63,5653.75) | 228.30(221.57,235.20) | 13810.45(11113.08,17220.03) | 241.18(237.08,245.33) | 3.78(3.72,3.83) | 0.26(0.23,0.30) |
| Antigua and Barbuda | male | 51.31(40.78,64.57) | 408.59(304.15,539.35) | 68.06(54.01,85.26) | 407.91(316.55,518.20) | 1.08(0.91,1.25) | 0.08(0.04,0.12) |
| Argentina | male | 20786.51(17535.22,25340.71) | 339.11(334.51,343.76) | 29232.83(24509.49,35142.08) | 339.36(335.47,343.27) | 1.15(1.08,1.23) | 0.00(-0.06,0.05) |
| Armenia | male | 2358.09(1957.61,2840.93) | 332.86(319.51,346.64) | 1626.67(1357.03,1952.01) | 310.91(295.65,326.79) | -1.06(-1.15,-0.97) | -0.24(-0.27,-0.21) |
| Australia | male | 39311.96(33088.85,46254.03) | 1177.48(1165.86,1189.19) | 38570.00(33001.34,44801.91) | 913.56(904.37,922.81) | -0.05(-0.14,0.04) | -0.84(-0.94,-0.74) |
| Austria | male | 8214.39(6863.09,9806.84) | 547.15(535.26,559.24) | 8419.39(6965.87,10076.67) | 597.46(584.56,610.59) | -0.22(-0.40,-0.03) | 0.01(-0.25,0.27) |
| Azerbaijan | male | 4974.95(4092.31,6023.18) | 316.03(307.24,325.03) | 6390.00(5247.04,7779.46) | 301.78(294.29,309.42) | 1.08(0.96,1.20) | -0.12(-0.18,-0.06) |
| Bahamas | male | 264.33(212.47,324.57) | 452.30(399.07,511.20) | 346.55(276.43,425.64) | 456.15(409.38,506.83) | 0.89(0.81,0.97) | 0.09(0.03,0.14) |
| Bahrain | male | 381.15(302.16,473.37) | 241.09(216.05,268.80) | 1094.58(875.58,1368.03) | 234.31(220.03,249.42) | 4.75(4.11,5.40) | 0.00(-0.06,0.06) |
| Bangladesh | male | 45819.14(36696.18,56590.03) | 220.15(218.12,222.21) | 70965.16(57161.22,86615.74) | 216.50(214.91,218.10) | 1.48(1.39,1.57) | 0.08(0.04,0.12) |
| Barbados | male | 229.80(181.73,288.18) | 431.40(377.39,491.05) | 203.66(164.91,249.53) | 426.58(369.88,489.66) | -0.32(-0.42,-0.23) | -0.04(-0.08,0.00) |
| Belarus | male | 8823.15(7376.38,10553.51) | 456.53(446.97,466.24) | 6559.50(5464.84,7952.08) | 454.25(442.89,465.85) | -0.85(-1.24,-0.46) | 0.05(-0.19,0.29) |
| Belgium | male | 10409.67(8385.90,12663.76) | 565.92(555.02,577.00) | 9851.96(8035.14,11746.78) | 569.76(558.48,581.23) | -0.17(-0.26,-0.08) | -0.04(-0.09,0.02) |
| Belize | male | 163.20(131.79,204.44) | 407.59(346.01,478.48) | 379.55(303.63,471.30) | 395.27(356.31,437.55) | 2.88(2.80,2.96) | -0.08(-0.13,-0.03) |
| Benin | male | 1316.96(1063.76,1647.54) | 172.45(163.11,182.23) | 4604.99(3742.72,5653.24) | 184.49(179.08,190.03) | 4.17(4.13,4.20) | 0.26(0.23,0.29) |
| Bermuda | male | 51.10(40.16,62.91) | 419.92(310.65,558.22) | 36.85(29.18,46.07) | 431.60(301.16,602.03) | -1.08(-1.17,-0.99) | 0.08(0.04,0.12) |
| Bhutan | male | 307.07(244.00,401.04) | 209.33(185.69,235.53) | 402.11(323.99,495.80) | 220.45(199.36,243.27) | 1.38(1.17,1.59) | 0.18(0.16,0.20) |
| Bolivia (Plurinational State of) | male | 3436.82(2870.35,4222.41) | 275.93(266.66,285.47) | 6800.10(5698.49,8170.01) | 275.62(269.10,282.26) | 2.31(2.13,2.49) | -0.04(-0.06,-0.02) |
| Bosnia and Herzegovina | male | 2663.30(2143.73,3309.85) | 271.71(261.48,282.25) | 1399.03(1130.87,1732.52) | 274.35(260.00,289.34) | -1.36(-1.73,-0.98) | 0.22(0.10,0.34) |
| Botswana | male | 557.70(452.99,697.23) | 230.07(210.79,250.83) | 1256.10(1021.85,1505.79) | 237.05(224.10,250.58) | 2.62(2.46,2.79) | 0.10(0.07,0.13) |
| Brazil | male | 118705.93(96457.49,147013.08) | 371.27(369.15,373.40) | 157600.18(132511.91,186831.75) | 382.30(380.40,384.20) | 1.08(0.89,1.26) | 0.24(0.17,0.32) |
| Brunei Darussalam | male | 284.24(229.86,346.93) | 438.11(388.45,492.57) | 435.26(353.49,534.37) | 407.46(369.58,448.42) | 1.50(1.44,1.56) | -0.28(-0.33,-0.23) |
| Bulgaria | male | 5386.65(4543.24,6412.29) | 362.13(352.51,371.96) | 4083.88(3449.51,4890.93) | 447.17(433.20,461.49) | -1.03(-1.25,-0.81) | 0.59(0.55,0.63) |
| Burkina Faso | male | 2279.17(1839.05,2791.17) | 161.11(154.34,168.13) | 6925.97(5573.79,8451.41) | 178.34(174.10,182.67) | 3.83(3.77,3.89) | 0.41(0.38,0.45) |
| Burundi | male | 2013.25(1602.00,2517.62) | 199.22(190.50,208.27) | 5164.53(4145.62,6402.18) | 194.96(189.63,200.40) | 3.43(3.16,3.69) | -0.06(-0.08,-0.04) |
| Cabo Verde | male | 105.24(85.17,129.22) | 178.35(143.55,220.75) | 289.79(222.85,358.18) | 218.81(194.26,245.74) | 3.51(3.26,3.77) | 0.80(0.71,0.90) |
| Cambodia | male | 5082.69(4112.81,6096.59) | 279.02(271.18,287.04) | 9991.29(8147.49,12156.41) | 274.78(269.41,280.22) | 2.30(2.15,2.44) | -0.03(-0.06,0.00) |
| Cameroon | male | 3002.40(2439.71,3671.81) | 170.18(164.03,176.52) | 11550.97(9395.41,14034.64) | 186.80(183.36,190.28) | 4.57(4.50,4.65) | 0.36(0.33,0.39) |
| Canada | male | 38831.39(32835.66,44564.35) | 753.98(746.41,761.60) | 47519.91(41271.25,54417.30) | 845.90(838.24,853.61) | 0.81(0.67,0.96) | 0.55(0.44,0.67) |
| Central African Republic | male | 1078.80(868.06,1367.39) | 214.34(201.49,227.87) | 2254.37(1808.77,2860.94) | 215.97(206.96,225.30) | 2.36(2.16,2.57) | 0.03(0.02,0.05) |
| Chad | male | 1576.76(1279.08,1949.19) | 162.80(154.66,171.28) | 4996.92(4072.55,6112.22) | 171.84(166.90,176.90) | 3.83(3.73,3.92) | 0.25(0.21,0.29) |
| Chile | male | 11162.28(9362.55,13334.80) | 390.48(383.25,397.82) | 15740.89(13023.66,18902.90) | 462.25(454.99,469.60) | 1.04(0.93,1.14) | 0.44(0.37,0.51) |
| China | male | 1234690.74(1025722.80,1463925.63) | 430.35(429.59,431.11) | 806346.58(668234.94,975026.23) | 342.46(341.70,343.23) | -1.82(-2.03,-1.61) | -1.25(-1.45,-1.05) |
| Colombia | male | 19773.66(16567.03,23687.86) | 286.22(282.21,290.27) | 35879.19(29547.24,43591.12) | 363.78(360.02,367.58) | 1.60(1.34,1.86) | 0.50(0.32,0.67) |
| Comoros | male | 174.13(136.90,221.38) | 202.96(172.73,237.58) | 329.88(264.20,407.60) | 212.51(190.12,236.90) | 2.07(1.99,2.14) | 0.18(0.16,0.20) |
| Congo | male | 1098.01(865.51,1388.12) | 240.80(226.32,256.04) | 2616.66(2069.46,3229.60) | 243.83(234.53,253.41) | 2.77(2.65,2.90) | 0.05(0.02,0.08) |
| Cook Islands | male | 15.41(12.05,19.27) | 376.40(210.91,631.46) | 10.92(8.58,13.50) | 392.33(194.83,710.93) | -1.28(-1.47,-1.09) | 0.13(0.11,0.15) |
| Costa Rica | male | 1853.32(1507.40,2314.55) | 290.77(277.61,304.42) | 2725.36(2198.74,3325.66) | 298.64(287.49,310.13) | 1.22(0.95,1.49) | 0.14(0.08,0.19) |
| Croatia | male | 4023.25(3336.34,4870.83) | 447.09(433.29,461.23) | 3193.53(2656.50,3828.17) | 516.40(498.36,534.96) | -0.75(-0.90,-0.60) | 0.40(0.24,0.57) |
| Cuba | male | 9244.70(7330.14,11852.74) | 369.46(361.88,377.17) | 6380.42(5092.35,7979.65) | 361.91(352.98,371.02) | -1.23(-1.33,-1.13) | -0.09(-0.11,-0.06) |
| Cyprus | male | 744.09(597.66,925.52) | 472.38(438.99,507.69) | 1154.14(922.09,1454.01) | 458.83(431.51,487.64) | 1.77(1.68,1.85) | -0.03(-0.09,0.04) |
| Czechia | male | 8870.88(7342.88,10564.47) | 468.79(459.05,478.70) | 7471.26(6120.75,8916.37) | 532.93(520.63,545.46) | -0.38(-0.75,0.01) | 0.71(0.48,0.95) |
| C?te d'Ivoire | male | 3908.27(3167.07,4830.49) | 166.76(161.50,172.15) | 10003.39(8057.63,12209.10) | 178.65(175.15,182.21) | 3.00(2.83,3.18) | 0.26(0.24,0.29) |
| Democratic People's Republic of Korea | male | 13328.61(10904.17,16186.30) | 339.68(333.92,345.52) | 17437.18(14223.03,20981.23) | 335.33(330.33,340.40) | 0.99(0.91,1.06) | -0.02(-0.05,0.01) |
| Democratic Republic of the Congo | male | 15542.42(12552.12,19786.66) | 219.84(216.31,223.41) | 40407.56(32243.25,50743.30) | 221.36(219.17,223.56) | 3.14(3.08,3.19) | 0.03(-0.02,0.08) |
| Denmark | male | 6484.14(5463.58,7723.63) | 669.26(653.02,685.81) | 6109.76(4954.91,7310.01) | 658.86(642.37,675.69) | -0.27(-0.43,-0.11) | -0.03(-0.08,0.01) |
| Djibouti | male | 190.25(150.96,240.79) | 202.83(174.24,235.26) | 626.04(508.73,766.25) | 216.30(199.68,233.95) | 3.92(3.76,4.09) | 0.25(0.23,0.26) |
| Dominica | male | 76.67(58.78,98.38) | 464.12(364.57,585.48) | 58.78(46.60,73.21) | 439.97(334.73,568.00) | -0.86(-0.95,-0.77) | -0.25(-0.36,-0.13) |
| Dominican Republic | male | 4470.23(3552.47,5706.59) | 297.38(288.56,306.42) | 7282.71(5882.72,9130.06) | 319.86(312.55,327.30) | 1.61(1.58,1.64) | 0.18(0.09,0.26) |
| Ecuador | male | 5595.28(4627.93,6818.37) | 268.88(261.79,276.14) | 9982.75(8097.50,12339.94) | 273.05(267.71,278.47) | 1.89(1.86,1.92) | 0.08(0.07,0.09) |
| Egypt | male | 22439.40(18530.96,27347.70) | 202.28(199.61,204.98) | 48070.85(40099.33,59042.33) | 222.05(220.07,224.04) | 2.56(2.53,2.59) | 0.31(0.24,0.38) |
| El Salvador | male | 2173.86(1795.00,2644.03) | 218.51(209.17,228.20) | 2896.40(2380.18,3583.43) | 235.81(227.22,244.66) | 0.67(0.58,0.75) | -0.02(-0.08,0.05) |
| Equatorial Guinea | male | 157.89(125.97,201.54) | 231.56(195.84,272.41) | 978.08(771.35,1229.08) | 255.25(239.06,272.38) | 6.49(6.35,6.64) | 0.47(0.38,0.56) |
| Eritrea | male | 1349.38(1065.81,1712.19) | 209.21(197.78,221.19) | 3275.14(2581.33,4030.92) | 227.32(219.54,235.31) | 3.37(3.17,3.57) | 0.29(0.26,0.32) |
| Estonia | male | 1637.84(1399.04,1958.11) | 584.04(556.01,613.14) | 2089.24(1814.57,2391.86) | 1113.52(1064.94,1163.85) | 0.79(0.38,1.21) | 1.92(1.61,2.22) |
| Eswatini | male | 350.35(288.03,434.54) | 249.86(223.15,279.25) | 694.53(569.13,832.44) | 277.30(256.92,298.93) | 1.94(1.73,2.15) | 0.25(0.22,0.28) |
| Ethiopia | male | 17892.08(14236.10,22703.73) | 200.23(197.23,203.27) | 48113.75(38202.96,60876.12) | 205.36(203.48,207.26) | 3.09(3.01,3.17) | 0.10(0.06,0.14) |
| Fiji | male | 560.87(444.67,704.19) | 336.42(309.03,365.73) | 612.74(495.07,762.93) | 336.43(310.31,364.18) | 0.22(0.12,0.33) | -0.01(-0.03,0.00) |
| Finland | male | 5350.56(4346.19,6560.07) | 581.70(566.00,597.76) | 6145.13(5147.93,7384.37) | 733.66(715.26,752.44) | 0.37(0.26,0.48) | 0.56(0.42,0.71) |
| France | male | 58404.35(48478.92,69543.54) | 535.66(531.32,540.03) | 55620.70(45237.01,68711.47) | 561.48(556.81,566.17) | -0.04(-0.09,0.01) | 0.25(0.19,0.31) |
| Gabon | male | 487.76(388.02,605.07) | 254.82(232.55,278.75) | 926.82(736.85,1152.56) | 267.46(250.42,285.41) | 2.07(2.05,2.09) | 0.15(0.14,0.16) |
| Gambia | male | 295.55(239.53,363.91) | 166.52(147.68,187.32) | 817.89(669.89,991.84) | 174.12(162.15,186.79) | 3.19(3.14,3.24) | 0.19(0.16,0.22) |
| Georgia | male | 3100.53(2573.22,3741.25) | 296.85(286.48,307.51) | 1695.29(1399.91,2031.30) | 293.83(279.83,308.38) | -1.76(-1.92,-1.60) | 0.15(0.00,0.30) |
| Germany | male | 83962.29(69032.00,103819.09) | 569.44(565.50,573.41) | 80934.90(67664.73,99718.80) | 622.94(618.57,627.34) | -0.44(-0.65,-0.24) | 0.21(0.16,0.27) |
| Ghana | male | 4601.93(3716.34,5677.94) | 168.37(163.46,173.40) | 11770.99(9533.04,14190.43) | 172.36(169.24,175.53) | 3.19(3.13,3.24) | 0.10(0.07,0.13) |
| Greece | male | 7728.22(6270.06,9547.21) | 409.45(400.37,418.68) | 6076.99(4962.05,7533.88) | 442.97(431.77,454.40) | -1.52(-2.05,-0.98) | -0.30(-0.56,-0.05) |
| Greenland | male | 78.96(65.06,95.10) | 612.07(479.07,774.19) | 58.22(47.84,70.77) | 591.64(447.85,769.18) | -0.75(-0.86,-0.63) | -0.06(-0.12,0.00) |
| Grenada | male | 70.92(56.23,88.62) | 402.66(313.40,511.80) | 83.76(66.55,103.05) | 403.81(321.62,501.45) | 0.52(0.22,0.82) | 0.12(0.09,0.16) |
| Guam | male | 128.37(102.92,160.01) | 374.93(312.45,446.66) | 120.29(94.23,147.07) | 417.05(345.80,498.87) | -0.14(-0.21,-0.08) | 0.38(0.36,0.40) |
| Guatemala | male | 4720.55(3665.27,5777.56) | 356.07(345.82,366.56) | 9033.52(7088.31,11506.79) | 283.50(277.60,289.49) | 2.34(1.99,2.70) | -0.57(-0.88,-0.26) |
| Guinea | male | 1504.68(1212.44,1852.45) | 162.58(154.38,171.14) | 4032.18(3266.59,4955.65) | 174.37(168.91,179.98) | 3.01(2.89,3.12) | 0.28(0.25,0.31) |
| Guinea-Bissau | male | 277.27(224.59,341.47) | 162.42(143.35,183.54) | 685.12(560.01,832.73) | 173.79(160.81,187.61) | 3.03(3.00,3.05) | 0.28(0.25,0.30) |
| Guyana | male | 558.72(453.89,686.62) | 323.30(296.71,351.84) | 505.23(402.22,640.31) | 329.33(300.99,359.78) | -0.33(-0.45,-0.21) | 0.01(-0.03,0.06) |
| Haiti | male | 3890.09(3133.61,4900.66) | 326.09(315.81,336.63) | 9475.73(7458.88,11887.88) | 349.27(342.27,356.39) | 2.86(2.74,2.98) | 0.10(0.07,0.14) |
| Honduras | male | 2526.94(2009.23,3177.06) | 297.89(286.11,310.06) | 6631.82(5277.28,8329.88) | 313.98(306.37,321.74) | 3.17(3.09,3.25) | 0.16(0.15,0.17) |
| Hungary | male | 6923.92(5766.27,8251.94) | 377.62(368.71,386.71) | 5442.45(4508.21,6516.96) | 403.06(392.31,414.05) | -0.71(-0.89,-0.53) | 0.33(0.25,0.42) |
| Iceland | male | 297.33(241.28,361.98) | 563.99(501.64,632.10) | 400.52(329.31,489.32) | 657.24(593.88,725.90) | 1.12(1.04,1.21) | 0.52(0.45,0.59) |
| India | male | 427320.50(335477.75,533151.72) | 239.83(239.11,240.56) | 818681.95(661668.27,1006162.36) | 260.17(259.61,260.74) | 2.39(2.27,2.51) | 0.44(0.32,0.57) |
| Indonesia | male | 86916.74(70114.22,105416.05) | 224.08(222.58,225.58) | 142593.39(116139.30,172551.27) | 246.34(245.07,247.63) | 1.37(1.23,1.51) | 0.16(0.03,0.30) |
| Iran (Islamic Republic of) | male | 38729.55(33133.60,45664.84) | 354.07(350.48,357.70) | 81130.89(69195.66,94157.04) | 480.97(477.56,484.40) | 2.41(1.72,3.11) | 0.89(0.63,1.15) |
| Iraq | male | 8318.35(6925.50,10090.51) | 222.62(217.73,227.60) | 21129.49(17694.01,25508.05) | 232.61(229.47,235.78) | 2.98(2.74,3.21) | 0.13(0.11,0.16) |
| Ireland | male | 4305.66(3588.69,5204.22) | 600.71(582.80,619.05) | 5443.07(4543.95,6402.78) | 706.15(687.42,725.27) | 0.74(0.53,0.95) | 0.35(0.25,0.45) |
| Israel | male | 5024.35(4032.58,6010.35) | 523.38(508.94,538.13) | 8157.32(6458.67,10046.01) | 483.16(472.73,493.77) | 1.73(1.62,1.84) | -0.11(-0.17,-0.04) |
| Italy | male | 73647.33(60975.06,89166.43) | 686.50(681.55,691.49) | 43230.67(35346.69,52226.35) | 543.32(538.16,548.51) | -1.73(-1.85,-1.62) | -0.81(-0.91,-0.72) |
| Jamaica | male | 1950.39(1558.24,2473.69) | 385.62(368.39,403.55) | 2260.90(1840.77,2750.87) | 392.73(376.65,409.32) | 0.49(0.37,0.62) | -0.06(-0.12,-0.01) |
| Japan | male | 95370.59(76708.50,119207.73) | 417.92(415.26,420.59) | 63263.27(50900.10,76990.71) | 394.21(391.11,397.32) | -1.44(-1.51,-1.38) | -0.19(-0.22,-0.16) |
| Jordan | male | 1828.95(1503.67,2220.24) | 223.64(212.92,234.84) | 6460.79(5286.49,7970.44) | 221.19(215.82,226.66) | 4.26(3.98,4.55) | 0.01(-0.01,0.04) |
| Kazakhstan | male | 14457.96(11970.91,17293.93) | 423.53(416.64,430.51) | 18787.53(15461.35,22351.27) | 536.95(529.21,544.78) | 0.94(0.86,1.01) | 0.57(0.40,0.73) |
| Kenya | male | 6557.71(5261.89,8133.33) | 155.16(151.29,159.11) | 19460.03(15618.81,23976.71) | 182.62(180.02,185.25) | 3.76(3.63,3.89) | 0.72(0.59,0.85) |
| Kiribati | male | 52.42(41.21,65.37) | 339.94(253.39,449.31) | 88.76(71.86,107.77) | 359.01(288.11,442.71) | 1.93(1.79,2.07) | 0.17(0.16,0.19) |
| Kuwait | male | 1302.28(1031.30,1633.07) | 263.33(248.65,278.78) | 3141.87(2461.08,4000.37) | 291.91(280.96,303.25) | 3.64(3.36,3.93) | 0.46(0.39,0.53) |
| Kyrgyzstan | male | 3006.92(2497.72,3605.49) | 332.30(320.46,344.50) | 4499.88(3707.08,5398.99) | 330.55(320.94,340.38) | 1.34(1.20,1.49) | -0.05(-0.11,0.02) |
| Lao People's Democratic Republic | male | 1997.61(1599.04,2452.22) | 265.17(253.50,277.28) | 4309.37(3481.94,5258.97) | 266.84(258.93,274.94) | 2.57(2.40,2.74) | 0.02(0.00,0.04) |
| Latvia | male | 2354.40(1982.91,2785.85) | 500.15(480.10,520.85) | 1455.60(1233.66,1720.73) | 561.82(532.52,592.41) | -1.79(-2.00,-1.58) | 0.10(-0.01,0.20) |
| Lebanon | male | 1393.80(1146.20,1677.16) | 255.37(241.89,269.45) | 3836.28(3206.23,4619.76) | 324.00(313.62,334.66) | 3.77(3.44,4.11) | 0.82(0.78,0.86) |
| Lesotho | male | 583.51(479.15,729.75) | 240.49(220.86,261.51) | 1054.95(860.23,1282.26) | 252.69(237.56,268.57) | 1.85(1.74,1.97) | 0.16(0.14,0.18) |
| Liberia | male | 778.27(630.92,958.16) | 175.71(163.50,188.62) | 2100.76(1709.78,2593.09) | 187.67(179.63,195.99) | 4.63(4.23,5.03) | 0.19(0.17,0.21) |
| Libya | male | 2270.47(1834.31,2751.71) | 259.40(248.66,270.51) | 4937.40(4023.69,6041.26) | 315.86(307.05,324.87) | 2.98(2.57,3.38) | 0.74(0.67,0.80) |
| Lithuania | male | 3042.88(2581.41,3564.87) | 435.74(420.36,451.55) | 2933.16(2504.82,3440.59) | 717.19(691.03,744.16) | -0.41(-0.64,-0.18) | 1.42(1.11,1.74) |
| Luxembourg | male | 501.02(408.05,614.85) | 691.19(630.67,756.48) | 751.44(599.26,963.03) | 691.80(642.02,744.72) | 1.16(0.95,1.38) | -0.18(-0.28,-0.07) |
| Madagascar | male | 4566.99(3603.10,5768.41) | 201.38(195.45,207.46) | 11820.20(9334.05,14924.36) | 204.95(201.20,208.76) | 3.11(3.07,3.15) | 0.08(0.06,0.10) |
| Malawi | male | 3658.88(2906.69,4627.82) | 198.03(191.49,204.76) | 8231.46(6531.24,10429.89) | 204.74(200.18,209.39) | 2.60(2.36,2.84) | 0.14(0.12,0.16) |
| Malaysia | male | 12233.25(9793.42,15005.92) | 329.01(323.19,334.92) | 22318.54(18018.17,27119.58) | 307.53(303.49,311.62) | 2.04(1.84,2.23) | -0.19(-0.23,-0.15) |
| Maldives | male | 128.68(102.79,155.94) | 308.56(255.80,370.71) | 519.05(410.65,640.22) | 319.93(289.26,353.59) | 4.94(4.71,5.17) | 0.14(0.13,0.15) |
| Mali | male | 2228.08(1813.49,2738.16) | 162.15(155.36,169.17) | 6884.33(5596.32,8498.36) | 165.32(161.27,169.46) | 3.78(3.66,3.89) | 0.11(0.07,0.14) |
| Malta | male | 312.57(253.47,386.88) | 447.88(399.30,501.00) | 397.13(322.51,481.74) | 591.52(533.02,655.32) | 0.91(0.78,1.04) | 0.84(0.77,0.91) |
| Marshall Islands | male | 33.01(25.74,41.81) | 359.90(246.08,512.37) | 45.27(36.01,55.13) | 366.34(267.40,490.63) | 0.81(0.61,1.01) | 0.04(0.02,0.06) |
| Mauritania | male | 616.37(498.38,760.52) | 168.03(154.82,182.14) | 1402.70(1152.15,1697.17) | 175.41(166.17,185.06) | 2.69(2.64,2.75) | 0.21(0.18,0.25) |
| Mauritius | male | 917.73(747.55,1108.32) | 364.90(341.63,389.38) | 1176.61(948.41,1497.34) | 513.58(484.59,543.90) | 0.94(0.72,1.15) | 1.29(1.13,1.46) |
| Mexico | male | 42320.36(35379.52,50895.06) | 241.21(238.87,243.58) | 60827.99(50535.56,71734.54) | 240.45(238.54,242.37) | 1.36(1.25,1.46) | 0.22(0.12,0.32) |
| Micronesia (Federated States of) | male | 76.28(60.35,96.44) | 356.70(279.98,449.31) | 80.91(63.55,100.04) | 357.11(283.03,445.80) | 0.01(-0.10,0.11) | -0.01(-0.03,0.00) |
| Monaco | male | 26.45(20.94,33.07) | 593.74(384.88,888.55) | 28.81(23.09,35.19) | 618.84(413.08,894.19) | 0.19(0.09,0.28) | 0.18(0.14,0.22) |
| Mongolia | male | 1488.13(1240.85,1781.09) | 325.63(308.94,343.13) | 2081.76(1732.97,2497.28) | 338.89(324.34,353.96) | 1.11(0.89,1.34) | 0.18(0.16,0.21) |
| Montenegro | male | 475.36(382.85,578.01) | 367.15(334.87,401.74) | 373.26(302.57,459.92) | 355.41(320.10,393.67) | -0.70(-0.76,-0.64) | -0.06(-0.10,-0.01) |
| Morocco | male | 12073.85(9951.69,14764.01) | 235.44(231.21,239.74) | 19046.98(15927.49,22839.99) | 257.89(254.24,261.58) | 1.45(1.36,1.53) | 0.29(0.27,0.30) |
| Mozambique | male | 4393.48(3479.47,5532.62) | 200.08(194.08,206.23) | 13572.92(10687.20,17040.25) | 245.35(241.10,249.66) | 3.73(3.62,3.83) | 0.75(0.71,0.79) |
| Myanmar | male | 27570.87(21772.38,33671.27) | 318.43(314.65,322.26) | 34517.24(27588.12,42241.36) | 310.37(307.10,313.67) | 0.66(0.56,0.76) | -0.04(-0.09,0.01) |
| Namibia | male | 691.78(566.07,860.08) | 250.51(231.60,270.72) | 1368.30(1122.99,1661.20) | 266.35(252.36,280.95) | 2.17(1.96,2.37) | 0.22(0.20,0.24) |
| Nauru | male | 7.50(5.96,9.28) | 361.19(150.16,743.20) | 8.85(7.03,10.91) | 365.41(164.91,713.38) | 0.37(0.30,0.44) | 0.03(-0.03,0.08) |
| Nepal | male | 7359.19(5856.61,9398.16) | 207.85(203.08,212.71) | 13082.05(10410.70,16599.31) | 213.08(209.39,216.83) | 1.76(1.66,1.86) | 0.05(0.02,0.08) |
| Netherlands | male | 16542.38(13654.00,19789.69) | 560.01(551.46,568.67) | 13793.56(11249.85,16719.42) | 523.29(514.56,532.13) | -0.60(-0.73,-0.48) | -0.20(-0.27,-0.13) |
| New Zealand | male | 5976.26(4743.72,7479.11) | 869.13(847.23,891.46) | 8484.67(6727.43,10575.41) | 957.05(936.65,977.81) | 0.63(0.44,0.83) | -0.15(-0.32,0.02) |
| Nicaragua | male | 1840.12(1521.65,2283.54) | 259.21(247.15,271.76) | 3247.47(2646.86,4004.90) | 228.81(221.00,236.84) | 1.93(1.80,2.06) | -0.31(-0.35,-0.27) |
| Niger | male | 2129.89(1736.51,2615.64) | 163.27(156.28,170.52) | 7039.14(5785.63,8688.71) | 165.15(161.15,169.24) | 3.73(3.62,3.84) | 0.07(0.04,0.11) |
| Nigeria | male | 24108.08(19459.79,29117.60) | 146.00(144.15,147.87) | 68232.64(54936.61,82772.46) | 168.75(167.45,170.05) | 3.41(3.34,3.48) | 0.53(0.46,0.59) |
| Niue | male | 1.64(1.30,2.04) | 370.04(29.85,1651.16) | 1.15(0.92,1.41) | 383.70(14.12,2098.74) | -1.44(-1.72,-1.17) | 0.14(0.12,0.15) |
| North Macedonia | male | 1317.19(1078.30,1587.19) | 325.48(308.14,343.56) | 1351.41(1104.68,1647.03) | 348.54(329.79,368.17) | 0.56(0.33,0.79) | 0.47(0.34,0.60) |
| Northern Mariana Islands | male | 42.24(34.27,51.60) | 381.36(273.31,521.12) | 35.38(28.71,42.82) | 409.92(285.88,571.88) | -0.95(-1.39,-0.51) | 0.25(0.19,0.31) |
| Norway | male | 4816.36(3986.34,5911.32) | 588.83(572.29,605.75) | 6125.19(5141.55,7231.56) | 682.41(665.30,699.87) | 0.66(0.57,0.75) | 0.25(0.05,0.45) |
| Oman | male | 1188.69(952.83,1466.79) | 219.29(206.75,232.45) | 3338.13(2642.95,4209.19) | 218.63(210.16,227.42) | 3.74(3.36,4.12) | 0.03(0.01,0.05) |
| Pakistan | male | 44550.97(34754.56,56123.20) | 206.33(204.37,208.30) | 106389.96(84692.77,131130.35) | 212.92(211.63,214.21) | 2.97(2.91,3.04) | 0.16(0.11,0.21) |
| Palau | male | 13.58(10.97,16.84) | 369.06(199.31,630.92) | 12.88(10.25,15.74) | 381.92(200.48,668.87) | -0.51(-0.93,-0.08) | 0.11(0.10,0.12) |
| Palestine | male | 904.41(755.76,1115.41) | 230.76(215.24,247.27) | 2629.91(2127.75,3203.11) | 234.50(225.51,243.79) | 3.43(3.21,3.65) | -0.03(-0.07,0.00) |
| Panama | male | 1663.81(1345.38,2030.56) | 321.83(306.40,337.90) | 2447.98(1965.44,3013.32) | 292.52(281.03,304.36) | 1.17(1.11,1.24) | -0.28(-0.34,-0.21) |
| Papua New Guinea | male | 3038.79(2393.51,3829.75) | 343.37(331.11,356.01) | 7731.04(6128.73,9578.58) | 346.77(339.05,354.63) | 3.20(3.14,3.26) | 0.03(0.02,0.04) |
| Paraguay | male | 2105.68(1730.99,2549.39) | 264.42(253.18,276.06) | 4213.52(3409.27,5139.33) | 270.17(262.07,278.46) | 2.37(2.28,2.45) | 0.07(0.03,0.10) |
| Peru | male | 11565.57(9476.05,14048.12) | 261.51(256.69,266.40) | 19785.32(16101.10,24035.52) | 263.34(259.68,267.04) | 1.65(1.61,1.68) | 0.05(0.03,0.07) |
| Philippines | male | 39853.52(32470.88,48410.17) | 300.36(297.38,303.36) | 66462.46(53995.36,81522.05) | 272.26(270.19,274.34) | 1.58(1.52,1.65) | -0.42(-0.46,-0.38) |
| Poland | male | 29992.37(24762.20,36039.33) | 426.11(421.26,431.01) | 24145.30(19988.99,28680.83) | 429.20(423.62,434.84) | -0.69(-0.87,-0.51) | 0.00(-0.05,0.06) |
| Portugal | male | 9005.55(7505.21,10754.49) | 472.57(462.85,482.44) | 6256.23(5059.67,7587.07) | 430.98(420.30,441.86) | -1.34(-1.45,-1.24) | -0.32(-0.45,-0.20) |
| Puerto Rico | male | 4237.44(3449.31,5228.55) | 614.49(596.06,633.36) | 2682.60(2146.79,3359.15) | 527.40(507.61,547.76) | -1.84(-2.22,-1.46) | -0.85(-1.06,-0.65) |
| Qatar | male | 388.73(308.24,485.56) | 226.51(202.03,253.93) | 2805.60(2192.09,3552.53) | 225.76(214.39,237.85) | 8.96(7.76,10.17) | 0.06(0.03,0.10) |
| Republic of Korea | male | 44923.05(36479.01,55702.34) | 416.52(412.67,420.40) | 33359.57(27046.29,40637.72) | 414.97(410.38,419.60) | -0.86(-0.97,-0.74) | -0.02(-0.06,0.01) |
| Republic of Moldova | male | 3488.03(2921.67,4134.89) | 416.65(402.85,430.83) | 2261.39(1853.85,2704.10) | 378.54(362.35,395.33) | -1.27(-1.50,-1.05) | -0.35(-0.41,-0.29) |
| Romania | male | 11110.37(9006.97,13598.36) | 252.52(247.81,257.30) | 7070.67(5681.75,8838.60) | 252.93(246.99,258.97) | -1.44(-1.57,-1.30) | 0.06(-0.06,0.17) |
| Russian Federation | male | 183261.48(156848.73,213580.93) | 655.25(652.22,658.29) | 156631.46(135750.41,179777.12) | 701.92(698.32,705.53) | -0.98(-1.63,-0.34) | -0.32(-0.78,0.13) |
| Rwanda | male | 2873.81(2280.05,3645.94) | 209.55(201.85,217.48) | 6612.83(5214.85,8321.96) | 230.22(224.65,235.90) | 3.30(2.79,3.81) | 0.35(0.29,0.41) |
| Saint Kitts and Nevis | male | 34.13(27.00,43.28) | 388.67(268.50,548.70) | 43.71(34.61,53.79) | 393.31(284.67,531.42) | 0.94(0.80,1.08) | 0.05(0.03,0.07) |
| Saint Lucia | male | 141.33(107.82,178.16) | 471.85(395.57,560.69) | 136.67(107.24,171.40) | 421.17(353.19,498.82) | 0.30(0.06,0.54) | 0.01(-0.09,0.11) |
| Saint Vincent and the Grenadines | male | 94.05(73.10,120.17) | 386.71(310.69,478.35) | 87.34(68.20,107.75) | 418.00(334.91,515.79) | -0.41(-0.51,-0.31) | 0.20(0.15,0.25) |
| Samoa | male | 148.15(114.12,193.05) | 376.07(315.44,447.06) | 186.69(151.83,229.30) | 422.68(363.66,489.36) | 0.45(0.36,0.54) | 0.18(0.09,0.28) |
| San Marino | male | 26.53(21.23,32.78) | 573.29(376.00,839.56) | 27.01(21.90,33.42) | 609.37(401.45,888.04) | -0.18(-0.29,-0.07) | 0.17(0.12,0.21) |
| Sao Tome and Principe | male | 34.53(27.92,42.62) | 168.09(114.23,242.52) | 81.93(66.56,99.57) | 181.35(143.99,225.76) | 2.90(2.85,2.94) | 0.30(0.25,0.36) |
| Saudi Arabia | male | 8869.11(7338.85,10829.34) | 229.97(225.19,234.83) | 25369.93(20411.85,31636.77) | 230.55(227.58,233.57) | 3.86(3.63,4.08) | 0.10(0.06,0.14) |
| Senegal | male | 2146.96(1745.58,2662.08) | 167.79(160.57,175.27) | 5448.59(4444.18,6705.49) | 173.40(168.72,178.19) | 2.98(2.91,3.05) | 0.15(0.12,0.19) |
| Serbia | male | 6324.69(5072.90,7755.00) | 346.00(337.51,354.66) | 5698.46(4711.19,6905.48) | 369.01(359.39,378.84) | -0.47(-0.61,-0.32) | 0.33(0.26,0.39) |
| Seychelles | male | 65.44(52.74,80.40) | 404.41(311.90,518.15) | 74.37(60.69,89.14) | 368.13(288.04,464.74) | 0.63(0.44,0.83) | -0.13(-0.25,-0.01) |
| Sierra Leone | male | 1225.94(993.14,1500.42) | 167.14(157.84,176.89) | 3071.96(2504.45,3788.80) | 172.80(166.65,179.13) | 3.60(3.24,3.96) | 0.15(0.11,0.19) |
| Singapore | male | 3218.04(2584.91,4009.33) | 422.92(408.36,437.88) | 3756.29(3004.01,4636.42) | 416.35(402.12,431.01) | 0.96(0.58,1.35) | -0.06(-0.09,-0.02) |
| Slovakia | male | 4253.45(3523.67,5126.05) | 416.31(403.85,429.07) | 3168.41(2615.88,3826.15) | 385.72(371.98,399.86) | -1.06(-1.36,-0.76) | -0.38(-0.49,-0.27) |
| Slovenia | male | 1667.86(1362.61,2027.47) | 444.72(423.49,466.77) | 1506.42(1276.83,1793.07) | 543.95(516.10,572.99) | -0.21(-0.48,0.06) | 0.79(0.63,0.94) |
| Solomon Islands | male | 227.49(177.38,295.84) | 321.50(279.63,368.72) | 470.85(377.29,580.53) | 325.62(296.55,356.95) | 2.17(2.07,2.28) | 0.02(0.00,0.04) |
| Somalia | male | 2989.91(2351.51,3838.29) | 191.52(184.57,198.69) | 8394.42(6584.63,10621.84) | 188.71(184.56,192.95) | 3.74(3.61,3.88) | -0.03(-0.06,0.00) |
| South Africa | male | 26131.13(22139.03,31095.72) | 333.21(329.15,337.32) | 41322.19(34101.20,49646.57) | 343.76(340.44,347.11) | 1.23(0.98,1.48) | -0.20(-0.31,-0.09) |
| South Sudan | male | 2472.76(1962.52,3144.16) | 202.88(194.69,211.38) | 3667.10(2864.46,4751.17) | 202.46(195.52,209.61) | 1.42(1.09,1.77) | 0.05(0.03,0.07) |
| Spain | male | 56101.26(48510.76,63935.03) | 740.52(734.39,746.68) | 42541.04(35217.32,50019.69) | 701.43(694.71,708.21) | -0.99(-1.35,-0.62) | -0.29(-0.49,-0.10) |
| Sri Lanka | male | 13003.10(10561.11,15588.12) | 350.40(344.39,356.49) | 12850.69(10506.39,15540.04) | 324.83(319.23,330.50) | -0.22(-0.31,-0.13) | -0.33(-0.38,-0.28) |
| Sudan | male | 7974.52(6576.06,9678.60) | 218.40(213.50,223.39) | 21741.00(18271.70,25673.55) | 237.15(233.95,240.38) | 3.19(3.12,3.26) | 0.33(0.30,0.36) |
| Suriname | male | 311.42(247.77,391.09) | 367.17(326.62,411.99) | 406.50(318.34,497.82) | 379.58(343.55,418.44) | 1.06(0.84,1.27) | 0.19(0.16,0.22) |
| Sweden | male | 7370.35(5936.01,9264.11) | 489.73(478.58,501.08) | 11777.56(9644.03,14326.59) | 715.31(702.31,728.51) | 1.75(1.62,1.88) | 1.43(1.36,1.51) |
| Switzerland | male | 12011.95(10140.88,14241.56) | 943.54(926.43,960.90) | 9638.18(7807.42,11933.91) | 705.00(690.63,719.63) | -0.77(-0.95,-0.59) | -1.10(-1.22,-0.99) |
| Syrian Arab Republic | male | 4722.99(3903.13,5836.76) | 198.18(192.37,204.13) | 4663.52(3851.60,5628.95) | 206.83(199.92,213.98) | 0.08(-1.05,1.23) | 0.16(0.12,0.21) |
| Taiwan (Province of China) | male | 18533.62(14642.47,22427.08) | 392.30(386.66,398.01) | 18108.65(14430.02,22091.19) | 459.64(452.82,466.55) | -0.18(-0.30,-0.06) | 0.37(0.28,0.46) |
| Tajikistan | male | 3339.77(2751.52,4028.70) | 311.21(300.55,322.19) | 6208.09(5171.19,7486.58) | 293.51(286.24,300.91) | 2.24(2.14,2.35) | -0.20(-0.25,-0.15) |
| Thailand | male | 49558.18(39557.88,60280.64) | 374.49(371.18,377.82) | 39200.58(32034.14,47651.47) | 388.20(384.35,392.09) | -0.91(-1.17,-0.64) | 0.07(-0.10,0.24) |
| Timor-Leste | male | 492.39(396.02,598.19) | 295.81(270.06,323.53) | 890.41(700.16,1091.81) | 300.23(280.07,321.67) | 1.95(1.75,2.15) | 0.08(0.06,0.11) |
| Togo | male | 968.52(775.25,1197.20) | 153.94(144.07,164.37) | 2603.24(2102.70,3131.54) | 165.06(158.68,171.64) | 3.23(3.18,3.27) | 0.30(0.26,0.34) |
| Tokelau | male | 1.10(0.86,1.39) | 356.85(10.87,2173.50) | 0.97(0.76,1.20) | 370.06(8.12,2292.80) | -0.44(-0.69,-0.19) | 0.13(0.12,0.14) |
| Tonga | male | 68.08(52.94,88.61) | 335.95(256.77,436.77) | 71.14(57.58,87.81) | 352.89(274.08,449.24) | 0.03(-0.08,0.14) | 0.14(0.14,0.15) |
| Trinidad and Tobago | male | 881.30(719.53,1108.50) | 348.15(325.49,372.03) | 897.95(731.32,1077.66) | 364.40(340.57,389.51) | -0.05(-0.26,0.16) | 0.19(0.17,0.20) |
| Tunisia | male | 3912.61(3242.37,4731.88) | 228.47(221.25,235.88) | 5573.42(4550.88,6636.00) | 257.78(251.00,264.71) | 1.12(0.88,1.35) | 0.43(0.41,0.45) |
| Türkiye | male | 24920.39(20369.88,30712.99) | 206.71(204.13,209.32) | 33225.19(26896.18,41429.92) | 203.47(201.28,205.68) | 0.88(0.77,0.98) | -0.01(-0.05,0.03) |
| Turkmenistan | male | 2473.68(2033.88,2985.40) | 319.10(306.49,332.14) | 3894.80(3251.38,4619.33) | 352.28(341.29,363.54) | 1.56(1.48,1.63) | 0.35(0.31,0.39) |
| Tuvalu | male | 5.95(4.69,7.37) | 344.07(125.18,768.63) | 9.65(7.69,11.88) | 356.81(168.12,672.49) | 1.77(1.66,1.89) | 0.11(0.11,0.12) |
| Uganda | male | 7222.78(5598.25,9314.22) | 221.07(215.81,226.45) | 19350.73(15289.53,24131.53) | 221.65(218.39,224.96) | 3.27(3.18,3.36) | 0.05(0.01,0.10) |
| Ukraine | male | 38504.45(31838.90,45719.01) | 410.54(406.43,414.68) | 37156.78(31789.40,43199.57) | 562.81(556.87,568.80) | 0.03(-0.11,0.17) | 1.07(0.98,1.16) |
| United Arab Emirates | male | 1941.52(1528.95,2403.50) | 285.06(271.27,299.55) | 10338.82(7826.08,13346.29) | 363.30(352.63,374.26) | 7.39(6.11,8.68) | 0.84(0.79,0.88) |
| United Kingdom | male | 77221.48(63329.44,91734.26) | 759.07(753.70,764.46) | 89779.24(74349.24,106993.34) | 863.57(857.89,869.28) | 0.55(0.44,0.66) | 0.33(0.19,0.46) |
| United Republic of Tanzania | male | 10353.04(8398.48,12754.29) | 218.46(214.14,222.86) | 25618.34(20463.00,32351.95) | 231.09(228.21,234.01) | 2.96(2.89,3.04) | 0.28(0.23,0.33) |
| United States of America | male | 369187.07(308457.80,440271.84) | 759.35(756.88,761.82) | 652598.62(569118.08,755839.90) | 1199.06(1196.15,1201.98) | 1.89(1.59,2.18) | 1.44(1.14,1.74) |
| United States Virgin Islands | male | 90.21(70.36,116.54) | 465.70(374.08,573.78) | 55.15(43.62,70.47) | 478.77(360.22,625.65) | -1.49(-1.69,-1.29) | 0.07(0.03,0.10) |
| Uruguay | male | 2412.64(2007.57,2907.56) | 421.32(404.67,438.50) | 2495.18(2087.41,2971.18) | 425.29(408.75,442.33) | 0.09(0.01,0.17) | 0.02(-0.01,0.05) |
| Uzbekistan | male | 13288.09(10955.99,16097.41) | 306.14(300.91,311.45) | 20515.25(16781.37,24433.98) | 299.80(295.69,303.96) | 1.58(1.44,1.72) | -0.05(-0.09,-0.01) |
| Vanuatu | male | 97.72(76.72,122.80) | 323.37(261.71,396.23) | 207.59(168.06,254.87) | 324.79(281.74,372.98) | 2.42(2.35,2.50) | 0.00(-0.01,0.02) |
| Venezuela (Bolivarian Republic of) | male | 10413.45(8373.35,13011.71) | 261.40(256.37,266.51) | 11154.59(8978.96,13678.66) | 251.40(246.70,256.18) | 1.01(0.59,1.43) | -0.11(-0.13,-0.10) |
| Viet Nam | male | 48048.21(38897.81,58217.78) | 344.02(340.91,347.15) | 77857.67(64366.18,93363.06) | 404.85(401.97,407.75) | 1.72(1.54,1.90) | 0.62(0.53,0.71) |
| Yemen | male | 4850.37(3984.73,5910.24) | 216.25(210.08,222.58) | 15537.87(12770.95,18611.42) | 226.89(223.30,230.52) | 3.90(3.74,4.06) | 0.22(0.18,0.26) |
| Zambia | male | 3268.91(2674.47,4021.58) | 215.48(207.78,223.44) | 8114.93(6660.57,9905.06) | 208.84(204.25,213.52) | 3.35(3.17,3.54) | 0.11(0.04,0.19) |
| Zimbabwe | male | 5583.79(4456.81,7034.76) | 295.60(287.59,303.80) | 9189.41(7254.99,11415.99) | 307.01(300.67,313.46) | 1.36(1.26,1.47) | 0.05(0.03,0.07) |

**Supplementary table 2.** DALYs cases and Age-standardized DALY rate of DUDs in 1990 and 2021 and its trends.

| location_name |  | 1990 |  | 2021 |  | EAPC_95%UI |  |
| --- | --- | --- | --- | --- | --- | --- | --- |
|  | sex | number_95%UI | ASR | number_95%UI | ASR | number_95%UI | ASR |
| Afghanistan | both | 9265.18(7029.03,11629.84) | 337.33(329.71,345.11) | 42614.93(33380.52,53076.93) | 359.17(355.62,362.74) | 4.52(3.98,5.06) | 0.45(0.25,0.65) |
| Albania | both | 2788.32(2190.74,3451.10) | 193.61(186.46,200.98) | 2489.60(1925.54,3107.71) | 254.78(244.84,265.05) | -0.24(-0.43,-0.05) | 1.04(0.73,1.34) |
| Algeria | both | 24461.54(18589.53,31672.19) | 249.17(245.99,252.38) | 53494.57(40885.85,66678.55) | 308.50(305.88,311.15) | 2.65(2.33,2.98) | 0.66(0.57,0.74) |
| American Samoa | both | 30.40(21.13,41.22) | 146.81(98.97,211.73) | 26.36(18.09,34.95) | 156.69(102.30,230.60) | -0.81(-1.02,-0.60) | 0.18(0.05,0.30) |
| Andorra | both | 52.68(37.11,70.87) | 206.04(153.39,272.95) | 54.48(37.88,71.80) | 216.39(161.93,284.74) | -0.33(-0.71,0.06) | 0.24(0.17,0.32) |
| Angola | both | 3712.13(2708.43,4839.91) | 98.31(95.14,101.56) | 12575.92(9213.31,16547.47) | 108.57(106.66,110.51) | 4.17(4.09,4.24) | 0.43(0.36,0.49) |
| Antigua and Barbuda | both | 45.38(32.20,61.73) | 173.86(126.88,233.41) | 61.77(44.57,80.96) | 178.45(136.63,229.65) | 0.69(0.14,1.24) | -0.15(-0.51,0.20) |
| Argentina | both | 25173.52(16707.02,34625.89) | 207.45(204.89,210.03) | 36676.71(25728.47,49480.29) | 208.61(206.48,210.76) | 1.42(1.24,1.60) | 0.13(0.00,0.26) |
| Armenia | both | 3710.84(2581.05,5060.62) | 251.49(243.43,259.77) | 2465.07(1666.83,3266.03) | 226.81(217.71,236.22) | -0.81(-1.08,-0.55) | -0.21(-0.38,-0.05) |
| Australia | both | 61131.40(49057.95,72149.02) | 902.35(895.20,909.54) | 82591.43(67950.30,97796.98) | 912.16(905.90,918.46) | 0.34(-0.06,0.74) | -0.57(-0.96,-0.17) |
| Austria | both | 13902.02(10716.17,17080.30) | 444.61(437.21,452.12) | 15146.20(12130.00,18010.16) | 527.76(519.29,536.35) | -0.27(-0.50,-0.03) | -0.01(-0.35,0.35) |
| Azerbaijan | both | 7477.35(5297.04,10058.19) | 229.31(224.10,234.62) | 9296.23(6564.77,12205.54) | 213.93(209.53,218.41) | 1.11(0.92,1.30) | -0.10(-0.20,0.00) |
| Bahamas | both | 221.93(161.77,302.24) | 185.44(161.72,211.91) | 288.08(207.74,385.53) | 186.80(165.85,209.69) | 0.51(0.24,0.78) | -0.21(-0.45,0.03) |
| Bahrain | both | 512.13(358.53,698.35) | 188.61(172.29,206.30) | 1341.44(947.65,1778.69) | 184.35(174.46,194.72) | 4.29(3.64,4.94) | 0.00(-0.11,0.10) |
| Bangladesh | both | 42366.54(30767.12,56627.09) | 101.79(100.81,102.78) | 74746.26(54084.09,99757.66) | 108.18(107.40,108.96) | 1.88(1.76,2.01) | 0.30(0.25,0.34) |
| Barbados | both | 204.23(141.17,280.64) | 186.04(161.39,213.47) | 181.09(129.89,241.50) | 184.09(158.19,213.16) | -0.50(-0.60,-0.41) | -0.18(-0.37,0.01) |
| Belarus | both | 18953.25(13879.93,24883.79) | 470.33(463.61,477.13) | 13661.40(11123.25,16704.79) | 446.39(438.65,454.25) | -1.39(-2.14,-0.62) | -0.69(-1.39,0.02) |
| Belgium | both | 10578.88(8188.26,13143.99) | 281.87(276.49,287.33) | 14736.07(12118.48,17827.18) | 412.40(405.72,419.16) | 0.71(0.55,0.87) | 0.79(0.59,0.99) |
| Belize | both | 126.47(87.85,171.62) | 170.18(141.23,204.05) | 313.71(221.90,432.61) | 165.40(147.54,184.91) | 2.98(2.80,3.16) | -0.18(-0.35,-0.01) |
| Benin | both | 1205.80(825.26,1679.20) | 71.96(67.92,76.21) | 3803.99(2560.93,5211.05) | 73.75(71.39,76.17) | 3.85(3.79,3.91) | 0.17(0.11,0.22) |
| Bermuda | both | 56.45(42.52,74.08) | 214.21(161.23,281.16) | 42.10(31.82,53.62) | 238.75(170.92,326.27) | -1.93(-2.65,-1.20) | -0.57(-1.22,0.09) |
| Bhutan | both | 268.84(189.79,365.54) | 102.42(90.21,116.01) | 385.26(279.16,521.77) | 108.95(98.33,120.45) | 1.74(1.51,1.96) | 0.25(0.19,0.31) |
| Bolivia (Plurinational State of) | both | 4769.57(3425.46,6146.91) | 194.34(188.81,199.99) | 9626.00(7007.25,12495.79) | 194.25(190.38,198.17) | 2.32(2.06,2.57) | -0.09(-0.21,0.02) |
| Bosnia and Herzegovina | both | 2278.92(1607.58,2996.37) | 118.76(113.93,123.74) | 1323.32(937.01,1772.58) | 130.12(123.15,137.40) | -0.74(-1.22,-0.25) | 0.81(0.56,1.07) |
| Botswana | both | 748.81(528.07,1009.19) | 151.95(141.05,163.52) | 1551.11(1103.51,2060.26) | 145.02(137.88,152.44) | 2.59(2.39,2.78) | 0.00(-0.10,0.09) |
| Brazil | both | 135418.83(91570.33,179885.14) | 215.00(213.85,216.16) | 233253.40(174358.17,292262.26) | 273.52(272.41,274.64) | 2.12(1.92,2.31) | 1.06(0.91,1.21) |
| Brunei Darussalam | both | 332.62(249.90,415.57) | 264.42(236.69,294.62) | 455.09(332.06,585.85) | 218.79(199.00,240.19) | 0.93(0.83,1.03) | -0.78(-0.89,-0.66) |
| Bulgaria | both | 5727.68(4307.06,7322.79) | 195.06(190.03,200.20) | 5037.61(3853.65,6286.28) | 274.51(266.79,282.41) | -0.39(-0.84,0.06) | 1.05(0.81,1.29) |
| Burkina Faso | both | 2017.41(1390.35,2754.56) | 66.34(63.43,69.36) | 5756.31(3877.60,7905.56) | 69.42(67.62,71.26) | 3.63(3.54,3.71) | 0.22(0.18,0.26) |
| Burundi | both | 2237.31(1604.09,3076.28) | 115.91(111.11,120.88) | 5922.74(4190.23,8090.80) | 121.03(117.95,124.17) | 3.48(3.07,3.89) | 0.09(0.02,0.16) |
| Cabo Verde | both | 110.13(77.89,148.83) | 85.57(69.80,104.47) | 211.85(147.68,285.94) | 83.49(72.61,95.60) | 2.41(2.25,2.58) | -0.02(-0.05,0.01) |
| Cambodia | both | 4829.51(3295.41,6617.80) | 124.49(120.96,128.10) | 9201.77(6169.87,12941.80) | 125.94(123.38,128.55) | 2.32(2.20,2.45) | 0.06(0.03,0.08) |
| Cameroon | both | 2993.14(2017.41,4175.17) | 80.96(78.04,83.96) | 10116.45(6895.23,13840.42) | 80.31(78.74,81.90) | 4.23(4.13,4.33) | 0.08(0.04,0.12) |
| Canada | both | 77570.52(57944.70,98345.13) | 684.30(679.44,689.18) | 204569.60(176385.82,234822.18) | 1665.49(1658.22,1672.78) | 3.16(2.57,3.76) | 2.94(2.48,3.39) |
| Central African Republic | both | 995.79(692.94,1341.61) | 98.48(92.36,104.94) | 2052.35(1463.33,2682.79) | 98.15(93.89,102.56) | 2.35(2.18,2.52) | 0.00(-0.03,0.03) |
| Chad | both | 1480.75(994.27,2083.65) | 73.33(69.60,77.23) | 4157.03(2789.39,5684.83) | 69.37(67.22,71.57) | 3.44(3.36,3.52) | -0.10(-0.14,-0.06) |
| Chile | both | 11951.11(8413.38,15862.57) | 206.70(203.01,210.46) | 17384.32(12641.84,22355.91) | 244.31(240.67,248.00) | 1.10(0.94,1.27) | 0.44(0.27,0.62) |
| China | both | 2735951.36(2147265.38,3309093.21) | 497.91(497.31,498.50) | 1073323.86(800801.22,1351708.79) | 230.43(229.99,230.88) | -4.16(-4.57,-3.74) | -3.71(-4.19,-3.23) |
| Colombia | both | 27060.00(19680.93,35582.55) | 190.51(188.23,192.81) | 40432.20(30200.25,52535.52) | 199.01(197.07,200.96) | 1.21(1.10,1.33) | 0.16(0.08,0.25) |
| Comoros | both | 171.75(118.63,237.22) | 114.39(97.54,133.48) | 412.92(298.19,550.14) | 139.83(126.64,154.06) | 2.65(2.47,2.83) | 0.46(0.26,0.66) |
| Congo | both | 962.45(703.99,1264.63) | 107.95(101.09,115.20) | 2510.32(1815.08,3164.38) | 116.44(111.92,121.10) | 3.16(3.00,3.32) | 0.26(0.21,0.31) |
| Cook Islands | both | 10.99(7.49,15.34) | 141.70(70.24,259.66) | 8.41(5.67,11.31) | 142.69(63.06,279.45) | -1.07(-1.19,-0.94) | 0.06(0.03,0.09) |
| Costa Rica | both | 2005.71(1421.47,2661.39) | 154.81(148.08,161.79) | 3242.89(2496.70,4149.71) | 168.31(162.55,174.24) | 1.50(1.23,1.76) | 0.18(0.07,0.30) |
| Croatia | both | 4707.27(3665.76,5757.04) | 260.25(252.83,267.84) | 4629.44(3624.30,5605.11) | 362.86(352.37,373.62) | 0.21(-0.42,0.85) | 1.23(0.57,1.89) |
| Cuba | both | 9208.91(6446.37,12325.29) | 186.43(182.60,190.33) | 5766.79(4008.42,7831.75) | 160.08(155.95,164.31) | -1.88(-2.00,-1.75) | -0.57(-0.60,-0.54) |
| Cyprus | both | 936.13(727.24,1145.23) | 300.01(281.08,319.93) | 1615.55(1264.14,2019.43) | 315.30(299.57,331.78) | 1.92(1.74,2.10) | -0.10(-0.27,0.08) |
| Czechia | both | 7646.86(5691.98,9831.26) | 212.87(208.10,217.72) | 8482.02(6571.20,10555.81) | 294.72(288.34,301.23) | 0.87(0.47,1.26) | 1.55(1.33,1.76) |
| C?te d'Ivoire | both | 3394.50(2314.22,4755.60) | 72.22(69.77,74.74) | 7885.56(5188.69,10843.44) | 71.50(69.93,73.10) | 2.74(2.53,2.96) | 0.03(-0.02,0.08) |
| Democratic People's Republic of Korea | both | 21408.60(15792.12,27673.21) | 252.81(249.42,256.24) | 22837.71(16268.32,29736.70) | 222.59(219.70,225.51) | 0.07(-0.03,0.17) | -0.48(-0.57,-0.39) |
| Democratic Republic of the Congo | both | 12602.05(9199.36,16762.30) | 91.59(89.97,93.23) | 36182.59(25821.33,47832.65) | 104.63(103.54,105.73) | 3.55(3.41,3.69) | 0.51(0.40,0.61) |
| Denmark | both | 13011.73(10744.88,15422.50) | 667.95(656.51,679.55) | 12533.86(10297.28,14651.08) | 671.00(659.27,682.90) | -0.49(-0.73,-0.25) | -0.14(-0.30,0.02) |
| Djibouti | both | 180.40(127.98,241.53) | 117.20(100.36,136.25) | 766.20(521.03,1050.75) | 139.59(129.87,149.84) | 4.74(4.50,4.97) | 0.48(0.37,0.59) |
| Dominica | both | 57.85(40.68,78.89) | 194.43(146.95,253.77) | 57.18(42.42,74.85) | 221.52(167.84,287.02) | -0.30(-0.44,-0.16) | 0.17(-0.05,0.38) |
| Dominican Republic | both | 3874.56(2638.84,5340.27) | 126.22(122.21,130.33) | 6472.64(4452.13,8792.92) | 141.09(137.67,144.57) | 1.68(1.61,1.76) | 0.38(0.32,0.44) |
| Ecuador | both | 6310.91(4514.62,8349.19) | 153.76(149.95,157.65) | 13456.24(10276.01,17189.00) | 182.20(179.14,185.31) | 2.55(2.36,2.74) | 0.67(0.51,0.83) |
| Egypt | both | 36508.35(23916.81,49743.50) | 168.75(167.01,170.50) | 79063.74(52437.88,105328.86) | 188.60(187.29,189.92) | 2.72(2.57,2.87) | 0.43(0.40,0.46) |
| El Salvador | both | 3349.10(2566.30,4265.85) | 164.86(159.22,170.66) | 4162.50(3104.05,5353.69) | 158.48(153.68,163.41) | 0.50(0.36,0.64) | -0.25(-0.37,-0.13) |
| Equatorial Guinea | both | 142.20(105.14,189.52) | 99.19(83.32,117.35) | 831.76(601.09,1090.79) | 124.15(115.75,133.06) | 6.18(6.07,6.29) | 0.84(0.79,0.89) |
| Eritrea | both | 1322.40(938.44,1741.07) | 114.86(108.65,121.36) | 3770.25(2572.60,5149.59) | 144.20(139.62,148.91) | 3.90(3.70,4.09) | 0.74(0.68,0.79) |
| Estonia | both | 3474.13(2771.48,4044.69) | 611.62(591.36,632.42) | 6522.06(5163.86,7897.87) | 1602.04(1562.32,1642.61) | 2.40(1.68,3.13) | 3.20(2.51,3.89) |
| Eswatini | both | 449.82(328.21,588.55) | 156.13(141.66,171.82) | 815.90(600.35,1062.58) | 160.93(150.04,172.43) | 1.89(1.67,2.11) | 0.16(0.08,0.23) |
| Ethiopia | both | 14151.56(10429.46,19050.50) | 82.70(81.33,84.10) | 34650.89(25636.15,45664.33) | 79.87(79.01,80.73) | 2.75(2.68,2.82) | -0.23(-0.27,-0.19) |
| Fiji | both | 471.17(327.69,642.38) | 144.22(131.45,157.97) | 465.68(316.40,638.86) | 131.24(119.58,143.72) | -0.12(-0.28,0.03) | -0.48(-0.57,-0.38) |
| Finland | both | 9494.30(7890.44,11071.47) | 503.65(493.47,514.00) | 17182.96(14462.12,19719.60) | 1014.62(999.38,1030.04) | 2.37(2.17,2.57) | 2.57(2.38,2.77) |
| France | both | 64534.24(51080.09,79044.80) | 293.98(291.71,296.26) | 70048.77(56708.93,84658.22) | 352.09(349.48,354.71) | 0.58(0.16,1.01) | 0.91(0.48,1.34) |
| Gabon | both | 444.67(325.51,577.86) | 120.28(109.24,132.20) | 894.69(665.67,1170.39) | 122.78(114.82,131.17) | 2.17(2.11,2.23) | -0.02(-0.06,0.03) |
| Gambia | both | 297.89(196.29,411.76) | 80.09(71.10,90.04) | 733.78(497.14,991.14) | 75.07(69.67,80.82) | 2.81(2.77,2.86) | -0.20(-0.21,-0.18) |
| Georgia | both | 5373.61(3726.65,7200.31) | 247.76(241.16,254.49) | 2668.03(1911.64,3470.76) | 232.49(223.63,241.64) | -1.69(-2.26,-1.12) | 0.29(-0.30,0.88) |
| Germany | both | 136587.98(112153.89,160374.12) | 438.29(435.93,440.65) | 118041.68(96187.52,141555.18) | 446.99(444.41,449.59) | -1.15(-1.38,-0.91) | -0.49(-0.63,-0.34) |
| Ghana | both | 4583.21(3058.59,6197.42) | 82.09(79.71,84.53) | 11868.94(7946.27,16429.87) | 83.31(81.81,84.83) | 3.23(3.17,3.30) | 0.05(-0.01,0.10) |
| Greece | both | 11350.02(8907.71,13900.08) | 300.80(295.29,306.39) | 14996.03(12380.07,17612.17) | 526.41(517.91,535.02) | -0.26(-1.43,0.93) | 0.75(-0.13,1.63) |
| Greenland | both | 148.11(112.09,185.62) | 530.75(446.91,628.47) | 125.83(99.42,153.95) | 598.92(498.22,715.48) | -0.41(-0.56,-0.26) | 0.54(0.43,0.65) |
| Grenada | both | 62.16(44.33,83.64) | 186.49(142.69,240.49) | 95.03(70.21,121.80) | 232.25(187.71,284.64) | 0.77(0.11,1.44) | 0.19(-0.17,0.55) |
| Guam | both | 113.87(81.62,148.93) | 173.76(143.21,209.18) | 82.98(56.86,115.01) | 147.89(117.77,183.45) | -1.15(-1.31,-0.98) | -0.55(-0.70,-0.40) |
| Guatemala | both | 5655.06(4696.44,6784.19) | 200.09(194.84,205.46) | 15532.83(12836.98,18556.60) | 228.98(225.36,232.65) | 3.60(3.26,3.95) | 0.53(0.15,0.92) |
| Guinea | both | 1361.77(924.83,1850.26) | 67.81(64.24,71.54) | 3298.87(2306.59,4500.70) | 65.58(63.33,67.88) | 2.79(2.68,2.89) | -0.08(-0.11,-0.06) |
| Guinea-Bissau | both | 247.10(161.55,345.56) | 68.98(60.53,78.38) | 566.28(386.88,745.98) | 68.27(62.72,74.20) | 2.90(2.82,2.99) | 0.02(-0.02,0.06) |
| Guyana | both | 486.17(338.93,659.19) | 142.18(129.68,155.65) | 474.27(341.59,635.50) | 149.42(136.17,163.70) | -0.44(-0.60,-0.27) | 0.00(-0.10,0.11) |
| Haiti | both | 3755.32(2661.49,4928.63) | 155.41(150.45,160.50) | 9428.95(6860.52,12326.20) | 172.05(168.59,175.56) | 3.16(2.98,3.34) | 0.30(0.24,0.36) |
| Honduras | both | 3150.11(2460.96,3942.21) | 188.69(182.06,195.52) | 8048.25(5802.73,10632.61) | 183.83(179.81,187.93) | 2.99(2.90,3.08) | -0.25(-0.31,-0.19) |
| Hungary | both | 6839.29(4837.86,9044.39) | 191.21(186.66,195.84) | 6408.04(4733.60,8281.93) | 234.03(228.28,239.90) | -0.09(-0.34,0.16) | 0.76(0.64,0.88) |
| Iceland | both | 546.95(444.61,658.38) | 520.49(477.75,566.13) | 1281.20(1084.44,1481.52) | 1035.08(978.93,1093.83) | 2.92(2.66,3.18) | 2.28(2.07,2.48) |
| India | both | 393649.86(301547.32,507632.31) | 116.11(115.74,116.47) | 811399.45(613730.57,1048057.48) | 132.90(132.61,133.19) | 1.99(1.65,2.33) | 0.01(-0.34,0.36) |
| Indonesia | both | 73227.50(48341.43,104972.72) | 93.38(92.70,94.06) | 112923.37(76456.32,160197.26) | 98.61(98.04,99.19) | 1.11(0.98,1.24) | 0.00(-0.10,0.10) |
| Iran (Islamic Republic of) | both | 128261.57(104056.29,152251.37) | 620.32(616.87,623.78) | 233459.56(191621.51,277530.31) | 664.78(662.00,667.57) | 2.25(1.47,3.04) | 0.18(-0.24,0.60) |
| Iraq | both | 17213.08(12954.43,22037.01) | 249.54(245.75,253.37) | 48233.84(36127.63,61813.05) | 279.20(276.71,281.72) | 3.21(2.82,3.60) | 0.27(0.08,0.45) |
| Ireland | both | 4996.61(3958.01,6189.58) | 369.85(359.64,380.28) | 12660.72(10458.29,14847.77) | 812.24(798.03,826.65) | 2.61(1.92,3.30) | 1.82(1.34,2.31) |
| Israel | both | 4753.01(3588.34,5979.47) | 253.96(246.78,261.30) | 9985.70(7926.40,12199.19) | 302.72(296.81,308.73) | 2.17(1.68,2.65) | 0.32(-0.06,0.69) |
| Italy | both | 132392.66(107698.23,156822.70) | 609.08(605.80,612.37) | 45057.69(33330.23,57366.06) | 284.11(281.48,286.77) | -4.31(-4.61,-4.00) | -3.25(-3.50,-3.00) |
| Jamaica | both | 1847.82(1297.48,2472.47) | 185.35(176.88,194.17) | 2224.69(1611.34,3021.74) | 184.33(176.72,192.18) | 0.38(0.20,0.56) | -0.25(-0.41,-0.09) |
| Japan | both | 76598.16(54154.91,102592.96) | 174.29(173.05,175.53) | 55975.31(40339.39,74301.79) | 172.45(171.01,173.89) | -1.19(-1.51,-0.86) | -0.04(-0.20,0.13) |
| Jordan | both | 2842.97(1929.52,3885.22) | 189.30(182.11,196.74) | 9790.07(6577.61,13600.77) | 183.65(180.03,187.34) | 4.19(4.01,4.38) | 0.02(-0.08,0.11) |
| Kazakhstan | both | 24145.88(18340.23,30350.54) | 352.00(347.56,356.48) | 43245.70(33667.46,52702.04) | 589.50(583.89,595.16) | 1.79(0.95,2.64) | 1.23(0.22,2.25) |
| Kenya | both | 5765.57(4266.58,7487.38) | 73.33(71.39,75.31) | 15743.97(11799.71,20037.32) | 78.40(77.17,79.64) | 3.38(3.18,3.58) | 0.28(0.18,0.38) |
| Kiribati | both | 155.44(119.04,199.43) | 490.21(415.63,575.46) | 309.24(228.44,418.84) | 616.21(549.39,689.15) | 2.60(2.37,2.83) | 0.75(0.63,0.87) |
| Kuwait | both | 1847.48(1307.98,2511.59) | 208.32(198.83,218.21) | 5336.39(4034.58,6843.13) | 243.00(236.12,250.06) | 4.23(3.89,4.56) | 0.50(0.28,0.72) |
| Kyrgyzstan | both | 5170.38(3885.77,6532.30) | 287.86(280.03,295.87) | 8657.15(6676.26,10826.49) | 311.36(304.82,318.01) | 2.03(1.35,2.72) | 0.53(-0.19,1.25) |
| Lao People's Democratic Republic | both | 1779.78(1187.52,2437.40) | 116.42(111.03,122.03) | 3767.27(2576.28,5179.83) | 116.11(112.43,119.89) | 2.53(2.30,2.77) | -0.05(-0.19,0.08) |
| Latvia | both | 4251.52(3275.23,5097.39) | 441.02(427.82,454.54) | 2928.06(2360.92,3450.07) | 535.82(516.01,556.26) | -1.86(-2.26,-1.46) | -0.19(-0.55,0.17) |
| Lebanon | both | 2343.10(1607.53,3082.87) | 205.39(197.08,213.98) | 6605.30(4868.23,8481.93) | 277.88(271.07,284.83) | 4.06(3.67,4.44) | 1.13(1.07,1.20) |
| Lesotho | both | 735.25(516.20,986.03) | 142.28(132.08,153.08) | 1221.53(884.95,1626.34) | 148.26(140.01,156.88) | 1.83(1.73,1.94) | 0.29(0.23,0.35) |
| Liberia | both | 704.26(482.93,969.75) | 77.42(71.77,83.42) | 1706.78(1149.09,2361.14) | 78.02(74.34,81.85) | 4.37(3.93,4.82) | 0.07(0.02,0.11) |
| Libya | both | 4228.14(3186.13,5440.23) | 265.79(257.68,274.12) | 13048.89(10641.09,16409.63) | 428.30(420.95,435.75) | 4.18(3.81,4.56) | 1.81(1.71,1.90) |
| Lithuania | both | 5306.82(4175.51,6328.40) | 374.49(364.46,384.73) | 6733.81(5455.61,8068.29) | 784.30(765.50,803.50) | 0.43(0.18,0.68) | 2.25(2.01,2.50) |
| Luxembourg | both | 878.01(712.93,1044.18) | 575.99(538.01,616.28) | 1155.43(944.42,1388.42) | 516.80(486.86,548.29) | -0.10(-0.44,0.24) | -1.29(-1.68,-0.89) |
| Madagascar | both | 4451.35(3286.66,5970.81) | 107.75(104.55,111.02) | 12773.16(9297.07,16908.10) | 120.40(118.30,122.54) | 3.36(3.30,3.41) | 0.27(0.21,0.32) |
| Malawi | both | 3749.00(2716.20,5121.99) | 111.28(107.66,115.00) | 9567.64(6834.50,12876.23) | 134.84(132.11,137.62) | 3.06(2.90,3.23) | 0.59(0.55,0.64) |
| Malaysia | both | 10286.44(6944.71,14115.73) | 138.14(135.48,140.84) | 19528.82(13336.99,26442.23) | 137.81(135.88,139.76) | 2.20(2.02,2.38) | 0.01(-0.02,0.05) |
| Maldives | both | 106.75(70.74,150.97) | 129.86(105.75,158.78) | 411.07(285.12,552.38) | 154.84(139.32,171.95) | 5.02(4.77,5.28) | 0.69(0.64,0.75) |
| Mali | both | 2046.59(1353.61,2803.92) | 70.61(67.55,73.77) | 5859.52(3924.88,8186.48) | 68.46(66.67,70.28) | 3.59(3.46,3.71) | -0.06(-0.09,-0.03) |
| Malta | both | 399.71(307.19,497.24) | 292.30(264.14,322.78) | 642.04(511.59,764.16) | 470.63(434.15,509.73) | 1.83(1.27,2.39) | 1.52(1.04,1.99) |
| Marshall Islands | both | 26.35(18.21,35.82) | 154.62(100.83,228.89) | 36.20(25.26,48.52) | 153.94(107.88,213.30) | 0.92(0.74,1.10) | -0.03(-0.07,0.01) |
| Mauritania | both | 592.01(408.00,811.70) | 79.19(72.87,85.94) | 1274.57(859.90,1764.96) | 77.19(72.96,81.62) | 2.53(2.50,2.56) | 0.00(-0.04,0.04) |
| Mauritius | both | 950.07(629.02,1312.27) | 186.76(175.05,199.06) | 1501.24(1206.91,1835.37) | 324.01(307.81,340.88) | 1.45(1.21,1.69) | 1.78(1.61,1.96) |
| Mexico | both | 60360.46(44437.89,78164.52) | 170.88(169.50,172.27) | 87875.24(66615.71,111572.59) | 170.32(169.20,171.45) | 1.12(0.93,1.32) | -0.01(-0.13,0.11) |
| Micronesia (Federated States of) | both | 62.95(44.18,83.95) | 160.23(122.79,206.15) | 67.30(46.44,90.04) | 157.36(121.84,200.55) | 0.00(-0.09,0.09) | -0.11(-0.14,-0.08) |
| Monaco | both | 22.52(15.46,30.17) | 239.10(149.50,371.50) | 24.50(17.52,32.19) | 262.85(168.85,392.35) | 0.22(0.20,0.24) | 0.36(0.27,0.45) |
| Mongolia | both | 2021.94(1417.90,2718.17) | 228.38(218.34,238.81) | 3253.87(2348.26,4257.55) | 255.47(246.69,264.51) | 1.94(1.55,2.33) | 0.68(0.44,0.92) |
| Montenegro | both | 376.49(261.65,495.51) | 149.50(134.78,165.42) | 311.83(216.85,415.78) | 151.45(135.04,169.39) | -0.37(-0.46,-0.29) | 0.20(0.15,0.25) |
| Morocco | both | 26948.79(20097.65,35160.50) | 263.23(260.07,266.43) | 43461.17(32817.92,56521.81) | 295.25(292.48,298.04) | 1.49(1.30,1.68) | 0.29(0.21,0.38) |
| Mozambique | both | 3708.78(2645.93,4949.01) | 83.38(80.69,86.14) | 12099.08(8737.71,16030.45) | 113.44(111.38,115.53) | 3.96(3.86,4.06) | 1.14(1.09,1.19) |
| Myanmar | both | 23507.73(16293.08,33194.90) | 135.57(133.83,137.33) | 28646.26(19000.03,39591.64) | 127.20(125.73,128.68) | 0.43(0.27,0.60) | -0.28(-0.36,-0.20) |
| Namibia | both | 850.02(599.42,1146.44) | 157.37(146.74,168.64) | 1627.87(1158.50,2118.62) | 155.84(148.34,163.64) | 2.08(1.91,2.26) | 0.02(-0.02,0.06) |
| Nauru | both | 6.35(4.49,8.61) | 156.75(59.38,341.70) | 7.19(5.00,9.64) | 153.93(62.61,320.57) | 0.25(0.18,0.33) | -0.08(-0.13,-0.04) |
| Nepal | both | 7848.04(5650.62,10743.98) | 109.30(106.88,111.77) | 14605.50(10341.11,19662.39) | 108.95(107.17,110.74) | 1.97(1.88,2.07) | -0.04(-0.08,0.00) |
| Netherlands | both | 16179.58(11984.50,20765.43) | 266.74(262.63,270.90) | 17812.66(13790.47,21927.61) | 333.72(328.83,338.67) | -0.21(-0.51,0.09) | 0.36(0.13,0.59) |
| New Zealand | both | 5888.04(4236.51,7613.52) | 426.73(415.89,437.77) | 10259.29(7865.39,12538.93) | 556.54(545.76,567.48) | 1.11(0.85,1.37) | 0.35(0.14,0.56) |
| Nicaragua | both | 2019.23(1405.36,2684.73) | 141.10(134.89,147.55) | 3602.29(2548.51,4758.20) | 125.81(121.74,129.99) | 1.88(1.67,2.10) | -0.38(-0.46,-0.31) |
| Niger | both | 1940.03(1351.65,2756.82) | 72.11(68.89,75.45) | 6070.98(3982.36,8529.74) | 71.83(69.98,73.73) | 3.69(3.65,3.72) | 0.11(0.04,0.19) |
| Nigeria | both | 20781.84(14594.39,28032.53) | 62.86(62.00,63.73) | 52218.94(36079.60,70744.29) | 60.75(60.22,61.28) | 3.01(2.93,3.09) | -0.04(-0.08,0.00) |
| Niue | both | 1.26(0.88,1.66) | 158.45(7.51,797.85) | 0.86(0.59,1.15) | 152.88(2.25,985.41) | -1.61(-1.93,-1.29) | -0.16(-0.19,-0.14) |
| North Macedonia | both | 1415.55(1018.77,1854.10) | 177.71(168.57,187.22) | 1548.83(1135.03,1977.28) | 200.54(190.52,211.01) | 1.32(0.84,1.80) | 1.14(0.77,1.51) |
| Northern Mariana Islands | both | 39.84(27.75,53.20) | 166.24(118.46,228.50) | 28.22(19.57,38.29) | 175.57(116.68,254.83) | -2.27(-3.03,-1.49) | 0.16(-0.01,0.33) |
| Norway | both | 9645.77(7752.29,11675.87) | 594.14(582.33,606.13) | 15548.60(12790.55,18420.51) | 859.43(845.89,873.13) | 0.36(-0.49,1.21) | 0.04(-0.91,0.99) |
| Oman | both | 1527.72(1077.09,2089.85) | 180.56(171.57,189.93) | 4243.14(3035.69,5647.28) | 177.30(171.64,183.13) | 3.95(3.75,4.15) | 0.06(-0.07,0.18) |
| Pakistan | both | 50670.09(37101.26,67147.29) | 128.29(127.15,129.43) | 118394.04(88034.38,154119.60) | 119.85(119.17,120.54) | 3.22(2.65,3.79) | 0.12(-0.41,0.65) |
| Palau | both | 9.97(6.75,13.87) | 141.86(67.88,264.26) | 8.60(5.67,11.72) | 149.78(66.73,291.30) | -0.90(-1.31,-0.49) | 0.17(0.15,0.20) |
| Palestine | both | 1419.18(940.18,1998.27) | 187.51(177.57,197.94) | 3840.15(2575.44,5361.78) | 174.07(168.56,179.73) | 3.42(3.21,3.63) | -0.17(-0.26,-0.08) |
| Panama | both | 1649.02(1199.35,2158.51) | 162.10(154.31,170.21) | 2704.16(2031.91,3485.18) | 162.82(156.73,169.08) | 1.39(1.25,1.52) | -0.04(-0.15,0.06) |
| Papua New Guinea | both | 2264.67(1532.74,3171.51) | 134.22(128.70,139.95) | 5833.43(4009.17,7969.33) | 134.04(130.62,137.54) | 3.18(3.14,3.22) | -0.01(-0.03,0.01) |
| Paraguay | both | 2414.75(1625.96,3246.39) | 155.56(149.38,161.93) | 5005.75(3501.15,6696.84) | 162.63(158.16,167.20) | 2.63(2.51,2.75) | 0.23(0.19,0.27) |
| Peru | both | 14347.01(10216.88,18588.90) | 161.87(159.20,164.57) | 25747.63(19417.75,32831.02) | 171.14(169.05,173.25) | 1.93(1.78,2.08) | 0.32(0.15,0.48) |
| Philippines | both | 31822.93(21676.73,43481.41) | 123.28(121.92,124.66) | 56608.16(38332.19,76637.76) | 118.70(117.72,119.68) | 1.85(1.78,1.93) | -0.12(-0.17,-0.07) |
| Poland | both | 32233.76(24303.15,41107.31) | 229.91(227.37,232.46) | 29627.40(22925.26,36676.76) | 250.30(247.37,253.25) | -0.44(-0.61,-0.27) | -0.18(-0.56,0.20) |
| Portugal | both | 10741.85(8403.66,13131.66) | 285.20(279.83,290.65) | 8048.90(6277.33,9867.67) | 271.44(265.51,277.49) | -1.90(-2.27,-1.52) | -1.02(-1.34,-0.69) |
| Puerto Rico | both | 4632.03(3375.27,5872.99) | 331.37(321.89,341.06) | 4739.21(3936.29,5655.25) | 450.64(437.89,463.68) | -2.64(-4.15,-1.10) | -1.72(-3.12,-0.29) |
| Qatar | both | 445.08(316.20,608.77) | 179.64(162.58,198.40) | 3018.12(2141.47,4088.64) | 173.62(166.52,181.09) | 8.63(7.56,9.72) | -0.04(-0.17,0.09) |
| Republic of Korea | both | 42363.39(29865.16,56431.30) | 198.89(196.99,200.79) | 33130.47(23025.99,44407.75) | 202.11(199.90,204.34) | -0.93(-1.07,-0.80) | -0.07(-0.17,0.03) |
| Republic of Moldova | both | 5467.64(4001.66,6916.49) | 316.19(307.80,324.77) | 3725.58(2823.60,4642.78) | 298.78(288.86,308.98) | -1.04(-1.35,-0.73) | -0.33(-0.52,-0.13) |
| Romania | both | 14180.58(10789.10,17489.31) | 165.48(162.74,168.25) | 8353.27(6716.64,10160.17) | 155.16(151.81,158.57) | -2.06(-2.22,-1.89) | -0.52(-0.81,-0.23) |
| Russian Federation | both | 421076.23(330616.75,507006.69) | 722.79(720.58,725.00) | 461582.57(389777.85,539688.16) | 937.37(934.55,940.19) | -0.14(-1.29,1.01) | 0.06(-1.02,1.14) |
| Rwanda | both | 3261.22(2403.16,4326.36) | 129.82(125.36,134.40) | 7433.47(5265.85,10179.21) | 139.61(136.44,142.84) | 3.09(2.69,3.50) | 0.07(-0.03,0.18) |
| Saint Kitts and Nevis | both | 29.86(20.92,40.36) | 172.53(116.02,248.99) | 38.71(27.24,51.63) | 170.43(120.80,234.64) | 0.70(0.48,0.93) | -0.32(-0.50,-0.14) |
| Saint Lucia | both | 108.89(74.75,147.97) | 188.97(154.56,229.68) | 119.41(82.95,158.60) | 179.84(148.91,215.61) | 0.34(-0.01,0.68) | -0.13(-0.34,0.09) |
| Saint Vincent and the Grenadines | both | 74.48(50.31,100.75) | 161.52(126.32,204.77) | 71.77(51.57,95.63) | 173.39(135.59,218.61) | -0.54(-0.79,-0.28) | -0.09(-0.30,0.12) |
| Samoa | both | 106.16(72.27,144.33) | 155.97(127.00,190.47) | 131.65(92.10,174.27) | 161.51(134.95,192.11) | 0.44(0.32,0.56) | 0.01(-0.04,0.05) |
| San Marino | both | 22.76(15.51,30.53) | 238.25(150.49,360.15) | 22.13(15.43,29.38) | 247.36(155.13,375.30) | -0.32(-0.51,-0.13) | 0.23(0.15,0.32) |
| Sao Tome and Principe | both | 35.78(24.87,49.52) | 87.40(60.41,123.93) | 87.94(62.54,115.36) | 98.60(78.98,121.77) | 2.98(2.94,3.03) | 0.38(0.32,0.44) |
| Saudi Arabia | both | 12099.85(7854.27,16730.69) | 182.66(179.40,185.97) | 35888.60(24696.63,49995.50) | 189.05(187.04,191.07) | 4.11(3.84,4.37) | 0.27(0.17,0.37) |
| Senegal | both | 2114.99(1418.07,2912.24) | 79.50(76.09,83.04) | 4673.33(3102.65,6421.97) | 74.24(72.10,76.43) | 2.52(2.42,2.62) | -0.21(-0.23,-0.19) |
| Serbia | both | 6453.32(5082.32,8046.75) | 180.02(175.65,184.48) | 6492.26(5023.46,8046.99) | 215.91(210.64,221.28) | 0.20(-0.13,0.53) | 0.94(0.54,1.34) |
| Seychelles | both | 56.47(39.70,76.37) | 177.25(133.71,232.05) | 73.69(54.59,95.19) | 190.50(149.25,240.19) | 0.98(0.70,1.25) | 0.31(0.14,0.48) |
| Sierra Leone | both | 1289.11(874.77,1767.19) | 81.10(76.70,85.70) | 2783.54(1901.44,3910.23) | 75.51(72.70,78.41) | 2.98(2.68,3.29) | -0.23(-0.26,-0.20) |
| Singapore | both | 3029.39(2064.68,4040.78) | 195.66(188.73,202.80) | 3699.29(2567.46,5006.10) | 194.22(187.58,201.06) | 0.98(0.54,1.42) | -0.16(-0.26,-0.05) |
| Slovakia | both | 4740.13(3493.25,5969.23) | 236.75(230.03,243.62) | 3673.60(2739.91,4660.59) | 216.75(209.62,224.08) | -0.79(-1.22,-0.36) | -0.43(-0.66,-0.20) |
| Slovenia | both | 1751.43(1418.83,2136.97) | 229.64(218.96,240.73) | 2120.37(1667.82,2585.74) | 375.84(359.66,392.62) | 1.31(0.62,2.00) | 2.15(1.61,2.69) |
| Solomon Islands | both | 182.43(126.98,245.71) | 141.64(121.33,164.78) | 395.49(285.74,526.46) | 144.14(130.21,159.22) | 2.30(2.15,2.44) | 0.01(-0.04,0.05) |
| Somalia | both | 2936.11(2074.60,4207.65) | 108.97(105.04,113.01) | 8193.22(5701.66,11575.34) | 112.63(110.15,115.15) | 4.13(3.88,4.37) | 0.16(0.11,0.20) |
| South Africa | both | 55030.70(42553.62,68098.24) | 358.41(355.40,361.45) | 61844.30(48893.32,74934.78) | 251.11(249.13,253.10) | 0.06(-0.58,0.70) | -1.50(-2.06,-0.94) |
| South Sudan | both | 2219.47(1603.05,2983.67) | 107.93(103.37,112.67) | 3494.39(2527.79,4802.54) | 111.32(107.58,115.17) | 1.67(1.30,2.05) | 0.08(0.01,0.15) |
| Spain | both | 88318.42(71622.03,105576.18) | 589.79(585.90,593.69) | 52065.51(40653.92,64237.82) | 423.79(420.11,427.49) | -2.82(-3.56,-2.07) | -2.30(-2.83,-1.77) |
| Sri Lanka | both | 16506.25(12573.87,20745.68) | 223.66(220.26,227.11) | 13380.31(9549.39,17592.64) | 167.53(164.70,170.40) | -1.35(-1.63,-1.06) | -1.56(-1.83,-1.29) |
| Sudan | both | 17055.05(12678.97,22521.99) | 232.26(228.73,235.83) | 54031.74(41088.75,67897.99) | 298.66(296.12,301.21) | 3.72(3.66,3.78) | 0.85(0.79,0.90) |
| Suriname | both | 262.10(182.71,346.27) | 159.20(140.20,180.35) | 420.68(313.31,544.01) | 196.17(177.86,215.88) | 1.34(0.88,1.81) | 0.35(0.04,0.66) |
| Sweden | both | 7733.56(6368.54,9227.16) | 257.94(252.21,263.77) | 28761.42(23380.74,34213.50) | 869.59(859.46,879.82) | 4.95(4.65,5.25) | 4.61(4.29,4.93) |
| Switzerland | both | 35545.89(28766.92,42183.36) | 1299.60(1286.00,1313.31) | 14830.59(12183.59,17774.99) | 523.67(515.11,532.34) | -3.42(-3.76,-3.08) | -3.62(-3.89,-3.35) |
| Syrian Arab Republic | both | 10145.57(7514.76,13227.83) | 216.98(212.67,221.36) | 11262.96(8290.58,14180.87) | 246.00(240.98,251.11) | 0.55(-0.61,1.72) | 0.54(0.46,0.63) |
| Taiwan (Province of China) | both | 27943.35(22214.91,34622.53) | 297.47(293.99,300.99) | 25194.98(19327.96,32010.19) | 313.61(309.69,317.57) | -0.64(-0.91,-0.38) | -0.23(-0.57,0.12) |
| Tajikistan | both | 4768.18(3309.68,6433.65) | 225.48(219.03,232.10) | 8973.31(6352.66,11674.47) | 211.55(207.19,215.99) | 2.35(2.21,2.49) | -0.17(-0.22,-0.11) |
| Thailand | both | 54238.63(34644.52,76592.06) | 205.68(203.94,207.42) | 47049.35(31825.79,64211.20) | 221.62(219.62,223.65) | -0.71(-1.12,-0.30) | 0.18(-0.11,0.46) |
| Timor-Leste | both | 426.26(281.35,593.50) | 130.76(118.56,143.97) | 758.99(496.29,1081.38) | 131.98(122.53,142.07) | 1.85(1.61,2.09) | 0.05(0.01,0.09) |
| Togo | both | 877.95(575.12,1200.84) | 66.19(61.80,70.83) | 2295.22(1527.59,3121.54) | 69.73(66.90,72.66) | 3.27(3.19,3.35) | 0.33(0.27,0.39) |
| Tokelau | both | 0.88(0.62,1.19) | 152.86(2.36,1040.03) | 0.75(0.52,1.00) | 151.67(1.29,1099.68) | -0.78(-1.12,-0.44) | -0.08(-0.13,-0.04) |
| Tonga | both | 52.88(36.22,71.49) | 142.89(105.98,190.31) | 57.56(39.93,78.64) | 147.61(111.64,192.09) | 0.11(-0.02,0.24) | 0.06(0.03,0.08) |
| Trinidad and Tobago | both | 808.88(554.33,1096.89) | 160.25(149.37,171.74) | 962.68(722.65,1217.61) | 194.05(181.80,206.94) | 0.50(0.11,0.89) | 0.43(0.17,0.68) |
| Tunisia | both | 8149.85(6150.24,10475.30) | 240.67(235.42,246.03) | 13466.24(10256.19,16958.00) | 305.49(300.31,310.75) | 1.71(1.37,2.04) | 0.79(0.67,0.90) |
| Türkiye | both | 48411.60(33297.87,65752.06) | 205.08(203.24,206.92) | 67268.96(48111.70,89308.03) | 209.33(207.75,210.92) | 1.09(0.94,1.25) | 0.19(0.14,0.24) |
| Turkmenistan | both | 3691.15(2570.96,4890.39) | 240.34(232.58,248.31) | 9184.81(6997.22,11402.05) | 436.96(428.07,446.00) | 3.22(2.95,3.50) | 2.15(1.88,2.42) |
| Tuvalu | both | 5.45(3.78,7.40) | 149.71(51.58,341.59) | 7.58(5.23,10.24) | 151.17(63.33,308.79) | 1.26(1.04,1.47) | 0.02(0.00,0.04) |
| Uganda | both | 6421.41(4527.52,8593.15) | 110.96(108.17,113.81) | 20054.43(15095.90,26174.34) | 133.63(131.74,135.55) | 3.63(3.54,3.72) | 0.49(0.43,0.55) |
| Ukraine | both | 65353.55(50475.50,82299.99) | 337.67(335.07,340.29) | 82095.51(63588.27,101408.25) | 575.74(571.65,579.86) | 0.74(0.33,1.15) | 1.49(1.16,1.82) |
| United Arab Emirates | both | 2474.89(1778.76,3204.88) | 245.43(235.49,255.76) | 14677.67(10715.22,18883.43) | 385.52(377.31,393.90) | 8.07(6.63,9.52) | 1.72(1.61,1.84) |
| United Kingdom | both | 107182.07(83260.93,131048.42) | 503.72(500.70,506.75) | 225315.87(188064.72,260991.25) | 1000.86(996.71,1005.03) | 1.93(1.52,2.35) | 1.70(1.19,2.21) |
| United Republic of Tanzania | both | 11899.44(8550.55,16047.66) | 138.65(136.12,141.22) | 33535.19(24312.98,44903.21) | 161.18(159.44,162.93) | 3.22(3.13,3.32) | 0.36(0.32,0.40) |
| United States of America | both | 744902.41(568062.38,923941.17) | 710.92(709.30,712.55) | 4009833.62(3332586.37,4692156.36) | 3520.13(3516.68,3523.58) | 6.12(5.64,6.60) | 5.81(5.41,6.22) |
| United States Virgin Islands | both | 121.62(95.25,151.80) | 312.56(259.36,373.71) | 112.18(83.79,152.51) | 487.36(400.85,587.77) | -0.11(-0.32,0.11) | 1.53(1.29,1.78) |
| Uruguay | both | 2786.86(1948.11,3779.53) | 245.72(236.67,255.01) | 2942.31(2097.39,3836.12) | 245.93(237.11,254.99) | 0.20(0.04,0.36) | 0.05(-0.07,0.17) |
| Uzbekistan | both | 18171.05(12629.62,24564.19) | 211.06(207.98,214.18) | 29910.68(20859.34,38470.29) | 213.49(211.06,215.93) | 2.04(1.78,2.29) | 0.23(0.08,0.38) |
| Vanuatu | both | 80.17(55.40,108.76) | 135.71(107.42,169.68) | 170.39(119.00,229.33) | 134.96(115.36,157.12) | 2.46(2.41,2.51) | -0.06(-0.08,-0.05) |
| Venezuela (Bolivarian Republic of) | both | 11011.48(7561.08,14990.57) | 138.77(136.17,141.40) | 11698.76(8123.07,15370.55) | 128.44(126.09,130.81) | 1.00(0.52,1.48) | -0.21(-0.32,-0.10) |
| Viet Nam | both | 61253.39(46065.13,79357.23) | 215.28(213.56,217.01) | 116135.40(85252.34,150234.04) | 297.21(295.48,298.95) | 2.29(2.15,2.43) | 1.09(0.88,1.30) |
| Yemen | both | 8032.96(5707.92,11008.16) | 183.96(179.92,188.07) | 30334.35(21812.97,41021.48) | 229.68(227.10,232.29) | 4.70(4.37,5.02) | 0.80(0.62,0.98) |
| Zambia | both | 3547.28(2653.21,4715.48) | 136.40(131.79,141.15) | 10928.55(7674.32,14627.01) | 152.13(149.25,155.04) | 3.86(3.68,4.04) | 0.41(0.35,0.47) |
| Zimbabwe | both | 6057.87(4232.84,8196.99) | 161.20(157.06,165.42) | 9841.23(7189.39,12650.63) | 159.17(156.02,162.38) | 1.37(1.29,1.45) | -0.08(-0.15,-0.01) |
| Afghanistan | female | 6435.46(4717.44,8237.80) | 406.35(395.63,417.34) | 24229.33(18046.78,30720.83) | 412.46(407.07,417.90) | 3.82(3.35,4.28) | 0.21(-0.02,0.45) |
| Albania | female | 784.75(578.24,1017.33) | 109.96(102.36,118.02) | 525.29(384.66,695.65) | 114.16(104.58,124.44) | -1.25(-1.44,-1.06) | 0.25(0.02,0.47) |
| Algeria | female | 10939.57(7718.78,14989.55) | 222.78(218.53,227.09) | 20100.63(14197.74,26827.06) | 237.10(233.81,240.43) | 2.16(1.83,2.49) | 0.23(0.17,0.30) |
| American Samoa | female | 11.72(7.94,16.63) | 113.10(57.41,205.35) | 9.64(6.39,13.34) | 116.72(54.80,219.49) | -0.90(-1.15,-0.64) | 0.10(0.00,0.21) |
| Andorra | female | 16.67(11.52,22.59) | 141.82(81.40,234.17) | 19.72(13.63,26.41) | 161.41(97.12,255.89) | 0.09(-0.28,0.47) | 0.51(0.45,0.57) |
| Angola | female | 1465.31(992.22,2040.76) | 77.66(73.69,81.79) | 4979.48(3448.27,7161.63) | 80.85(78.59,83.15) | 4.21(4.12,4.30) | 0.23(0.18,0.28) |
| Antigua and Barbuda | female | 21.54(14.68,30.40) | 160.20(99.72,245.38) | 27.63(19.02,37.64) | 157.43(104.11,230.15) | 0.63(0.26,1.01) | -0.10(-0.21,0.02) |
| Argentina | female | 10610.29(6893.44,14853.50) | 173.63(170.34,176.97) | 15504.45(10505.17,21509.87) | 174.66(171.92,177.44) | 1.46(1.28,1.65) | 0.16(0.03,0.29) |
| Armenia | female | 1779.75(1165.88,2483.23) | 237.04(226.09,248.41) | 1210.63(799.72,1664.79) | 221.69(208.97,235.07) | -0.71(-0.92,-0.50) | -0.08(-0.17,0.01) |
| Australia | female | 17754.34(13649.73,21557.33) | 528.03(520.27,535.87) | 26397.07(20609.88,32016.48) | 587.41(580.26,594.63) | 0.94(0.65,1.22) | 0.01(-0.28,0.30) |
| Austria | female | 3400.08(2440.49,4372.36) | 223.17(215.69,230.87) | 4316.44(3336.86,5376.38) | 312.84(303.45,322.48) | 0.65(0.35,0.96) | 0.94(0.70,1.17) |
| Azerbaijan | female | 3563.71(2335.65,4929.72) | 211.35(204.41,218.50) | 4135.43(2830.09,5713.56) | 194.59(188.59,200.76) | 0.78(0.62,0.94) | -0.20(-0.27,-0.14) |
| Bahamas | female | 103.79(73.35,144.39) | 171.43(139.90,208.41) | 133.60(92.99,179.66) | 169.34(141.84,200.70) | 0.62(0.46,0.79) | -0.13(-0.25,0.00) |
| Bahrain | female | 230.84(151.16,326.29) | 218.51(190.95,249.27) | 541.61(348.74,755.72) | 219.41(201.28,238.80) | 3.69(3.19,4.20) | 0.21(0.12,0.29) |
| Bangladesh | female | 18627.89(12998.41,25963.62) | 90.20(88.88,91.54) | 33903.39(23892.30,46444.87) | 93.75(92.75,94.76) | 2.02(1.89,2.15) | 0.27(0.22,0.32) |
| Barbados | female | 103.28(70.07,145.14) | 185.36(151.31,224.99) | 89.70(63.30,122.78) | 179.17(143.88,220.87) | -0.47(-0.52,-0.43) | -0.17(-0.29,-0.04) |
| Belarus | female | 5356.09(3941.93,7060.27) | 268.22(261.03,275.56) | 3568.20(2707.81,4521.77) | 247.15(238.78,255.77) | -1.35(-1.82,-0.88) | -0.52(-0.89,-0.15) |
| Belgium | female | 3281.83(2477.51,4139.56) | 178.76(172.66,185.04) | 4541.61(3565.08,5580.94) | 258.23(250.72,265.93) | 1.27(0.95,1.58) | 1.32(1.09,1.54) |
| Belize | female | 57.13(38.83,77.36) | 158.58(119.29,208.32) | 143.15(96.74,199.37) | 149.90(126.28,176.78) | 3.10(2.95,3.24) | -0.18(-0.28,-0.08) |
| Benin | female | 664.99(416.54,953.36) | 71.92(66.49,77.71) | 1985.16(1269.76,2790.39) | 75.06(71.75,78.50) | 3.64(3.55,3.72) | 0.21(0.15,0.28) |
| Bermuda | female | 25.92(18.61,35.14) | 193.00(124.94,290.09) | 17.11(12.18,23.15) | 192.29(110.87,314.70) | -1.61(-1.90,-1.32) | -0.29(-0.53,-0.04) |
| Bhutan | female | 108.01(70.09,149.43) | 90.50(73.85,110.16) | 152.18(102.58,215.93) | 91.40(77.45,107.20) | 1.56(1.38,1.74) | 0.04(0.00,0.09) |
| Bolivia (Plurinational State of) | female | 2521.72(1757.86,3327.35) | 201.00(193.17,209.09) | 4576.81(3328.83,5958.19) | 185.95(180.60,191.42) | 1.93(1.67,2.19) | -0.35(-0.48,-0.22) |
| Bosnia and Herzegovina | female | 741.35(488.65,1006.90) | 80.44(74.75,86.46) | 470.51(309.20,654.38) | 94.51(86.10,103.58) | -0.92(-1.19,-0.65) | 0.66(0.46,0.86) |
| Botswana | female | 423.42(290.53,577.36) | 159.66(144.56,176.04) | 802.64(549.46,1110.93) | 148.87(138.73,159.57) | 2.36(2.17,2.55) | -0.02(-0.15,0.11) |
| Brazil | female | 63415.53(43106.52,84403.41) | 198.93(197.38,200.49) | 93291.70(67006.58,119471.57) | 216.23(214.84,217.63) | 1.39(1.20,1.58) | 0.34(0.26,0.42) |
| Brunei Darussalam | female | 118.47(85.50,152.89) | 202.69(167.72,243.15) | 168.19(115.96,224.28) | 176.28(150.47,205.60) | 1.19(1.02,1.36) | -0.38(-0.43,-0.34) |
| Bulgaria | female | 1708.41(1211.97,2261.71) | 117.64(112.12,123.38) | 1251.89(904.43,1664.91) | 141.63(133.70,149.94) | -0.93(-1.25,-0.60) | 0.62(0.51,0.73) |
| Burkina Faso | female | 1107.92(698.50,1565.96) | 66.19(62.31,70.27) | 3060.22(1987.67,4310.50) | 68.30(65.87,70.79) | 3.47(3.38,3.57) | 0.16(0.10,0.23) |
| Burundi | female | 780.61(522.68,1029.71) | 74.17(69.01,79.65) | 1797.93(1264.09,2467.40) | 70.09(66.86,73.45) | 3.12(2.79,3.45) | -0.23(-0.26,-0.20) |
| Cabo Verde | female | 56.22(36.76,80.37) | 83.58(62.73,110.06) | 101.84(63.63,140.98) | 83.31(67.91,101.25) | 2.12(2.00,2.24) | 0.02(-0.02,0.06) |
| Cambodia | female | 2041.94(1313.42,2898.47) | 96.64(92.47,100.97) | 3402.49(2243.24,4812.69) | 93.84(90.71,97.05) | 1.92(1.82,2.01) | -0.08(-0.09,-0.06) |
| Cameroon | female | 1620.57(1006.41,2384.98) | 83.13(79.06,87.37) | 5187.43(3208.82,7550.42) | 81.05(78.84,83.31) | 3.99(3.87,4.11) | -0.01(-0.06,0.04) |
| Canada | female | 37813.14(26485.59,49655.88) | 666.78(660.00,673.61) | 67300.58(56072.28,80365.46) | 1117.76(1109.25,1126.33) | 1.85(1.37,2.34) | 1.65(1.29,2.02) |
| Central African Republic | female | 408.84(277.52,571.85) | 77.38(69.95,85.47) | 851.02(565.01,1170.80) | 76.66(71.54,82.07) | 2.47(2.32,2.62) | 0.03(-0.02,0.08) |
| Chad | female | 799.47(488.49,1137.61) | 74.73(69.59,80.18) | 2204.70(1389.97,3057.57) | 71.03(68.03,74.13) | 3.36(3.31,3.41) | -0.12(-0.16,-0.09) |
| Chile | female | 5136.52(3453.79,6947.20) | 175.33(170.56,180.21) | 7109.72(4975.54,9445.10) | 202.46(197.75,207.26) | 1.00(0.86,1.13) | 0.40(0.25,0.55) |
| China | female | 1207313.64(913417.22,1501345.19) | 451.67(450.86,452.48) | 395584.00(275038.78,520476.84) | 179.55(178.97,180.13) | -4.41(-4.71,-4.11) | -3.96(-4.38,-3.52) |
| Colombia | female | 13198.18(9345.65,17886.65) | 180.94(177.85,184.08) | 18238.09(12675.62,24580.28) | 180.73(178.11,183.38) | 0.97(0.85,1.09) | 0.05(-0.03,0.14) |
| Comoros | female | 58.06(39.68,80.18) | 69.18(52.24,90.35) | 112.84(78.29,157.96) | 73.77(60.76,88.84) | 2.08(1.98,2.17) | 0.18(0.12,0.25) |
| Congo | female | 359.18(239.84,499.39) | 77.98(69.97,86.73) | 934.41(634.45,1266.59) | 83.95(78.65,89.53) | 3.27(3.08,3.47) | 0.34(0.28,0.39) |
| Cook Islands | female | 4.20(2.72,5.87) | 112.38(31.45,294.79) | 3.57(2.35,5.03) | 113.18(27.75,310.44) | -0.73(-0.86,-0.60) | 0.04(0.00,0.08) |
| Costa Rica | female | 971.06(648.15,1334.01) | 148.74(139.48,158.48) | 1577.68(1109.74,2139.81) | 158.34(150.59,166.41) | 1.67(1.43,1.91) | 0.26(0.15,0.38) |
| Croatia | female | 1288.27(948.58,1689.73) | 145.25(137.39,153.47) | 1095.60(783.27,1411.94) | 179.14(168.56,190.25) | 0.61(0.14,1.07) | 1.74(1.29,2.20) |
| Cuba | female | 4299.21(2884.98,6104.47) | 176.19(170.91,181.60) | 2737.60(1810.77,3806.41) | 154.71(148.93,160.68) | -1.89(-2.05,-1.73) | -0.50(-0.54,-0.46) |
| Cyprus | female | 224.55(162.23,290.03) | 148.50(129.69,169.37) | 372.26(262.11,487.23) | 147.54(132.33,164.31) | 1.73(1.61,1.85) | -0.29(-0.48,-0.10) |
| Czechia | female | 2605.92(1848.97,3435.98) | 147.57(141.93,153.39) | 2637.90(1913.71,3415.60) | 190.16(182.81,197.75) | 0.56(0.18,0.95) | 1.34(1.05,1.63) |
| C?te d'Ivoire | female | 1694.47(1090.48,2428.30) | 73.60(70.06,77.29) | 3980.12(2533.41,5615.37) | 72.99(70.74,75.31) | 2.75(2.54,2.97) | 0.01(-0.06,0.07) |
| Democratic People's Republic of Korea | female | 9929.23(7243.52,13075.38) | 221.15(216.78,225.59) | 9154.37(6366.17,12134.00) | 187.76(183.92,191.66) | -0.52(-0.65,-0.38) | -0.62(-0.71,-0.52) |
| Democratic Republic of the Congo | female | 4956.27(3394.12,6877.07) | 70.85(68.86,72.88) | 13488.62(8896.16,18507.22) | 78.27(76.94,79.62) | 3.41(3.31,3.50) | 0.42(0.37,0.47) |
| Denmark | female | 2958.52(2332.62,3605.39) | 312.53(301.35,324.04) | 3340.30(2666.50,4094.88) | 366.70(354.33,379.41) | 0.20(-0.16,0.55) | 0.49(0.24,0.75) |
| Djibouti | female | 51.56(32.45,70.92) | 65.24(48.28,86.90) | 179.09(123.40,249.22) | 70.73(60.73,81.95) | 4.25(3.94,4.57) | 0.31(0.22,0.40) |
| Dominica | female | 23.32(16.01,32.07) | 166.61(105.13,254.20) | 22.86(16.99,29.97) | 182.04(115.19,273.95) | -0.15(-0.23,-0.07) | 0.20(0.06,0.34) |
| Dominican Republic | female | 1917.28(1288.10,2578.14) | 121.50(116.04,127.18) | 3148.29(2140.24,4298.91) | 138.47(133.67,143.40) | 1.60(1.53,1.67) | 0.49(0.42,0.55) |
| Ecuador | female | 3192.63(2164.16,4295.62) | 153.41(148.09,158.90) | 6084.89(4299.02,7946.96) | 165.30(161.17,169.51) | 2.18(1.93,2.44) | 0.36(0.15,0.57) |
| Egypt | female | 20815.39(13321.24,28376.33) | 196.37(193.69,199.07) | 40548.29(25915.17,56677.74) | 197.27(195.36,199.20) | 2.33(2.14,2.52) | 0.05(0.01,0.09) |
| El Salvador | female | 1459.96(964.95,2035.55) | 137.63(130.54,145.03) | 1961.81(1307.70,2667.03) | 141.42(135.21,147.85) | 0.88(0.77,0.99) | 0.10(0.02,0.17) |
| Equatorial Guinea | female | 61.49(42.60,87.92) | 77.48(59.15,99.96) | 261.03(170.16,361.91) | 87.33(77.00,98.72) | 5.03(4.82,5.25) | 0.51(0.41,0.61) |
| Eritrea | female | 457.45(300.10,626.60) | 72.30(65.74,79.37) | 983.84(685.25,1349.17) | 74.35(69.75,79.18) | 2.92(2.76,3.08) | 0.08(0.06,0.10) |
| Estonia | female | 996.86(743.86,1257.23) | 356.00(334.10,379.02) | 1270.91(939.11,1598.36) | 686.38(648.25,726.31) | 1.00(0.82,1.18) | 2.09(1.89,2.29) |
| Eswatini | female | 264.34(187.46,353.73) | 165.03(145.30,186.97) | 414.61(292.61,548.48) | 158.34(143.43,174.43) | 1.46(1.22,1.70) | -0.05(-0.16,0.06) |
| Ethiopia | female | 6784.91(4958.26,9022.33) | 73.74(71.97,75.54) | 14251.87(10039.20,19426.04) | 63.47(62.42,64.54) | 2.24(2.16,2.31) | -0.66(-0.73,-0.59) |
| Fiji | female | 197.68(134.58,272.31) | 123.01(106.40,141.61) | 188.04(127.21,267.87) | 108.13(93.22,124.75) | -0.26(-0.41,-0.10) | -0.61(-0.72,-0.50) |
| Finland | female | 2401.85(1847.26,2935.27) | 264.93(254.33,275.90) | 4167.86(3458.87,4925.28) | 510.42(494.91,526.31) | 2.21(1.95,2.46) | 2.44(2.28,2.60) |
| France | female | 21016.57(16107.77,26609.17) | 192.60(190.00,195.23) | 25372.47(20148.85,31423.40) | 255.57(252.42,258.75) | 0.96(0.70,1.23) | 1.26(0.99,1.53) |
| Gabon | female | 163.32(106.78,227.23) | 88.26(75.00,103.39) | 334.21(223.84,464.37) | 85.01(76.08,94.75) | 2.30(2.27,2.34) | -0.11(-0.13,-0.08) |
| Gambia | female | 160.23(98.31,229.04) | 82.93(70.34,97.42) | 383.26(253.13,538.38) | 75.89(68.39,84.04) | 2.72(2.66,2.78) | -0.30(-0.33,-0.27) |
| Georgia | female | 2628.57(1746.99,3666.19) | 236.47(227.48,245.73) | 1213.69(799.69,1665.78) | 218.62(206.27,231.57) | -2.13(-2.51,-1.75) | 0.04(-0.34,0.43) |
| Germany | female | 34164.87(26322.92,41843.60) | 228.86(226.40,231.34) | 30772.19(23626.97,38019.51) | 251.65(248.80,254.53) | -1.00(-1.20,-0.80) | -0.33(-0.52,-0.14) |
| Ghana | female | 2623.00(1721.65,3677.79) | 90.05(86.60,93.61) | 6788.43(4372.48,9595.39) | 91.26(89.10,93.47) | 3.25(3.19,3.31) | 0.05(0.00,0.09) |
| Greece | female | 2697.80(1960.54,3439.80) | 143.52(138.16,149.05) | 3253.06(2450.32,4092.25) | 234.56(226.46,242.89) | 0.88(0.53,1.23) | 1.95(1.81,2.08) |
| Greenland | female | 68.21(50.95,84.40) | 533.81(412.02,686.27) | 58.54(45.62,71.59) | 574.31(436.04,745.32) | -0.43(-0.56,-0.30) | 0.35(0.19,0.50) |
| Grenada | female | 27.79(19.95,37.03) | 170.31(112.59,249.23) | 32.84(23.49,43.33) | 167.33(114.88,236.45) | 0.32(-0.24,0.88) | -0.20(-0.40,0.00) |
| Guam | female | 35.83(24.35,48.69) | 122.56(85.70,170.36) | 30.62(20.09,42.82) | 114.99(77.91,163.83) | -0.76(-0.92,-0.60) | -0.28(-0.35,-0.21) |
| Guatemala | female | 2405.63(1818.50,3097.69) | 160.69(154.24,167.35) | 6499.50(4996.69,8236.55) | 182.14(177.71,186.66) | 3.75(3.46,4.03) | 0.70(0.43,0.98) |
| Guinea | female | 747.48(475.22,1032.81) | 67.70(62.91,72.77) | 1783.35(1167.91,2492.13) | 65.45(62.43,68.59) | 2.79(2.71,2.87) | -0.14(-0.17,-0.11) |
| Guinea-Bissau | female | 129.26(82.51,192.48) | 68.24(56.87,81.38) | 289.35(191.09,402.65) | 66.88(59.36,75.13) | 2.84(2.76,2.93) | -0.05(-0.08,-0.01) |
| Guyana | female | 235.07(157.38,317.66) | 137.34(120.15,156.49) | 234.46(165.68,315.05) | 146.40(128.13,166.67) | -0.39(-0.57,-0.21) | 0.05(-0.05,0.15) |
| Haiti | female | 1861.62(1321.12,2471.43) | 146.41(139.80,153.27) | 4285.38(3094.95,5679.98) | 152.40(147.87,157.04) | 2.89(2.69,3.10) | 0.10(0.04,0.17) |
| Honduras | female | 1324.90(945.14,1757.55) | 152.81(144.57,161.43) | 3214.19(2255.64,4421.63) | 139.98(135.16,144.94) | 2.90(2.80,3.01) | -0.40(-0.46,-0.34) |
| Hungary | female | 2193.24(1521.57,3004.31) | 124.78(119.56,130.18) | 1825.75(1229.28,2502.63) | 136.50(130.24,143.00) | -0.37(-0.68,-0.06) | 0.50(0.31,0.68) |
| Iceland | female | 184.30(143.41,222.44) | 357.92(308.06,413.76) | 440.58(352.44,523.94) | 742.10(674.22,815.38) | 3.19(3.02,3.36) | 2.65(2.51,2.78) |
| India | female | 154581.63(116922.48,204047.26) | 94.19(93.72,94.66) | 316259.19(227190.91,425465.40) | 106.73(106.36,107.11) | 1.50(1.01,1.99) | -0.46(-0.97,0.05) |
| Indonesia | female | 30774.62(20151.11,44166.45) | 76.33(75.48,77.20) | 41704.99(27398.82,60036.28) | 74.60(73.89,75.32) | 0.73(0.61,0.86) | -0.20(-0.28,-0.12) |
| Iran (Islamic Republic of) | female | 37256.91(28293.42,46058.93) | 353.85(350.21,357.53) | 53473.34(41536.57,66437.64) | 320.33(317.53,323.14) | 1.44(0.75,2.14) | -0.40(-0.69,-0.11) |
| Iraq | female | 7411.98(5202.58,10044.56) | 220.63(215.55,225.82) | 17608.65(12921.22,23902.51) | 211.51(208.38,214.67) | 2.73(2.43,3.04) | -0.19(-0.36,-0.03) |
| Ireland | female | 1564.06(1203.73,1982.69) | 231.81(220.45,243.63) | 3559.95(2803.10,4312.66) | 451.40(436.52,466.67) | 2.59(2.24,2.95) | 1.75(1.45,2.05) |
| Israel | female | 1597.86(1150.74,2056.99) | 169.89(161.65,178.45) | 3241.72(2396.75,4138.27) | 199.13(192.33,206.11) | 2.50(2.29,2.70) | 0.70(0.61,0.80) |
| Italy | female | 29846.49(21921.23,37756.90) | 278.60(275.44,281.78) | 14842.53(10382.91,19214.96) | 194.42(191.28,197.60) | -2.82(-3.22,-2.41) | -1.69(-2.03,-1.35) |
| Jamaica | female | 834.75(571.66,1146.00) | 164.93(153.78,176.75) | 1010.28(709.16,1373.59) | 166.28(156.16,176.92) | 0.51(0.37,0.65) | -0.06(-0.16,0.05) |
| Japan | female | 34591.70(24084.95,46440.25) | 159.47(157.79,161.16) | 26387.66(19289.51,34678.34) | 165.42(163.41,167.45) | -0.99(-1.33,-0.64) | 0.16(-0.02,0.34) |
| Jordan | female | 1517.37(997.96,2114.13) | 216.87(205.70,228.57) | 5220.09(3271.08,7423.49) | 216.47(210.62,222.45) | 4.19(4.05,4.33) | 0.13(0.06,0.20) |
| Kazakhstan | female | 9808.76(7002.79,12864.30) | 287.25(281.58,293.01) | 11656.06(8502.84,14920.09) | 330.09(324.03,336.23) | 0.78(0.37,1.20) | 0.26(-0.26,0.78) |
| Kenya | female | 2520.16(1739.50,3407.57) | 60.40(57.99,62.90) | 5708.09(4051.23,7565.80) | 53.50(52.11,54.92) | 2.74(2.46,3.01) | -0.33(-0.49,-0.16) |
| Kiribati | female | 31.36(24.28,39.08) | 197.24(133.88,282.49) | 65.45(49.50,85.11) | 256.87(198.40,327.54) | 3.02(2.72,3.32) | 1.21(1.01,1.40) |
| Kuwait | female | 837.46(550.84,1177.60) | 227.52(212.31,243.60) | 2261.06(1435.80,3168.62) | 213.95(204.68,223.61) | 4.34(3.84,4.83) | -0.04(-0.13,0.05) |
| Kyrgyzstan | female | 2200.13(1559.16,2910.01) | 242.70(232.62,253.13) | 3252.92(2339.10,4295.96) | 235.95(227.89,244.24) | 1.62(1.31,1.94) | 0.11(-0.20,0.42) |
| Lao People's Democratic Republic | female | 888.94(561.23,1299.02) | 110.43(103.22,118.06) | 1762.52(1100.20,2572.41) | 109.25(104.21,114.48) | 2.30(2.02,2.58) | -0.09(-0.29,0.11) |
| Latvia | female | 1379.00(988.66,1793.96) | 288.30(273.21,304.04) | 638.19(456.16,818.81) | 248.84(229.34,269.67) | -3.12(-3.52,-2.71) | -1.29(-1.61,-0.96) |
| Lebanon | female | 1366.17(917.91,1846.68) | 226.24(214.33,238.67) | 2567.96(1753.62,3486.53) | 227.49(218.55,236.73) | 2.67(2.40,2.94) | 0.14(0.07,0.20) |
| Lesotho | female | 440.37(291.72,606.68) | 148.87(135.20,163.60) | 653.25(459.07,871.12) | 157.17(145.26,169.84) | 1.51(1.41,1.62) | 0.38(0.29,0.47) |
| Liberia | female | 369.40(231.56,525.67) | 78.53(70.65,87.10) | 869.88(568.70,1208.76) | 79.24(74.03,84.74) | 3.99(3.62,4.36) | 0.03(-0.04,0.09) |
| Libya | female | 1686.90(1174.30,2311.90) | 224.13(213.21,235.52) | 4046.35(2911.61,5255.61) | 276.11(267.64,284.78) | 3.15(2.74,3.55) | 0.93(0.80,1.05) |
| Lithuania | female | 1518.48(1153.70,1932.10) | 216.04(205.28,227.24) | 922.52(686.83,1154.48) | 227.40(212.80,242.85) | -2.38(-2.64,-2.11) | -0.48(-0.75,-0.21) |
| Luxembourg | female | 206.55(154.53,262.57) | 275.16(238.40,316.77) | 400.07(311.13,492.25) | 365.07(329.47,403.92) | 1.16(0.83,1.48) | -0.06(-0.47,0.35) |
| Madagascar | female | 1649.37(1201.05,2243.83) | 75.08(71.45,78.87) | 4338.68(3044.49,5830.51) | 75.09(72.84,77.39) | 3.11(3.06,3.16) | -0.03(-0.07,0.02) |
| Malawi | female | 1386.64(947.83,1876.32) | 74.81(70.83,78.97) | 2936.74(2079.39,4148.48) | 73.15(70.48,75.90) | 2.45(2.29,2.61) | -0.07(-0.09,-0.04) |
| Malaysia | female | 3694.57(2393.10,5253.56) | 99.04(95.87,102.30) | 6714.91(4254.67,9716.12) | 99.69(97.32,102.11) | 2.04(1.89,2.19) | 0.06(0.04,0.08) |
| Maldives | female | 39.28(24.74,56.28) | 94.53(66.49,132.77) | 84.93(55.44,114.42) | 96.55(76.73,120.39) | 2.94(2.65,3.24) | 0.11(0.08,0.14) |
| Mali | female | 1114.18(703.69,1594.82) | 71.67(67.49,76.07) | 3095.29(1923.91,4450.14) | 70.01(67.51,72.58) | 3.52(3.39,3.65) | -0.08(-0.11,-0.04) |
| Malta | female | 130.92(90.76,175.26) | 193.79(161.76,230.61) | 151.43(107.90,195.74) | 229.58(193.75,270.93) | 0.99(-0.08,2.08) | 0.82(-0.16,1.81) |
| Marshall Islands | female | 9.83(6.52,13.61) | 118.50(55.84,225.95) | 13.63(9.35,19.25) | 119.92(64.88,203.58) | 0.89(0.66,1.12) | -0.03(-0.05,0.00) |
| Mauritania | female | 311.04(202.22,444.82) | 81.11(72.26,90.81) | 688.09(427.88,976.10) | 80.42(74.46,86.77) | 2.58(2.54,2.61) | 0.02(-0.02,0.06) |
| Mauritius | female | 350.02(221.60,479.15) | 140.16(125.83,155.71) | 349.07(238.32,473.77) | 152.78(137.15,169.76) | 0.16(-0.02,0.35) | 0.50(0.38,0.62) |
| Mexico | female | 31099.22(22339.84,40686.92) | 171.45(169.52,173.40) | 41840.89(29634.46,55059.19) | 159.18(157.65,160.71) | 0.99(0.84,1.14) | -0.09(-0.16,-0.03) |
| Micronesia (Federated States of) | female | 23.89(15.98,33.09) | 124.00(78.97,187.23) | 24.91(16.34,34.46) | 121.22(78.16,180.70) | -0.12(-0.24,-0.01) | -0.12(-0.14,-0.10) |
| Monaco | female | 7.44(4.94,10.02) | 158.69(64.64,348.03) | 8.32(5.69,11.27) | 179.55(78.52,356.81) | 0.44(0.39,0.49) | 0.49(0.43,0.56) |
| Mongolia | female | 907.10(604.64,1260.94) | 204.69(191.33,218.86) | 1364.75(927.60,1851.06) | 214.78(203.42,226.64) | 1.59(1.29,1.90) | 0.35(0.19,0.51) |
| Montenegro | female | 115.62(78.45,153.02) | 95.38(78.79,114.47) | 96.46(63.91,129.96) | 96.35(77.99,117.93) | -0.39(-0.51,-0.27) | 0.16(0.09,0.22) |
| Morocco | female | 13410.27(9358.60,18261.30) | 256.07(251.72,260.49) | 17868.50(12757.80,23697.45) | 244.16(240.59,247.77) | 0.91(0.61,1.21) | -0.18(-0.33,-0.02) |
| Mozambique | female | 1663.82(1136.74,2356.74) | 66.97(63.77,70.31) | 4218.20(2921.90,5823.13) | 68.99(66.88,71.16) | 2.97(2.83,3.11) | 0.15(0.11,0.19) |
| Myanmar | female | 9162.92(6074.66,13103.71) | 103.55(101.42,105.71) | 11032.57(7119.59,15877.44) | 95.79(94.01,97.59) | 0.32(0.18,0.46) | -0.38(-0.43,-0.33) |
| Namibia | female | 469.01(326.29,654.63) | 168.16(152.98,184.58) | 835.78(582.88,1124.63) | 156.54(146.08,167.58) | 1.83(1.67,1.99) | -0.19(-0.24,-0.13) |
| Nauru | female | 2.44(1.67,3.39) | 121.84(19.49,413.33) | 2.77(1.88,3.76) | 120.70(22.61,379.78) | 0.28(0.20,0.36) | -0.04(-0.06,-0.02) |
| Nepal | female | 3696.60(2495.26,5213.73) | 98.95(95.76,102.23) | 6923.49(4696.26,9741.79) | 94.62(92.39,96.88) | 2.07(1.99,2.15) | -0.17(-0.22,-0.13) |
| Netherlands | female | 5097.48(3661.21,6760.14) | 172.67(167.94,177.50) | 5347.30(3943.60,6851.92) | 204.35(198.89,209.92) | -0.53(-0.96,-0.11) | -0.02(-0.37,0.32) |
| New Zealand | female | 2091.50(1479.37,2767.91) | 301.27(288.49,314.48) | 3590.63(2764.81,4447.15) | 403.91(390.73,417.45) | 1.41(1.25,1.56) | 0.73(0.62,0.84) |
| Nicaragua | female | 1124.91(730.81,1532.67) | 151.40(142.52,160.74) | 1932.56(1286.15,2715.97) | 134.74(128.80,140.89) | 1.75(1.57,1.93) | -0.39(-0.44,-0.35) |
| Niger | female | 1041.55(635.63,1547.79) | 74.38(69.85,79.15) | 3192.47(2043.54,4581.79) | 75.41(72.73,78.18) | 3.65(3.57,3.72) | 0.16(0.07,0.25) |
| Nigeria | female | 10622.60(7179.21,14677.49) | 64.49(63.24,65.76) | 27451.14(18338.85,37597.20) | 60.23(59.50,60.96) | 3.03(2.92,3.13) | -0.19(-0.24,-0.14) |
| Niue | female | 0.44(0.30,0.61) | 117.37(0.05,1267.03) | 0.32(0.22,0.44) | 117.75(0.00,1617.96) | -1.37(-1.72,-1.01) | -0.03(-0.06,0.00) |
| North Macedonia | female | 399.24(276.80,535.22) | 102.37(92.57,112.93) | 413.07(283.48,560.13) | 110.33(99.78,121.81) | 1.18(0.84,1.53) | 1.06(0.81,1.31) |
| Northern Mariana Islands | female | 13.97(9.13,19.87) | 114.74(62.50,195.88) | 8.86(6.05,12.32) | 115.16(52.04,223.46) | -2.85(-3.98,-1.71) | 0.01(-0.10,0.12) |
| Norway | female | 2499.74(1888.70,3128.03) | 316.49(304.19,329.17) | 4648.53(3716.03,5645.91) | 532.65(517.36,548.31) | 1.00(0.29,1.70) | 0.68(-0.12,1.49) |
| Oman | female | 582.58(376.56,831.59) | 206.04(189.58,223.63) | 1671.13(1060.79,2363.59) | 208.39(198.32,218.87) | 3.83(3.57,4.09) | 0.13(0.04,0.22) |
| Pakistan | female | 27195.67(18524.77,36866.11) | 145.61(143.85,147.38) | 61676.82(44098.83,84767.02) | 125.66(124.67,126.66) | 3.30(2.48,4.12) | 0.00(-0.75,0.75) |
| Palau | female | 3.95(2.70,5.59) | 118.85(32.00,312.02) | 2.77(1.84,3.88) | 119.55(22.23,377.12) | -1.55(-1.97,-1.13) | 0.01(0.00,0.03) |
| Palestine | female | 810.84(501.31,1157.53) | 217.57(202.44,233.69) | 2210.81(1379.70,3250.71) | 204.99(196.47,213.82) | 3.52(3.33,3.72) | -0.12(-0.19,-0.05) |
| Panama | female | 804.85(557.55,1085.19) | 160.18(149.21,171.78) | 1311.90(933.94,1750.99) | 160.75(152.17,169.71) | 1.55(1.47,1.62) | 0.11(0.05,0.18) |
| Papua New Guinea | female | 848.96(569.61,1204.07) | 104.49(97.50,111.89) | 2252.63(1458.65,3114.81) | 106.37(102.02,110.86) | 3.27(3.21,3.33) | 0.07(0.04,0.09) |
| Paraguay | female | 1214.67(809.13,1679.75) | 158.34(149.50,167.58) | 2409.83(1674.87,3347.79) | 159.57(153.26,166.07) | 2.39(2.29,2.49) | 0.04(0.01,0.08) |
| Peru | female | 7599.24(5380.75,9999.56) | 168.65(164.84,172.54) | 12872.53(9293.45,16807.13) | 173.19(170.20,176.22) | 1.68(1.54,1.82) | 0.14(-0.01,0.29) |
| Philippines | female | 12040.89(8163.44,16756.61) | 93.41(91.74,95.12) | 21243.41(14137.94,29213.47) | 91.13(89.91,92.37) | 1.87(1.81,1.93) | -0.03(-0.07,0.01) |
| Poland | female | 9401.36(6815.43,12455.83) | 137.00(134.21,139.84) | 7573.69(5427.46,10005.78) | 132.23(129.18,135.34) | -0.87(-0.96,-0.78) | -0.55(-0.84,-0.26) |
| Portugal | female | 3133.55(2170.22,4036.83) | 165.67(159.92,171.57) | 2823.30(2058.07,3619.70) | 190.20(183.19,197.43) | -0.49(-0.66,-0.32) | 0.40(0.32,0.49) |
| Puerto Rico | female | 1416.30(1045.27,1856.62) | 194.98(184.95,205.40) | 1070.96(802.01,1371.63) | 202.13(190.18,214.66) | -1.59(-2.19,-1.00) | -0.57(-1.02,-0.12) |
| Qatar | female | 139.06(91.86,198.44) | 211.35(177.21,250.58) | 942.99(632.97,1299.60) | 218.57(203.66,234.47) | 7.57(7.00,8.13) | 0.29(0.19,0.39) |
| Republic of Korea | female | 17998.57(12419.03,24632.20) | 172.94(170.41,175.49) | 14517.72(10077.49,19359.91) | 185.89(182.83,189.00) | -0.83(-0.98,-0.68) | 0.15(0.04,0.25) |
| Republic of Moldova | female | 1913.42(1337.43,2505.69) | 216.47(206.79,226.51) | 1196.01(839.53,1575.85) | 197.02(185.54,209.10) | -1.35(-1.57,-1.12) | -0.47(-0.60,-0.35) |
| Romania | female | 4792.27(3826.66,5965.86) | 112.84(109.63,116.11) | 2420.66(1848.90,3085.35) | 92.75(89.05,96.58) | -2.73(-2.94,-2.53) | -1.09(-1.38,-0.81) |
| Russian Federation | female | 121136.12(90983.75,151674.57) | 424.40(421.98,426.83) | 103080.28(81363.38,125769.54) | 444.68(441.85,447.52) | -0.74(-1.68,0.20) | -0.43(-1.23,0.38) |
| Rwanda | female | 1060.16(754.96,1421.94) | 79.16(74.42,84.14) | 2022.92(1378.44,2821.45) | 71.72(68.62,74.94) | 2.52(2.22,2.82) | -0.42(-0.48,-0.36) |
| Saint Kitts and Nevis | female | 14.86(10.38,20.17) | 172.32(95.84,289.66) | 18.87(13.10,25.41) | 164.90(98.85,260.81) | 0.68(0.51,0.84) | -0.37(-0.50,-0.24) |
| Saint Lucia | female | 50.08(34.02,69.45) | 172.69(127.52,230.51) | 52.62(35.61,72.17) | 160.18(119.68,210.79) | 0.29(0.06,0.52) | -0.08(-0.17,0.01) |
| Saint Vincent and the Grenadines | female | 33.50(22.13,46.70) | 150.28(102.98,214.53) | 29.69(20.61,41.54) | 146.06(98.30,209.26) | -0.47(-0.56,-0.37) | -0.10(-0.17,-0.03) |
| Samoa | female | 35.40(23.81,48.59) | 115.15(79.72,162.65) | 47.30(31.59,63.85) | 120.44(88.44,160.96) | 0.79(0.71,0.87) | 0.13(0.08,0.18) |
| San Marino | female | 7.67(5.34,10.40) | 158.67(66.84,321.99) | 7.86(5.26,10.64) | 173.93(74.17,349.63) | -0.16(-0.39,0.08) | 0.41(0.33,0.49) |
| Sao Tome and Principe | female | 17.89(11.45,25.77) | 86.77(50.62,141.63) | 34.60(21.71,50.13) | 79.03(54.81,110.67) | 2.12(2.07,2.18) | -0.37(-0.41,-0.34) |
| Saudi Arabia | female | 5767.00(3574.27,8124.62) | 211.17(205.69,216.78) | 17233.06(11174.08,24715.36) | 220.89(217.55,224.28) | 4.12(3.88,4.36) | 0.28(0.22,0.34) |
| Senegal | female | 1146.78(732.12,1639.61) | 82.30(77.52,87.32) | 2403.59(1496.81,3515.99) | 76.29(73.25,79.44) | 2.32(2.19,2.45) | -0.28(-0.32,-0.23) |
| Serbia | female | 1628.35(1176.49,2115.74) | 93.27(88.79,97.93) | 1285.55(927.26,1712.91) | 91.10(86.15,96.28) | -0.76(-0.85,-0.66) | 0.06(0.01,0.12) |
| Seychelles | female | 22.60(15.59,30.58) | 143.90(90.17,222.74) | 22.93(16.70,31.11) | 133.39(84.41,201.09) | 0.18(0.08,0.29) | 0.00(-0.07,0.08) |
| Sierra Leone | female | 730.07(458.27,1030.52) | 85.13(79.01,91.65) | 1490.74(974.93,2123.96) | 79.15(75.13,83.35) | 2.67(2.43,2.91) | -0.26(-0.29,-0.23) |
| Singapore | female | 1288.53(888.12,1743.56) | 169.30(160.13,178.90) | 1685.09(1137.56,2268.45) | 173.15(164.39,182.35) | 1.24(0.88,1.61) | 0.04(-0.06,0.13) |
| Slovakia | female | 1442.53(1034.45,1934.54) | 145.84(138.37,153.61) | 1194.38(842.02,1566.48) | 144.75(136.45,153.47) | -0.36(-0.85,0.14) | 0.07(-0.22,0.35) |
| Slovenia | female | 451.71(334.73,580.35) | 121.16(110.20,132.97) | 454.51(319.93,595.43) | 170.13(154.53,186.98) | 1.57(0.73,2.41) | 2.54(1.84,3.25) |
| Solomon Islands | female | 70.06(46.13,95.98) | 111.99(86.68,143.33) | 156.97(105.88,213.41) | 115.68(98.23,135.44) | 2.47(2.32,2.62) | 0.07(0.05,0.09) |
| Somalia | female | 936.33(629.31,1307.20) | 70.43(65.97,75.11) | 2602.53(1762.96,3512.29) | 69.04(66.36,71.81) | 3.80(3.58,4.02) | -0.08(-0.10,-0.06) |
| South Africa | female | 30784.56(23279.58,38120.60) | 391.10(386.71,395.54) | 28928.48(22509.58,36108.82) | 235.56(232.84,238.31) | -0.57(-1.40,0.27) | -1.95(-2.69,-1.20) |
| South Sudan | female | 760.54(508.09,1065.77) | 71.64(66.51,77.10) | 1231.28(841.51,1681.61) | 71.46(67.44,75.66) | 1.75(1.40,2.10) | -0.02(-0.06,0.01) |
| Spain | female | 20330.72(15458.44,25498.22) | 275.22(271.45,279.03) | 16864.89(12504.47,21984.00) | 281.82(277.52,286.16) | -2.03(-2.92,-1.13) | -1.41(-2.12,-0.70) |
| Sri Lanka | female | 5150.14(3681.49,6683.51) | 138.33(134.57,142.17) | 4836.86(3304.85,6510.42) | 118.66(115.34,122.06) | -0.43(-0.54,-0.31) | -0.69(-0.81,-0.56) |
| Sudan | female | 8576.14(5970.64,11716.65) | 220.76(216.05,225.55) | 21723.18(15886.07,28604.06) | 235.97(232.83,239.15) | 3.05(2.97,3.14) | 0.25(0.19,0.31) |
| Suriname | female | 114.46(79.51,156.54) | 144.98(119.31,175.03) | 164.07(117.38,223.96) | 151.20(128.94,176.26) | 1.32(1.10,1.54) | 0.15(0.10,0.20) |
| Sweden | female | 1994.51(1514.06,2529.34) | 137.12(131.16,143.30) | 5989.02(4734.59,7264.72) | 376.37(366.78,386.16) | 4.28(3.99,4.57) | 3.94(3.73,4.15) |
| Switzerland | female | 8208.33(6319.33,10280.49) | 627.28(613.65,641.16) | 4897.21(3862.05,6060.86) | 356.39(346.27,366.77) | -2.13(-2.37,-1.89) | -2.38(-2.58,-2.17) |
| Syrian Arab Republic | female | 5814.94(4272.25,7805.61) | 249.36(242.83,256.04) | 7218.33(5201.13,9430.75) | 270.42(263.73,277.26) | 0.82(-0.14,1.79) | 0.36(0.29,0.42) |
| Taiwan (Province of China) | female | 10853.43(8361.05,14057.78) | 236.95(232.51,241.46) | 6595.50(4664.13,8731.05) | 174.77(170.51,179.13) | -1.86(-2.18,-1.53) | -1.30(-1.75,-0.85) |
| Tajikistan | female | 2235.29(1511.81,3114.44) | 209.72(200.99,218.79) | 4069.45(2731.16,5567.69) | 194.35(188.42,200.43) | 2.22(2.08,2.35) | -0.21(-0.26,-0.16) |
| Thailand | female | 14519.58(9474.51,20012.71) | 109.86(108.08,111.67) | 12970.61(8780.56,17651.45) | 118.65(116.60,120.72) | -0.58(-0.93,-0.23) | 0.22(0.01,0.44) |
| Timor-Leste | female | 153.71(99.17,219.81) | 96.09(81.40,112.89) | 287.65(184.91,419.45) | 98.86(87.52,111.45) | 2.07(1.81,2.33) | 0.12(0.09,0.15) |
| Togo | female | 463.45(289.18,657.40) | 66.37(60.38,72.84) | 1189.22(774.96,1714.56) | 69.50(65.60,73.57) | 3.28(3.14,3.42) | 0.30(0.23,0.36) |
| Tokelau | female | 0.33(0.22,0.46) | 116.51(0.00,1731.70) | 0.28(0.18,0.39) | 116.35(0.00,1871.78) | -0.90(-1.31,-0.49) | -0.07(-0.09,-0.04) |
| Tonga | female | 20.62(13.28,28.84) | 114.16(69.47,179.83) | 23.83(15.92,33.20) | 120.29(76.74,180.51) | 0.31(0.20,0.43) | 0.09(0.04,0.13) |
| Trinidad and Tobago | female | 381.38(257.76,528.40) | 151.98(137.07,168.13) | 390.14(280.29,523.27) | 158.27(142.71,175.14) | 0.12(-0.14,0.38) | 0.04(-0.08,0.16) |
| Tunisia | female | 3610.62(2474.53,4962.51) | 211.05(204.15,218.14) | 4933.64(3375.10,6617.75) | 223.14(216.90,229.54) | 1.22(0.85,1.59) | 0.30(0.17,0.43) |
| Türkiye | female | 27135.66(17722.77,38848.32) | 233.16(230.38,235.97) | 35185.06(22689.14,50133.54) | 222.94(220.61,225.29) | 0.86(0.67,1.04) | -0.05(-0.10,0.01) |
| Turkmenistan | female | 1749.15(1184.51,2401.62) | 224.88(214.38,235.80) | 3311.06(2451.63,4179.55) | 338.58(327.14,350.32) | 2.43(2.28,2.59) | 1.67(1.52,1.81) |
| Tuvalu | female | 2.26(1.49,3.17) | 117.44(17.06,404.25) | 2.67(1.71,3.81) | 115.04(20.62,373.16) | 0.67(0.42,0.92) | -0.05(-0.07,-0.03) |
| Uganda | female | 2389.63(1551.90,3322.56) | 74.51(71.45,77.68) | 6481.62(4324.49,8995.70) | 76.24(74.35,78.16) | 3.21(3.10,3.32) | 0.00(-0.07,0.07) |
| Ukraine | female | 19344.59(13914.25,24816.92) | 201.42(198.58,204.30) | 16272.67(11881.43,20598.01) | 239.60(235.77,243.48) | -0.67(-0.94,-0.40) | 0.19(-0.02,0.40) |
| United Arab Emirates | female | 705.90(468.40,998.91) | 228.67(211.97,246.42) | 2380.61(1645.62,3182.84) | 235.43(225.14,246.11) | 5.45(4.36,6.55) | 0.16(0.12,0.21) |
| United Kingdom | female | 33935.87(25151.23,42416.50) | 320.05(316.64,323.49) | 72569.03(57793.99,86259.58) | 636.95(632.28,641.64) | 2.21(2.00,2.41) | 1.95(1.67,2.23) |
| United Republic of Tanzania | female | 4360.87(3025.76,6018.82) | 87.98(85.33,90.70) | 10498.96(6891.16,14501.05) | 88.22(86.52,89.95) | 2.44(2.27,2.60) | -0.35(-0.47,-0.23) |
| United States of America | female | 262453.40(190818.36,333729.15) | 506.66(504.71,508.62) | 1494283.83(1189504.26,1799251.19) | 2643.30(2639.05,2647.55) | 6.63(5.90,7.36) | 6.29(5.65,6.95) |
| United States Virgin Islands | female | 37.68(27.46,49.66) | 184.24(130.03,253.93) | 20.52(14.75,27.61) | 174.60(106.99,271.03) | -1.83(-2.18,-1.48) | -0.20(-0.31,-0.08) |
| Uruguay | female | 1222.18(821.68,1684.99) | 214.17(202.33,226.53) | 1265.45(865.64,1677.53) | 209.57(198.16,221.47) | 0.19(0.04,0.34) | 0.01(-0.10,0.13) |
| Uzbekistan | female | 8341.12(5546.65,11355.37) | 193.08(188.93,197.31) | 13800.20(9557.45,18635.99) | 199.21(195.88,202.59) | 2.03(1.82,2.25) | 0.27(0.17,0.38) |
| Vanuatu | female | 31.35(20.16,43.00) | 105.47(71.59,151.12) | 66.79(43.64,94.36) | 105.23(81.43,134.19) | 2.51(2.46,2.55) | -0.04(-0.06,-0.02) |
| Venezuela (Bolivarian Republic of) | female | 6110.25(4076.71,8276.23) | 153.18(149.35,157.09) | 7023.18(4699.50,9609.26) | 143.49(140.12,146.93) | 1.08(0.69,1.47) | -0.19(-0.26,-0.12) |
| Viet Nam | female | 16218.53(10909.73,22900.71) | 108.86(107.18,110.56) | 20898.71(13967.09,29131.76) | 111.10(109.58,112.64) | 1.15(0.96,1.34) | 0.18(-0.02,0.38) |
| Yemen | female | 3919.97(2652.15,5430.37) | 172.55(167.16,178.08) | 12629.93(8656.91,17348.04) | 188.84(185.55,192.18) | 4.20(3.93,4.47) | 0.36(0.24,0.49) |
| Zambia | female | 1362.52(938.69,1834.92) | 92.64(87.60,97.92) | 3178.46(2208.39,4328.36) | 79.45(76.68,82.30) | 2.86(2.67,3.05) | -0.49(-0.55,-0.43) |
| Zimbabwe | female | 3106.70(2027.97,4410.45) | 158.40(152.76,164.22) | 4846.39(3318.62,6693.75) | 148.83(144.64,153.11) | 1.36(1.27,1.46) | -0.16(-0.20,-0.11) |
| Afghanistan | male | 2829.72(2070.44,3746.93) | 240.03(229.84,250.64) | 18385.61(13960.23,24046.48) | 308.32(303.70,313.01) | 5.68(4.98,6.39) | 1.12(0.89,1.34) |
| Albania | male | 2003.58(1590.39,2480.63) | 275.82(263.84,288.23) | 1964.32(1533.79,2436.72) | 381.04(364.32,398.38) | 0.08(-0.12,0.29) | 1.15(0.76,1.54) |
| Algeria | male | 13521.97(10545.58,17402.30) | 274.36(269.67,279.13) | 33393.94(25486.82,42944.94) | 378.62(374.54,382.73) | 2.99(2.66,3.33) | 0.95(0.84,1.07) |
| American Samoa | male | 18.68(13.11,25.17) | 181.02(108.01,288.49) | 16.72(11.74,22.09) | 195.89(112.80,318.68) | -0.75(-0.99,-0.51) | 0.22(0.00,0.45) |
| Andorra | male | 36.00(25.41,47.75) | 261.28(181.63,368.37) | 34.77(24.44,46.97) | 267.47(184.94,377.11) | -0.54(-0.94,-0.15) | 0.14(0.05,0.24) |
| Angola | male | 2246.83(1580.09,2963.06) | 118.57(113.67,123.64) | 7596.45(5343.97,10249.76) | 139.57(136.40,142.80) | 4.14(4.02,4.25) | 0.65(0.55,0.74) |
| Antigua and Barbuda | male | 23.84(16.76,32.61) | 187.70(119.85,282.47) | 34.13(24.47,45.76) | 199.00(137.82,279.16) | 0.75(0.05,1.45) | -0.22(-0.81,0.36) |
| Argentina | male | 14563.24(9492.92,20147.42) | 241.84(237.93,245.81) | 21172.26(14620.46,28839.79) | 242.64(239.38,245.93) | 1.39(1.20,1.58) | 0.11(-0.03,0.25) |
| Armenia | male | 1931.09(1323.17,2618.93) | 266.41(254.61,278.64) | 1254.43(867.53,1640.37) | 231.71(218.82,245.21) | -0.91(-1.23,-0.59) | -0.35(-0.59,-0.10) |
| Australia | male | 43377.06(35509.03,51216.43) | 1275.39(1263.41,1287.46) | 56194.36(46868.82,66752.34) | 1239.98(1229.68,1250.35) | 0.08(-0.37,0.53) | -0.82(-1.26,-0.38) |
| Austria | male | 10501.94(8262.51,12756.17) | 656.81(644.23,669.60) | 10829.76(8740.82,12833.04) | 729.62(715.81,743.67) | -0.56(-0.88,-0.24) | -0.31(-0.79,0.17) |
| Azerbaijan | male | 3913.64(2822.25,5246.27) | 248.35(240.57,256.33) | 5160.80(3731.69,6665.95) | 232.47(226.08,239.01) | 1.39(1.17,1.62) | -0.04(-0.17,0.09) |
| Bahamas | male | 118.13(84.49,163.75) | 199.46(164.91,239.70) | 154.48(112.50,208.12) | 204.70(173.69,239.67) | 0.42(0.06,0.78) | -0.28(-0.62,0.06) |
| Bahrain | male | 281.29(192.22,396.06) | 167.88(148.02,190.31) | 799.83(574.30,1062.42) | 165.44(153.84,177.84) | 4.73(3.98,5.49) | -0.03(-0.17,0.11) |
| Bangladesh | male | 23738.65(16582.78,31778.79) | 113.35(111.90,114.82) | 40842.87(28283.97,55015.36) | 124.19(122.99,125.40) | 1.78(1.66,1.90) | 0.36(0.31,0.41) |
| Barbados | male | 100.94(68.80,142.40) | 186.08(151.54,226.28) | 91.40(65.58,124.59) | 188.42(151.72,231.50) | -0.53(-0.69,-0.37) | -0.19(-0.46,0.07) |
| Belarus | male | 13597.16(9736.04,18145.72) | 672.70(661.36,684.19) | 10093.19(8098.88,12287.30) | 638.29(625.45,651.35) | -1.40(-2.26,-0.54) | -0.79(-1.61,0.03) |
| Belgium | male | 7297.05(5658.68,8904.25) | 381.01(372.28,389.91) | 10194.46(8356.88,12226.62) | 564.47(553.51,575.61) | 0.48(0.26,0.70) | 0.60(0.31,0.89) |
| Belize | male | 69.35(47.38,97.35) | 181.99(140.98,232.72) | 170.55(121.13,238.86) | 181.15(154.88,210.80) | 2.89(2.67,3.11) | -0.18(-0.41,0.05) |
| Benin | male | 540.81(367.70,741.61) | 71.97(65.97,78.41) | 1818.83(1218.12,2528.35) | 72.32(68.99,75.79) | 4.10(4.05,4.16) | 0.12(0.07,0.17) |
| Bermuda | male | 30.53(22.90,39.89) | 235.54(158.55,341.13) | 24.99(19.44,31.11) | 286.47(183.59,429.43) | -2.09(-3.09,-1.08) | -0.70(-1.63,0.23) |
| Bhutan | male | 160.83(112.90,222.61) | 112.38(95.16,132.21) | 233.09(165.21,317.32) | 124.59(109.08,141.82) | 1.85(1.59,2.11) | 0.39(0.32,0.46) |
| Bolivia (Plurinational State of) | male | 2247.85(1606.58,2943.36) | 187.32(179.58,195.32) | 5049.19(3640.15,6690.28) | 202.42(196.87,208.09) | 2.71(2.45,2.97) | 0.17(0.06,0.29) |
| Bosnia and Herzegovina | male | 1537.57(1099.20,2044.04) | 154.34(146.72,162.27) | 852.81(605.88,1165.52) | 164.25(153.33,175.80) | -0.66(-1.27,-0.05) | 0.85(0.50,1.21) |
| Botswana | male | 325.39(226.87,461.99) | 142.77(127.36,159.69) | 748.48(536.13,988.43) | 141.08(131.14,151.59) | 2.85(2.63,3.07) | 0.05(-0.02,0.11) |
| Brazil | male | 72003.30(48575.70,97060.44) | 231.53(229.83,233.23) | 139961.69(106644.91,174066.84) | 330.95(329.21,332.69) | 2.67(2.43,2.91) | 1.59(1.37,1.82) |
| Brunei Darussalam | male | 214.15(158.76,269.62) | 318.64(277.25,364.73) | 286.91(214.85,372.83) | 254.93(226.04,286.81) | 0.78(0.60,0.95) | -1.05(-1.23,-0.88) |
| Bulgaria | male | 4019.27(3040.66,5144.49) | 271.11(262.78,279.65) | 3785.72(2953.26,4695.56) | 399.42(386.48,412.71) | -0.19(-0.69,0.31) | 1.16(0.87,1.46) |
| Burkina Faso | male | 909.49(626.96,1269.57) | 66.24(61.91,70.82) | 2696.08(1793.17,3662.44) | 70.73(68.05,73.48) | 3.81(3.71,3.91) | 0.31(0.24,0.37) |
| Burundi | male | 1456.70(975.80,2160.34) | 160.13(151.93,168.68) | 4124.81(2780.49,5970.09) | 170.41(165.23,175.71) | 3.65(3.19,4.12) | 0.09(-0.01,0.18) |
| Cabo Verde | male | 53.91(37.61,72.37) | 86.87(64.12,117.21) | 110.01(78.49,146.49) | 83.95(68.96,101.37) | 2.69(2.48,2.90) | -0.03(-0.07,0.01) |
| Cambodia | male | 2787.57(1892.16,3904.97) | 158.38(152.43,164.53) | 5799.29(3860.21,8211.56) | 157.59(153.56,161.70) | 2.58(2.44,2.73) | 0.02(-0.02,0.06) |
| Cameroon | male | 1372.57(940.16,1840.83) | 78.61(74.46,82.96) | 4929.02(3319.11,6563.25) | 79.48(77.26,81.75) | 4.50(4.39,4.61) | 0.19(0.11,0.26) |
| Canada | male | 39757.37(30422.61,49130.99) | 701.52(694.58,708.52) | 137269.01(119008.53,156240.74) | 2204.19(2192.48,2215.95) | 4.04(3.39,4.69) | 3.80(3.31,4.29) |
| Central African Republic | male | 586.94(409.30,786.84) | 120.96(111.22,131.40) | 1201.33(818.67,1631.40) | 122.17(115.25,129.41) | 2.27(2.08,2.46) | 0.01(-0.04,0.06) |
| Chad | male | 681.28(447.01,950.66) | 71.47(66.12,77.18) | 1952.33(1305.08,2735.08) | 67.28(64.24,70.45) | 3.53(3.41,3.66) | -0.07(-0.13,-0.01) |
| Chile | male | 6814.59(4859.73,9221.52) | 238.82(233.16,244.58) | 10274.60(7459.38,13139.85) | 284.37(278.86,289.97) | 1.18(0.99,1.38) | 0.45(0.25,0.65) |
| China | male | 1528637.72(1212381.59,1844842.27) | 541.28(540.42,542.14) | 677739.86(519753.50,847029.49) | 276.64(275.96,277.31) | -3.99(-4.48,-3.49) | -3.55(-4.07,-3.02) |
| Colombia | male | 13861.82(10178.67,17887.23) | 200.68(197.33,204.07) | 22194.11(16666.90,28723.13) | 216.42(213.57,219.29) | 1.43(1.31,1.54) | 0.24(0.14,0.33) |
| Comoros | male | 113.69(71.00,163.49) | 164.69(135.11,199.15) | 300.08(201.59,423.30) | 205.45(182.81,230.16) | 2.90(2.67,3.12) | 0.46(0.19,0.72) |
| Congo | male | 603.26(431.98,795.50) | 139.24(128.08,151.19) | 1575.90(1117.93,2132.73) | 151.04(143.65,158.71) | 3.09(2.94,3.24) | 0.26(0.18,0.35) |
| Cook Islands | male | 6.80(4.69,9.63) | 169.48(66.34,367.47) | 4.84(3.32,6.56) | 176.73(55.88,425.06) | -1.30(-1.44,-1.16) | 0.19(0.12,0.25) |
| Costa Rica | male | 1034.64(747.40,1372.49) | 160.85(151.16,171.05) | 1665.20(1301.69,2102.14) | 178.58(170.08,187.40) | 1.33(1.02,1.64) | 0.12(-0.04,0.27) |
| Croatia | male | 3419.00(2698.07,4128.73) | 371.65(359.23,384.40) | 3533.83(2785.42,4274.07) | 538.83(521.01,557.14) | 0.09(-0.66,0.85) | 1.04(0.26,1.83) |
| Cuba | male | 4909.70(3457.06,6538.43) | 196.24(190.72,201.89) | 3029.19(2095.37,4151.46) | 165.03(159.16,171.07) | -1.87(-1.96,-1.77) | -0.63(-0.66,-0.61) |
| Cyprus | male | 711.58(558.17,884.19) | 442.77(410.80,476.64) | 1243.28(969.28,1544.37) | 475.68(448.77,504.03) | 1.97(1.75,2.20) | -0.02(-0.23,0.20) |
| Czechia | male | 5040.94(3739.49,6529.20) | 275.89(268.29,283.66) | 5844.12(4574.71,7221.11) | 394.05(383.77,404.55) | 1.01(0.60,1.43) | 1.63(1.43,1.83) |
| C?te d'Ivoire | male | 1700.03(1144.17,2316.25) | 71.27(67.89,74.79) | 3905.44(2493.22,5404.69) | 69.94(67.75,72.18) | 2.73(2.52,2.95) | 0.04(-0.01,0.10) |
| Democratic People's Republic of Korea | male | 11479.37(8271.35,15191.47) | 288.05(282.79,293.39) | 13683.34(9696.98,18278.72) | 254.30(250.03,258.63) | 0.51(0.43,0.59) | -0.48(-0.57,-0.39) |
| Democratic Republic of the Congo | male | 7645.78(5481.36,10219.48) | 112.89(110.32,115.50) | 22693.97(15684.70,31644.97) | 130.16(128.45,131.88) | 3.64(3.47,3.81) | 0.52(0.37,0.68) |
| Denmark | male | 10053.21(8275.40,12099.08) | 1005.79(986.20,1025.69) | 9193.56(7555.44,10842.65) | 961.53(941.92,981.47) | -0.69(-0.92,-0.47) | -0.32(-0.49,-0.15) |
| Djibouti | male | 128.84(85.46,180.34) | 162.64(135.28,194.19) | 587.12(375.80,848.39) | 204.86(188.62,222.15) | 4.91(4.70,5.12) | 0.66(0.55,0.78) |
| Dominica | male | 34.53(23.48,48.46) | 220.40(152.32,311.42) | 34.32(24.98,46.06) | 258.75(179.52,361.15) | -0.40(-0.58,-0.21) | 0.16(-0.12,0.44) |
| Dominican Republic | male | 1957.28(1325.58,2792.64) | 131.31(125.47,137.38) | 3324.35(2287.63,4541.55) | 143.79(138.94,148.77) | 1.76(1.68,1.85) | 0.27(0.21,0.33) |
| Ecuador | male | 3118.28(2198.87,4091.45) | 154.10(148.68,159.68) | 7371.34(5763.10,9340.66) | 198.94(194.41,203.54) | 2.89(2.74,3.05) | 0.96(0.81,1.10) |
| Egypt | male | 15692.97(10549.75,21170.81) | 142.42(140.18,144.68) | 38515.45(26735.78,51094.96) | 180.15(178.35,181.96) | 3.19(3.08,3.31) | 0.88(0.83,0.93) |
| El Salvador | male | 1889.15(1512.27,2353.76) | 195.08(186.21,204.31) | 2200.69(1727.73,2766.46) | 177.30(169.90,184.97) | 0.18(-0.02,0.38) | -0.55(-0.75,-0.35) |
| Equatorial Guinea | male | 80.71(58.41,110.98) | 126.20(99.62,158.12) | 570.73(408.70,791.95) | 154.82(142.09,168.51) | 6.90(6.76,7.04) | 0.75(0.70,0.79) |
| Eritrea | male | 864.95(593.64,1207.59) | 162.71(151.81,174.21) | 2786.41(1767.11,4033.97) | 209.45(201.71,217.42) | 4.32(4.10,4.53) | 0.77(0.70,0.84) |
| Estonia | male | 2477.27(2018.64,2875.79) | 861.61(827.91,896.36) | 5251.14(4180.83,6403.71) | 2449.88(2382.12,2519.26) | 2.78(1.88,3.70) | 3.42(2.53,4.31) |
| Eswatini | male | 185.49(134.45,250.35) | 144.48(123.79,167.96) | 401.30(295.72,523.11) | 163.53(147.85,180.49) | 2.42(2.22,2.63) | 0.44(0.40,0.48) |
| Ethiopia | male | 7366.64(5427.10,10033.50) | 92.70(90.56,94.89) | 20399.01(14927.54,26930.69) | 97.15(95.80,98.53) | 3.16(3.10,3.23) | 0.09(0.07,0.12) |
| Fiji | male | 273.48(187.84,378.17) | 164.99(145.95,185.96) | 277.64(186.08,372.77) | 153.44(135.92,172.61) | -0.03(-0.18,0.12) | -0.38(-0.47,-0.29) |
| Finland | male | 7092.45(5907.23,8230.07) | 732.23(715.13,749.68) | 13015.10(11044.52,15133.04) | 1490.07(1464.37,1516.13) | 2.42(2.19,2.65) | 2.60(2.34,2.85) |
| France | male | 43517.67(34179.08,52798.04) | 394.64(390.94,398.37) | 44676.30(36003.49,54382.24) | 449.65(445.48,453.85) | 0.38(-0.13,0.90) | 0.74(0.22,1.26) |
| Gabon | male | 281.36(204.11,370.50) | 150.02(132.90,168.81) | 560.49(396.42,742.44) | 165.19(151.74,179.56) | 2.09(2.01,2.18) | 0.17(0.09,0.25) |
| Gambia | male | 137.67(91.28,192.81) | 77.01(64.50,91.50) | 350.52(233.35,472.89) | 74.08(66.42,82.45) | 2.91(2.85,2.98) | -0.08(-0.14,-0.03) |
| Georgia | male | 2745.04(1899.54,3651.60) | 259.58(249.94,269.50) | 1454.34(1096.73,1856.23) | 245.75(233.16,258.90) | -1.30(-2.03,-0.57) | 0.48(-0.28,1.25) |
| Germany | male | 102423.11(84443.58,118862.55) | 636.26(632.32,640.22) | 87269.48(71665.42,103494.25) | 621.98(617.80,626.17) | -1.19(-1.45,-0.94) | -0.55(-0.70,-0.41) |
| Ghana | male | 1960.21(1258.71,2761.57) | 73.18(69.94,76.53) | 5080.50(3484.97,7185.61) | 74.30(72.26,76.38) | 3.21(3.14,3.29) | 0.04(-0.04,0.12) |
| Greece | male | 8652.22(6822.02,10519.49) | 457.52(447.93,467.27) | 11742.97(9871.11,13703.90) | 817.36(802.46,832.47) | -0.49(-1.84,0.87) | 0.51(-0.50,1.54) |
| Greenland | male | 79.90(59.29,102.62) | 527.93(416.10,666.30) | 67.29(52.40,84.47) | 622.44(481.57,795.20) | -0.39(-0.57,-0.21) | 0.72(0.63,0.80) |
| Grenada | male | 34.37(24.20,46.40) | 202.24(139.86,285.28) | 62.20(45.77,80.41) | 291.72(223.38,375.41) | 1.07(0.31,1.83) | 0.42(-0.07,0.91) |
| Guam | male | 78.04(55.84,102.10) | 215.20(169.84,269.62) | 52.36(36.34,69.88) | 177.60(132.75,233.06) | -1.35(-1.64,-1.05) | -0.65(-0.86,-0.44) |
| Guatemala | male | 3249.43(2748.70,3790.18) | 243.07(234.67,251.71) | 9033.33(7396.56,10731.66) | 281.10(275.27,287.04) | 3.50(3.05,3.95) | 0.42(-0.08,0.93) |
| Guinea | male | 614.29(400.68,851.82) | 67.75(62.46,73.39) | 1515.52(1047.71,2083.98) | 65.44(62.14,68.88) | 2.79(2.65,2.93) | -0.02(-0.07,0.04) |
| Guinea-Bissau | male | 117.85(78.31,166.96) | 69.58(57.37,83.87) | 276.94(183.99,375.83) | 69.50(61.47,78.37) | 2.97(2.88,3.07) | 0.09(0.01,0.17) |
| Guyana | male | 251.09(173.33,354.09) | 147.21(129.38,167.01) | 239.82(163.72,326.99) | 151.99(133.19,172.91) | -0.48(-0.63,-0.33) | -0.05(-0.17,0.07) |
| Haiti | male | 1893.71(1306.39,2603.07) | 165.31(157.89,173.00) | 5143.57(3636.59,6936.41) | 192.64(187.40,197.98) | 3.41(3.25,3.57) | 0.46(0.40,0.53) |
| Honduras | male | 1825.22(1418.59,2278.41) | 227.37(216.90,238.25) | 4834.06(3320.98,6658.07) | 232.36(225.78,239.10) | 3.05(2.96,3.15) | -0.12(-0.19,-0.04) |
| Hungary | male | 4646.06(3336.85,6149.87) | 255.97(248.60,263.52) | 4582.28(3384.90,5805.53) | 327.37(317.87,337.09) | 0.04(-0.19,0.27) | 0.85(0.75,0.96) |
| Iceland | male | 362.64(294.65,440.13) | 677.87(609.85,751.58) | 840.62(705.99,977.25) | 1305.92(1218.62,1398.28) | 2.77(2.43,3.11) | 2.03(1.76,2.30) |
| India | male | 239068.24(184693.28,303177.67) | 136.66(136.11,137.21) | 495140.26(378008.28,620188.23) | 157.64(157.20,158.08) | 2.27(1.95,2.59) | 0.28(-0.04,0.60) |
| Indonesia | male | 42452.87(28078.77,60911.54) | 111.19(110.13,112.26) | 71218.38(48599.73,99324.55) | 121.54(120.65,122.44) | 1.36(1.22,1.51) | 0.08(-0.04,0.19) |
| Iran (Islamic Republic of) | male | 91004.65(75087.77,106912.89) | 883.74(877.92,889.60) | 179986.22(148094.87,211066.29) | 995.89(991.15,1000.64) | 2.53(1.71,3.35) | 0.36(-0.11,0.83) |
| Iraq | male | 9801.09(7549.54,12783.26) | 276.21(270.66,281.86) | 30625.19(22804.50,40499.93) | 341.06(337.24,344.91) | 3.52(3.08,3.97) | 0.55(0.36,0.75) |
| Ireland | male | 3432.55(2734.34,4201.68) | 507.09(490.21,524.41) | 9100.77(7603.00,10646.20) | 1179.43(1155.17,1204.08) | 2.62(1.79,3.44) | 1.90(1.32,2.49) |
| Israel | male | 3155.15(2413.56,3923.81) | 339.17(327.41,351.26) | 6743.98(5410.66,8253.74) | 403.81(394.22,413.57) | 2.03(1.41,2.65) | 0.11(-0.40,0.63) |
| Italy | male | 102546.16(85521.90,120359.17) | 933.04(927.34,938.78) | 30215.16(22701.46,38102.35) | 369.39(365.21,373.60) | -4.88(-5.19,-4.57) | -3.87(-4.13,-3.61) |
| Jamaica | male | 1013.07(697.72,1379.44) | 206.63(193.91,220.04) | 1214.41(852.00,1663.03) | 201.82(190.59,213.56) | 0.28(0.06,0.50) | -0.44(-0.65,-0.22) |
| Japan | male | 42006.46(29536.92,56697.41) | 188.62(186.82,190.44) | 29587.66(20997.91,39204.50) | 179.15(177.10,181.22) | -1.36(-1.68,-1.04) | -0.20(-0.36,-0.04) |
| Jordan | male | 1325.60(906.62,1804.49) | 165.69(156.47,175.39) | 4569.98(3198.39,6374.95) | 156.65(152.13,161.27) | 4.20(3.95,4.44) | -0.08(-0.19,0.03) |
| Kazakhstan | male | 14337.12(11151.41,17908.81) | 417.04(410.23,423.94) | 31589.64(24533.98,38272.24) | 854.93(845.43,864.51) | 2.28(1.24,3.33) | 1.73(0.50,2.97) |
| Kenya | male | 3245.41(2385.64,4184.45) | 86.30(83.28,89.41) | 10035.88(7503.21,12660.25) | 104.47(102.42,106.55) | 3.81(3.66,3.97) | 0.67(0.61,0.73) |
| Kiribati | male | 124.07(90.59,164.25) | 795.31(660.45,952.27) | 243.80(169.41,341.08) | 991.72(871.00,1125.07) | 2.50(2.28,2.71) | 0.60(0.47,0.72) |
| Kuwait | male | 1010.02(741.39,1342.14) | 193.73(181.67,206.53) | 3075.33(2442.38,3871.91) | 271.47(261.39,281.91) | 4.17(3.77,4.57) | 0.95(0.64,1.26) |
| Kyrgyzstan | male | 2970.25(2232.34,3731.73) | 333.48(321.53,345.79) | 5404.23(4218.85,6790.52) | 387.06(376.78,397.55) | 2.29(1.39,3.19) | 0.78(-0.16,1.73) |
| Lao People's Democratic Republic | male | 890.84(610.66,1219.98) | 122.75(114.75,131.20) | 2004.75(1385.43,2701.77) | 122.81(117.49,128.32) | 2.76(2.57,2.95) | -0.03(-0.10,0.05) |
| Latvia | male | 2872.52(2268.76,3444.43) | 592.13(570.62,614.26) | 2289.86(1875.15,2685.07) | 808.19(774.47,843.14) | -1.41(-1.83,-0.99) | 0.15(-0.24,0.55) |
| Lebanon | male | 976.93(704.43,1281.13) | 182.11(170.71,194.13) | 4037.34(2962.75,5194.68) | 324.46(314.33,334.85) | 5.37(4.89,5.85) | 2.06(1.96,2.17) |
| Lesotho | male | 294.89(208.35,404.04) | 132.78(117.82,149.19) | 568.28(406.80,767.79) | 139.20(127.93,151.23) | 2.24(2.11,2.38) | 0.25(0.22,0.28) |
| Liberia | male | 334.86(227.76,460.83) | 76.42(68.42,85.13) | 836.91(557.09,1164.95) | 76.73(71.58,82.17) | 4.82(4.29,5.35) | 0.12(0.07,0.18) |
| Libya | male | 2541.24(1950.18,3283.73) | 298.47(286.84,310.47) | 9002.54(7139.11,11765.90) | 571.28(559.46,583.29) | 4.76(4.39,5.15) | 2.32(2.22,2.42) |
| Lithuania | male | 3788.34(3001.85,4478.43) | 531.45(514.63,548.70) | 5811.29(4714.09,6955.34) | 1314.58(1280.70,1349.24) | 1.11(0.85,1.38) | 2.87(2.61,3.13) |
| Luxembourg | male | 671.46(556.00,787.94) | 868.23(802.96,938.05) | 755.36(615.55,897.84) | 662.52(615.28,712.80) | -0.60(-0.96,-0.24) | -1.79(-2.20,-1.37) |
| Madagascar | male | 2801.98(2009.57,3855.59) | 140.88(135.63,146.29) | 8434.48(5769.47,11618.67) | 168.56(164.93,172.24) | 3.49(3.41,3.57) | 0.46(0.38,0.55) |
| Malawi | male | 2362.36(1646.35,3360.40) | 149.16(143.07,155.45) | 6630.90(4415.32,9471.32) | 204.04(199.07,209.10) | 3.38(3.21,3.54) | 0.97(0.93,1.01) |
| Malaysia | male | 6591.88(4498.93,8961.70) | 177.26(172.99,181.60) | 12813.91(8902.27,17227.82) | 172.36(169.38,175.38) | 2.29(2.09,2.48) | -0.09(-0.13,-0.05) |
| Maldives | male | 67.47(44.12,94.09) | 165.77(127.70,213.47) | 326.14(225.32,437.05) | 186.66(165.00,211.16) | 5.90(5.54,6.27) | 0.47(0.40,0.55) |
| Mali | male | 932.41(633.44,1293.64) | 69.24(64.82,73.91) | 2764.22(1903.85,3833.48) | 66.43(63.90,69.04) | 3.66(3.54,3.79) | -0.04(-0.09,0.02) |
| Malta | male | 268.79(210.78,334.05) | 387.25(342.05,437.02) | 490.61(395.44,583.01) | 697.02(635.32,763.83) | 2.14(1.76,2.52) | 1.74(1.44,2.04) |
| Marshall Islands | male | 16.52(11.64,22.07) | 190.21(109.16,311.82) | 22.56(15.36,29.78) | 186.25(117.44,281.21) | 0.94(0.78,1.09) | -0.07(-0.14,0.01) |
| Mauritania | male | 280.97(187.19,385.60) | 77.06(68.22,86.81) | 586.47(393.40,784.78) | 73.59(67.68,79.92) | 2.48(2.40,2.56) | -0.04(-0.09,0.01) |
| Mauritius | male | 600.05(389.74,829.31) | 231.68(213.48,251.06) | 1152.17(943.44,1392.94) | 491.42(463.41,520.73) | 2.02(1.71,2.33) | 2.36(2.10,2.62) |
| Mexico | male | 29261.24(22247.54,37514.54) | 170.20(168.22,172.20) | 46034.35(36493.50,57599.38) | 181.80(180.14,183.46) | 1.25(0.99,1.51) | 0.06(-0.14,0.27) |
| Micronesia (Federated States of) | male | 39.06(27.28,51.58) | 196.46(139.27,270.32) | 42.38(29.35,56.55) | 191.43(137.93,259.96) | 0.08(0.00,0.15) | -0.16(-0.21,-0.11) |
| Monaco | male | 15.08(10.65,20.38) | 319.86(177.68,548.38) | 16.17(11.66,21.36) | 345.54(197.75,564.20) | 0.11(0.07,0.15) | 0.32(0.21,0.43) |
| Mongolia | male | 1114.84(794.11,1501.96) | 252.17(237.32,267.83) | 1889.12(1380.33,2411.97) | 295.66(282.36,309.46) | 2.20(1.75,2.66) | 0.92(0.62,1.23) |
| Montenegro | male | 260.87(181.24,348.74) | 199.81(176.29,225.64) | 215.37(148.38,285.46) | 203.55(177.18,232.88) | -0.37(-0.45,-0.28) | 0.23(0.17,0.30) |
| Morocco | male | 13538.53(10219.67,17579.20) | 270.49(265.91,275.14) | 25592.67(18975.32,35078.00) | 346.16(341.93,350.43) | 2.00(1.89,2.10) | 0.69(0.63,0.74) |
| Mozambique | male | 2044.96(1466.52,2705.76) | 103.53(99.04,108.18) | 7880.88(5615.46,10491.88) | 163.90(160.23,167.64) | 4.62(4.53,4.72) | 1.70(1.62,1.78) |
| Myanmar | male | 14344.80(9881.50,19941.40) | 169.07(166.29,171.88) | 17613.69(11680.22,24765.28) | 160.06(157.71,162.45) | 0.50(0.31,0.69) | -0.22(-0.35,-0.10) |
| Namibia | male | 381.02(263.77,525.32) | 145.65(131.06,161.59) | 792.09(567.08,1032.80) | 155.02(144.37,166.28) | 2.37(2.17,2.57) | 0.27(0.23,0.30) |
| Nauru | male | 3.91(2.74,5.23) | 191.39(50.91,511.18) | 4.42(3.03,6.02) | 186.16(54.78,472.71) | 0.24(0.17,0.31) | -0.12(-0.20,-0.04) |
| Nepal | male | 4151.44(3014.47,5708.93) | 120.64(116.97,124.40) | 7682.01(5480.18,10480.65) | 126.34(123.50,129.24) | 1.88(1.76,2.00) | 0.15(0.11,0.19) |
| Netherlands | male | 11082.09(8387.05,13866.86) | 356.91(350.27,363.65) | 12465.36(9960.52,15458.59) | 459.56(451.51,467.72) | -0.07(-0.34,0.19) | 0.55(0.33,0.76) |
| New Zealand | male | 3796.54(2777.86,4847.35) | 553.40(535.94,571.30) | 6668.65(5102.14,8214.94) | 700.22(683.43,717.33) | 0.95(0.62,1.27) | 0.10(-0.18,0.38) |
| Nicaragua | male | 894.32(643.05,1177.77) | 129.47(120.94,138.49) | 1669.74(1202.42,2257.11) | 116.63(111.09,122.37) | 2.05(1.78,2.31) | -0.34(-0.46,-0.21) |
| Niger | male | 898.48(601.10,1253.18) | 69.77(65.23,74.57) | 2878.50(1906.66,4024.36) | 68.21(65.67,70.84) | 3.73(3.68,3.78) | 0.04(-0.02,0.10) |
| Nigeria | male | 10159.24(7318.64,13523.32) | 61.72(60.52,62.94) | 24767.80(17481.24,32751.68) | 61.34(60.56,62.12) | 2.99(2.93,3.06) | 0.10(0.02,0.17) |
| Niue | male | 0.82(0.59,1.10) | 195.35(2.29,1377.76) | 0.53(0.37,0.72) | 186.33(0.26,1774.74) | -1.75(-2.06,-1.44) | -0.21(-0.26,-0.15) |
| North Macedonia | male | 1016.31(718.06,1337.57) | 250.09(234.95,265.96) | 1135.76(837.73,1449.69) | 284.24(267.69,301.66) | 1.37(0.83,1.91) | 1.12(0.70,1.55) |
| Northern Mariana Islands | male | 25.87(18.73,33.84) | 221.47(143.74,330.58) | 19.36(13.24,27.05) | 230.75(139.43,361.42) | -1.92(-2.52,-1.31) | 0.03(-0.22,0.27) |
| Norway | male | 7146.03(5830.51,8633.56) | 857.79(838.00,877.95) | 10900.08(8970.49,12836.65) | 1166.98(1145.06,1189.24) | 0.13(-0.77,1.04) | -0.20(-1.21,0.82) |
| Oman | male | 945.14(675.64,1297.86) | 167.12(156.46,178.39) | 2572.02(1872.91,3363.90) | 159.81(152.99,166.95) | 4.04(3.65,4.43) | 0.03(-0.10,0.16) |
| Pakistan | male | 23474.42(17789.55,30541.33) | 112.74(111.27,114.22) | 56717.22(43160.84,71158.03) | 114.20(113.26,115.15) | 3.09(2.86,3.33) | 0.18(-0.06,0.41) |
| Palau | male | 6.02(3.99,8.41) | 162.52(59.66,361.49) | 5.83(3.86,7.99) | 171.08(60.97,390.98) | -0.52(-0.96,-0.07) | 0.16(0.14,0.18) |
| Palestine | male | 608.34(397.37,863.53) | 158.56(145.75,172.36) | 1629.34(1120.20,2220.91) | 144.51(137.51,151.81) | 3.28(3.04,3.51) | -0.27(-0.38,-0.16) |
| Panama | male | 844.17(627.52,1111.85) | 163.96(153.00,175.56) | 1392.26(1046.49,1811.44) | 164.70(156.15,173.60) | 1.24(1.03,1.45) | -0.19(-0.37,-0.01) |
| Papua New Guinea | male | 1415.71(961.09,1963.70) | 162.47(154.04,171.29) | 3580.80(2455.54,4958.48) | 160.09(154.87,165.45) | 3.12(3.07,3.17) | -0.08(-0.11,-0.04) |
| Paraguay | male | 1200.08(793.69,1633.06) | 153.04(144.47,162.02) | 2595.92(1833.88,3502.06) | 165.58(159.27,172.08) | 2.87(2.73,3.00) | 0.41(0.35,0.47) |
| Peru | male | 6747.77(4837.58,8779.77) | 154.81(151.09,158.60) | 12875.10(9658.49,16430.67) | 169.00(166.09,171.94) | 2.20(2.03,2.37) | 0.52(0.32,0.71) |
| Philippines | male | 19782.04(13594.58,26819.04) | 153.17(151.03,155.34) | 35364.74(24281.00,47573.41) | 145.11(143.60,146.63) | 1.85(1.76,1.93) | -0.20(-0.26,-0.14) |
| Poland | male | 22832.40(17446.19,28482.76) | 319.83(315.65,324.06) | 22053.71(17406.62,26986.41) | 363.84(358.92,368.81) | -0.28(-0.48,-0.07) | -0.04(-0.46,0.38) |
| Portugal | male | 7608.29(6090.24,9160.61) | 405.87(396.80,415.10) | 5225.60(4190.90,6375.68) | 353.25(343.69,363.02) | -2.49(-2.97,-2.01) | -1.61(-2.03,-1.19) |
| Puerto Rico | male | 3215.73(2325.38,4097.69) | 479.88(463.40,496.80) | 3668.25(3048.11,4380.11) | 711.71(688.85,735.14) | -2.90(-4.67,-1.10) | -2.06(-3.73,-0.36) |
| Qatar | male | 306.02(214.23,407.90) | 167.54(147.95,189.93) | 2075.12(1452.09,2861.40) | 158.07(149.84,166.97) | 9.09(7.79,10.41) | -0.05(-0.19,0.10) |
| Republic of Korea | male | 24364.83(17070.35,32248.22) | 223.70(220.89,226.53) | 18612.75(12636.03,25072.50) | 216.91(213.74,220.12) | -1.01(-1.15,-0.87) | -0.26(-0.36,-0.15) |
| Republic of Moldova | male | 3554.23(2616.46,4488.25) | 420.14(406.34,434.31) | 2529.57(1915.66,3140.68) | 396.08(380.18,412.56) | -0.89(-1.24,-0.53) | -0.33(-0.55,-0.11) |
| Romania | male | 9388.32(6788.95,12257.03) | 217.01(212.60,221.50) | 5932.61(4804.57,7258.36) | 214.33(208.85,219.92) | -1.75(-1.91,-1.60) | -0.29(-0.59,0.01) |
| Russian Federation | male | 299940.11(239091.08,358853.13) | 1014.33(1010.67,1018.01) | 358502.30(307856.70,412610.60) | 1421.31(1416.48,1426.16) | 0.05(-1.16,1.28) | 0.24(-0.93,1.42) |
| Rwanda | male | 2201.06(1535.98,3077.53) | 181.17(173.62,188.98) | 5410.55(3533.38,7846.49) | 211.24(205.62,216.98) | 3.35(2.87,3.82) | 0.29(0.20,0.39) |
| Saint Kitts and Nevis | male | 14.99(9.88,21.11) | 172.38(96.08,290.21) | 19.84(13.62,26.72) | 176.08(106.98,275.22) | 0.73(0.44,1.02) | -0.26(-0.49,-0.03) |
| Saint Lucia | male | 58.82(40.32,81.14) | 205.65(155.57,268.88) | 66.79(46.84,89.17) | 198.55(153.66,253.05) | 0.38(-0.06,0.81) | -0.19(-0.52,0.14) |
| Saint Vincent and the Grenadines | male | 40.97(27.05,58.46) | 172.42(122.87,238.14) | 42.08(29.88,57.66) | 199.44(143.75,269.98) | -0.57(-0.95,-0.20) | -0.07(-0.40,0.26) |
| Samoa | male | 70.76(48.57,96.66) | 190.99(147.86,244.91) | 84.35(58.66,111.87) | 200.43(159.70,249.14) | 0.26(0.11,0.40) | 0.01(-0.05,0.07) |
| San Marino | male | 15.09(10.12,20.37) | 319.62(179.09,529.90) | 14.27(9.66,19.15) | 322.44(177.35,539.94) | -0.41(-0.58,-0.24) | 0.13(0.02,0.25) |
| Sao Tome and Principe | male | 17.89(12.75,24.47) | 87.57(50.62,145.73) | 53.34(38.24,71.09) | 118.29(88.58,155.10) | 3.68(3.60,3.76) | 1.01(0.91,1.11) |
| Saudi Arabia | male | 6332.85(4185.23,8861.63) | 162.75(158.75,166.83) | 18655.54(13089.91,25813.42) | 165.90(163.45,168.39) | 4.10(3.79,4.40) | 0.24(0.11,0.37) |
| Senegal | male | 968.20(657.03,1348.11) | 76.29(71.48,81.38) | 2269.74(1508.34,3091.41) | 71.76(68.79,74.84) | 2.74(2.67,2.82) | -0.13(-0.18,-0.08) |
| Serbia | male | 4824.96(3791.44,6053.24) | 262.36(255.00,269.89) | 5206.70(4022.65,6456.74) | 328.25(319.31,337.39) | 0.47(0.05,0.89) | 1.15(0.60,1.69) |
| Seychelles | male | 33.86(23.06,46.02) | 209.86(144.92,296.54) | 50.76(36.96,65.12) | 236.71(175.44,314.11) | 1.38(1.01,1.75) | 0.29(-0.01,0.60) |
| Sierra Leone | male | 559.04(381.56,771.42) | 76.35(70.11,83.03) | 1292.80(856.68,1825.95) | 71.65(67.75,75.73) | 3.38(2.98,3.79) | -0.16(-0.21,-0.11) |
| Singapore | male | 1740.86(1154.47,2366.32) | 220.85(210.57,231.54) | 2014.20(1396.97,2742.45) | 215.47(205.55,225.79) | 0.78(0.27,1.29) | -0.27(-0.39,-0.16) |
| Slovakia | male | 3297.60(2447.42,4182.78) | 324.68(313.65,336.01) | 2479.22(1844.35,3169.34) | 285.37(273.97,297.17) | -0.98(-1.39,-0.58) | -0.68(-0.90,-0.46) |
| Slovenia | male | 1299.72(1055.43,1571.52) | 335.32(317.24,354.20) | 1665.86(1327.75,2038.28) | 567.37(539.82,596.07) | 1.20(0.52,1.89) | 1.93(1.40,2.47) |
| Solomon Islands | male | 112.37(77.90,153.85) | 170.84(139.99,207.27) | 238.52(168.15,321.94) | 172.13(150.82,195.77) | 2.18(2.04,2.33) | -0.02(-0.07,0.04) |
| Somalia | male | 1999.78(1331.84,3047.34) | 147.22(140.78,153.88) | 5590.69(3633.37,8480.08) | 154.24(150.14,158.43) | 4.29(3.98,4.59) | 0.14(0.09,0.20) |
| South Africa | male | 24246.14(18706.03,29511.35) | 324.11(320.01,328.25) | 32915.82(26392.70,40385.18) | 266.24(263.36,269.15) | 0.70(0.25,1.15) | -1.04(-1.42,-0.67) |
| South Sudan | male | 1458.93(1008.34,2062.32) | 141.55(134.19,149.24) | 2263.11(1546.12,3217.31) | 161.10(154.34,168.09) | 1.63(1.24,2.03) | 0.40(0.30,0.49) |
| Spain | male | 67987.70(55506.76,80980.31) | 897.07(890.33,903.85) | 35200.62(27710.42,43037.94) | 563.46(557.52,569.45) | -3.11(-3.81,-2.41) | -2.65(-3.11,-2.18) |
| Sri Lanka | male | 11356.10(8719.24,14429.80) | 309.21(303.54,314.97) | 8543.45(6319.00,11276.11) | 218.89(214.27,223.59) | -1.80(-2.17,-1.43) | -1.95(-2.30,-1.59) |
| Sudan | male | 8478.91(6383.00,11291.65) | 245.22(239.93,250.60) | 32308.56(23876.51,41551.37) | 365.20(361.18,369.27) | 4.27(4.21,4.34) | 1.35(1.29,1.42) |
| Suriname | male | 147.65(103.70,198.99) | 172.45(145.20,204.00) | 256.61(194.81,331.78) | 242.19(213.43,273.80) | 1.38(0.73,2.03) | 0.55(0.07,1.05) |
| Sweden | male | 5739.05(4799.38,6719.12) | 372.99(363.39,382.80) | 22772.41(18710.69,27193.16) | 1329.94(1312.55,1347.51) | 5.14(4.79,5.50) | 4.78(4.37,5.18) |
| Switzerland | male | 27337.56(21959.28,31931.69) | 1926.60(1903.64,1949.81) | 9933.38(8183.47,11842.85) | 681.89(668.31,695.70) | -3.93(-4.31,-3.56) | -4.11(-4.41,-3.82) |
| Syrian Arab Republic | male | 4330.62(3189.58,5682.38) | 185.51(179.88,191.29) | 4044.62(3040.47,5077.18) | 209.75(202.29,217.45) | 0.13(-1.31,1.59) | 0.59(0.43,0.74) |
| Taiwan (Province of China) | male | 17089.92(13868.83,21450.07) | 355.29(349.98,360.67) | 18599.47(14463.12,23760.25) | 445.83(439.37,452.37) | -0.11(-0.37,0.16) | 0.28(-0.03,0.59) |
| Tajikistan | male | 2532.89(1760.03,3422.07) | 241.46(232.01,251.25) | 4903.86(3392.40,6357.08) | 228.37(222.02,234.87) | 2.47(2.32,2.61) | -0.14(-0.20,-0.08) |
| Thailand | male | 39719.05(25369.36,57480.01) | 303.03(300.04,306.04) | 34078.74(22754.86,47809.15) | 329.22(325.72,332.75) | -0.76(-1.20,-0.33) | 0.18(-0.12,0.49) |
| Timor-Leste | male | 272.56(178.93,384.74) | 163.96(144.95,184.96) | 471.33(314.15,674.51) | 166.27(151.17,182.67) | 1.73(1.50,1.96) | 0.07(0.02,0.12) |
| Togo | male | 414.50(279.04,577.71) | 65.68(59.36,72.56) | 1106.00(732.04,1536.34) | 69.48(65.40,73.77) | 3.26(3.24,3.28) | 0.35(0.26,0.44) |
| Tokelau | male | 0.55(0.39,0.76) | 187.63(0.30,1905.92) | 0.47(0.33,0.63) | 184.65(0.11,1981.03) | -0.71(-1.02,-0.41) | -0.15(-0.22,-0.09) |
| Tonga | male | 32.27(21.96,44.15) | 171.09(115.27,249.69) | 33.72(23.21,45.51) | 175.94(120.88,249.32) | -0.03(-0.17,0.11) | 0.08(0.07,0.09) |
| Trinidad and Tobago | male | 427.50(285.61,595.17) | 168.33(152.72,185.17) | 572.54(430.19,725.52) | 228.93(210.29,248.86) | 0.79(0.29,1.29) | 0.72(0.36,1.09) |
| Tunisia | male | 4539.23(3478.39,5853.40) | 270.22(262.32,278.32) | 8532.60(6591.60,10879.47) | 391.42(383.09,399.88) | 2.04(1.71,2.36) | 1.15(1.02,1.27) |
| Türkiye | male | 21275.94(15325.61,28594.91) | 177.90(175.50,180.32) | 32083.90(24226.18,41427.44) | 196.21(194.07,198.37) | 1.37(1.23,1.51) | 0.46(0.37,0.56) |
| Turkmenistan | male | 1942.00(1342.05,2530.51) | 256.24(244.85,268.06) | 5873.75(4504.00,7474.96) | 521.58(508.32,535.11) | 3.81(3.41,4.21) | 2.44(2.04,2.83) |
| Tuvalu | male | 3.19(2.19,4.17) | 184.98(40.50,544.27) | 4.91(3.47,6.62) | 182.86(58.47,439.76) | 1.62(1.43,1.81) | -0.08(-0.12,-0.04) |
| Uganda | male | 4031.77(2846.98,5570.45) | 149.05(144.35,153.88) | 13572.81(9625.19,18373.99) | 198.13(194.71,201.59) | 3.86(3.77,3.94) | 0.79(0.73,0.86) |
| Ukraine | male | 46008.96(36277.42,57582.18) | 475.83(471.47,480.22) | 65822.84(50363.96,82160.16) | 900.27(893.14,907.44) | 1.18(0.71,1.65) | 1.84(1.44,2.24) |
| United Arab Emirates | male | 1768.99(1285.95,2280.67) | 251.61(239.21,264.67) | 12297.06(8948.14,15802.19) | 474.45(462.08,487.11) | 8.72(7.20,10.26) | 2.23(2.12,2.34) |
| United Kingdom | male | 73246.20(58062.95,88557.05) | 686.21(681.24,691.22) | 152746.84(129844.95,175508.38) | 1375.00(1368.08,1381.95) | 1.82(1.30,2.34) | 1.60(1.00,2.21) |
| United Republic of Tanzania | male | 7538.57(5220.51,10475.30) | 194.35(189.91,198.88) | 23036.24(15804.41,32892.92) | 242.48(239.33,245.66) | 3.62(3.56,3.69) | 0.69(0.65,0.74) |
| United States of America | male | 482449.01(375892.46,592167.81) | 913.27(910.68,915.87) | 2515549.79(2111330.63,2934270.87) | 4384.29(4378.87,4389.72) | 5.85(5.49,6.21) | 5.56(5.27,5.86) |
| United States Virgin Islands | male | 83.94(67.18,104.69) | 457.96(364.97,567.90) | 91.66(65.03,130.60) | 816.87(657.38,1004.75) | 0.44(0.18,0.69) | 2.02(1.72,2.33) |
| Uruguay | male | 1564.69(1083.34,2174.23) | 277.34(263.76,291.45) | 1676.86(1199.01,2191.04) | 282.25(268.90,296.11) | 0.21(0.04,0.38) | 0.08(-0.04,0.21) |
| Uzbekistan | male | 9829.93(6795.61,13288.82) | 229.16(224.61,233.78) | 16110.49(11665.54,20712.79) | 227.60(224.08,231.16) | 2.04(1.75,2.33) | 0.19(-0.01,0.39) |
| Vanuatu | male | 48.82(34.04,68.02) | 166.95(123.09,222.37) | 103.60(73.25,139.64) | 165.23(134.77,200.94) | 2.43(2.37,2.48) | -0.08(-0.10,-0.05) |
| Venezuela (Bolivarian Republic of) | male | 4901.24(3374.38,6851.50) | 123.84(120.38,127.38) | 4675.59(3258.72,6347.74) | 111.00(107.80,114.27) | 0.89(0.28,1.50) | -0.24(-0.41,-0.07) |
| Viet Nam | male | 45034.86(34309.25,58525.60) | 330.84(327.76,333.95) | 95236.69(69381.13,124361.15) | 474.88(471.83,477.94) | 2.61(2.48,2.74) | 1.22(1.00,1.45) |
| Yemen | male | 4112.99(2838.92,5861.83) | 196.48(190.45,202.66) | 17704.42(12358.92,23995.92) | 271.29(267.29,275.34) | 5.10(4.72,5.48) | 1.12(0.91,1.33) |
| Zambia | male | 2184.76(1570.08,3011.66) | 182.27(174.46,190.37) | 7750.09(5288.75,11329.82) | 227.65(222.57,232.83) | 4.37(4.19,4.54) | 0.77(0.69,0.85) |
| Zimbabwe | male | 2951.17(2154.20,3921.68) | 164.01(157.98,170.24) | 4994.84(3691.09,6413.44) | 170.61(165.86,175.48) | 1.38(1.24,1.52) | 0.00(-0.17,0.17) |

**Supplementary table 3.** Deaths cases and ASDR of DUDs in 1990 and 2021 and its trends.

| location_name |  | 1990 |  | 2021 |  | EAPC_95%UI |  |
| --- | --- | --- | --- | --- | --- | --- | --- |
|  | sex | number_95%UI | ASR | number_95%UI | ASR | number_95%UI | ASR |
| Afghanistan | both | 46.47(32.64,62.93) | 1.81(1.28,2.52) | 260.41(191.59,360.48) | 2.36(2.07,2.69) | 5.19(4.52,5.86) | 0.84(0.67,1.01) |
| Albania | both | 16.15(12.82,20.06) | 1.13(0.65,1.86) | 11.88(8.53,16.52) | 1.21(0.62,2.17) | -0.85(-1.40,-0.30) | 0.47(-0.20,1.15) |
| Algeria | both | 132.79(97.38,183.19) | 1.45(1.21,1.72) | 358.18(241.77,498.65) | 1.99(1.79,2.21) | 3.16(2.80,3.52) | 0.85(0.67,1.03) |
| American Samoa | both | 0.04(0.03,0.06) | 0.21(0.00,23.65) | 0.05(0.04,0.08) | 0.32(0.00,25.25) | 0.09(-1.58,1.78) | 1.06(-0.67,2.83) |
| Andorra | both | 0.04(0.03,0.05) | 0.15(0.00,20.67) | 0.04(0.03,0.05) | 0.14(0.00,19.44) | -0.25(-0.75,0.26) | 0.20(0.00,0.40) |
| Angola | both | 14.09(7.95,22.99) | 0.39(0.21,0.67) | 54.27(31.20,85.58) | 0.50(0.37,0.65) | 4.69(4.44,4.93) | 0.93(0.71,1.16) |
| Antigua and Barbuda | both | 0.04(0.03,0.04) | 0.15(0.00,16.90) | 0.10(0.08,0.12) | 0.28(0.00,13.02) | 0.94(-1.53,3.48) | -0.02(-2.23,2.23) |
| Argentina | both | 5.82(5.04,6.68) | 0.05(0.02,0.11) | 25.17(21.00,30.69) | 0.14(0.09,0.21) | 6.03(5.35,6.71) | 4.60(3.96,5.25) |
| Armenia | both | 2.78(2.23,3.51) | 0.19(0.04,0.59) | 2.79(2.24,3.51) | 0.24(0.04,0.85) | 1.86(0.29,3.46) | 2.52(0.96,4.10) |
| Australia | both | 347.84(319.55,377.50) | 5.08(4.56,5.65) | 610.25(536.07,691.15) | 6.49(5.98,7.04) | 0.42(-0.41,1.25) | -0.54(-1.34,0.27) |
| Austria | both | 56.88(50.84,62.82) | 1.83(1.38,2.38) | 90.20(79.30,101.91) | 3.04(2.44,3.77) | -0.10(-0.79,0.59) | 0.12(-0.69,0.94) |
| Azerbaijan | both | 11.00(8.38,14.44) | 0.34(0.17,0.64) | 21.16(12.42,32.44) | 0.47(0.29,0.73) | 3.00(2.51,3.50) | 1.75(1.26,2.25) |
| Bahamas | both | 0.20(0.17,0.23) | 0.17(0.00,4.23) | 0.51(0.38,0.68) | 0.33(0.00,3.09) | 0.20(-1.48,1.91) | -0.62(-2.17,0.95) |
| Bahrain | both | 0.62(0.47,0.79) | 0.22(0.00,2.53) | 2.43(1.82,3.16) | 0.31(0.05,1.22) | 4.65(3.92,5.38) | 0.20(-0.28,0.68) |
| Bangladesh | both | 155.95(92.60,225.15) | 0.37(0.31,0.44) | 280.63(142.86,428.87) | 0.41(0.36,0.46) | 1.81(1.70,1.93) | 0.25(0.10,0.40) |
| Barbados | both | 0.07(0.07,0.08) | 0.07(0.00,3.72) | 0.21(0.16,0.28) | 0.20(0.00,4.57) | 0.79(-1.07,2.68) | 1.06(-0.85,3.01) |
| Belarus | both | 144.49(94.12,230.17) | 3.51(2.95,4.14) | 110.77(83.44,143.89) | 3.34(2.72,4.07) | -2.06(-2.99,-1.13) | -1.49(-2.45,-0.52) |
| Belgium | both | 45.48(41.45,50.44) | 1.19(0.87,1.60) | 99.16(86.34,113.12) | 2.69(2.18,3.28) | 1.11(0.57,1.64) | 1.18(0.58,1.79) |
| Belize | both | 0.03(0.03,0.03) | 0.04(0.00,7.47) | 0.26(0.21,0.31) | 0.14(0.00,2.50) | 4.37(2.01,6.80) | 1.05(-1.24,3.39) |
| Benin | both | 0.66(0.40,1.06) | 0.04(0.00,0.35) | 1.45(0.73,2.42) | 0.03(0.00,0.14) | 3.90(2.40,5.42) | 0.17(-1.28,1.63) |
| Bermuda | both | 0.16(0.14,0.18) | 0.57(0.00,21.21) | 0.19(0.15,0.25) | 0.99(0.00,28.09) | -2.45(-4.40,-0.47) | -1.22(-3.11,0.71) |
| Bhutan | both | 0.85(0.41,1.29) | 0.32(0.00,2.60) | 1.41(0.71,2.17) | 0.40(0.03,1.89) | 2.11(1.91,2.31) | 0.67(0.58,0.77) |
| Bolivia (Plurinational State of) | both | 13.56(9.90,18.10) | 0.54(0.29,0.94) | 31.97(20.68,46.55) | 0.65(0.44,0.92) | 2.70(2.47,2.93) | 0.39(0.30,0.48) |
| Bosnia and Herzegovina | both | 3.93(3.07,5.03) | 0.20(0.05,0.54) | 2.23(1.53,3.19) | 0.22(0.03,0.81) | -1.35(-1.70,-1.00) | 0.22(-0.02,0.47) |
| Botswana | both | 2.04(1.28,3.15) | 0.43(0.05,1.71) | 3.90(2.12,6.07) | 0.36(0.10,0.95) | 2.70(2.20,3.19) | -0.08(-0.55,0.39) |
| Brazil | both | 87.60(82.55,92.69) | 0.14(0.11,0.17) | 832.37(771.38,901.30) | 0.97(0.90,1.04) | 8.94(8.06,9.82) | 7.78(6.85,8.71) |
| Brunei Darussalam | both | 1.22(0.92,1.60) | 0.97(0.04,5.02) | 1.45(1.09,1.87) | 0.66(0.04,3.39) | 0.05(-0.34,0.45) | -1.73(-2.14,-1.32) |
| Bulgaria | both | 18.76(15.35,22.38) | 0.63(0.37,0.98) | 21.40(16.50,27.72) | 1.07(0.66,1.67) | 0.46(-0.36,1.29) | 1.64(0.96,2.33) |
| Burkina Faso | both | 0.98(0.50,1.64) | 0.03(0.00,0.21) | 1.85(0.92,3.12) | 0.02(0.00,0.09) | 3.56(2.05,5.11) | 0.21(-1.28,1.72) |
| Burundi | both | 15.10(8.60,26.07) | 0.87(0.48,1.44) | 45.08(22.16,75.85) | 0.99(0.72,1.32) | 3.54(2.90,4.18) | 0.30(0.12,0.47) |
| Cabo Verde | both | 0.14(0.09,0.22) | 0.10(0.00,5.07) | 0.23(0.15,0.38) | 0.09(0.00,1.81) | 2.16(1.87,2.45) | -0.11(-0.38,0.16) |
| Cambodia | both | 5.54(4.11,7.89) | 0.15(0.05,0.36) | 10.73(6.96,15.60) | 0.15(0.07,0.27) | 1.80(1.62,1.99) | -0.42(-0.59,-0.25) |
| Cameroon | both | 1.77(1.11,2.83) | 0.05(0.00,0.21) | 4.67(2.73,7.55) | 0.04(0.01,0.09) | 4.69(3.23,6.18) | 0.56(-0.89,2.03) |
| Canada | both | 238.48(209.61,271.15) | 1.99(1.74,2.26) | 1761.25(1566.00,1942.10) | 13.92(13.28,14.60) | 6.20(5.44,6.98) | 6.21(5.61,6.81) |
| Central African Republic | both | 3.86(2.11,6.77) | 0.41(0.10,1.14) | 8.13(4.05,14.23) | 0.41(0.18,0.82) | 2.31(2.16,2.45) | -0.10(-0.21,0.01) |
| Chad | both | 0.69(0.36,1.13) | 0.03(0.00,0.29) | 1.70(0.93,2.78) | 0.03(0.00,0.13) | 4.42(2.96,5.90) | 0.82(-0.54,2.20) |
| Chile | both | 6.79(5.83,7.75) | 0.12(0.05,0.26) | 25.76(21.09,32.32) | 0.35(0.23,0.52) | 4.93(4.37,5.49) | 4.19(3.64,4.73) |
| China | both | 17949.07(15542.25,20514.39) | 3.36(3.31,3.41) | 4834.36(3891.56,5818.35) | 0.94(0.92,0.97) | -6.17(-6.95,-5.38) | -5.96(-6.79,-5.11) |
| Colombia | both | 77.41(69.67,85.84) | 0.55(0.44,0.69) | 98.04(80.01,118.16) | 0.48(0.39,0.59) | 0.44(-0.30,1.20) | -0.61(-1.36,0.15) |
| Comoros | both | 1.16(0.41,1.95) | 0.91(0.04,4.78) | 3.69(2.14,5.93) | 1.31(0.33,3.50) | 3.29(2.88,3.71) | 0.68(0.22,1.14) |
| Congo | both | 4.32(2.59,6.71) | 0.52(0.15,1.38) | 12.91(7.78,20.86) | 0.61(0.32,1.05) | 3.57(3.34,3.79) | 0.39(0.21,0.56) |
| Cook Islands | both | 0.01(0.01,0.02) | 0.15(0.00,59.10) | 0.01(0.01,0.02) | 0.20(0.00,68.40) | 0.49(-0.28,1.27) | 1.61(0.73,2.50) |
| Costa Rica | both | 3.78(3.16,4.37) | 0.30(0.08,0.82) | 10.49(8.87,12.40) | 0.54(0.26,0.99) | 2.07(1.36,2.77) | 0.70(0.04,1.36) |
| Croatia | both | 24.07(20.19,28.32) | 1.30(0.83,1.96) | 22.85(18.34,28.45) | 1.72(1.08,2.64) | 0.09(-0.84,1.03) | 1.02(0.03,2.02) |
| Cuba | both | 12.49(8.96,15.02) | 0.27(0.14,0.48) | 2.81(2.31,3.35) | 0.07(0.01,0.24) | -6.17(-6.69,-5.65) | -5.06(-5.41,-4.71) |
| Cyprus | both | 5.78(4.46,7.60) | 1.84(0.66,4.15) | 9.62(7.23,12.41) | 1.79(0.82,3.65) | 1.70(1.31,2.08) | -0.43(-0.80,-0.06) |
| Czechia | both | 24.63(21.36,28.70) | 0.67(0.43,1.00) | 31.96(26.54,37.18) | 1.03(0.70,1.48) | 0.95(0.50,1.40) | 1.33(1.01,1.66) |
| C?te d'Ivoire | both | 2.21(1.36,3.50) | 0.05(0.01,0.18) | 3.84(2.07,6.11) | 0.03(0.01,0.09) | 2.79(1.54,4.06) | 0.16(-1.25,1.58) |
| Democratic People's Republic of Korea | both | 102.79(68.66,149.81) | 1.26(1.03,1.53) | 84.05(57.26,125.15) | 0.80(0.64,0.99) | -1.23(-1.48,-0.97) | -1.86(-2.13,-1.58) |
| Democratic Republic of the Congo | both | 48.06(25.10,76.13) | 0.37(0.27,0.50) | 156.28(77.88,258.06) | 0.48(0.41,0.56) | 3.92(3.53,4.32) | 0.87(0.53,1.22) |
| Denmark | both | 108.72(90.71,130.78) | 5.54(4.54,6.69) | 96.84(84.36,109.23) | 5.18(4.20,6.34) | -0.99(-1.32,-0.65) | -0.60(-0.90,-0.30) |
| Djibouti | both | 1.24(0.57,2.14) | 0.95(0.04,4.78) | 6.88(3.41,11.24) | 1.24(0.49,2.59) | 5.49(5.22,5.76) | 0.62(0.42,0.82) |
| Dominica | both | 0.04(0.04,0.05) | 0.16(0.00,16.99) | 0.15(0.11,0.20) | 0.58(0.00,15.81) | 2.36(1.18,3.56) | 2.71(1.56,3.88) |
| Dominican Republic | both | 2.37(1.87,3.00) | 0.08(0.01,0.30) | 4.58(3.20,6.38) | 0.10(0.03,0.25) | 1.93(1.24,2.63) | 0.43(-0.20,1.06) |
| Ecuador | both | 12.10(10.45,13.76) | 0.29(0.15,0.52) | 53.31(42.45,67.34) | 0.73(0.54,0.95) | 5.06(4.44,5.69) | 3.20(2.62,3.79) |
| Egypt | both | 12.51(9.52,16.00) | 0.06(0.03,0.11) | 60.32(44.03,80.45) | 0.14(0.11,0.19) | 6.14(5.66,6.62) | 3.63(3.20,4.06) |
| El Salvador | both | 16.31(12.91,20.35) | 0.82(0.47,1.36) | 16.44(12.12,21.35) | 0.64(0.37,1.04) | -0.39(-0.75,-0.03) | -1.15(-1.49,-0.82) |
| Equatorial Guinea | both | 0.55(0.32,0.95) | 0.41(0.00,3.88) | 4.11(2.18,7.01) | 0.65(0.18,1.74) | 7.00(6.81,7.19) | 1.63(1.48,1.78) |
| Eritrea | both | 8.60(4.96,15.27) | 0.86(0.39,1.68) | 32.63(16.30,55.86) | 1.33(0.91,1.87) | 4.92(4.70,5.14) | 1.43(1.33,1.53) |
| Estonia | both | 23.19(19.74,26.88) | 4.00(2.53,6.04) | 31.39(25.12,37.27) | 7.24(4.88,10.49) | 1.37(0.03,2.72) | 2.04(0.68,3.42) |
| Eswatini | both | 1.10(0.79,1.50) | 0.40(0.01,2.48) | 2.40(1.40,3.51) | 0.49(0.08,1.65) | 2.82(2.34,3.30) | 1.06(0.66,1.47) |
| Ethiopia | both | 48.10(35.71,76.56) | 0.32(0.24,0.43) | 112.88(70.34,152.08) | 0.31(0.25,0.37) | 2.34(2.11,2.57) | -0.52(-0.68,-0.36) |
| Fiji | both | 0.86(0.65,1.13) | 0.26(0.00,1.83) | 0.33(0.23,0.47) | 0.09(0.00,1.24) | -5.24(-6.67,-3.78) | -5.57(-7.01,-4.11) |
| Finland | both | 76.31(68.63,83.22) | 3.93(3.09,4.95) | 143.43(127.29,159.07) | 8.36(7.03,9.87) | 2.61(2.29,2.92) | 2.90(2.54,3.25) |
| France | both | 322.34(289.49,357.90) | 1.46(1.30,1.62) | 432.31(372.59,499.01) | 2.12(1.93,2.33) | 0.43(0.12,0.74) | 0.72(0.42,1.02) |
| Gabon | both | 2.03(1.25,3.02) | 0.59(0.07,2.23) | 4.31(2.34,6.84) | 0.62(0.18,1.57) | 2.11(1.93,2.29) | -0.13(-0.29,0.03) |
| Gambia | both | 0.18(0.10,0.30) | 0.05(0.00,1.52) | 0.44(0.25,0.70) | 0.04(0.00,0.56) | 3.64(2.22,5.07) | 0.61(-0.79,2.03) |
| Georgia | both | 7.75(6.43,9.39) | 0.36(0.15,0.72) | 7.20(5.25,9.54) | 0.60(0.24,1.28) | 1.18(-0.19,2.58) | 3.17(1.69,4.67) |
| Germany | both | 893.62(823.29,971.44) | 2.81(2.63,3.00) | 715.95(632.77,816.54) | 2.58(2.39,2.78) | -1.98(-2.40,-1.57) | -1.34(-1.65,-1.03) |
| Ghana | both | 2.50(1.48,3.86) | 0.05(0.01,0.16) | 4.15(2.60,6.64) | 0.03(0.01,0.08) | 1.67(1.02,2.33) | -1.54(-2.13,-0.95) |
| Greece | both | 49.71(43.69,56.90) | 1.32(0.98,1.74) | 111.27(97.00,128.26) | 3.72(3.05,4.50) | 0.64(-1.04,2.35) | 1.44(0.02,2.87) |
| Greenland | both | 0.49(0.39,0.65) | 1.77(0.00,24.68) | 0.64(0.44,0.85) | 2.95(0.01,27.28) | 0.81(0.55,1.08) | 2.11(1.79,2.43) |
| Grenada | both | 0.09(0.08,0.11) | 0.31(0.00,14.67) | 0.36(0.30,0.45) | 0.92(0.00,12.22) | 1.24(-0.66,3.17) | 0.54(-1.09,2.19) |
| Guam | both | 0.35(0.24,0.46) | 0.54(0.00,7.49) | 0.05(0.04,0.07) | 0.09(0.00,7.06) | -6.59(-8.00,-5.16) | -6.00(-7.43,-4.55) |
| Guatemala | both | 43.05(38.80,47.55) | 1.56(1.12,2.12) | 129.90(108.97,153.30) | 1.95(1.62,2.32) | 4.06(3.26,4.87) | 0.89(0.06,1.74) |
| Guinea | both | 0.72(0.36,1.13) | 0.04(0.00,0.28) | 1.50(0.79,2.48) | 0.03(0.00,0.14) | 3.36(1.87,4.87) | 0.43(-0.97,1.84) |
| Guinea-Bissau | both | 0.19(0.11,0.30) | 0.05(0.00,1.45) | 0.33(0.19,0.56) | 0.04(0.00,0.62) | 3.36(1.96,4.77) | 0.53(-0.83,1.90) |
| Guyana | both | 0.12(0.10,0.15) | 0.04(0.00,1.49) | 0.70(0.49,0.98) | 0.24(0.00,1.86) | 2.54(0.80,4.31) | 3.01(1.32,4.74) |
| Haiti | both | 7.41(5.42,10.23) | 0.32(0.13,0.66) | 26.69(18.33,37.51) | 0.49(0.32,0.72) | 4.61(4.31,4.91) | 1.57(1.30,1.84) |
| Honduras | both | 18.24(14.03,23.03) | 1.13(0.66,1.81) | 43.76(22.66,72.33) | 1.03(0.75,1.39) | 2.60(2.42,2.78) | -0.64(-0.83,-0.46) |
| Hungary | both | 16.42(13.63,19.15) | 0.43(0.25,0.71) | 19.17(15.12,23.98) | 0.67(0.40,1.06) | 0.36(-0.03,0.74) | 1.07(0.70,1.44) |
| Iceland | both | 3.77(3.29,4.26) | 3.58(0.92,9.60) | 11.17(9.81,12.59) | 8.85(4.43,16.17) | 3.68(3.31,4.04) | 3.02(2.71,3.33) |
| India | both | 1612.78(1400.63,1874.10) | 0.48(0.45,0.50) | 3006.65(2571.63,3475.00) | 0.49(0.48,0.51) | 1.50(1.07,1.94) | -0.45(-0.83,-0.06) |
| Indonesia | both | 70.52(59.08,85.19) | 0.09(0.07,0.12) | 191.44(147.74,257.52) | 0.16(0.14,0.19) | 3.78(3.41,4.16) | 2.43(2.04,2.82) |
| Iran (Islamic Republic of) | both | 1089.62(924.32,1302.31) | 5.49(5.16,5.83) | 1695.07(1492.97,1903.64) | 4.52(4.30,4.75) | 1.76(1.09,2.44) | -0.58(-0.98,-0.18) |
| Iraq | both | 93.78(67.99,130.66) | 1.44(1.16,1.78) | 311.09(212.98,478.37) | 1.85(1.65,2.07) | 3.70(3.17,4.23) | 0.58(0.26,0.90) |
| Ireland | both | 24.32(21.89,26.99) | 1.82(1.17,2.70) | 92.63(82.19,105.92) | 5.77(4.65,7.10) | 3.72(2.59,4.86) | 2.75(1.84,3.68) |
| Israel | both | 19.75(17.19,22.54) | 1.06(0.65,1.65) | 59.69(51.71,68.62) | 1.81(1.38,2.33) | 2.81(1.76,3.87) | 0.87(-0.08,1.84) |
| Italy | both | 873.06(815.25,933.37) | 4.01(3.75,4.29) | 145.30(133.20,158.23) | 0.87(0.73,1.03) | -6.93(-7.35,-6.51) | -6.10(-6.57,-5.64) |
| Jamaica | both | 1.06(0.83,1.37) | 0.12(0.00,0.72) | 1.95(1.43,2.71) | 0.16(0.02,0.62) | -0.53(-1.82,0.78) | -1.26(-2.45,-0.06) |
| Japan | both | 47.00(44.95,49.40) | 0.11(0.08,0.14) | 85.72(80.89,91.04) | 0.25(0.20,0.31) | 1.14(-0.34,2.64) | 2.01(0.73,3.31) |
| Jordan | both | 3.03(2.40,3.71) | 0.22(0.04,0.75) | 6.85(5.16,8.94) | 0.13(0.05,0.28) | 2.06(1.78,2.34) | -2.29(-2.67,-1.90) |
| Kazakhstan | both | 82.81(64.28,105.33) | 1.21(0.97,1.51) | 226.82(169.00,291.55) | 2.98(2.60,3.40) | 2.92(1.10,4.78) | 2.35(0.33,4.42) |
| Kenya | both | 18.38(11.07,23.35) | 0.29(0.17,0.46) | 75.59(49.58,100.92) | 0.42(0.33,0.52) | 4.76(4.62,4.90) | 1.19(1.11,1.27) |
| Kiribati | both | 1.76(1.27,2.36) | 5.60(0.53,24.43) | 3.71(2.42,5.46) | 7.41(1.89,20.05) | 2.78(2.52,3.03) | 0.92(0.77,1.08) |
| Kuwait | both | 3.61(2.89,4.85) | 0.40(0.10,1.21) | 22.34(16.93,30.96) | 0.90(0.55,1.46) | 5.66(4.69,6.64) | 1.56(0.48,2.64) |
| Kyrgyzstan | both | 21.23(16.00,27.55) | 1.21(0.75,1.87) | 50.02(35.15,66.55) | 1.79(1.33,2.36) | 3.60(2.02,5.21) | 2.12(0.48,3.79) |
| Lao People's Democratic Republic | both | 2.32(1.59,3.48) | 0.16(0.02,0.57) | 4.72(3.17,6.73) | 0.15(0.05,0.36) | 1.94(1.77,2.10) | -0.63(-0.80,-0.45) |
| Latvia | both | 23.07(20.08,26.56) | 2.36(1.49,3.57) | 18.92(15.83,22.45) | 3.21(1.90,5.20) | -1.87(-2.60,-1.13) | -0.30(-1.01,0.41) |
| Lebanon | both | 5.46(3.95,7.54) | 0.50(0.17,1.17) | 11.37(8.82,14.84) | 0.45(0.23,0.84) | 2.87(2.36,3.38) | -0.17(-0.39,0.05) |
| Lesotho | both | 1.33(0.92,1.86) | 0.26(0.01,1.28) | 3.51(2.32,4.86) | 0.44(0.10,1.24) | 4.34(3.86,4.83) | 2.81(2.35,3.28) |
| Liberia | both | 0.41(0.22,0.66) | 0.04(0.00,0.56) | 0.71(0.32,1.26) | 0.03(0.00,0.25) | 5.70(4.07,7.36) | 1.32(-0.26,2.94) |
| Libya | both | 24.42(17.41,33.09) | 1.64(1.04,2.47) | 101.77(69.63,148.07) | 3.25(2.65,3.96) | 5.31(4.96,5.67) | 2.50(2.32,2.67) |
| Lithuania | both | 32.87(28.49,38.25) | 2.31(1.59,3.27) | 39.52(32.35,46.11) | 4.47(3.18,6.20) | 0.23(-0.49,0.95) | 2.06(1.34,2.80) |
| Luxembourg | both | 5.70(5.11,6.38) | 3.69(1.29,8.95) | 6.82(5.99,7.70) | 2.94(1.14,6.60) | -0.70(-1.19,-0.21) | -1.92(-2.43,-1.41) |
| Madagascar | both | 26.69(15.90,42.56) | 0.74(0.48,1.08) | 89.26(50.74,141.61) | 0.94(0.76,1.16) | 3.66(3.50,3.83) | 0.51(0.34,0.68) |
| Malawi | both | 21.33(10.90,37.26) | 0.76(0.47,1.16) | 73.25(39.97,121.05) | 1.17(0.92,1.48) | 4.08(3.97,4.19) | 1.30(1.18,1.43) |
| Malaysia | both | 12.26(8.58,15.68) | 0.17(0.09,0.30) | 21.57(15.21,28.47) | 0.15(0.09,0.23) | 1.35(1.12,1.59) | -0.80(-1.03,-0.57) |
| Maldives | both | 0.09(0.05,0.13) | 0.12(0.00,7.72) | 1.14(0.72,1.66) | 0.36(0.01,3.15) | 9.31(8.91,9.71) | 4.20(3.88,4.52) |
| Mali | both | 1.05(0.53,1.72) | 0.04(0.00,0.22) | 2.43(1.10,3.91) | 0.03(0.00,0.10) | 4.14(2.54,5.77) | 0.39(-1.05,1.86) |
| Malta | both | 1.56(1.35,1.80) | 1.13(0.09,5.09) | 3.55(2.94,4.29) | 2.49(0.58,7.73) | 2.67(2.10,3.25) | 2.23(1.70,2.77) |
| Marshall Islands | both | 0.05(0.03,0.08) | 0.30(0.00,27.78) | 0.05(0.03,0.07) | 0.19(0.00,16.90) | -0.88(-1.04,-0.72) | -1.81(-2.07,-1.55) |
| Mauritania | both | 0.38(0.24,0.57) | 0.05(0.00,0.70) | 0.54(0.29,0.87) | 0.03(0.00,0.34) | 2.32(0.84,3.82) | -0.21(-1.64,1.24) |
| Mauritius | both | 0.30(0.25,0.37) | 0.06(0.00,0.92) | 9.55(7.82,11.46) | 2.04(0.96,3.85) | 9.02(6.32,11.79) | 9.30(6.70,11.96) |
| Mexico | both | 182.30(172.74,192.78) | 0.53(0.45,0.61) | 331.81(298.67,371.31) | 0.64(0.58,0.72) | 0.88(0.22,1.54) | -0.33(-0.93,0.26) |
| Micronesia (Federated States of) | both | 0.12(0.08,0.18) | 0.31(0.00,12.00) | 0.08(0.05,0.12) | 0.18(0.00,10.64) | -1.95(-2.21,-1.69) | -2.01(-2.28,-1.74) |
| Monaco | both | 0.01(0.01,0.01) | 0.09(0.00,61.41) | 0.04(0.02,0.06) | 0.42(0.00,45.93) | 4.17(3.20,5.14) | 4.35(3.36,5.35) |
| Mongolia | both | 2.67(1.45,4.44) | 0.32(0.05,1.13) | 10.49(6.79,15.45) | 0.80(0.39,1.48) | 5.61(4.45,6.78) | 4.16(3.11,5.21) |
| Montenegro | both | 0.64(0.48,0.83) | 0.25(0.00,2.01) | 0.62(0.43,0.85) | 0.29(0.00,2.60) | 0.20(-0.09,0.49) | 0.74(0.44,1.05) |
| Morocco | both | 127.78(91.88,174.59) | 1.31(1.09,1.56) | 274.36(168.34,452.08) | 1.85(1.64,2.08) | 2.22(1.93,2.50) | 0.83(0.63,1.03) |
| Mozambique | both | 13.20(7.67,19.75) | 0.33(0.18,0.57) | 61.65(38.31,96.23) | 0.69(0.53,0.89) | 5.52(5.37,5.66) | 2.69(2.60,2.78) |
| Myanmar | both | 28.49(20.07,40.98) | 0.17(0.11,0.25) | 30.40(20.43,43.16) | 0.14(0.09,0.20) | -0.44(-0.71,-0.18) | -1.25(-1.48,-1.02) |
| Namibia | both | 1.90(1.19,2.77) | 0.37(0.04,1.55) | 3.84(2.22,6.09) | 0.38(0.10,1.02) | 2.21(2.14,2.28) | 0.12(0.01,0.23) |
| Nauru | both | 0.01(0.01,0.02) | 0.33(0.00,105.47) | 0.01(0.01,0.02) | 0.19(0.00,91.15) | -1.76(-2.03,-1.48) | -2.08(-2.36,-1.80) |
| Nepal | both | 22.11(14.80,31.43) | 0.30(0.19,0.47) | 48.91(31.35,70.64) | 0.37(0.27,0.49) | 2.54(2.48,2.60) | 0.56(0.45,0.66) |
| Netherlands | both | 47.58(40.48,54.88) | 0.76(0.56,1.02) | 86.87(74.20,102.28) | 1.60(1.28,1.97) | 1.27(0.89,1.65) | 1.97(1.67,2.28) |
| New Zealand | both | 17.00(14.73,19.48) | 1.23(0.72,1.97) | 50.85(44.96,57.77) | 2.72(2.02,3.60) | 2.80(2.41,3.20) | 2.07(1.76,2.38) |
| Nicaragua | both | 3.09(2.50,3.75) | 0.22(0.04,0.70) | 5.84(4.55,7.31) | 0.21(0.07,0.46) | 1.71(1.20,2.23) | -0.55(-0.96,-0.13) |
| Niger | both | 0.89(0.41,1.57) | 0.03(0.00,0.24) | 1.93(0.67,3.51) | 0.02(0.00,0.10) | 3.68(2.20,5.18) | 0.01(-1.41,1.45) |
| Nigeria | both | 13.37(8.20,18.80) | 0.04(0.02,0.07) | 27.64(11.95,41.37) | 0.03(0.02,0.05) | 3.78(2.33,5.25) | 0.68(-0.79,2.17) |
| Niue | both | 0.00(0.00,0.00) | 0.32(0.00,518.81) | 0.00(0.00,0.00) | 0.20(0.00,703.62) | -3.58(-4.15,-3.00) | -2.16(-2.46,-1.86) |
| North Macedonia | both | 4.07(2.87,5.85) | 0.51(0.14,1.30) | 5.25(3.39,7.98) | 0.66(0.22,1.65) | 1.48(0.83,2.13) | 1.27(0.67,1.86) |
| Northern Mariana Islands | both | 0.07(0.05,0.10) | 0.30(0.00,20.45) | 0.02(0.01,0.03) | 0.13(0.00,26.24) | -6.50(-8.33,-4.63) | -4.29(-5.97,-2.58) |
| Norway | both | 55.71(51.32,60.31) | 3.42(2.58,4.45) | 107.14(99.74,115.40) | 5.84(4.78,7.09) | 0.72(-0.52,1.96) | 0.37(-0.94,1.70) |
| Oman | both | 3.14(1.96,4.70) | 0.36(0.08,1.09) | 8.98(6.18,12.82) | 0.33(0.14,0.73) | 4.30(3.94,4.66) | 0.21(-0.11,0.52) |
| Pakistan | both | 161.99(114.54,207.50) | 0.41(0.35,0.48) | 516.08(391.46,676.51) | 0.52(0.48,0.57) | 3.58(3.40,3.75) | 0.50(0.33,0.68) |
| Palau | both | 0.00(0.00,0.00) | 0.01(0.00,59.04) | 0.00(0.00,0.00) | 0.01(0.00,69.25) | 0.48(0.02,0.94) | 1.59(1.30,1.87) |
| Palestine | both | 0.19(0.12,0.28) | 0.03(0.00,0.84) | 0.61(0.42,0.86) | 0.03(0.00,0.27) | 3.41(3.16,3.65) | -0.22(-0.42,-0.02) |
| Panama | both | 3.99(3.41,4.56) | 0.40(0.11,1.08) | 8.61(6.62,10.71) | 0.52(0.23,1.01) | 1.75(1.24,2.26) | 0.28(-0.19,0.75) |
| Papua New Guinea | both | 3.24(1.14,6.19) | 0.19(0.04,0.59) | 5.65(2.85,9.45) | 0.13(0.05,0.30) | 1.44(1.03,1.84) | -1.70(-2.11,-1.28) |
| Paraguay | both | 0.76(0.63,0.92) | 0.05(0.00,0.37) | 7.97(5.81,10.46) | 0.26(0.11,0.52) | 9.02(8.49,9.55) | 6.58(6.10,7.06) |
| Peru | both | 46.90(37.46,57.83) | 0.52(0.38,0.70) | 85.50(63.26,110.85) | 0.58(0.46,0.71) | 1.98(1.53,2.44) | 0.45(-0.01,0.91) |
| Philippines | both | 32.82(29.30,37.27) | 0.13(0.09,0.19) | 62.94(52.77,73.24) | 0.14(0.10,0.17) | 1.70(1.44,1.96) | -0.34(-0.60,-0.09) |
| Poland | both | 145.40(134.36,157.61) | 0.99(0.83,1.17) | 161.60(147.14,178.86) | 1.28(1.09,1.51) | -0.31(-1.08,0.48) | -0.15(-1.06,0.77) |
| Portugal | both | 54.24(47.09,61.88) | 1.45(1.09,1.89) | 45.71(39.61,52.83) | 1.49(1.09,2.00) | -2.81(-3.59,-2.03) | -2.16(-2.93,-1.39) |
| Puerto Rico | both | 11.47(10.21,12.90) | 0.83(0.42,1.47) | 46.65(38.01,57.25) | 4.37(3.21,5.84) | -1.09(-3.94,1.85) | -0.24(-3.00,2.60) |
| Qatar | both | 0.65(0.44,0.92) | 0.25(0.00,3.22) | 4.00(2.55,5.67) | 0.20(0.04,1.01) | 7.94(6.91,8.99) | -0.59(-1.08,-0.11) |
| Republic of Korea | both | 49.55(42.94,56.27) | 0.24(0.17,0.31) | 32.22(25.79,40.75) | 0.18(0.13,0.27) | -2.98(-3.75,-2.21) | -2.34(-3.06,-1.62) |
| Republic of Moldova | both | 18.08(15.67,20.88) | 1.02(0.60,1.63) | 17.75(13.81,21.76) | 1.30(0.75,2.19) | -0.67(-1.15,-0.18) | -0.06(-0.57,0.46) |
| Romania | both | 109.95(77.91,143.33) | 1.27(1.05,1.54) | 58.37(46.19,72.15) | 1.06(0.80,1.38) | -2.60(-2.84,-2.36) | -1.16(-1.43,-0.89) |
| Russian Federation | both | 2350.52(2180.38,2511.27) | 3.90(3.75,4.07) | 3848.78(3499.86,4203.21) | 7.17(6.94,7.41) | 0.99(-0.38,2.38) | 1.07(-0.32,2.48) |
| Rwanda | both | 26.07(16.38,40.64) | 1.13(0.74,1.66) | 60.79(31.31,102.82) | 1.22(0.93,1.57) | 2.79(2.13,3.44) | -0.21(-0.49,0.07) |
| Saint Kitts and Nevis | both | 0.04(0.03,0.04) | 0.23(0.00,27.19) | 0.05(0.03,0.06) | 0.20(0.00,19.54) | -1.87(-3.11,-0.61) | -3.04(-4.23,-1.85) |
| Saint Lucia | both | 0.04(0.04,0.05) | 0.08(0.00,9.53) | 0.17(0.14,0.22) | 0.25(0.00,7.04) | 1.36(-0.94,3.72) | 0.35(-1.77,2.52) |
| Saint Vincent and the Grenadines | both | 0.02(0.02,0.02) | 0.05(0.00,12.01) | 0.07(0.05,0.09) | 0.17(0.00,9.69) | -0.89(-3.20,1.48) | -0.86(-3.07,1.41) |
| Samoa | both | 0.19(0.11,0.27) | 0.27(0.00,8.80) | 0.13(0.08,0.19) | 0.16(0.00,5.92) | -1.69(-2.16,-1.21) | -2.06(-2.44,-1.68) |
| San Marino | both | 0.01(0.01,0.02) | 0.15(0.00,43.18) | 0.01(0.01,0.02) | 0.16(0.00,44.23) | 0.55(0.21,0.89) | 1.08(0.78,1.37) |
| Sao Tome and Principe | both | 0.05(0.02,0.08) | 0.11(0.00,13.90) | 0.24(0.13,0.39) | 0.27(0.00,5.17) | 5.36(5.06,5.67) | 2.83(2.57,3.09) |
| Saudi Arabia | both | 5.11(3.36,7.19) | 0.08(0.03,0.20) | 38.76(24.80,58.35) | 0.18(0.13,0.25) | 8.03(7.24,8.82) | 3.46(2.82,4.11) |
| Senegal | both | 1.23(0.72,2.06) | 0.04(0.00,0.26) | 2.58(1.35,4.29) | 0.04(0.01,0.14) | 3.97(2.50,5.46) | 1.22(-0.29,2.76) |
| Serbia | both | 35.16(26.51,46.17) | 0.97(0.68,1.35) | 33.52(23.95,44.87) | 1.07(0.74,1.52) | 0.28(-0.34,0.90) | 0.92(0.20,1.64) |
| Seychelles | both | 0.10(0.09,0.12) | 0.35(0.00,16.85) | 0.25(0.20,0.32) | 0.61(0.00,12.41) | 2.47(1.55,3.41) | 1.47(0.66,2.28) |
| Sierra Leone | both | 0.52(0.28,0.89) | 0.03(0.00,0.35) | 1.04(0.49,1.78) | 0.03(0.00,0.18) | 4.47(2.42,6.57) | 1.11(-0.60,2.85) |
| Singapore | both | 2.11(1.81,2.43) | 0.13(0.02,0.52) | 3.34(2.84,3.93) | 0.16(0.03,0.57) | -0.26(-1.41,0.91) | -1.62(-2.64,-0.58) |
| Slovakia | both | 15.93(11.70,21.54) | 0.78(0.44,1.27) | 13.72(9.24,20.04) | 0.76(0.40,1.34) | -0.25(-0.50,0.01) | -0.06(-0.26,0.14) |
| Slovenia | both | 9.69(7.96,11.80) | 1.24(0.58,2.35) | 9.31(7.20,11.86) | 1.54(0.70,3.05) | -0.02(-0.59,0.55) | 0.62(0.08,1.16) |
| Solomon Islands | both | 0.28(0.14,0.50) | 0.22(0.00,4.65) | 0.42(0.25,0.66) | 0.15(0.00,1.85) | 0.65(0.31,0.99) | -1.60(-1.97,-1.24) |
| Somalia | both | 19.47(9.71,38.10) | 0.76(0.46,1.19) | 52.87(24.84,99.96) | 0.85(0.64,1.12) | 4.52(3.89,5.16) | 0.43(0.29,0.56) |
| South Africa | both | 273.65(240.85,314.45) | 1.84(1.63,2.08) | 318.61(278.27,363.26) | 1.27(1.13,1.42) | 0.24(-0.78,1.27) | -1.38(-2.36,-0.39) |
| South Sudan | both | 11.81(5.97,20.86) | 0.70(0.36,1.25) | 22.23(11.97,38.09) | 0.80(0.50,1.21) | 2.36(1.99,2.73) | 0.35(0.16,0.55) |
| Spain | both | 505.04(448.58,572.36) | 3.38(3.09,3.69) | 227.02(199.14,262.16) | 1.72(1.50,1.97) | -4.32(-4.89,-3.75) | -4.26(-4.85,-3.68) |
| Sri Lanka | both | 86.51(70.87,105.08) | 1.19(0.96,1.48) | 44.16(31.35,58.96) | 0.55(0.40,0.73) | -3.97(-4.79,-3.14) | -4.31(-5.13,-3.49) |
| Sudan | both | 95.15(65.54,139.63) | 1.37(1.10,1.68) | 374.30(232.67,539.40) | 2.14(1.93,2.37) | 4.34(4.16,4.52) | 1.38(1.21,1.55) |
| Suriname | both | 0.27(0.18,0.34) | 0.18(0.00,3.54) | 1.56(1.11,2.18) | 0.72(0.06,3.08) | 3.22(1.34,5.13) | 1.98(0.32,3.66) |
| Sweden | both | 57.02(50.87,63.50) | 1.88(1.42,2.45) | 212.04(185.82,237.59) | 6.30(5.47,7.22) | 5.11(4.57,5.65) | 4.79(4.23,5.36) |
| Switzerland | both | 258.70(214.42,298.43) | 9.32(8.21,10.55) | 87.96(78.22,97.98) | 3.00(2.39,3.73) | -4.16(-4.57,-3.75) | -4.36(-4.71,-4.02) |
| Syrian Arab Republic | both | 40.52(31.18,52.35) | 0.91(0.64,1.25) | 53.30(38.04,74.15) | 1.14(0.83,1.56) | 1.56(0.44,2.69) | 1.18(0.86,1.51) |
| Taiwan (Province of China) | both | 186.92(166.77,205.88) | 1.99(1.71,2.30) | 122.55(105.20,141.01) | 1.43(1.19,1.72) | -1.49(-1.83,-1.14) | -1.36(-1.78,-0.93) |
| Tajikistan | both | 6.83(5.11,8.88) | 0.34(0.13,0.75) | 18.73(10.09,30.81) | 0.44(0.26,0.70) | 3.63(3.31,3.95) | 1.08(0.88,1.28) |
| Thailand | both | 28.27(21.40,35.61) | 0.11(0.07,0.16) | 49.18(35.82,65.95) | 0.22(0.16,0.29) | 1.32(0.81,1.83) | 1.88(1.50,2.25) |
| Timor-Leste | both | 0.38(0.23,0.56) | 0.12(0.00,1.70) | 0.65(0.42,0.90) | 0.12(0.00,1.20) | 1.22(0.39,2.06) | -0.38(-1.00,0.24) |
| Togo | both | 0.56(0.33,0.89) | 0.04(0.00,0.45) | 1.05(0.56,1.76) | 0.03(0.00,0.18) | 3.19(1.84,4.57) | 0.32(-1.06,1.72) |
| Tokelau | both | 0.00(0.00,0.00) | 0.32(0.00,774.15) | 0.00(0.00,0.00) | 0.25(0.00,821.99) | -2.39(-3.11,-1.67) | -1.70(-2.16,-1.24) |
| Tonga | both | 0.07(0.03,0.11) | 0.20(0.00,15.79) | 0.06(0.03,0.09) | 0.15(0.00,11.67) | -1.15(-1.32,-0.98) | -1.18(-1.36,-1.00) |
| Trinidad and Tobago | both | 0.49(0.43,0.54) | 0.10(0.00,1.02) | 3.30(2.44,4.35) | 0.61(0.13,1.84) | 3.52(1.50,5.57) | 3.13(1.17,5.13) |
| Tunisia | both | 41.82(28.35,58.23) | 1.30(0.93,1.78) | 89.88(62.28,126.41) | 1.95(1.56,2.41) | 2.29(1.89,2.70) | 1.09(0.87,1.32) |
| Türkiye | both | 96.63(73.82,127.69) | 0.42(0.34,0.51) | 198.44(148.66,255.93) | 0.61(0.53,0.71) | 2.65(2.25,3.05) | 1.63(1.13,2.13) |
| Turkmenistan | both | 7.02(5.50,8.84) | 0.47(0.19,1.01) | 60.31(40.34,86.80) | 2.87(2.19,3.69) | 8.37(7.17,9.59) | 7.23(6.03,8.45) |
| Tuvalu | both | 0.01(0.01,0.02) | 0.28(0.00,108.91) | 0.01(0.01,0.01) | 0.20(0.00,84.75) | -0.40(-0.78,-0.02) | -1.58(-1.83,-1.32) |
| Uganda | both | 28.59(13.11,50.11) | 0.63(0.42,0.92) | 132.04(75.32,206.26) | 1.05(0.88,1.25) | 4.63(4.43,4.83) | 1.29(1.14,1.44) |
| Ukraine | both | 339.79(282.99,396.52) | 1.69(1.52,1.88) | 440.81(302.36,609.20) | 2.82(2.56,3.11) | 0.06(-0.91,1.03) | 0.68(-0.30,1.66) |
| United Arab Emirates | both | 7.87(5.33,11.17) | 0.74(0.31,1.68) | 33.54(21.74,46.10) | 0.85(0.51,1.36) | 6.86(5.56,8.17) | 0.88(0.53,1.22) |
| United Kingdom | both | 517.86(504.42,531.55) | 2.43(2.23,2.66) | 1678.64(1619.97,1742.82) | 7.26(6.91,7.61) | 3.04(2.34,3.75) | 2.75(1.99,3.52) |
| United Republic of Tanzania | both | 74.75(38.06,123.06) | 1.03(0.81,1.29) | 257.98(134.10,431.29) | 1.38(1.22,1.56) | 4.10(4.04,4.16) | 0.91(0.86,0.95) |
| United States of America | both | 3545.21(3338.46,3757.25) | 3.24(3.13,3.35) | 31106.91(28842.42,33786.12) | 26.86(26.57,27.16) | 7.75(7.46,8.04) | 7.64(7.33,7.95) |
| United States Virgin Islands | both | 0.84(0.63,1.09) | 2.15(0.03,14.20) | 1.19(0.76,1.82) | 5.08(0.20,27.37) | 1.31(0.95,1.67) | 3.02(2.58,3.45) |
| Uruguay | both | 1.20(1.06,1.35) | 0.11(0.00,0.53) | 4.63(4.01,5.39) | 0.38(0.12,0.94) | 4.93(4.37,5.50) | 4.72(4.18,5.26) |
| Uzbekistan | both | 22.32(18.38,27.12) | 0.27(0.17,0.41) | 76.99(60.58,96.46) | 0.54(0.42,0.67) | 5.55(4.75,6.36) | 3.62(2.87,4.38) |
| Vanuatu | both | 0.13(0.08,0.21) | 0.22(0.00,8.29) | 0.19(0.11,0.29) | 0.15(0.00,3.81) | 0.60(0.29,0.90) | -1.86(-2.20,-1.52) |
| Venezuela (Bolivarian Republic of) | both | 13.44(11.22,15.92) | 0.17(0.09,0.29) | 18.43(12.59,25.52) | 0.20(0.12,0.32) | 2.22(1.36,3.09) | 1.02(0.50,1.55) |
| Viet Nam | both | 321.89(220.91,462.58) | 1.18(1.05,1.32) | 713.66(461.22,1103.27) | 1.74(1.61,1.87) | 2.44(2.30,2.58) | 1.06(0.85,1.27) |
| Yemen | both | 34.51(16.98,63.05) | 0.84(0.58,1.18) | 181.93(96.10,293.86) | 1.41(1.21,1.63) | 5.76(5.22,6.31) | 1.65(1.19,2.10) |
| Zambia | both | 21.08(12.85,32.54) | 1.00(0.62,1.55) | 94.24(53.62,155.61) | 1.44(1.17,1.77) | 5.01(4.82,5.20) | 1.14(1.06,1.22) |
| Zimbabwe | both | 14.05(9.80,19.00) | 0.39(0.21,0.68) | 36.09(25.56,50.62) | 0.60(0.42,0.83) | 3.04(2.78,3.30) | 1.39(1.12,1.67) |
| Afghanistan | female | 27.73(18.71,39.44) | 1.84(1.17,2.83) | 112.83(76.93,164.90) | 2.05(1.67,2.50) | 3.96(3.36,4.56) | 0.10(-0.15,0.35) |
| Albania | female | 3.10(2.39,3.86) | 0.44(0.09,1.37) | 1.55(1.11,2.16) | 0.34(0.03,1.55) | -1.90(-2.63,-1.17) | -0.40(-1.14,0.36) |
| Algeria | female | 29.53(20.85,42.71) | 0.65(0.43,0.95) | 63.43(43.67,89.47) | 0.71(0.55,0.92) | 2.38(2.02,2.75) | 0.14(-0.06,0.34) |
| American Samoa | female | 0.00(0.00,0.01) | 0.05(0.00,47.87) | 0.00(0.00,0.01) | 0.04(0.00,49.01) | -1.93(-4.47,0.68) | -1.42(-3.93,1.15) |
| Andorra | female | 0.01(0.00,0.01) | 0.04(0.00,42.81) | 0.00(0.00,0.01) | 0.04(0.00,40.29) | -0.73(-1.08,-0.38) | -0.47(-0.63,-0.31) |
| Angola | female | 1.32(0.83,1.99) | 0.07(0.00,0.36) | 4.39(2.67,6.49) | 0.07(0.02,0.18) | 4.11(3.88,4.35) | 0.10(-0.12,0.32) |
| Antigua and Barbuda | female | 0.01(0.01,0.02) | 0.11(0.00,31.62) | 0.02(0.02,0.03) | 0.14(0.00,25.84) | 0.28(-1.12,1.70) | -0.55(-1.67,0.57) |
| Argentina | female | 1.15(0.99,1.33) | 0.02(0.00,0.10) | 7.25(6.01,8.52) | 0.08(0.03,0.17) | 7.40(6.09,8.73) | 5.98(4.70,7.27) |
| Armenia | female | 0.75(0.62,0.89) | 0.10(0.00,0.77) | 0.47(0.36,0.58) | 0.09(0.00,1.15) | 1.19(-0.08,2.47) | 2.02(0.81,3.24) |
| Australia | female | 80.27(73.74,87.30) | 2.37(1.88,2.95) | 147.48(127.66,168.36) | 3.15(2.66,3.72) | 1.06(0.45,1.67) | 0.09(-0.52,0.70) |
| Austria | female | 8.73(7.57,9.93) | 0.58(0.26,1.15) | 15.43(13.43,17.69) | 1.08(0.60,1.82) | 1.52(0.99,2.06) | 1.71(1.06,2.36) |
| Azerbaijan | female | 3.12(2.49,3.84) | 0.19(0.04,0.59) | 3.09(2.11,4.17) | 0.15(0.03,0.49) | 0.38(-0.11,0.87) | -0.46(-0.88,-0.05) |
| Bahamas | female | 0.08(0.06,0.09) | 0.13(0.00,7.80) | 0.18(0.13,0.25) | 0.23(0.00,5.34) | 0.80(-0.16,1.77) | -0.04(-0.90,0.82) |
| Bahrain | female | 0.11(0.09,0.14) | 0.11(0.00,4.86) | 0.16(0.11,0.21) | 0.06(0.00,1.87) | 2.18(1.56,2.80) | -1.30(-1.68,-0.91) |
| Bangladesh | female | 44.89(30.88,62.73) | 0.21(0.15,0.29) | 57.29(37.86,81.44) | 0.16(0.12,0.21) | 0.94(0.69,1.19) | -0.70(-1.05,-0.34) |
| Barbados | female | 0.04(0.03,0.05) | 0.07(0.00,7.40) | 0.09(0.07,0.12) | 0.17(0.00,8.88) | 1.22(0.21,2.23) | 1.49(0.43,2.57) |
| Belarus | female | 24.63(18.85,40.78) | 1.22(0.78,1.83) | 17.03(12.74,23.08) | 1.09(0.62,1.84) | -1.96(-2.86,-1.06) | -1.25(-2.12,-0.37) |
| Belgium | female | 12.42(10.90,13.97) | 0.66(0.35,1.17) | 26.30(23.02,29.55) | 1.46(0.95,2.16) | 1.95(1.69,2.22) | 2.05(1.74,2.37) |
| Belize | female | 0.02(0.01,0.02) | 0.05(0.00,15.36) | 0.10(0.08,0.13) | 0.11(0.00,4.54) | 4.27(2.59,5.98) | 0.86(-0.74,2.48) |
| Benin | female | 0.01(0.00,0.01) | 0.00(0.00,0.53) | 0.01(0.00,0.01) | 0.00(0.00,0.19) | 1.00(-1.11,3.16) | -2.13(-4.20,-0.03) |
| Bermuda | female | 0.06(0.05,0.07) | 0.41(0.00,41.91) | 0.04(0.03,0.05) | 0.38(0.00,54.13) | -3.24(-4.38,-2.09) | -2.03(-3.11,-0.93) |
| Bhutan | female | 0.21(0.11,0.33) | 0.17(0.00,4.68) | 0.19(0.11,0.30) | 0.12(0.00,2.63) | -0.50(-0.64,-0.36) | -1.67(-1.87,-1.48) |
| Bolivia (Plurinational State of) | female | 8.20(5.74,11.72) | 0.62(0.27,1.25) | 13.64(8.35,21.57) | 0.56(0.30,0.94) | 1.48(1.11,1.85) | -0.60(-0.80,-0.41) |
| Bosnia and Herzegovina | female | 0.55(0.42,0.70) | 0.06(0.00,0.54) | 0.35(0.25,0.47) | 0.07(0.00,1.07) | -1.02(-1.43,-0.61) | 0.61(0.29,0.93) |
| Botswana | female | 0.90(0.57,1.38) | 0.33(0.00,2.43) | 1.34(0.78,2.02) | 0.25(0.01,1.19) | 2.66(1.57,3.77) | 0.42(-0.70,1.56) |
| Brazil | female | 31.00(28.87,33.69) | 0.10(0.07,0.14) | 200.68(187.12,215.88) | 0.47(0.41,0.54) | 6.58(5.98,7.19) | 5.55(4.88,6.22) |
| Brunei Darussalam | female | 0.31(0.22,0.43) | 0.54(0.00,8.42) | 0.30(0.21,0.42) | 0.30(0.00,5.54) | 0.52(0.22,0.82) | -1.15(-1.47,-0.83) |
| Bulgaria | female | 3.35(2.83,3.96) | 0.23(0.05,0.64) | 2.89(2.23,3.66) | 0.31(0.06,1.02) | -0.53(-1.12,0.05) | 0.89(0.46,1.33) |
| Burkina Faso | female | 0.01(0.01,0.02) | 0.00(0.00,0.28) | 0.01(0.01,0.02) | 0.00(0.00,0.10) | 1.08(-1.13,3.35) | -2.07(-4.20,0.10) |
| Burundi | female | 1.84(1.12,2.97) | 0.18(0.02,0.76) | 3.52(2.21,5.49) | 0.15(0.04,0.41) | 2.10(1.55,2.65) | -1.18(-1.43,-0.93) |
| Cabo Verde | female | 0.00(0.00,0.00) | 0.00(0.00,8.28) | 0.00(0.00,0.00) | 0.00(0.00,3.29) | 0.78(0.41,1.16) | -1.27(-1.60,-0.93) |
| Cambodia | female | 2.02(1.34,3.24) | 0.10(0.01,0.38) | 1.76(1.09,2.75) | 0.05(0.00,0.19) | -1.01(-1.23,-0.78) | -2.80(-3.04,-2.57) |
| Cameroon | female | 0.02(0.01,0.04) | 0.00(0.00,0.26) | 0.03(0.01,0.04) | 0.00(0.00,0.07) | 1.74(-0.21,3.73) | -2.09(-3.97,-0.16) |
| Canada | female | 58.49(50.81,67.50) | 0.98(0.75,1.28) | 443.11(393.41,494.88) | 7.12(6.46,7.82) | 6.56(6.10,7.03) | 6.57(6.22,6.92) |
| Central African Republic | female | 0.35(0.25,0.50) | 0.07(0.00,1.09) | 0.82(0.50,1.28) | 0.07(0.00,0.54) | 2.77(2.63,2.90) | 0.24(0.11,0.37) |
| Chad | female | 0.01(0.00,0.01) | 0.00(0.00,0.42) | 0.01(0.00,0.01) | 0.00(0.00,0.16) | 2.17(0.10,4.29) | -1.13(-3.11,0.89) |
| Chile | female | 1.23(1.08,1.40) | 0.04(0.00,0.22) | 7.10(6.13,8.09) | 0.20(0.08,0.42) | 6.42(5.25,7.60) | 5.77(4.62,6.93) |
| China | female | 6550.76(5187.34,8241.47) | 2.51(2.44,2.57) | 911.56(674.54,1212.37) | 0.39(0.37,0.42) | -7.91(-8.61,-7.22) | -7.59(-8.42,-6.75) |
| Colombia | female | 24.88(22.20,28.32) | 0.34(0.22,0.50) | 26.51(21.46,32.49) | 0.27(0.17,0.39) | 0.12(-0.48,0.72) | -0.73(-1.37,-0.08) |
| Comoros | female | 0.14(0.06,0.24) | 0.19(0.00,6.27) | 0.33(0.20,0.51) | 0.22(0.00,3.14) | 2.08(1.49,2.67) | 0.01(-0.58,0.59) |
| Congo | female | 0.41(0.26,0.59) | 0.09(0.00,1.24) | 1.27(0.81,1.82) | 0.11(0.01,0.56) | 3.48(3.02,3.95) | 0.41(0.04,0.78) |
| Cook Islands | female | 0.00(0.00,0.00) | 0.00(0.00,119.47) | 0.00(0.00,0.00) | 0.00(0.00,126.27) | -6.96(-8.62,-5.26) | -6.31(-7.99,-4.59) |
| Costa Rica | female | 0.89(0.76,1.11) | 0.14(0.00,0.93) | 2.25(1.87,2.64) | 0.23(0.03,0.83) | 2.50(2.10,2.90) | 1.14(0.76,1.53) |
| Croatia | female | 3.97(3.41,4.57) | 0.44(0.12,1.18) | 2.92(2.26,3.70) | 0.46(0.09,1.52) | -0.72(-1.11,-0.33) | 0.40(-0.04,0.83) |
| Cuba | female | 3.28(2.28,3.91) | 0.14(0.03,0.41) | 0.84(0.66,1.02) | 0.05(0.00,0.34) | -5.30(-5.61,-4.98) | -4.06(-4.28,-3.85) |
| Cyprus | female | 0.68(0.47,0.96) | 0.45(0.00,3.56) | 0.83(0.57,1.17) | 0.30(0.00,2.94) | 0.36(-0.22,0.94) | -1.80(-2.41,-1.18) |
| Czechia | female | 6.38(5.38,7.57) | 0.35(0.13,0.76) | 6.40(5.15,8.06) | 0.43(0.16,0.98) | 0.22(-0.23,0.68) | 0.78(0.46,1.11) |
| C?te d'Ivoire | female | 0.02(0.01,0.02) | 0.00(0.00,0.24) | 0.01(0.01,0.02) | 0.00(0.00,0.08) | 2.04(0.04,4.08) | -0.64(-2.68,1.45) |
| Democratic People's Republic of Korea | female | 42.85(27.07,64.80) | 0.99(0.71,1.34) | 31.25(19.59,46.99) | 0.63(0.43,0.91) | -1.60(-1.85,-1.35) | -1.80(-2.06,-1.54) |
| Democratic Republic of the Congo | female | 4.44(2.91,6.47) | 0.06(0.02,0.17) | 12.04(7.42,18.10) | 0.07(0.04,0.12) | 3.15(2.84,3.46) | 0.15(-0.11,0.40) |
| Denmark | female | 16.23(14.09,18.49) | 1.70(0.98,2.78) | 19.43(17.30,21.79) | 2.13(1.29,3.35) | 0.19(-0.08,0.46) | 0.55(0.34,0.75) |
| Djibouti | female | 0.07(0.04,0.12) | 0.10(0.00,6.93) | 0.30(0.16,0.53) | 0.12(0.00,1.81) | 4.92(4.44,5.41) | 0.38(0.06,0.70) |
| Dominica | female | 0.03(0.02,0.03) | 0.19(0.00,35.60) | 0.07(0.05,0.10) | 0.55(0.00,31.56) | 2.27(1.58,2.96) | 2.53(1.86,3.20) |
| Dominican Republic | female | 1.32(0.96,1.80) | 0.09(0.00,0.46) | 2.51(1.60,3.80) | 0.11(0.02,0.36) | 2.18(1.76,2.60) | 0.96(0.58,1.34) |
| Ecuador | female | 5.24(4.43,6.25) | 0.24(0.08,0.58) | 13.84(10.40,17.50) | 0.38(0.21,0.64) | 3.24(2.26,4.22) | 1.64(0.71,2.57) |
| Egypt | female | 2.10(1.53,2.67) | 0.02(0.00,0.08) | 8.11(5.54,11.25) | 0.04(0.02,0.08) | 5.85(5.22,6.48) | 3.52(2.93,4.11) |
| El Salvador | female | 1.30(1.05,1.60) | 0.12(0.01,0.65) | 1.83(1.34,2.39) | 0.13(0.01,0.53) | 1.11(0.84,1.38) | 0.44(0.20,0.67) |
| Equatorial Guinea | female | 0.06(0.04,0.09) | 0.08(0.00,5.64) | 0.24(0.12,0.41) | 0.08(0.00,1.62) | 4.20(3.96,4.44) | -0.29(-0.51,-0.07) |
| Eritrea | female | 0.88(0.55,1.31) | 0.15(0.00,1.01) | 2.37(1.34,3.81) | 0.19(0.03,0.64) | 4.02(3.75,4.29) | 1.01(0.86,1.17) |
| Estonia | female | 3.80(3.27,4.40) | 1.33(0.34,3.64) | 2.21(1.74,2.78) | 1.17(0.15,4.48) | -1.85(-2.63,-1.06) | -0.80(-1.55,-0.04) |
| Eswatini | female | 0.62(0.42,0.90) | 0.38(0.00,3.92) | 1.10(0.52,1.81) | 0.42(0.01,2.37) | 2.42(1.68,3.18) | 1.09(0.40,1.79) |
| Ethiopia | female | 21.43(15.77,30.60) | 0.25(0.15,0.38) | 25.22(17.70,36.43) | 0.12(0.08,0.18) | -0.40(-0.78,-0.02) | -3.21(-3.57,-2.84) |
| Fiji | female | 0.33(0.23,0.46) | 0.21(0.00,3.14) | 0.02(0.02,0.04) | 0.01(0.00,2.18) | -12.96(-15.94,-9.86) | -13.27(-16.22,-10.21) |
| Finland | female | 13.92(12.22,15.57) | 1.50(0.81,2.60) | 30.47(27.27,34.00) | 3.69(2.49,5.31) | 2.99(2.77,3.21) | 3.32(3.10,3.55) |
| France | female | 83.24(75.87,91.08) | 0.75(0.60,0.94) | 126.74(113.10,142.46) | 1.25(1.04,1.49) | 1.22(1.05,1.40) | 1.51(1.33,1.68) |
| Gabon | female | 0.13(0.09,0.18) | 0.07(0.00,2.84) | 0.24(0.13,0.40) | 0.06(0.00,1.26) | 1.78(1.43,2.14) | -0.64(-0.97,-0.30) |
| Gambia | female | 0.00(0.00,0.00) | 0.00(0.00,2.87) | 0.00(0.00,0.00) | 0.00(0.00,0.94) | 1.81(-0.36,4.02) | -1.01(-3.12,1.15) |
| Georgia | female | 1.88(1.50,2.34) | 0.17(0.02,0.68) | 1.36(1.03,1.77) | 0.24(0.01,1.30) | 0.20(-0.90,1.31) | 2.47(1.34,3.61) |
| Germany | female | 160.53(148.51,174.59) | 1.06(0.90,1.24) | 127.07(111.27,143.24) | 0.98(0.82,1.18) | -1.49(-1.88,-1.10) | -0.87(-1.14,-0.60) |
| Ghana | female | 2.50(1.48,3.85) | 0.09(0.01,0.31) | 4.15(2.60,6.63) | 0.06(0.02,0.15) | 1.67(1.02,2.33) | -1.55(-2.14,-0.97) |
| Greece | female | 7.74(6.80,8.74) | 0.41(0.17,0.83) | 13.25(11.61,15.00) | 0.91(0.48,1.59) | 1.12(0.15,2.10) | 2.04(1.32,2.76) |
| Greenland | female | 0.23(0.17,0.29) | 1.80(0.00,49.56) | 0.25(0.16,0.34) | 2.39(0.00,48.69) | 0.35(-0.11,0.80) | 1.37(0.87,1.87) |
| Grenada | female | 0.04(0.04,0.05) | 0.28(0.00,28.73) | 0.08(0.06,0.10) | 0.40(0.00,22.24) | 0.19(-1.11,1.51) | -0.40(-1.39,0.60) |
| Guam | female | 0.03(0.03,0.04) | 0.12(0.00,14.13) | 0.00(0.00,0.00) | 0.01(0.00,14.51) | -9.64(-11.71,-7.52) | -9.23(-11.24,-7.19) |
| Guatemala | female | 10.98(9.55,12.47) | 0.72(0.36,1.34) | 33.14(26.81,40.00) | 0.92(0.63,1.31) | 4.88(3.89,5.89) | 1.78(0.79,2.78) |
| Guinea | female | 0.01(0.01,0.01) | 0.00(0.00,0.41) | 0.01(0.01,0.01) | 0.00(0.00,0.17) | 1.53(-0.72,3.85) | -1.27(-3.36,0.87) |
| Guinea-Bissau | female | 0.00(0.00,0.00) | 0.00(0.00,2.47) | 0.00(0.00,0.00) | 0.00(0.00,1.02) | 0.67(-1.47,2.85) | -2.14(-4.14,-0.09) |
| Guyana | female | 0.10(0.07,0.12) | 0.06(0.00,2.93) | 0.46(0.30,0.67) | 0.30(0.00,3.31) | 2.54(1.13,3.96) | 2.97(1.58,4.37) |
| Haiti | female | 4.34(2.89,6.64) | 0.35(0.10,0.89) | 11.60(7.28,17.65) | 0.41(0.21,0.73) | 3.63(3.36,3.90) | 0.75(0.57,0.93) |
| Honduras | female | 3.69(2.64,5.03) | 0.42(0.10,1.20) | 6.36(3.22,10.34) | 0.28(0.11,0.61) | 1.29(0.94,1.64) | -1.88(-2.22,-1.54) |
| Hungary | female | 3.16(2.64,3.72) | 0.17(0.04,0.51) | 2.51(2.04,3.06) | 0.18(0.03,0.63) | -0.81(-1.09,-0.54) | 0.01(-0.28,0.31) |
| Iceland | female | 1.08(0.93,1.26) | 2.10(0.07,11.63) | 2.93(2.53,3.40) | 4.84(0.97,15.27) | 3.70(3.45,3.96) | 3.15(2.90,3.40) |
| India | female | 539.45(439.62,649.66) | 0.33(0.30,0.36) | 716.39(597.78,848.79) | 0.24(0.23,0.26) | 0.06(-0.51,0.64) | -1.82(-2.32,-1.32) |
| Indonesia | female | 24.49(18.33,31.48) | 0.06(0.04,0.09) | 35.51(25.74,51.88) | 0.06(0.04,0.09) | 1.54(1.16,1.92) | 0.56(0.19,0.94) |
| Iran (Islamic Republic of) | female | 195.17(160.42,240.22) | 1.92(1.65,2.22) | 263.43(221.04,315.59) | 1.49(1.31,1.69) | 1.22(0.45,2.00) | -0.82(-1.27,-0.36) |
| Iraq | female | 21.05(14.77,28.82) | 0.66(0.40,1.03) | 54.31(36.30,84.54) | 0.67(0.51,0.88) | 2.74(2.06,3.43) | -0.29(-0.83,0.25) |
| Ireland | female | 6.66(5.82,7.52) | 0.99(0.39,2.09) | 18.54(15.54,22.17) | 2.28(1.35,3.63) | 3.98(3.24,4.72) | 2.96(2.43,3.50) |
| Israel | female | 3.69(3.28,4.14) | 0.39(0.10,1.05) | 11.57(10.03,13.03) | 0.71(0.36,1.25) | 4.05(3.64,4.45) | 2.17(1.84,2.50) |
| Italy | female | 99.65(94.81,104.91) | 0.93(0.76,1.13) | 27.60(25.45,29.69) | 0.35(0.23,0.52) | -4.74(-5.13,-4.35) | -3.74(-4.22,-3.27) |
| Jamaica | female | 0.35(0.27,0.55) | 0.07(0.00,1.14) | 0.77(0.51,1.11) | 0.13(0.00,0.93) | 1.23(0.33,2.14) | 0.55(-0.28,1.38) |
| Japan | female | 19.12(18.31,20.08) | 0.09(0.05,0.14) | 45.43(42.90,48.31) | 0.28(0.20,0.37) | 2.55(1.05,4.08) | 3.52(2.21,4.85) |
| Jordan | female | 1.25(0.93,1.64) | 0.19(0.01,1.12) | 1.77(1.27,2.37) | 0.07(0.01,0.30) | 0.05(-0.60,0.70) | -3.98(-4.70,-3.25) |
| Kazakhstan | female | 19.72(15.90,24.35) | 0.58(0.35,0.91) | 36.55(28.19,46.32) | 1.00(0.70,1.40) | 1.53(0.18,2.91) | 1.02(-0.47,2.53) |
| Kenya | female | 2.93(2.19,3.85) | 0.08(0.01,0.25) | 10.08(6.66,14.65) | 0.10(0.05,0.19) | 4.38(4.07,4.68) | 1.12(0.92,1.32) |
| Kiribati | female | 0.21(0.15,0.28) | 1.33(0.00,31.89) | 0.56(0.37,0.85) | 2.22(0.00,19.73) | 4.38(3.87,4.89) | 2.56(2.13,3.00) |
| Kuwait | female | 0.72(0.59,0.90) | 0.20(0.00,1.57) | 0.58(0.43,0.75) | 0.05(0.00,0.74) | -0.54(-1.35,0.27) | -4.89(-5.73,-4.05) |
| Kyrgyzstan | female | 5.05(3.84,6.40) | 0.56(0.18,1.36) | 10.18(7.39,13.50) | 0.74(0.36,1.37) | 3.58(2.29,4.89) | 2.15(0.85,3.46) |
| Lao People's Democratic Republic | female | 0.76(0.48,1.24) | 0.10(0.00,0.77) | 0.86(0.55,1.34) | 0.05(0.00,0.35) | -0.01(-0.26,0.25) | -2.30(-2.51,-2.09) |
| Latvia | female | 4.67(4.07,5.38) | 0.97(0.30,2.40) | 1.67(1.32,2.10) | 0.62(0.05,2.94) | -4.20(-4.95,-3.44) | -2.37(-3.03,-1.71) |
| Lebanon | female | 3.02(2.07,4.31) | 0.52(0.11,1.58) | 3.01(2.08,4.20) | 0.26(0.05,0.84) | 0.08(-0.39,0.56) | -2.44(-2.73,-2.15) |
| Lesotho | female | 0.70(0.40,1.06) | 0.23(0.00,1.86) | 1.87(1.12,2.74) | 0.45(0.05,1.84) | 5.10(4.23,5.98) | 4.14(3.25,5.04) |
| Liberia | female | 0.00(0.00,0.01) | 0.00(0.00,0.98) | 0.00(0.00,0.01) | 0.00(0.00,0.39) | 2.95(0.69,5.25) | -0.97(-3.15,1.26) |
| Libya | female | 4.25(2.94,5.79) | 0.61(0.16,1.67) | 17.28(10.71,25.25) | 1.15(0.67,1.85) | 5.34(4.74,5.95) | 2.54(2.18,2.91) |
| Lithuania | female | 6.32(5.43,7.32) | 0.90(0.34,1.96) | 2.72(2.13,3.35) | 0.65(0.12,2.28) | -3.42(-4.17,-2.67) | -1.51(-2.19,-0.83) |
| Luxembourg | female | 0.80(0.71,0.91) | 1.05(0.01,9.14) | 1.82(1.53,2.12) | 1.63(0.16,7.47) | 1.78(1.38,2.18) | 0.52(0.04,1.01) |
| Madagascar | female | 4.67(3.03,6.86) | 0.23(0.07,0.58) | 12.28(7.26,18.96) | 0.23(0.12,0.40) | 2.91(2.70,3.11) | -0.23(-0.44,-0.03) |
| Malawi | female | 2.88(1.81,4.47) | 0.17(0.03,0.55) | 5.90(3.39,9.45) | 0.16(0.06,0.37) | 2.24(2.15,2.34) | -0.34(-0.49,-0.20) |
| Malaysia | female | 1.72(1.17,2.47) | 0.05(0.00,0.20) | 2.12(1.47,3.08) | 0.03(0.00,0.12) | 0.39(0.06,0.71) | -1.46(-1.88,-1.05) |
| Maldives | female | 0.02(0.01,0.02) | 0.04(0.00,15.55) | 0.02(0.01,0.03) | 0.02(0.00,5.79) | 0.54(0.32,0.76) | -2.53(-2.70,-2.35) |
| Mali | female | 0.02(0.01,0.03) | 0.00(0.00,0.30) | 0.01(0.01,0.02) | 0.00(0.00,0.12) | 0.65(-1.69,3.05) | -2.67(-4.80,-0.49) |
| Malta | female | 0.19(0.16,0.22) | 0.28(0.00,7.00) | 0.39(0.32,0.46) | 0.55(0.00,9.17) | 3.49(3.03,3.95) | 3.28(2.86,3.71) |
| Marshall Islands | female | 0.01(0.00,0.01) | 0.08(0.00,56.95) | 0.01(0.00,0.01) | 0.05(0.00,34.61) | -0.97(-1.43,-0.51) | -2.00(-2.32,-1.67) |
| Mauritania | female | 0.00(0.00,0.00) | 0.00(0.00,1.19) | 0.00(0.00,0.00) | 0.00(0.00,0.54) | -0.15(-2.16,1.91) | -2.48(-4.46,-0.45) |
| Mauritius | female | 0.02(0.01,0.02) | 0.01(0.00,1.64) | 0.51(0.43,0.59) | 0.22(0.00,2.23) | 10.39(7.32,13.55) | 10.72(7.74,13.78) |
| Mexico | female | 57.47(54.61,60.38) | 0.31(0.24,0.41) | 68.50(57.74,79.35) | 0.26(0.20,0.33) | 0.26(-0.06,0.59) | -0.77(-1.07,-0.47) |
| Micronesia (Federated States of) | female | 0.01(0.01,0.02) | 0.08(0.00,24.24) | 0.01(0.00,0.01) | 0.04(0.00,21.34) | -2.89(-3.18,-2.60) | -2.86(-3.10,-2.62) |
| Monaco | female | 0.00(0.00,0.00) | 0.01(0.00,125.62) | 0.00(0.00,0.00) | 0.05(0.00,92.98) | 4.20(3.34,5.07) | 4.26(3.32,5.21) |
| Mongolia | female | 0.70(0.41,1.12) | 0.16(0.00,1.57) | 1.81(1.10,2.89) | 0.28(0.03,1.19) | 3.26(2.47,4.05) | 1.99(1.34,2.63) |
| Montenegro | female | 0.14(0.10,0.18) | 0.11(0.00,3.36) | 0.09(0.07,0.12) | 0.09(0.00,4.49) | -1.10(-1.77,-0.43) | -0.57(-1.22,0.08) |
| Morocco | female | 32.06(22.10,44.31) | 0.63(0.43,0.91) | 55.45(34.24,91.94) | 0.75(0.56,0.98) | 1.45(0.82,2.08) | 0.23(-0.27,0.73) |
| Mozambique | female | 2.69(1.59,4.25) | 0.12(0.02,0.37) | 8.13(4.47,13.64) | 0.15(0.06,0.30) | 3.87(3.74,4.00) | 1.09(0.94,1.24) |
| Myanmar | female | 13.00(8.29,19.41) | 0.15(0.08,0.26) | 6.37(4.25,9.70) | 0.06(0.02,0.12) | -3.26(-3.71,-2.81) | -3.99(-4.35,-3.63) |
| Namibia | female | 0.93(0.64,1.32) | 0.33(0.01,2.38) | 1.30(0.74,2.18) | 0.25(0.01,1.22) | 0.97(0.83,1.12) | -0.91(-1.10,-0.73) |
| Nauru | female | 0.00(0.00,0.00) | 0.10(0.00,211.86) | 0.00(0.00,0.00) | 0.06(0.00,180.88) | -2.12(-2.50,-1.75) | -2.43(-2.75,-2.12) |
| Nepal | female | 7.32(4.64,10.92) | 0.19(0.08,0.41) | 10.79(6.89,16.99) | 0.15(0.07,0.27) | 1.30(1.12,1.49) | -0.82(-1.05,-0.60) |
| Netherlands | female | 6.64(5.82,7.64) | 0.22(0.09,0.48) | 13.95(12.05,16.36) | 0.52(0.28,0.89) | 1.98(1.70,2.27) | 2.62(2.37,2.88) |
| New Zealand | female | 3.86(3.41,4.32) | 0.56(0.15,1.46) | 16.02(14.14,18.15) | 1.79(1.02,2.96) | 3.76(3.27,4.25) | 3.09(2.64,3.55) |
| Nicaragua | female | 1.03(0.78,1.33) | 0.13(0.00,0.89) | 1.51(1.08,2.06) | 0.11(0.01,0.46) | 1.11(0.78,1.45) | -0.74(-0.97,-0.50) |
| Niger | female | 0.01(0.01,0.02) | 0.00(0.00,0.38) | 0.01(0.00,0.01) | 0.00(0.00,0.13) | 0.19(-1.94,2.35) | -2.98(-5.05,-0.87) |
| Nigeria | female | 0.11(0.07,0.17) | 0.00(0.00,0.03) | 0.11(0.06,0.18) | 0.00(0.00,0.01) | 1.13(-0.93,3.24) | -1.87(-3.95,0.24) |
| Niue | female | 0.00(0.00,0.00) | 0.05(0.00,1042.29) | 0.00(0.00,0.00) | 0.05(0.00,1385.99) | -3.81(-4.83,-2.79) | -2.52(-3.34,-1.70) |
| North Macedonia | female | 0.60(0.47,0.75) | 0.15(0.00,1.27) | 0.51(0.36,0.69) | 0.14(0.00,1.63) | -0.09(-0.59,0.41) | -0.14(-0.59,0.30) |
| Northern Mariana Islands | female | 0.00(0.00,0.00) | 0.01(0.00,37.03) | 0.00(0.00,0.00) | 0.00(0.00,55.00) | -5.13(-9.95,-0.05) | -2.75(-7.28,2.00) |
| Norway | female | 9.95(9.29,10.63) | 1.26(0.60,2.34) | 24.12(22.53,25.78) | 2.75(1.76,4.14) | 1.63(0.44,2.84) | 1.30(0.02,2.60) |
| Oman | female | 0.28(0.18,0.41) | 0.10(0.00,1.78) | 0.63(0.43,0.90) | 0.08(0.00,0.74) | 3.32(2.61,4.04) | -0.39(-1.03,0.27) |
| Pakistan | female | 43.00(32.36,56.73) | 0.22(0.16,0.30) | 163.88(110.35,238.17) | 0.33(0.28,0.39) | 3.83(3.41,4.25) | 0.69(0.30,1.09) |
| Palau | female | 0.00(0.00,0.00) | 0.02(0.00,121.52) | 0.00(0.00,0.00) | 0.03(0.00,179.84) | 0.48(0.02,0.94) | 2.00(1.81,2.19) |
| Palestine | female | 0.01(0.01,0.01) | 0.00(0.00,1.57) | 0.02(0.01,0.03) | 0.00(0.00,0.45) | 2.87(2.76,2.98) | -0.79(-0.94,-0.64) |
| Panama | female | 1.52(1.27,1.82) | 0.30(0.02,1.41) | 2.66(2.02,3.36) | 0.33(0.06,1.03) | 2.08(1.82,2.34) | 0.68(0.41,0.95) |
| Papua New Guinea | female | 0.37(0.15,0.65) | 0.05(0.00,0.69) | 0.47(0.28,0.76) | 0.02(0.00,0.24) | 0.14(-0.20,0.48) | -3.02(-3.38,-2.67) |
| Paraguay | female | 0.34(0.26,0.43) | 0.04(0.00,0.66) | 2.46(1.77,3.43) | 0.16(0.03,0.54) | 7.07(6.75,7.40) | 4.79(4.54,5.05) |
| Peru | female | 26.79(19.56,35.15) | 0.57(0.37,0.84) | 34.02(22.84,49.95) | 0.48(0.33,0.67) | 0.36(-0.14,0.86) | -0.91(-1.38,-0.43) |
| Philippines | female | 9.45(8.32,10.91) | 0.07(0.03,0.14) | 14.95(11.52,18.86) | 0.07(0.04,0.11) | 1.32(1.09,1.55) | -0.58(-0.80,-0.36) |
| Poland | female | 30.20(28.22,32.28) | 0.42(0.28,0.60) | 23.44(20.05,26.61) | 0.40(0.25,0.61) | -1.72(-2.42,-1.01) | -1.33(-2.17,-0.47) |
| Portugal | female | 8.24(7.34,9.20) | 0.44(0.19,0.85) | 10.77(9.32,12.23) | 0.70(0.34,1.29) | 0.18(-0.19,0.55) | 0.88(0.57,1.19) |
| Puerto Rico | female | 3.92(3.39,4.53) | 0.54(0.14,1.40) | 3.95(3.04,5.04) | 0.73(0.20,1.94) | -2.42(-3.94,-0.87) | -1.48(-2.88,-0.06) |
| Qatar | female | 0.08(0.06,0.12) | 0.12(0.00,7.30) | 0.31(0.20,0.46) | 0.07(0.00,1.70) | 4.77(3.92,5.62) | -2.05(-2.71,-1.39) |
| Republic of Korea | female | 19.67(15.80,23.87) | 0.19(0.12,0.30) | 15.90(12.54,21.05) | 0.19(0.11,0.33) | -1.74(-2.52,-0.97) | -0.94(-1.66,-0.21) |
| Republic of Moldova | female | 4.95(4.28,5.76) | 0.55(0.18,1.35) | 2.87(2.16,3.91) | 0.45(0.08,1.63) | -2.69(-3.25,-2.12) | -1.83(-2.39,-1.25) |
| Romania | female | 31.54(23.67,38.85) | 0.74(0.50,1.05) | 12.20(9.52,15.41) | 0.46(0.24,0.81) | -3.29(-3.57,-3.01) | -1.74(-2.00,-1.49) |
| Russian Federation | female | 454.75(430.30,521.75) | 1.57(1.43,1.72) | 535.12(463.60,603.38) | 2.08(1.90,2.27) | -0.06(-1.23,1.12) | 0.07(-1.06,1.22) |
| Rwanda | female | 3.93(2.56,5.69) | 0.31(0.08,0.84) | 3.88(2.37,6.21) | 0.15(0.04,0.38) | -0.44(-0.85,-0.03) | -3.42(-3.86,-2.97) |
| Saint Kitts and Nevis | female | 0.03(0.03,0.04) | 0.39(0.00,53.39) | 0.03(0.02,0.04) | 0.25(0.00,38.84) | -2.14(-2.86,-1.42) | -3.24(-3.93,-2.54) |
| Saint Lucia | female | 0.01(0.01,0.02) | 0.05(0.00,18.25) | 0.03(0.02,0.04) | 0.09(0.00,13.80) | 0.89(-0.24,2.02) | 0.15(-0.82,1.13) |
| Saint Vincent and the Grenadines | female | 0.00(0.00,0.00) | 0.00(0.00,24.07) | 0.00(0.00,0.00) | 0.01(0.00,19.03) | -1.40(-2.71,-0.07) | -1.25(-2.52,0.03) |
| Samoa | female | 0.01(0.01,0.02) | 0.05(0.00,16.99) | 0.01(0.01,0.02) | 0.03(0.00,11.50) | -0.94(-1.36,-0.51) | -1.58(-1.95,-1.21) |
| San Marino | female | 0.00(0.00,0.00) | 0.04(0.00,85.84) | 0.00(0.00,0.00) | 0.06(0.00,90.91) | 1.87(1.29,2.46) | 2.37(1.89,2.85) |
| Sao Tome and Principe | female | 0.00(0.00,0.00) | 0.01(0.00,25.35) | 0.00(0.00,0.01) | 0.01(0.00,9.53) | 2.95(2.24,3.66) | 0.41(-0.25,1.07) |
| Saudi Arabia | female | 0.48(0.31,0.71) | 0.02(0.00,0.22) | 2.61(1.69,3.95) | 0.03(0.01,0.12) | 6.73(6.09,7.38) | 2.50(1.97,3.04) |
| Senegal | female | 0.01(0.01,0.02) | 0.00(0.00,0.36) | 0.01(0.01,0.01) | 0.00(0.00,0.15) | 0.32(-1.76,2.45) | -2.14(-4.24,0.00) |
| Serbia | female | 5.27(3.92,7.06) | 0.30(0.10,0.70) | 3.28(2.41,4.37) | 0.23(0.05,0.67) | -1.78(-1.99,-1.57) | -1.03(-1.22,-0.85) |
| Seychelles | female | 0.06(0.05,0.08) | 0.43(0.00,36.34) | 0.06(0.05,0.08) | 0.36(0.00,23.89) | 0.42(0.08,0.76) | 0.04(-0.45,0.54) |
| Sierra Leone | female | 0.01(0.00,0.01) | 0.00(0.00,0.58) | 0.01(0.00,0.01) | 0.00(0.00,0.26) | 2.78(0.41,5.20) | 0.01(-2.06,2.12) |
| Singapore | female | 0.55(0.48,0.61) | 0.07(0.00,0.76) | 0.80(0.70,0.93) | 0.08(0.00,0.88) | 0.40(-0.90,1.71) | -0.86(-2.01,0.31) |
| Slovakia | female | 2.84(2.28,3.49) | 0.28(0.05,0.88) | 2.14(1.63,2.69) | 0.25(0.03,1.02) | -0.15(-0.47,0.16) | 0.30(-0.01,0.61) |
| Slovenia | female | 1.14(1.00,1.29) | 0.30(0.01,1.67) | 0.61(0.49,0.76) | 0.22(0.00,2.16) | -1.79(-2.40,-1.16) | -0.88(-1.44,-0.31) |
| Solomon Islands | female | 0.02(0.01,0.04) | 0.04(0.00,8.98) | 0.03(0.02,0.05) | 0.03(0.00,3.13) | 0.67(0.33,1.00) | -1.83(-2.16,-1.49) |
| Somalia | female | 2.05(1.18,3.33) | 0.16(0.02,0.58) | 5.22(3.07,8.46) | 0.16(0.05,0.36) | 3.83(3.52,4.14) | -0.17(-0.34,-0.01) |
| South Africa | female | 148.09(127.45,174.53) | 1.92(1.62,2.26) | 133.25(110.64,159.76) | 1.06(0.89,1.26) | -0.63(-2.31,1.08) | -1.97(-3.60,-0.32) |
| South Sudan | female | 1.10(0.60,1.99) | 0.11(0.00,0.70) | 2.11(1.18,3.65) | 0.14(0.02,0.51) | 2.45(2.03,2.87) | 0.57(-0.01,1.15) |
| Spain | female | 65.40(58.66,72.71) | 0.89(0.68,1.13) | 43.08(36.02,49.93) | 0.67(0.48,0.91) | -2.03(-2.33,-1.74) | -1.80(-2.08,-1.51) |
| Sri Lanka | female | 18.15(14.50,22.89) | 0.49(0.29,0.78) | 8.94(5.74,12.90) | 0.22(0.10,0.41) | -3.28(-3.87,-2.69) | -3.59(-4.19,-2.99) |
| Sudan | female | 30.61(19.26,45.83) | 0.82(0.55,1.18) | 86.23(46.78,138.34) | 0.95(0.76,1.18) | 3.31(3.10,3.52) | 0.45(0.26,0.64) |
| Suriname | female | 0.07(0.04,0.09) | 0.09(0.00,6.46) | 0.24(0.17,0.34) | 0.22(0.00,4.01) | 2.71(1.92,3.51) | 1.42(0.72,2.12) |
| Sweden | female | 8.82(7.86,9.94) | 0.60(0.27,1.16) | 35.26(29.31,41.98) | 2.18(1.51,3.05) | 5.26(4.88,5.64) | 4.96(4.58,5.34) |
| Switzerland | female | 32.89(27.81,37.51) | 2.49(1.71,3.55) | 19.64(17.33,22.32) | 1.39(0.83,2.22) | -2.07(-2.26,-1.88) | -2.31(-2.52,-2.11) |
| Syrian Arab Republic | female | 20.05(14.67,26.60) | 0.88(0.53,1.40) | 29.74(20.34,42.55) | 1.09(0.71,1.63) | 1.87(1.05,2.70) | 1.16(0.79,1.54) |
| Taiwan (Province of China) | female | 68.14(60.18,76.21) | 1.49(1.16,1.89) | 18.94(15.72,22.35) | 0.48(0.28,0.78) | -4.14(-4.97,-3.31) | -3.86(-4.79,-2.92) |
| Tajikistan | female | 1.95(1.33,2.80) | 0.19(0.02,0.80) | 2.26(1.29,3.63) | 0.11(0.02,0.38) | 0.45(0.02,0.88) | -1.88(-2.26,-1.49) |
| Thailand | female | 8.36(5.92,11.12) | 0.06(0.03,0.13) | 11.30(7.65,15.83) | 0.10(0.05,0.18) | -0.18(-1.17,0.82) | 0.42(-0.45,1.29) |
| Timor-Leste | female | 0.08(0.04,0.13) | 0.05(0.00,3.13) | 0.12(0.08,0.19) | 0.04(0.00,2.01) | 1.20(0.09,2.33) | -0.83(-1.74,0.09) |
| Togo | female | 0.01(0.00,0.01) | 0.00(0.00,0.71) | 0.01(0.00,0.01) | 0.00(0.00,0.23) | 1.33(-0.86,3.57) | -1.58(-3.75,0.64) |
| Tokelau | female | 0.00(0.00,0.00) | 0.08(0.00,1516.44) | 0.00(0.00,0.00) | 0.06(0.00,1645.65) | -3.52(-4.58,-2.44) | -2.76(-3.56,-1.95) |
| Tonga | female | 0.01(0.00,0.01) | 0.03(0.00,28.52) | 0.00(0.00,0.01) | 0.02(0.00,21.19) | -2.03(-2.25,-1.81) | -2.50(-2.76,-2.24) |
| Trinidad and Tobago | female | 0.21(0.19,0.24) | 0.09(0.00,1.85) | 0.68(0.48,0.96) | 0.26(0.00,2.25) | 1.73(0.38,3.09) | 1.46(0.18,2.75) |
| Tunisia | female | 6.75(5.02,9.04) | 0.42(0.16,0.91) | 13.08(8.10,19.49) | 0.56(0.29,0.98) | 2.12(1.65,2.59) | 0.87(0.60,1.15) |
| Türkiye | female | 31.27(21.10,44.65) | 0.27(0.19,0.39) | 19.50(13.91,26.20) | 0.12(0.07,0.19) | -1.55(-1.86,-1.24) | -2.53(-2.87,-2.18) |
| Turkmenistan | female | 2.41(1.90,3.05) | 0.31(0.05,1.12) | 11.71(7.43,17.36) | 1.20(0.62,2.12) | 6.88(5.93,7.84) | 6.11(5.17,7.06) |
| Tuvalu | female | 0.00(0.00,0.00) | 0.08(0.00,203.60) | 0.00(0.00,0.00) | 0.03(0.00,185.12) | -2.63(-3.09,-2.17) | -3.24(-3.59,-2.89) |
| Uganda | female | 2.16(1.18,3.66) | 0.08(0.01,0.30) | 9.71(5.30,15.40) | 0.13(0.06,0.24) | 3.69(3.05,4.34) | 0.41(-0.18,1.01) |
| Ukraine | female | 53.98(48.45,60.68) | 0.55(0.41,0.72) | 58.24(31.96,93.81) | 0.78(0.59,1.03) | -0.55(-1.45,0.36) | 0.19(-0.69,1.08) |
| United Arab Emirates | female | 0.39(0.27,0.55) | 0.13(0.00,1.75) | 1.28(0.92,1.74) | 0.13(0.00,0.79) | 5.24(4.57,5.91) | 0.14(-0.44,0.72) |
| United Kingdom | female | 110.52(107.56,114.07) | 1.04(0.86,1.26) | 413.63(398.39,428.87) | 3.52(3.19,3.89) | 4.17(3.85,4.48) | 3.85(3.51,4.20) |
| United Republic of Tanzania | female | 7.75(5.06,11.77) | 0.17(0.07,0.36) | 19.97(11.84,31.26) | 0.18(0.11,0.28) | 2.90(2.78,3.01) | -0.04(-0.13,0.06) |
| United States of America | female | 820.68(777.24,867.63) | 1.51(1.41,1.62) | 9028.83(8116.22,10007.96) | 15.65(15.33,15.98) | 8.81(8.51,9.11) | 8.70(8.32,9.08) |
| United States Virgin Islands | female | 0.11(0.08,0.15) | 0.53(0.00,20.48) | 0.04(0.03,0.07) | 0.36(0.00,37.46) | -3.00(-3.63,-2.37) | -1.35(-1.87,-0.83) |
| Uruguay | female | 0.30(0.26,0.34) | 0.05(0.00,0.76) | 1.50(1.26,1.79) | 0.25(0.02,1.10) | 5.69(4.68,6.72) | 5.46(4.46,6.46) |
| Uzbekistan | female | 5.99(4.78,7.48) | 0.14(0.05,0.32) | 21.27(15.53,28.07) | 0.31(0.19,0.48) | 5.87(5.11,6.64) | 4.10(3.44,4.78) |
| Vanuatu | female | 0.01(0.01,0.02) | 0.04(0.00,16.05) | 0.02(0.01,0.02) | 0.02(0.00,6.95) | -0.03(-0.40,0.35) | -2.53(-2.91,-2.14) |
| Venezuela (Bolivarian Republic of) | female | 5.94(4.90,7.47) | 0.15(0.05,0.33) | 10.59(6.33,15.08) | 0.22(0.11,0.40) | 2.88(2.37,3.40) | 1.74(1.44,2.04) |
| Viet Nam | female | 31.49(21.08,45.29) | 0.22(0.15,0.32) | 35.19(24.32,51.23) | 0.17(0.12,0.25) | 0.53(0.36,0.69) | -0.70(-0.95,-0.44) |
| Yemen | female | 8.10(3.39,16.64) | 0.38(0.16,0.76) | 36.09(16.80,61.54) | 0.55(0.38,0.76) | 4.99(4.46,5.53) | 0.98(0.52,1.46) |
| Zambia | female | 3.64(2.37,5.38) | 0.28(0.07,0.82) | 8.79(5.07,14.16) | 0.23(0.10,0.46) | 2.45(2.14,2.76) | -1.08(-1.31,-0.85) |
| Zimbabwe | female | 2.75(1.88,3.89) | 0.14(0.02,0.47) | 11.41(7.55,16.11) | 0.35(0.17,0.62) | 6.47(5.32,7.62) | 4.92(3.76,6.09) |
| Afghanistan | male | 18.75(12.12,29.43) | 1.77(0.97,3.08) | 147.59(100.47,213.69) | 2.66(2.23,3.15) | 6.43(5.64,7.22) | 1.47(1.34,1.60) |
| Albania | male | 13.05(10.34,16.34) | 1.81(0.96,3.13) | 10.33(7.34,14.31) | 2.00(0.97,3.74) | -0.65(-1.17,-0.12) | 0.49(-0.23,1.22) |
| Algeria | male | 103.26(72.15,144.92) | 2.21(1.80,2.70) | 294.74(186.59,421.20) | 3.24(2.88,3.64) | 3.35(2.98,3.71) | 1.04(0.85,1.23) |
| American Samoa | male | 0.04(0.02,0.05) | 0.37(0.00,46.07) | 0.05(0.03,0.07) | 0.59(0.00,50.72) | 0.25(-1.74,2.27) | 1.27(-0.79,3.37) |
| Andorra | male | 0.03(0.02,0.05) | 0.23(0.00,39.51) | 0.03(0.02,0.05) | 0.24(0.00,37.12) | -0.17(-0.70,0.36) | 0.43(0.17,0.68) |
| Angola | male | 12.77(6.75,21.44) | 0.71(0.37,1.24) | 49.88(27.77,80.95) | 0.97(0.72,1.29) | 4.74(4.49,5.00) | 1.24(1.01,1.47) |
| Antigua and Barbuda | male | 0.02(0.02,0.03) | 0.19(0.00,35.64) | 0.07(0.06,0.09) | 0.43(0.00,25.28) | 1.27(-1.53,4.15) | 0.17(-2.42,2.83) |
| Argentina | male | 4.68(3.95,5.48) | 0.08(0.02,0.19) | 17.92(14.22,22.94) | 0.20(0.12,0.32) | 5.61(5.12,6.11) | 4.17(3.72,4.63) |
| Armenia | male | 2.03(1.56,2.60) | 0.28(0.03,1.05) | 2.33(1.85,2.93) | 0.40(0.06,1.53) | 2.05(0.38,3.75) | 2.63(0.96,4.33) |
| Australia | male | 267.58(240.08,295.76) | 7.80(6.89,8.79) | 462.77(397.34,536.29) | 9.89(9.01,10.85) | 0.23(-0.67,1.14) | -0.71(-1.58,0.16) |
| Austria | male | 48.16(42.07,53.88) | 3.02(2.23,4.04) | 74.77(64.02,85.73) | 4.90(3.84,6.19) | -0.40(-1.14,0.33) | -0.18(-1.04,0.70) |
| Azerbaijan | male | 7.88(5.79,10.59) | 0.51(0.22,1.06) | 18.08(10.35,28.51) | 0.77(0.45,1.25) | 3.77(3.24,4.30) | 2.26(1.73,2.79) |
| Bahamas | male | 0.12(0.10,0.14) | 0.22(0.00,8.52) | 0.33(0.24,0.43) | 0.43(0.00,5.82) | -0.02(-1.99,2.00) | -0.82(-2.66,1.06) |
| Bahrain | male | 0.51(0.36,0.68) | 0.28(0.00,4.80) | 2.27(1.68,3.00) | 0.43(0.06,1.94) | 4.91(4.14,5.69) | 0.05(-0.56,0.65) |
| Bangladesh | male | 111.06(52.21,178.23) | 0.53(0.43,0.64) | 223.34(83.57,354.28) | 0.68(0.59,0.77) | 2.08(1.93,2.24) | 0.67(0.54,0.80) |
| Barbados | male | 0.03(0.03,0.04) | 0.07(0.00,7.34) | 0.12(0.08,0.17) | 0.23(0.00,8.66) | 0.68(-1.70,3.11) | 0.95(-1.48,3.43) |
| Belarus | male | 119.86(71.28,194.70) | 5.79(4.80,6.95) | 93.74(69.60,124.68) | 5.51(4.42,6.83) | -2.08(-3.01,-1.14) | -1.58(-2.57,-0.59) |
| Belgium | male | 33.06(29.51,37.21) | 1.70(1.17,2.40) | 72.86(61.00,85.14) | 3.91(3.06,4.94) | 0.85(0.21,1.48) | 0.95(0.25,1.67) |
| Belize | male | 0.01(0.01,0.01) | 0.04(0.00,14.40) | 0.16(0.12,0.20) | 0.18(0.00,4.94) | 4.63(1.75,7.59) | 1.40(-1.39,4.26) |
| Benin | male | 0.65(0.39,1.05) | 0.09(0.00,0.77) | 1.45(0.73,2.41) | 0.06(0.00,0.30) | 3.91(2.42,5.43) | -0.10(-1.52,1.34) |
| Bermuda | male | 0.10(0.08,0.12) | 0.73(0.00,41.18) | 0.16(0.12,0.21) | 1.62(0.00,54.99) | -2.15(-4.34,0.10) | -0.88(-3.03,1.31) |
| Bhutan | male | 0.64(0.28,1.02) | 0.45(0.00,4.68) | 1.22(0.53,1.94) | 0.65(0.03,3.48) | 2.73(2.47,2.99) | 1.25(1.15,1.34) |
| Bolivia (Plurinational State of) | male | 5.36(3.59,7.69) | 0.46(0.16,1.09) | 18.33(10.51,27.81) | 0.74(0.44,1.18) | 4.09(3.97,4.20) | 1.44(1.37,1.51) |
| Bosnia and Herzegovina | male | 3.39(2.57,4.43) | 0.34(0.08,0.96) | 1.88(1.28,2.79) | 0.36(0.04,1.48) | -1.41(-1.76,-1.06) | 0.12(-0.15,0.39) |
| Botswana | male | 1.14(0.49,1.97) | 0.55(0.02,3.14) | 2.56(1.02,4.42) | 0.47(0.08,1.53) | 2.78(2.51,3.05) | -0.45(-0.64,-0.27) |
| Brazil | male | 56.59(52.65,60.35) | 0.19(0.14,0.24) | 631.69(576.76,696.03) | 1.48(1.36,1.60) | 9.90(8.88,10.93) | 8.65(7.59,9.72) |
| Brunei Darussalam | male | 0.91(0.67,1.23) | 1.35(0.02,8.78) | 1.15(0.82,1.52) | 0.96(0.04,5.90) | -0.11(-0.64,0.42) | -2.01(-2.51,-1.51) |
| Bulgaria | male | 15.41(12.28,18.84) | 1.02(0.57,1.68) | 18.51(14.14,24.16) | 1.79(1.05,2.89) | 0.65(-0.23,1.54) | 1.71(0.97,2.46) |
| Burkina Faso | male | 0.97(0.49,1.62) | 0.07(0.00,0.48) | 1.84(0.91,3.11) | 0.05(0.00,0.19) | 3.59(2.07,5.12) | 0.13(-1.36,1.64) |
| Burundi | male | 13.26(6.98,23.79) | 1.59(0.85,2.73) | 41.57(19.37,71.65) | 1.79(1.29,2.43) | 3.70(3.05,4.35) | 0.19(0.03,0.36) |
| Cabo Verde | male | 0.14(0.09,0.22) | 0.22(0.00,12.45) | 0.23(0.15,0.38) | 0.18(0.00,3.58) | 2.16(1.87,2.45) | -0.41(-0.72,-0.10) |
| Cambodia | male | 3.52(2.42,5.33) | 0.22(0.05,0.65) | 8.97(5.72,13.45) | 0.25(0.11,0.47) | 2.78(2.61,2.95) | 0.12(-0.03,0.27) |
| Cameroon | male | 1.75(1.09,2.80) | 0.10(0.01,0.44) | 4.65(2.71,7.52) | 0.07(0.02,0.19) | 4.71(3.25,6.20) | 0.41(-1.03,1.88) |
| Canada | male | 179.99(152.91,210.28) | 3.00(2.58,3.48) | 1318.14(1163.40,1467.63) | 20.66(19.56,21.82) | 6.08(5.21,6.97) | 6.08(5.39,6.77) |
| Central African Republic | male | 3.51(1.81,6.39) | 0.78(0.18,2.25) | 7.31(3.32,13.42) | 0.79(0.32,1.64) | 2.26(2.11,2.41) | -0.05(-0.20,0.11) |
| Chad | male | 0.69(0.35,1.13) | 0.07(0.00,0.62) | 1.69(0.92,2.77) | 0.06(0.00,0.28) | 4.43(2.97,5.91) | 0.78(-0.56,2.15) |
| Chile | male | 5.56(4.63,6.51) | 0.20(0.07,0.46) | 18.67(14.12,24.92) | 0.50(0.30,0.79) | 4.48(4.06,4.90) | 3.66(3.25,4.06) |
| China | male | 11398.31(9510.23,13420.58) | 4.15(4.08,4.23) | 3922.80(3066.76,4810.80) | 1.45(1.41,1.50) | -5.53(-6.35,-4.70) | -5.33(-6.17,-4.49) |
| Colombia | male | 52.53(45.80,60.66) | 0.78(0.58,1.03) | 71.53(56.72,88.32) | 0.69(0.54,0.88) | 0.57(-0.27,1.41) | -0.65(-1.47,0.17) |
| Comoros | male | 1.02(0.35,1.78) | 1.73(0.05,9.80) | 3.36(1.87,5.52) | 2.39(0.55,6.65) | 3.44(3.04,3.83) | 0.56(0.09,1.03) |
| Congo | male | 3.91(2.22,6.31) | 0.98(0.25,2.70) | 11.64(6.53,19.43) | 1.14(0.58,2.01) | 3.58(3.38,3.78) | 0.46(0.28,0.65) |
| Cook Islands | male | 0.01(0.01,0.02) | 0.28(0.00,118.15) | 0.01(0.01,0.02) | 0.44(0.00,148.22) | 0.53(-0.24,1.31) | 2.03(1.11,2.96) |
| Costa Rica | male | 2.89(2.28,3.45) | 0.47(0.09,1.47) | 8.24(6.75,10.02) | 0.87(0.38,1.73) | 1.93(1.12,2.75) | 0.63(-0.12,1.40) |
| Croatia | male | 20.10(16.27,24.45) | 2.14(1.30,3.33) | 19.94(15.66,25.05) | 2.92(1.77,4.63) | 0.22(-0.80,1.25) | 1.09(0.01,2.19) |
| Cuba | male | 9.21(6.18,11.68) | 0.40(0.18,0.78) | 1.97(1.55,2.42) | 0.10(0.01,0.41) | -6.55(-7.18,-5.93) | -5.55(-6.00,-5.10) |
| Cyprus | male | 5.09(3.80,6.78) | 3.16(1.04,7.46) | 8.79(6.52,11.57) | 3.23(1.42,6.77) | 1.88(1.48,2.28) | -0.20(-0.58,0.18) |
| Czechia | male | 18.25(15.24,22.03) | 0.98(0.58,1.55) | 25.57(20.41,30.49) | 1.60(1.03,2.41) | 1.17(0.70,1.64) | 1.47(1.11,1.84) |
| C?te d'Ivoire | male | 2.19(1.35,3.49) | 0.09(0.01,0.35) | 3.82(2.06,6.09) | 0.07(0.02,0.18) | 2.79(1.55,4.06) | 0.15(-1.27,1.60) |
| Democratic People's Republic of Korea | male | 59.94(37.67,92.59) | 1.55(1.18,2.00) | 52.80(33.67,82.96) | 0.94(0.70,1.24) | -0.99(-1.25,-0.73) | -2.03(-2.31,-1.74) |
| Democratic Republic of the Congo | male | 43.62(21.50,71.12) | 0.70(0.50,0.94) | 144.24(67.36,246.40) | 0.88(0.74,1.03) | 3.99(3.59,4.40) | 0.84(0.47,1.21) |
| Denmark | male | 92.49(75.22,114.88) | 9.18(7.41,11.28) | 77.41(65.41,89.33) | 8.09(6.39,10.14) | -1.23(-1.60,-0.87) | -0.82(-1.14,-0.49) |
| Djibouti | male | 1.17(0.52,2.06) | 1.68(0.07,8.74) | 6.58(3.19,10.89) | 2.32(0.90,4.90) | 5.52(5.26,5.79) | 0.86(0.69,1.03) |
| Dominica | male | 0.02(0.01,0.02) | 0.12(0.00,31.97) | 0.08(0.06,0.11) | 0.62(0.00,29.43) | 2.55(0.91,4.22) | 2.99(1.38,4.63) |
| Dominican Republic | male | 1.04(0.78,1.39) | 0.08(0.00,0.48) | 2.07(1.32,3.12) | 0.09(0.01,0.32) | 1.70(0.72,2.69) | -0.10(-1.00,0.81) |
| Ecuador | male | 6.86(5.46,8.42) | 0.35(0.14,0.75) | 39.47(30.74,50.73) | 1.08(0.77,1.47) | 6.12(5.61,6.64) | 4.04(3.54,4.55) |
| Egypt | male | 10.41(7.67,13.75) | 0.10(0.05,0.19) | 52.21(37.26,71.23) | 0.25(0.18,0.32) | 6.19(5.73,6.66) | 3.62(3.21,4.04) |
| El Salvador | male | 15.01(11.60,19.02) | 1.61(0.89,2.72) | 14.61(10.50,19.33) | 1.22(0.67,2.07) | -0.55(-0.94,-0.16) | -1.26(-1.64,-0.89) |
| Equatorial Guinea | male | 0.49(0.26,0.88) | 0.82(0.00,8.81) | 3.88(2.05,6.63) | 1.14(0.29,3.19) | 7.27(7.06,7.48) | 1.13(1.04,1.22) |
| Eritrea | male | 7.73(4.20,14.22) | 1.67(0.71,3.36) | 30.26(14.73,52.97) | 2.40(1.62,3.42) | 5.00(4.78,5.23) | 1.08(0.97,1.19) |
| Estonia | male | 19.39(16.08,22.93) | 6.63(4.01,10.39) | 29.19(22.92,34.73) | 12.84(8.51,18.90) | 1.76(0.35,3.18) | 2.27(0.83,3.73) |
| Eswatini | male | 0.48(0.25,0.80) | 0.43(0.00,4.84) | 1.31(0.81,1.96) | 0.56(0.03,2.74) | 3.23(3.01,3.44) | 0.92(0.84,1.01) |
| Ethiopia | male | 26.66(17.30,47.53) | 0.40(0.27,0.59) | 87.66(47.14,123.32) | 0.51(0.41,0.62) | 3.69(3.56,3.83) | 0.60(0.50,0.71) |
| Fiji | male | 0.53(0.37,0.75) | 0.32(0.00,3.28) | 0.30(0.21,0.44) | 0.17(0.00,2.40) | -3.47(-5.02,-1.90) | -3.81(-5.38,-2.21) |
| Finland | male | 62.39(55.30,69.12) | 6.25(4.78,8.08) | 112.95(98.77,127.45) | 12.75(10.49,15.38) | 2.51(2.16,2.86) | 2.77(2.36,3.17) |
| France | male | 239.11(209.00,270.86) | 2.15(1.89,2.45) | 305.57(248.05,371.88) | 3.02(2.69,3.38) | 0.13(-0.27,0.52) | 0.45(0.06,0.84) |
| Gabon | male | 1.91(1.14,2.89) | 1.06(0.12,4.08) | 4.07(2.19,6.53) | 1.24(0.34,3.21) | 2.14(1.95,2.32) | 0.19(0.03,0.36) |
| Gambia | male | 0.18(0.10,0.30) | 0.10(0.00,3.09) | 0.44(0.25,0.70) | 0.09(0.00,1.19) | 3.65(2.23,5.08) | 0.64(-0.75,2.06) |
| Georgia | male | 5.87(4.83,7.25) | 0.55(0.20,1.23) | 5.84(4.15,7.88) | 0.94(0.33,2.17) | 1.44(-0.01,2.92) | 3.22(1.64,4.81) |
| Germany | male | 733.09(667.91,806.39) | 4.46(4.14,4.81) | 588.88(511.67,678.13) | 4.01(3.69,4.36) | -2.08(-2.51,-1.65) | -1.44(-1.77,-1.12) |
| Ghana | male | 0.00(0.00,0.00) | 0.00(0.00,0.17) | 0.01(0.01,0.01) | 0.00(0.00,0.06) | 4.27(4.07,4.47) | 1.10(0.93,1.27) |
| Greece | male | 41.98(36.30,48.83) | 2.22(1.60,3.00) | 98.02(84.86,115.00) | 6.53(5.28,7.99) | 0.59(-1.18,2.39) | 1.39(-0.08,2.88) |
| Greenland | male | 0.26(0.17,0.39) | 1.71(0.00,44.15) | 0.39(0.26,0.55) | 3.48(0.00,50.61) | 1.15(0.94,1.35) | 2.71(2.46,2.96) |
| Grenada | male | 0.05(0.04,0.06) | 0.33(0.00,28.83) | 0.29(0.23,0.36) | 1.42(0.00,23.15) | 1.75(-0.48,4.02) | 0.97(-0.97,2.96) |
| Guam | male | 0.32(0.21,0.43) | 0.88(0.00,13.81) | 0.05(0.03,0.07) | 0.17(0.00,13.77) | -6.29(-7.84,-4.73) | -5.58(-7.10,-4.04) |
| Guatemala | male | 32.06(27.86,36.42) | 2.47(1.68,3.51) | 96.76(79.71,115.56) | 3.09(2.50,3.79) | 3.79(3.00,4.60) | 0.65(-0.19,1.50) |
| Guinea | male | 0.71(0.35,1.13) | 0.08(0.00,0.61) | 1.49(0.78,2.47) | 0.06(0.00,0.32) | 3.37(1.90,4.87) | 0.53(-0.87,1.95) |
| Guinea-Bissau | male | 0.18(0.11,0.30) | 0.11(0.00,3.21) | 0.33(0.19,0.55) | 0.08(0.00,1.36) | 3.38(1.99,4.79) | 0.53(-0.83,1.91) |
| Guyana | male | 0.03(0.02,0.03) | 0.02(0.00,2.90) | 0.25(0.19,0.33) | 0.17(0.00,3.34) | 2.72(0.32,5.18) | 3.26(0.94,5.63) |
| Haiti | male | 3.07(2.03,4.56) | 0.29(0.06,0.86) | 15.09(9.56,22.50) | 0.58(0.33,0.96) | 5.63(5.26,6.01) | 2.42(2.04,2.81) |
| Honduras | male | 14.54(10.51,18.95) | 1.89(1.04,3.20) | 37.40(18.62,63.54) | 1.88(1.32,2.61) | 2.88(2.72,3.03) | -0.30(-0.45,-0.15) |
| Hungary | male | 13.26(10.83,15.70) | 0.69(0.37,1.20) | 16.66(12.99,20.96) | 1.14(0.65,1.86) | 0.58(0.16,1.01) | 1.25(0.85,1.65) |
| Iceland | male | 2.69(2.24,3.18) | 5.01(0.91,15.84) | 8.24(7.03,9.52) | 12.55(5.47,25.42) | 3.66(3.17,4.16) | 2.91(2.48,3.33) |
| India | male | 1073.33(917.13,1248.35) | 0.62(0.58,0.66) | 2290.26(1848.30,2714.23) | 0.73(0.70,0.76) | 2.08(1.69,2.47) | 0.10(-0.26,0.46) |
| Indonesia | male | 46.03(37.82,56.57) | 0.13(0.09,0.17) | 155.94(115.63,217.47) | 0.26(0.22,0.31) | 4.59(4.11,5.07) | 3.02(2.53,3.50) |
| Iran (Islamic Republic of) | male | 894.45(750.97,1076.35) | 9.03(8.43,9.65) | 1431.64(1246.81,1631.10) | 7.44(7.05,7.85) | 1.87(1.22,2.53) | -0.55(-0.95,-0.16) |
| Iraq | male | 72.73(47.93,107.28) | 2.16(1.68,2.74) | 256.77(165.04,392.69) | 2.92(2.57,3.30) | 3.97(3.48,4.46) | 0.81(0.57,1.04) |
| Ireland | male | 17.66(15.41,20.28) | 2.64(1.55,4.20) | 74.09(62.61,86.36) | 9.37(7.35,11.81) | 3.67(2.43,4.93) | 2.78(1.76,3.80) |
| Israel | male | 16.07(13.53,18.70) | 1.75(1.00,2.85) | 48.11(39.99,56.34) | 2.88(2.13,3.83) | 2.55(1.38,3.75) | 0.55(-0.53,1.64) |
| Italy | male | 773.41(719.05,831.76) | 7.04(6.55,7.55) | 117.70(106.18,129.40) | 1.37(1.13,1.64) | -7.29(-7.73,-6.86) | -6.53(-6.99,-6.06) |
| Jamaica | male | 0.71(0.49,0.94) | 0.17(0.00,1.35) | 1.18(0.83,1.69) | 0.20(0.01,1.04) | -1.42(-2.92,0.11) | -2.18(-3.57,-0.76) |
| Japan | male | 27.88(26.36,29.66) | 0.12(0.08,0.18) | 40.29(37.08,43.57) | 0.23(0.16,0.31) | 0.01(-1.50,1.54) | 0.79(-0.52,2.11) |
| Jordan | male | 1.78(1.29,2.29) | 0.26(0.02,1.19) | 5.08(3.68,6.89) | 0.18(0.06,0.42) | 3.07(2.90,3.24) | -1.53(-1.79,-1.27) |
| Kazakhstan | male | 63.09(48.27,81.26) | 1.85(1.42,2.37) | 190.27(134.00,246.90) | 5.01(4.32,5.79) | 3.27(1.33,5.25) | 2.72(0.56,4.92) |
| Kenya | male | 15.45(7.89,20.36) | 0.50(0.28,0.83) | 65.51(39.33,91.02) | 0.75(0.58,0.95) | 4.83(4.72,4.94) | 1.25(1.19,1.30) |
| Kiribati | male | 1.56(1.07,2.15) | 10.07(0.75,48.29) | 3.15(1.99,4.74) | 12.85(2.78,37.83) | 2.55(2.32,2.78) | 0.65(0.50,0.80) |
| Kuwait | male | 2.89(2.18,4.11) | 0.54(0.10,2.01) | 21.76(16.40,30.39) | 1.73(1.05,2.82) | 6.08(5.02,7.16) | 2.55(1.47,3.65) |
| Kyrgyzstan | male | 16.18(11.53,21.80) | 1.87(1.07,3.07) | 39.83(27.31,53.67) | 2.84(2.02,3.88) | 3.61(1.95,5.29) | 2.10(0.38,3.85) |
| Lao People's Democratic Republic | male | 1.57(1.00,2.42) | 0.23(0.02,1.03) | 3.85(2.52,5.55) | 0.24(0.06,0.63) | 2.63(2.47,2.79) | -0.17(-0.35,0.01) |
| Latvia | male | 18.40(15.54,21.66) | 3.75(2.23,5.94) | 17.25(14.40,20.48) | 5.67(3.26,9.40) | -1.48(-2.23,-0.72) | -0.02(-0.76,0.73) |
| Lebanon | male | 2.44(1.58,3.87) | 0.48(0.07,1.68) | 8.36(6.05,11.41) | 0.64(0.28,1.30) | 4.69(4.25,5.14) | 1.20(1.07,1.33) |
| Lesotho | male | 0.62(0.29,1.01) | 0.30(0.00,2.54) | 1.63(1.08,2.33) | 0.42(0.04,1.78) | 3.62(3.46,3.78) | 1.44(1.31,1.57) |
| Liberia | male | 0.40(0.22,0.66) | 0.09(0.00,1.12) | 0.71(0.32,1.26) | 0.06(0.00,0.51) | 5.73(4.10,7.38) | 0.94(-0.66,2.57) |
| Libya | male | 20.17(13.45,29.04) | 2.47(1.50,3.85) | 84.49(55.47,129.30) | 5.22(4.16,6.48) | 5.30(4.99,5.61) | 2.54(2.34,2.73) |
| Lithuania | male | 26.55(22.80,31.06) | 3.73(2.45,5.46) | 36.80(30.02,43.04) | 8.12(5.70,11.39) | 0.76(0.02,1.50) | 2.52(1.76,3.30) |
| Luxembourg | male | 4.89(4.30,5.57) | 6.25(1.96,16.18) | 5.00(4.32,5.77) | 4.20(1.32,10.77) | -1.23(-1.78,-0.68) | -2.45(-3.02,-1.88) |
| Madagascar | male | 22.02(12.24,37.26) | 1.25(0.78,1.91) | 76.99(40.37,125.92) | 1.71(1.35,2.14) | 3.80(3.62,3.98) | 0.72(0.55,0.90) |
| Malawi | male | 18.45(8.37,33.20) | 1.37(0.81,2.16) | 67.35(34.48,114.00) | 2.31(1.79,2.94) | 4.30(4.19,4.41) | 1.60(1.52,1.69) |
| Malaysia | male | 10.54(6.96,14.06) | 0.29(0.14,0.54) | 19.44(13.18,26.52) | 0.26(0.16,0.40) | 1.48(1.24,1.72) | -0.89(-1.11,-0.67) |
| Maldives | male | 0.07(0.04,0.11) | 0.19(0.00,14.96) | 1.12(0.70,1.64) | 0.53(0.01,5.66) | 10.02(9.62,10.43) | 3.75(3.24,4.26) |
| Mali | male | 1.03(0.52,1.70) | 0.07(0.00,0.46) | 2.41(1.08,3.89) | 0.06(0.01,0.22) | 4.17(2.58,5.79) | 0.39(-1.07,1.88) |
| Malta | male | 1.37(1.17,1.61) | 1.95(0.11,9.55) | 3.16(2.55,3.86) | 4.31(0.89,14.31) | 2.59(1.98,3.20) | 2.06(1.49,2.64) |
| Marshall Islands | male | 0.05(0.03,0.07) | 0.53(0.00,53.29) | 0.04(0.02,0.06) | 0.32(0.00,32.60) | -0.86(-1.02,-0.71) | -1.86(-2.17,-1.54) |
| Mauritania | male | 0.37(0.23,0.56) | 0.10(0.00,1.44) | 0.53(0.29,0.87) | 0.07(0.00,0.71) | 2.33(0.85,3.83) | -0.18(-1.59,1.25) |
| Mauritius | male | 0.29(0.24,0.36) | 0.11(0.00,1.80) | 9.04(7.28,10.98) | 3.80(1.74,7.32) | 8.96(6.26,11.72) | 9.25(6.64,11.92) |
| Mexico | male | 124.83(116.68,133.63) | 0.76(0.63,0.91) | 263.30(232.07,302.63) | 1.04(0.92,1.18) | 1.09(0.31,1.87) | -0.21(-0.93,0.51) |
| Micronesia (Federated States of) | male | 0.11(0.07,0.17) | 0.54(0.00,22.78) | 0.07(0.04,0.11) | 0.32(0.00,20.56) | -1.83(-2.09,-1.57) | -2.02(-2.30,-1.74) |
| Monaco | male | 0.01(0.01,0.01) | 0.18(0.00,119.88) | 0.04(0.02,0.06) | 0.78(0.00,89.36) | 4.16(3.19,5.15) | 4.44(3.46,5.43) |
| Mongolia | male | 1.97(0.95,3.36) | 0.47(0.05,2.01) | 8.68(5.61,12.84) | 1.30(0.58,2.58) | 6.27(4.98,7.57) | 4.74(3.57,5.92) |
| Montenegro | male | 0.50(0.35,0.69) | 0.38(0.00,3.72) | 0.52(0.34,0.75) | 0.49(0.00,4.87) | 0.52(0.27,0.76) | 1.09(0.82,1.36) |
| Morocco | male | 95.71(65.11,139.29) | 2.00(1.61,2.45) | 218.92(118.98,361.62) | 2.94(2.57,3.36) | 2.46(2.28,2.63) | 0.98(0.84,1.12) |
| Mozambique | male | 10.51(5.66,16.49) | 0.60(0.29,1.08) | 53.52(32.42,87.02) | 1.30(0.97,1.70) | 5.84(5.67,6.00) | 2.86(2.77,2.95) |
| Myanmar | male | 15.49(10.27,23.76) | 0.19(0.11,0.32) | 24.03(15.61,34.80) | 0.22(0.14,0.33) | 1.00(0.81,1.20) | 0.13(-0.06,0.31) |
| Namibia | male | 0.97(0.42,1.65) | 0.41(0.01,2.70) | 2.55(1.23,4.38) | 0.52(0.09,1.72) | 3.04(2.95,3.14) | 0.72(0.61,0.83) |
| Nauru | male | 0.01(0.01,0.02) | 0.54(0.00,208.80) | 0.01(0.00,0.01) | 0.33(0.00,182.97) | -1.68(-1.99,-1.37) | -2.02(-2.36,-1.67) |
| Nepal | male | 14.79(8.75,22.67) | 0.43(0.24,0.72) | 38.12(20.79,56.92) | 0.63(0.45,0.88) | 3.02(2.94,3.09) | 1.29(1.21,1.38) |
| Netherlands | male | 40.94(33.79,48.37) | 1.29(0.92,1.76) | 72.92(60.59,88.16) | 2.64(2.07,3.33) | 1.14(0.73,1.55) | 1.90(1.57,2.23) |
| New Zealand | male | 13.14(11.13,15.39) | 1.91(1.02,3.27) | 34.83(29.83,41.00) | 3.61(2.51,5.07) | 2.41(1.99,2.84) | 1.62(1.30,1.93) |
| Nicaragua | male | 2.07(1.54,2.65) | 0.32(0.04,1.27) | 4.33(3.10,5.75) | 0.31(0.09,0.77) | 1.97(1.36,2.58) | -0.57(-1.09,-0.05) |
| Niger | male | 0.88(0.40,1.56) | 0.07(0.00,0.49) | 1.92(0.67,3.50) | 0.04(0.00,0.20) | 3.70(2.22,5.20) | -0.03(-1.44,1.39) |
| Nigeria | male | 13.26(8.10,18.68) | 0.08(0.04,0.14) | 27.53(11.88,41.23) | 0.07(0.04,0.10) | 3.79(2.35,5.26) | 0.85(-0.63,2.36) |
| Niue | male | 0.00(0.00,0.00) | 0.57(0.00,1031.69) | 0.00(0.00,0.00) | 0.35(0.00,1428.26) | -3.57(-4.10,-3.03) | -2.06(-2.36,-1.76) |
| North Macedonia | male | 3.47(2.37,5.13) | 0.85(0.20,2.34) | 4.74(3.03,7.34) | 1.15(0.35,3.02) | 1.69(1.01,2.38) | 1.39(0.76,2.03) |
| Northern Mariana Islands | male | 0.07(0.04,0.10) | 0.59(0.00,44.19) | 0.02(0.01,0.03) | 0.24(0.00,49.75) | -6.95(-9.67,-4.14) | -5.13(-7.74,-2.44) |
| Norway | male | 45.76(41.58,50.11) | 5.47(4.00,7.33) | 83.02(75.66,90.93) | 8.76(6.97,10.91) | 0.51(-0.74,1.77) | 0.15(-1.19,1.50) |
| Oman | male | 2.86(1.72,4.39) | 0.49(0.09,1.66) | 8.35(5.57,12.12) | 0.45(0.18,1.15) | 4.38(3.96,4.81) | 0.29(-0.10,0.67) |
| Pakistan | male | 118.99(75.30,156.28) | 0.58(0.48,0.69) | 352.19(245.38,494.52) | 0.72(0.64,0.80) | 3.47(3.40,3.54) | 0.55(0.47,0.63) |
| Palau | male | 0.00(0.00,0.00) | 0.00(0.00,114.81) | 0.00(0.00,0.00) | 0.00(0.00,132.96) | 0.80(0.44,1.15) | 1.28(1.04,1.52) |
| Palestine | male | 0.18(0.11,0.27) | 0.05(0.00,1.72) | 0.59(0.41,0.84) | 0.06(0.00,0.54) | 3.43(3.17,3.69) | -0.14(-0.32,0.04) |
| Panama | male | 2.46(1.98,2.99) | 0.50(0.08,1.72) | 5.95(4.44,7.57) | 0.71(0.26,1.56) | 1.64(0.92,2.37) | 0.14(-0.52,0.80) |
| Papua New Guinea | male | 2.86(0.95,5.63) | 0.33(0.06,1.09) | 5.18(2.55,8.89) | 0.23(0.08,0.56) | 1.59(1.16,2.01) | -1.54(-1.97,-1.12) |
| Paraguay | male | 0.41(0.31,0.55) | 0.05(0.00,0.65) | 5.51(3.73,7.83) | 0.35(0.12,0.80) | 10.13(9.45,10.82) | 7.55(6.92,8.18) |
| Peru | male | 20.11(14.97,26.26) | 0.48(0.29,0.75) | 51.48(37.07,70.23) | 0.68(0.51,0.90) | 3.62(3.06,4.19) | 1.82(1.25,2.39) |
| Philippines | male | 23.37(20.48,27.06) | 0.19(0.12,0.29) | 47.98(38.97,58.47) | 0.20(0.15,0.27) | 1.82(1.52,2.13) | -0.31(-0.60,-0.02) |
| Poland | male | 115.20(105.60,125.74) | 1.54(1.27,1.86) | 138.16(124.85,153.65) | 2.13(1.78,2.54) | 0.01(-0.78,0.80) | 0.11(-0.82,1.04) |
| Portugal | male | 46.00(38.96,53.90) | 2.47(1.81,3.30) | 34.93(28.69,41.51) | 2.29(1.59,3.21) | -3.40(-4.26,-2.54) | -2.75(-3.59,-1.89) |
| Puerto Rico | male | 7.55(6.46,8.71) | 1.16(0.48,2.33) | 42.70(34.48,52.26) | 8.23(5.95,11.12) | -0.74(-3.88,2.51) | 0.04(-3.00,3.18) |
| Qatar | male | 0.56(0.35,0.85) | 0.29(0.00,5.53) | 3.69(2.26,5.31) | 0.25(0.04,1.78) | 8.31(7.24,9.39) | -0.67(-1.18,-0.17) |
| Republic of Korea | male | 29.88(24.96,35.84) | 0.28(0.19,0.40) | 16.32(12.58,20.99) | 0.18(0.10,0.30) | -3.95(-4.80,-3.09) | -3.46(-4.28,-2.65) |
| Republic of Moldova | male | 13.12(11.08,15.26) | 1.51(0.80,2.62) | 14.87(10.69,18.56) | 2.12(1.15,3.73) | -0.08(-0.57,0.42) | 0.35(-0.17,0.87) |
| Romania | male | 78.41(49.60,109.81) | 1.80(1.42,2.26) | 46.16(35.34,59.77) | 1.63(1.19,2.19) | -2.36(-2.62,-2.10) | -1.00(-1.31,-0.70) |
| Russian Federation | male | 1895.77(1725.33,2017.18) | 6.20(5.92,6.49) | 3313.67(2966.64,3656.56) | 12.20(11.78,12.64) | 1.20(-0.21,2.63) | 1.29(-0.16,2.76) |
| Rwanda | male | 22.14(12.56,35.43) | 1.96(1.23,2.97) | 56.91(27.97,97.96) | 2.35(1.78,3.05) | 3.19(2.51,3.86) | 0.21(0.02,0.40) |
| Saint Kitts and Nevis | male | 0.01(0.00,0.01) | 0.08(0.00,54.51) | 0.02(0.01,0.03) | 0.15(0.00,38.60) | -0.74(-3.11,1.69) | -2.08(-4.32,0.21) |
| Saint Lucia | male | 0.03(0.02,0.03) | 0.11(0.00,19.61) | 0.14(0.11,0.18) | 0.40(0.00,13.49) | 1.58(-1.00,4.24) | 0.38(-2.01,2.82) |
| Saint Vincent and the Grenadines | male | 0.02(0.01,0.02) | 0.09(0.00,23.78) | 0.07(0.05,0.09) | 0.32(0.00,19.31) | -0.87(-3.21,1.54) | -0.75(-2.97,1.52) |
| Samoa | male | 0.17(0.10,0.25) | 0.47(0.00,17.26) | 0.11(0.07,0.18) | 0.28(0.00,11.61) | -1.76(-2.24,-1.28) | -1.96(-2.34,-1.57) |
| San Marino | male | 0.01(0.01,0.02) | 0.26(0.00,86.31) | 0.01(0.01,0.02) | 0.27(0.00,87.10) | 0.29(-0.03,0.61) | 0.83(0.53,1.14) |
| Sao Tome and Principe | male | 0.05(0.02,0.08) | 0.22(0.00,30.27) | 0.24(0.13,0.38) | 0.52(0.00,10.19) | 5.43(5.12,5.73) | 2.79(2.53,3.05) |
| Saudi Arabia | male | 4.63(3.02,6.63) | 0.12(0.04,0.31) | 36.15(22.61,55.55) | 0.28(0.19,0.40) | 8.13(7.33,8.95) | 3.59(2.94,4.25) |
| Senegal | male | 1.22(0.71,2.05) | 0.09(0.00,0.55) | 2.57(1.35,4.28) | 0.08(0.01,0.28) | 3.99(2.52,5.48) | 1.09(-0.43,2.62) |
| Serbia | male | 29.89(21.63,40.24) | 1.61(1.08,2.31) | 30.25(21.35,41.07) | 1.84(1.24,2.65) | 0.56(-0.14,1.26) | 1.14(0.29,2.00) |
| Seychelles | male | 0.04(0.03,0.05) | 0.26(0.00,30.33) | 0.19(0.14,0.26) | 0.79(0.00,23.52) | 3.66(2.12,5.24) | 2.14(0.69,3.61) |
| Sierra Leone | male | 0.51(0.27,0.89) | 0.07(0.00,0.74) | 1.04(0.49,1.78) | 0.06(0.00,0.36) | 4.48(2.44,6.57) | 0.84(-0.81,2.53) |
| Singapore | male | 1.56(1.30,1.87) | 0.20(0.02,0.89) | 2.54(2.07,3.09) | 0.24(0.03,0.99) | -0.49(-1.64,0.68) | -1.87(-2.91,-0.83) |
| Slovakia | male | 13.09(9.14,18.61) | 1.26(0.67,2.17) | 11.58(7.30,17.70) | 1.24(0.62,2.31) | -0.26(-0.53,0.00) | -0.16(-0.37,0.05) |
| Slovenia | male | 8.55(6.83,10.60) | 2.15(0.95,4.24) | 8.70(6.64,11.20) | 2.77(1.21,5.63) | 0.17(-0.42,0.77) | 0.69(0.13,1.26) |
| Solomon Islands | male | 0.26(0.13,0.47) | 0.39(0.00,8.94) | 0.38(0.22,0.62) | 0.28(0.00,3.73) | 0.65(0.31,0.99) | -1.52(-1.89,-1.15) |
| Somalia | male | 17.42(8.05,35.47) | 1.37(0.81,2.19) | 47.65(19.72,95.12) | 1.52(1.12,2.02) | 4.61(3.92,5.30) | 0.26(0.14,0.38) |
| South Africa | male | 125.56(101.39,150.18) | 1.76(1.46,2.10) | 185.36(157.00,220.76) | 1.47(1.27,1.70) | 0.94(0.35,1.54) | -0.93(-1.51,-0.36) |
| South Sudan | male | 10.71(5.09,19.24) | 1.25(0.61,2.27) | 20.12(10.33,35.21) | 1.63(1.00,2.52) | 2.35(1.96,2.75) | 0.82(0.62,1.03) |
| Spain | male | 439.64(385.06,504.16) | 5.82(5.29,6.39) | 183.94(157.11,216.22) | 2.77(2.38,3.21) | -4.72(-5.34,-4.10) | -4.71(-5.36,-4.06) |
| Sri Lanka | male | 68.36(52.78,85.88) | 1.90(1.48,2.41) | 35.22(24.54,49.04) | 0.90(0.62,1.25) | -4.13(-5.03,-3.23) | -4.41(-5.29,-3.51) |
| Sudan | male | 64.54(41.21,99.63) | 1.98(1.52,2.54) | 288.06(176.99,428.77) | 3.40(3.02,3.83) | 4.73(4.56,4.91) | 1.76(1.59,1.93) |
| Suriname | male | 0.20(0.13,0.27) | 0.27(0.00,7.05) | 1.32(0.90,1.91) | 1.25(0.07,5.94) | 3.39(1.30,5.53) | 2.28(0.45,4.14) |
| Sweden | male | 48.20(42.20,54.47) | 3.10(2.29,4.13) | 176.79(151.00,202.83) | 10.15(8.69,11.79) | 5.08(4.50,5.66) | 4.74(4.13,5.36) |
| Switzerland | male | 225.81(185.15,264.13) | 15.68(13.69,17.92) | 68.32(58.95,78.13) | 4.52(3.50,5.79) | -4.62(-5.09,-4.14) | -4.81(-5.19,-4.42) |
| Syrian Arab Republic | male | 20.47(15.08,27.56) | 0.93(0.56,1.47) | 23.56(16.10,33.96) | 1.24(0.73,2.05) | 1.23(-0.18,2.65) | 1.28(0.93,1.64) |
| Taiwan (Province of China) | male | 118.78(102.93,134.27) | 2.46(2.04,2.95) | 103.61(88.10,120.09) | 2.35(1.92,2.88) | -0.71(-0.98,-0.45) | -0.56(-0.86,-0.25) |
| Tajikistan | male | 4.89(3.62,6.40) | 0.50(0.15,1.26) | 16.46(8.28,27.11) | 0.77(0.44,1.25) | 4.38(4.11,4.66) | 1.70(1.55,1.86) |
| Thailand | male | 19.91(14.44,26.42) | 0.16(0.10,0.25) | 37.89(26.42,52.07) | 0.35(0.25,0.48) | 1.93(1.59,2.26) | 2.48(2.25,2.72) |
| Timor-Leste | male | 0.30(0.17,0.44) | 0.19(0.00,3.22) | 0.52(0.32,0.76) | 0.21(0.00,2.40) | 1.23(0.45,2.01) | -0.19(-0.77,0.40) |
| Togo | male | 0.55(0.33,0.88) | 0.09(0.00,0.99) | 1.05(0.56,1.75) | 0.06(0.00,0.40) | 3.21(1.86,4.58) | 0.32(-1.05,1.70) |
| Tokelau | male | 0.00(0.00,0.00) | 0.55(0.00,1580.23) | 0.00(0.00,0.00) | 0.43(0.00,1641.31) | -2.26(-2.94,-1.57) | -1.72(-2.18,-1.26) |
| Tonga | male | 0.07(0.03,0.10) | 0.37(0.00,34.44) | 0.06(0.03,0.09) | 0.30(0.00,25.20) | -1.09(-1.26,-0.91) | -0.94(-1.12,-0.76) |
| Trinidad and Tobago | male | 0.27(0.24,0.32) | 0.11(0.00,1.88) | 2.62(1.92,3.47) | 0.95(0.16,3.27) | 4.25(1.93,6.62) | 3.79(1.52,6.11) |
| Tunisia | male | 35.07(21.61,50.68) | 2.18(1.51,3.07) | 76.81(52.11,112.02) | 3.41(2.68,4.28) | 2.33(1.93,2.72) | 1.21(0.97,1.44) |
| Türkiye | male | 65.36(46.42,88.94) | 0.56(0.43,0.71) | 178.93(131.41,233.53) | 1.09(0.94,1.26) | 3.62(3.19,4.04) | 2.60(2.07,3.13) |
| Turkmenistan | male | 4.61(3.41,6.15) | 0.64(0.19,1.63) | 48.60(32.09,69.86) | 4.30(3.18,5.70) | 8.95(7.59,10.32) | 7.43(6.09,8.79) |
| Tuvalu | male | 0.01(0.01,0.01) | 0.51(0.00,245.89) | 0.01(0.01,0.01) | 0.34(0.00,155.74) | -0.13(-0.49,0.23) | -1.76(-2.03,-1.48) |
| Uganda | male | 26.43(11.01,47.58) | 1.21(0.79,1.77) | 122.33(66.18,194.72) | 2.10(1.75,2.52) | 4.72(4.56,4.88) | 1.51(1.39,1.63) |
| Ukraine | male | 285.81(228.82,340.59) | 2.86(2.54,3.22) | 382.57(248.91,548.66) | 4.80(4.32,5.33) | 0.17(-0.82,1.16) | 0.69(-0.32,1.70) |
| United Arab Emirates | male | 7.48(4.98,10.65) | 1.01(0.39,2.55) | 32.26(20.82,44.79) | 1.29(0.72,2.19) | 6.92(5.60,8.26) | 1.08(0.63,1.54) |
| United Kingdom | male | 407.34(395.13,419.99) | 3.82(3.46,4.21) | 1265.01(1212.94,1327.08) | 11.12(10.51,11.75) | 2.75(1.94,3.57) | 2.50(1.63,3.37) |
| United Republic of Tanzania | male | 67.00(31.67,114.33) | 1.97(1.52,2.50) | 238.00(115.48,406.52) | 2.71(2.38,3.08) | 4.22(4.15,4.28) | 1.02(0.94,1.11) |
| United States of America | male | 2724.53(2540.11,2911.78) | 4.96(4.78,5.16) | 22078.08(20512.00,23962.51) | 37.96(37.46,38.47) | 7.38(7.06,7.69) | 7.26(6.95,7.57) |
| United States Virgin Islands | male | 0.73(0.53,0.97) | 4.01(0.03,29.19) | 1.14(0.72,1.76) | 10.08(0.37,55.10) | 1.69(1.31,2.06) | 3.30(2.86,3.74) |
| Uruguay | male | 0.90(0.78,1.04) | 0.16(0.00,0.98) | 3.13(2.59,3.76) | 0.52(0.11,1.52) | 4.63(4.22,5.03) | 4.43(4.05,4.82) |
| Uzbekistan | male | 16.33(12.62,20.76) | 0.40(0.23,0.66) | 55.72(43.19,71.25) | 0.77(0.58,1.00) | 5.44(4.59,6.30) | 3.43(2.60,4.27) |
| Vanuatu | male | 0.12(0.07,0.19) | 0.41(0.00,16.38) | 0.17(0.10,0.27) | 0.28(0.00,7.74) | 0.66(0.36,0.96) | -1.78(-2.13,-1.43) |
| Venezuela (Bolivarian Republic of) | male | 7.50(5.65,9.35) | 0.19(0.08,0.40) | 7.85(5.39,12.49) | 0.18(0.08,0.37) | 1.66(0.44,2.89) | 0.44(-0.39,1.27) |
| Viet Nam | male | 290.40(196.05,428.03) | 2.23(1.97,2.50) | 678.46(424.94,1064.20) | 3.24(2.99,3.49) | 2.58(2.43,2.73) | 1.01(0.79,1.23) |
| Yemen | male | 26.40(13.18,49.41) | 1.34(0.88,1.98) | 145.84(76.12,241.29) | 2.29(1.93,2.70) | 5.97(5.41,6.53) | 1.74(1.32,2.17) |
| Zambia | male | 17.44(9.67,28.43) | 1.76(1.03,2.82) | 85.44(45.83,145.04) | 2.70(2.16,3.34) | 5.40(5.22,5.58) | 1.37(1.27,1.47) |
| Zimbabwe | male | 11.30(7.46,15.98) | 0.68(0.34,1.25) | 24.68(16.63,37.01) | 0.89(0.57,1.33) | 1.95(1.37,2.53) | 0.35(-0.28,0.98) |
